# Leptomeningeal fibroblasts promote glioblastoma progression by regulating cerebrospinal fluid dynamics

**DOI:** 10.1038/s41467-026-76032-2

**Published:** 2026-08-26

**Authors:** Maoyuan Sun, Shan Jiang, Wenli Zhang, Zhen Fan, Beining Liu, Yue Wang, Zhen Li, Yun Guan, Yulai Zeng, Jiawen Chen, Ruize Zhu, Weiqiu Ping, Yanlin Teng, Songlin Yan, Qisheng Tang, Yudan Chi, Zhiyong Qin, Houshi Xu, Chong Liu, Ying Mao, Zhifeng Shi

**Affiliations:** 1 Department of Neurosurgery, Huashan Hospital, Fudan University, Shanghai, China; 2 National Center for Neurological Disorders, Shanghai, China; 3 Neurosurgical Institute, Fudan University, Shanghai, China; 4 Department of Radiology, Second Affiliated Hospital of Chongqing Medical University, Chongqing, China; 5 Department of Neurosurgery, Shanghai General Hospital, Shanghai Jiaotong University, Shanghai, China; 6 Department of Neurosurgery of the Second Affiliated Hospital, Zhejiang University School of Medicine, Hangzhou, China; 7 Jinfeng Laboratory, Chongqing, China

**Keywords:** CNS cancer, Tumour immunology, Oncology

## Abstract

Cerebrospinal fluid contributes to homeostasis in the central nervous system, but how its dynamics are altered in glioblastoma is unclear. We find that glioblastoma drives leptomeningeal perivascular fibrosis that is associated with impaired fluid transport and clearance. Lineage tracing and single-cell analyses in male tumor-bearing mice identify leptomeningeal fibroblasts as the principal source of this fibrotic response, with limited contribution from pericytes. Fibrosis is linked to activation of nuclear factor kappa B signaling in collagen-producing fibroblasts, extracellular matrix deposition around perivascular spaces, reduced intratumoural cytotoxic T cell accumulation and a less permissive immune microenvironment. Here, we show that inhibiting nuclear factor kappa B signaling in leptomeningeal fibroblasts reduces fibrosis, improves fluid clearance, enhances intratumoural cytotoxic T cell accumulation and restores responsiveness to programmed cell death protein 1 blockade, identifying this pathway as a therapeutic target in glioblastoma.

## Abstract (web summary)

Cerebrospinal fluid (CSF) dynamics are implicated in glioblastoma (GBM) pathogenesis and tumor microenvironment alterations. This study identifies leptomeningeal fibroblast–driven fibrosis as a contributing factor to impaired CSF clearance in GBM, linking it to immune suppression and therapeutic resistance.

## Introduction

Glioblastoma (GBM) is a leading malignant primary central nervous system (CNS) tumor in adults, accounting for ~50% of malignant primary brain tumors in population-based datasets^1,2^. Despite multimodal treatment including surgical resection, radiotherapy, and chemotherapy, the median overall survival remains ~14–16 months, and the 5-year survival rate remains below 17% worldwide^3–5^. The limited efficacy of current therapies reflects the highly infiltrative growth pattern of GBM, marked intratumoural heterogeneity, profound treatment resistance, and the presence of an immunosuppressive tumor immune microenvironment^6,7^. Although immune checkpoint blockade has transformed the treatment landscape of several cancers, its clinical benefit in GBM has been limited, suggesting that additional anatomical, metabolic, and immune barriers within the CNS tumor ecosystem remain incompletely understood^8,9^.

Cerebrospinal fluid (CSF) provides mechanical cushioning and nutritional support to the CNS^10,11^. However, CSF is now increasingly recognized as a dynamic regulatory system involved in the distribution of signaling molecules, removal of metabolic waste, immune surveillance, and communication between the brain parenchyma, meninges, lymphatic vessels, and peripheral immune system^12,13^. CSF circulates through the ventricular system and subarachnoid space and is cleared through multiple routes, including perivascular spaces (PVS), meningeal lymphatic vessels, arachnoid granulations, and drainage pathways leading to cervical lymph nodes or systemic circulation^14^. These clearance routes are important regulators of neuroinflammation, antigen trafficking, and CNS immune homeostasis^15,16^. Thus, disruption of CSF flow or clearance may profoundly affect GBM pathogenesis by altering not only fluid dynamics but also the biochemical and immunological milieu surrounding brain tumors. Its dysfunction may profoundly impact GBM pathogenesis^17–19^. Nevertheless, the regulation of CSF dynamics and its role in shaping the tumor immune microenvironment (TIME) of GBM have long been overlooked.

Clinical observations indicate that tumors located in the vicinity of the CSF-filled ventricles are associated with poor prognosis^20–23^, while alterations in CSF composition can influence the therapeutic sensitivity of GBM cells-phenomena that suggest a critically underappreciated role of CSF in GBM biology^17^. Accumulating evidence demonstrates CSF system dysfunction in GBM patients, with abnormal CSF composition reflecting the complex state of GBM progression^24–27^. CSF from GBM patients contains tumor-derived DNA, extracellular vesicles, cytokines, metabolites, and immune-related factors that may reflect tumor burden and disease progression^28–30^. Previous studies have reported impaired CSF outflow in GBM models^24,31^ and reduced water diffusion rates in PVS of cerebral blood vessels in GBM patients^32^, indicating that GBM may disrupt healthy CSF circulation and waste clearance through specific mechanisms. Nevertheless, how GBM regulates CSF clearance impairment and how these alterations reshape the TIME to influence treatment responses remain poorly understood.

CSF drains along the PVS of the leptomeninges,which are composed of the pia and arachnoid mater, into lymphatic vessels, ultimately draining into cervical lymph nodes or entering the systemic circulation^33–35^. The leptomeninges form a critical interface among CSF, cerebral vasculature, perivascular spaces, immune cells, and lymphatic drainage pathways^36,37^. Because CSF drains along leptomeningeal and perivascular routes before reaching lymphatic vessels and cervical lymph nodes, structural remodeling of this compartment could have broad effects on CNS fluid transport and immune communication^12,38,39^. However, whether GBM induces pathological remodeling of the leptomeningeal perivascular niche, and whether this process affects CSF clearance and antitumor immunity, remains unknown. We therefore hypothesized that GBM might induce CSF clearance dysfunction by affecting leptomeningeal structure. Here, by analyzing leptomeninges from GBM patients and experimental GBM models, we show that GBM-associated CSF promotes fibroblast activation accompanied by NF-κB/P65 signaling. This response is associated with leptomeningeal perivascular fibrosis, impaired CSF clearance, and reduced intratumoural CD8⁺ T-cell accumulation, consistent with a less immune-permissive tumor microenvironment. In murine models, fibroblast-targeted conditional deletion of P65 reduced pathological extracellular matrix deposition, improved CSF clearance, and enhanced anti-PD-1 efficacy. Collectively, these findings identify leptomeningeal perivascular fibrosis as an important contributor to CSF clearance dysfunction in GBM and support a role for NF-κB/P65 signaling in leptomeningeal fibroblasts as a potential therapeutic target for remodeling the GBM immune microenvironment.

## Results

### GBM-induced leptomeningeal fibrosis impairs CSF clearance

**Fig. 1:**
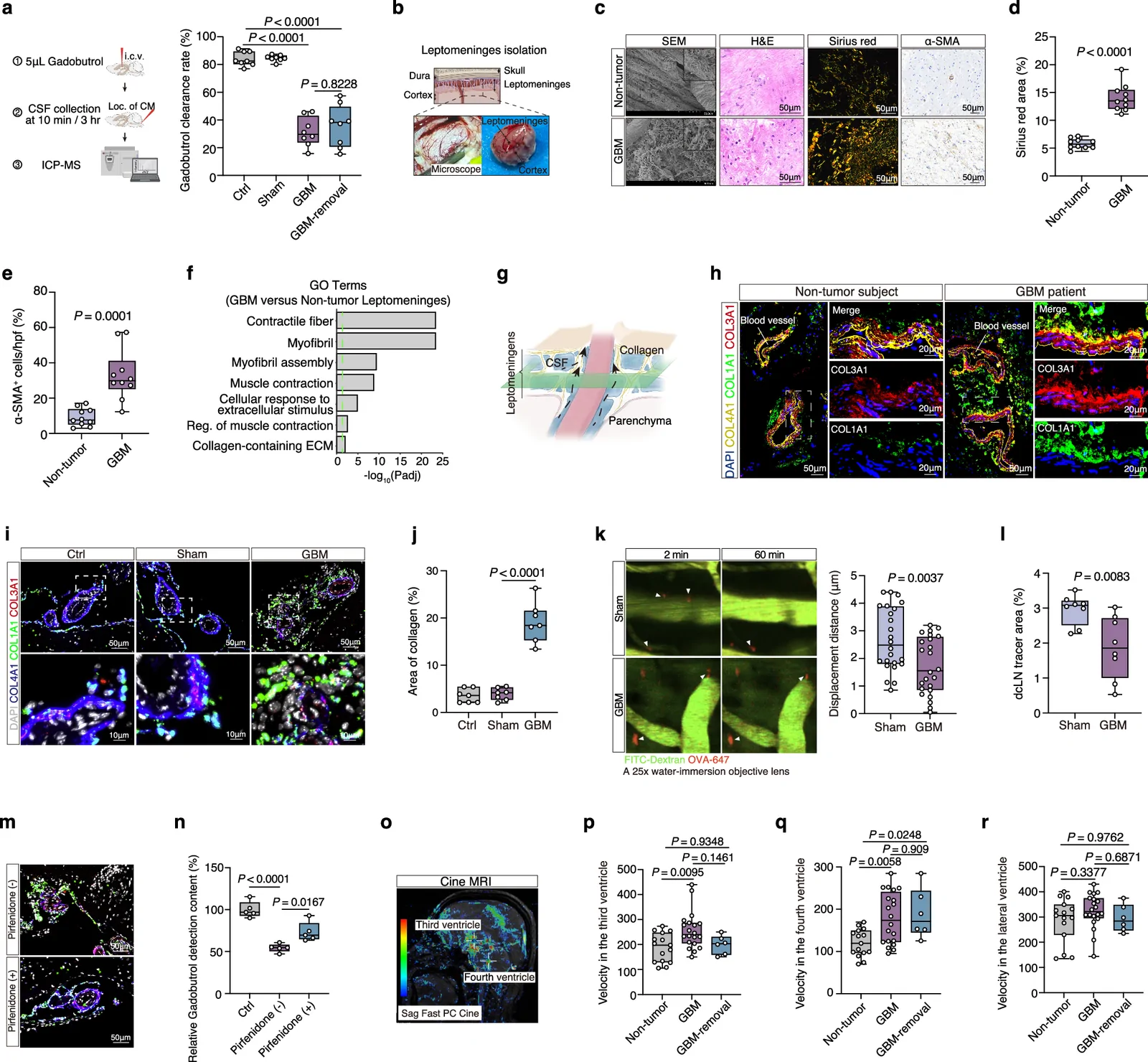
Leptomeningeal fibrosis and CSF clearance analyses in GBM. **a** CSF clearance within 3 h after intraventricular (icv) gadobutrol injection in sham, GBM-bearing and tumor-resected mice (*n* = 8 mice per group); *P* values, one-way ANOVA with Tukey’s test. Figure created with BioRender.com. cm: cisterna magna. **b** Schematic of leptomeningeal tissue sampling. Figure created with BioRender.com. **c–e** SEM, H&E, Sirius Red, and α-SMA staining and quantification in controls and GBM patients (*n* = 10 human participants per group); *P* values, unpaired two-tailed Student t-test. **f** Enrichment analysis of differentially expressed genes in leptomeningeal tissues from patients with GBM (*n* = 15 patients) and non-tumor controls (*n* = 44 control individuals); two‑sided hypergeometric test with Benjamini-Hochberg FDR correction. **g**,**h** Schematic of CSF drainage and representative multiplex immunofluorescence of perivascular spaces (representative of five human tissue samples; quantification in Supplementary Fig. 1b). Scale bar, 50 μm; zoom-in, 20 μm. **i**,**j** Representative images and quantification of PVS and type I/III collagen fibers in leptomeninges from control, sham-operated and GBM-bearing mice (*n* = 7 mice per group). Scale bar, 50 μm; zoom-in, 10 μm; *P* values, one-way ANOVA with Tukey’s test. **k** In vivo two-photon imaging of solute transport along leptomeningeal PVS after intraparenchymal ovalbumin-AF647 injection (*n* = 24 vessels from 3 mice per group; vessels were sampled from biologically independent mice); *P* values, unpaired two-tailed Student t-test. **l** Fluorescent tracer coverage in deep cervical lymph nodes (*n* = 8 mice per group); *P* values, unpaired two-tailed Student t-test. **m-n**, Representative images (m) and relative gadobutrol content (n) in vehicle- or pirfenidone-treated GBM-bearing mice (*n* = 5 mice per group). Scale bar, 50 μm; *P* values, one-way ANOVA with Tukey’s test. **o–r** Phase-contrast MRI analysis of CSF dynamics in controls (*n* = 15 human participants), patients with GBM (*n* = 20 patients) and postoperative patients with GBM (*n* = 6 patients); *P* values, one-way ANOVA with Tukey’s test. Box plots display the median (center line), 25 and 75th percentiles (box limits), minimum and maximum values (whiskers), with all individual data points shown for Fig. 1a, d, j, k, l, n, p-r. Source data are provided as a Source Data file.

Accumulating evidence indicates that GBM disrupts CSF circulation^24,31,32^. In this study, we first used the clinical contrast agent gadobutrol as a tracer to assess an overall CSF tracer clearance readout in mice after intracerebroventricular injection, by quantifying its relative abundance in cisterna magna CSF 3 h later (Fig. 1a). Relative to sham animals, tumor-bearing GBM mice exhibited a markedly reduced gadobutrol clearance-associated readout (from 84.67 to 31.47%, *P* < 0.0001) (Fig. 1a), consistent with prior studies. Notably, this abnormality persisted after surgical tumor resection (Fig. 1a and Supplementary Fig. 1a), suggesting that the defect is not solely attributable to acute tumor mass effect.

The leptomeninges represent an important interface for CSF transport and clearance pathways, and their PVS may participate in fluid and solute drainage to meningeal lymphatic vessels, cervical lymph nodes, and systemic compartments^33–35^. We therefore asked whether GBM is associated with leptomeningeal remodeling that could constrain these processes. To address this, we collected intraoperative leptomeningeal samples from GBM patients and from non-neoplastic control individuals undergoing epilepsy surgery (Fig. 1b). Scanning electron microscopy revealed striking architectural disruption in tumor-distal leptomeninges from GBM patients, including disorganization and thickened, cord-like fibrous structures (Fig. 1c). H&E staining further showed loss of the typical linear collagen organization seen in non-tumor leptomeninges (Fig. 1c). Consistently, Sirius Red quantification demonstrated increased type I/III collagen area in GBM leptomeninges (5.80 to 14.01%, *P* < 0.0001; Fig. 1c, d), and α-SMA^+^ cells were enriched (Fig. 1c, e), supporting a fibrotic, contractile stromal phenotype. To provide transcriptomic-level support for these findings, we performed bulk RNA sequencing on leptomeningeal samples from 15 GBM patients. Comparison with published non-neoplastic leptomeningeal reference datasets^40^ suggested enrichment of extracellular matrix organization, contractile programs and matrix remodeling pathways in GBM leptomeninges, further supporting prominent fibrotic remodeling in this compartment (Fig. 1f and Supplementary Dataset 1).

Given that leptomeningeal PVS constitute one important CSF drainage interface (Fig. 1g), we next examined PVS microstructure by multiplex fluorescence staining. In GBM patients, type I/III collagen (COL1A1^+^/COL3A1^+^) was deposited within ColIV^+^ PVS compared to non-tumor controls (Fig. 1h and Supplementary Fig. 1b), consistent with structural fibrotic remodeling at this drainage-associated interface. We observed a concordant phenotype in mice intracranially transplanted with GBM cells, which exhibited expanded COL1A1^+^/COL3A1^+^ signal within leptomeningeal PVS (*P* < 0.0001; Fig. 1i, j).

We next asked whether these structural changes were accompanied by functional abnormalities in brain fluid and solute drainage. To do so, we used a set of complementary assays designed to interrogate distinct but related steps of transport. Following intraparenchymal tracer injection, tracer recovery in cisterna magna CSF at 4 h, used here as a surrogate readout of parenchyma-to-CSF efflux, was significantly reduced in GBM mice (Supplementary Fig. 1c). Intravital two-photon imaging further revealed slower tracer movement within leptomeningeal PVS (Fig. 1k), consistent with impeded transport along the brain-surface perivascular compartment. Finally, after intraparenchymal injection of ovalbumin-AF647, GBM mice showed reduced tracer coverage in cervical lymph nodes compared with sham controls (*P* = 0.0083; Fig. 1l and Supplementary Fig. 1d), consistent with impaired downstream drainage. To functionally test whether fibrosis contributes to this phenotype, we administered the anti-fibrotic drug pirfenidone into the CSF of tumor-bearing mice via osmotic pump. Pirfenidone reduced leptomeningeal fibrosis (Fig. 1m) and significantly improved the parenchyma-to-CSF tracer efflux readout (Fig. 1n). Together, these data support leptomeningeal perivascular fibrosis as an important contributor to GBM-associated impairment of CSF transport and clearance.

To determine whether GBM is also associated with altered regional CSF hydrodynamics in patients^41,42^, we next assessed ventricular CSF flow by phase-contrast MRI (PC-MRI). We measured ventricular CSF velocity metrics in 15 non-tumor controls, 20 GBM patients (temporal, frontal, or thalamic tumors), and six post-operative GBM patients (Fig. 1o and Supplementary Fig. 1e, f). GBM patients exhibited higher CSF flow velocities in the third (*P* = 0.0095) and fourth ventricles (*P* = 0.0058), with no significant change in the lateral ventricles (Fig. 1o–r). Elevated fourth-ventricle velocities persisted after resection (*P* = 0.0248; Fig. 1q). Because PC-MRI primarily reports local, plane-specific CSF motion rather than whole-system CSF turnover or net clearance, these findings indicate altered regional ventricular CSF dynamics in GBM. Notably, the increased local velocity metrics observed by PC-MRI should not be taken to imply increased global CSF clearance.

### GBM-associated CSF promotes fibroblast activation and profibrotic programs

**Fig. 2:**
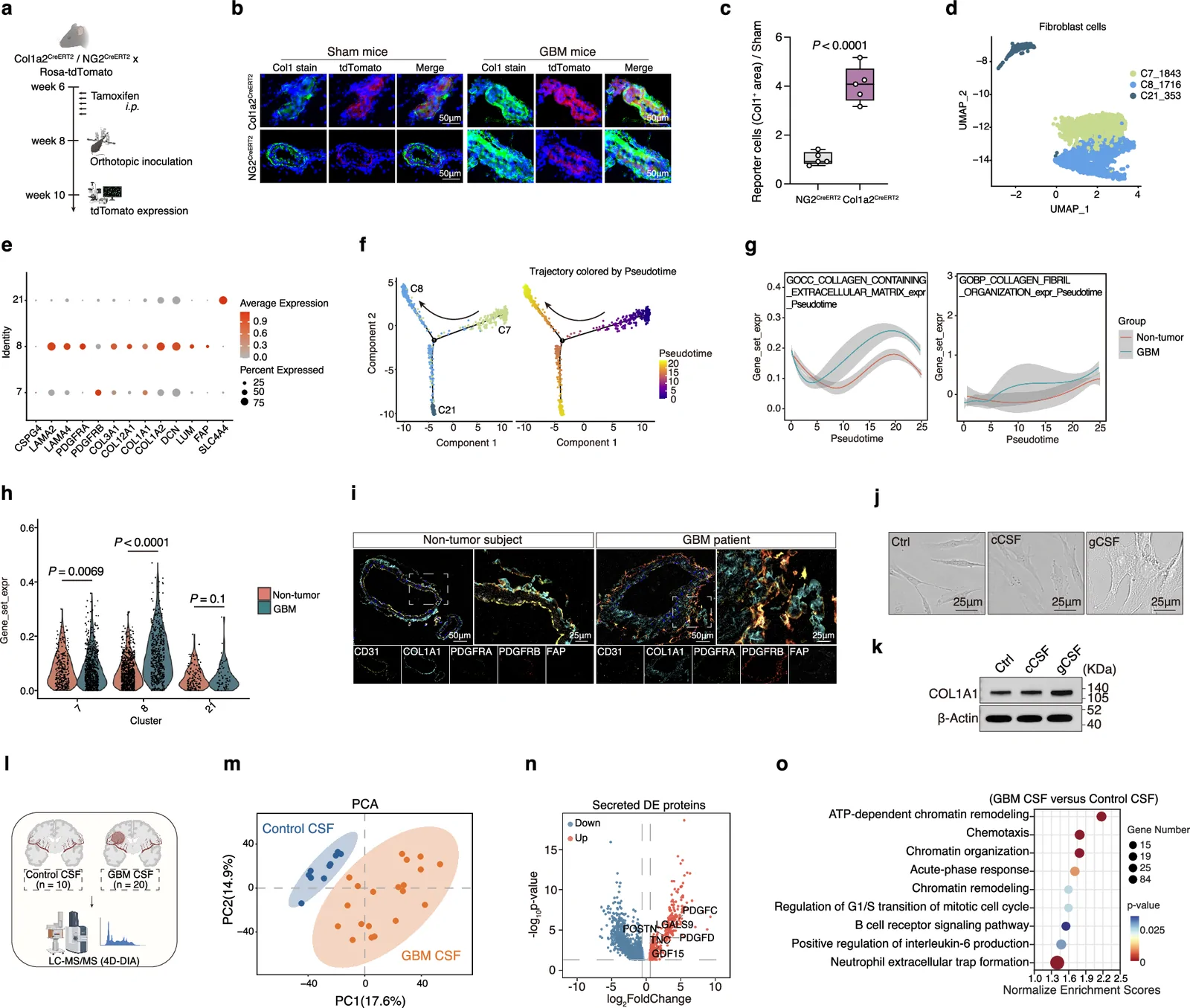
Characterization of leptomeningeal fibroblasts and fibroblast responses to GBM-derived CSF. **a** Genetic fate mapping of COL1-lineage fibroblasts and NG2-lineage mural cells in mouse leptomeninges (sham vs GBM). ip: intraperitoneal injection. **b** Representative images of the leptomeningeal interface showing tdTomato-labeled cells and COL1 immunostaining in sham-operated and GBM-bearing mice. Scale bar, 50 μm. **c** Quantification of reporter cells at the leptomeningeal interface, normalized to sham controls (*n* = 5 mice per group); *P* values, unpaired two-tailed Student *t*-test. Box plots display the median (center line), 25th and 75th percentiles (box limits), minimum and maximum values (whiskers), with all individual data points. **d** UMAP embedding of single-cell transcriptomes from leptomeningeal tissues of non-tumor controls and patients with GBM (*n* = 3 human participants per group), highlighting fibroblast clusters. **e** Feature plots showing expression of representative fibroblast marker genes in leptomeningeal single-cell transcriptomes from non-tumor controls and patients with GBM. **f** Monocle 2 pseudotime trajectory of fibroblast subclusters (clusters 7, 8 and 21), with inferred transcriptional states. **g**,**h** Gene set scores along pseudotime and across fibroblast subclusters for collagen fibril organization, collagen-containing extracellular matrix and collagen biosynthetic process (Values are gene set scores plotted; shaded areas represent mean ± SD); *P* values, two‑sided Wilcoxon tests with FDR correction. **i**, Representative multiplex immunofluorescence images of leptomeningeal tissues from non-tumor controls and patients with GBM (representative of four human tissue samples; quantification in Supplementary Fig. 2e). Scale bar, 25 μm. **j**,**k** Representative phase-contrast images and immunoblot analysis of COL1A1 in human embryonic fibroblasts treated with control CSF (cCSF) or GBM-derived CSF (gCSF); image is representative of independent cell-culture experiments with similar results. Scale bar, 25 μm. **l**, Schematic of the proteomics workflow for CSF samples from patients with GBM (*n* = 20 patients) and non-tumor controls (*n* = 10 human participants). Figure created with BioRender.com. **m–o** PCA, volcano plot, and pathway enrichment of CSF proteins; limma (*FDR* < 0.05, *FC* ≥ 1.2) and GSEA (two-sided hypergeometric test, FDR-corrected). Source data are provided as a Source Data file.

Fibroblasts are core drivers of tissue fibrosis, with their aberrant activation and phenotypic transformation directly leading to ECM overdeposition^43,44^. In the CNS, collagen-producing stromal cells implicated in fibrosis have been reported to include COL1-expressing fibroblasts and pericytes^45^. To assess the lineage contribution to GBM-associated leptomeningeal perivascular collagen deposition, we performed genetic fate mapping using Col1a2^CreERT2^; Rosa26-LSL-tdTomato and NG2(Cspg4)^CreERT2^; Rosa26-LSL-tdTomato mice (Fig. 2a). In GBM-bearing mice, we observed an approximately 3.9-fold increase in Col1a2^CreERT2^-labeled cells at the leptomeningeal interface that co-localized with COL1^+^ fibrotic regions, whereas NG2^CreERT2^-traced cells did not show a comparable increase (Fig. 2b, c). These results suggest that Col1a2-lineage fibroblasts represent a major expanding stromal population associated with leptomeningeal perivascular collagen deposition in this model, whereas contribution from NG2^+^ mural cells appears comparatively limited.

To define the cellular programs associated with GBM-related fibroblast activation, we performed single-cell RNA sequencing on leptomeningeal samples from control individuals and GBM patients. Across 26 clusters, we annotated four major putative compartments, fibroblasts, mural cells (including pericytes), immune cells and endothelial cells, based on canonical markers (Supplementary Fig. 2a–c). Fibroblasts were primarily distributed across clusters 7, 8 and 21 (Fig. 2d). All three clusters expressed fibroblast-associated markers, including COL1A1, COL1A2 and DCN, and lacked the canonical mural-cell marker CSPG4 (NG2) (Fig. 2e). Notably, cluster 7 was enriched for COL1A1 and PDGFRB, whereas cluster 8 preferentially expressed COL1A1 and PDGFRA and showed higher DCN expression (Fig. 2e), suggesting heterogeneous fibroblast-like stromal states with differential PDGFRB and PDGFRA expression that may be differentially engaged in GBM.

We next used Monocle 2 to infer relationships among fibroblast-like states across the three subclusters, identifying three pseudotime-associated states. State 3, composed largely of cluster 7 cells, exhibited a PDGFRB^+^ PDGFRA^-^ FAP^-^ profile, consistent with a less activated fibroblast-like state located near the beginning of the inferred trajectory (Fig. 2e, f). Across the inferred transition from state 3 to state 1, gene set scores for COLLAGEN_FIBRIL_ORGANIZATION and COLLAGEN_CONTAINING_EXTRACELLULAR_MATRIX progressively increased, consistent with acquisition of a more fibrogenic program (Fig. 2g). Importantly, these fibrosis-related scores were consistently higher in the GBM group than in controls (Fig. 2g and Supplementary Fig. 2d), indicating enhanced fibrogenic activation of leptomeningeal fibroblast-like cells in GBM. A COLLAGEN_BIOSYNTHETIC_PROCESS gene set further highlighted clusters 7 and 8 as the fibroblast-enriched populations with the strongest collagen biosynthetic signatures within this inferred fibrotic program (Fig. 2h), whereas cluster 21 showed substantially weaker enrichment of collagen biosynthetic pathways. Consistent with these transcriptomic findings, multiplex immunofluorescence of patient leptomeninges showed increased COL1A1 abundance together with elevated proportions of PDGFRB^+^ and FAP^+^ cells in GBM compared with controls (Fig. 2i and Supplementary Fig. 2e), supporting expansion and activation of collagen-producing leptomeningeal fibroblast-like stromal cells in GBM.

**Fig. 3:**
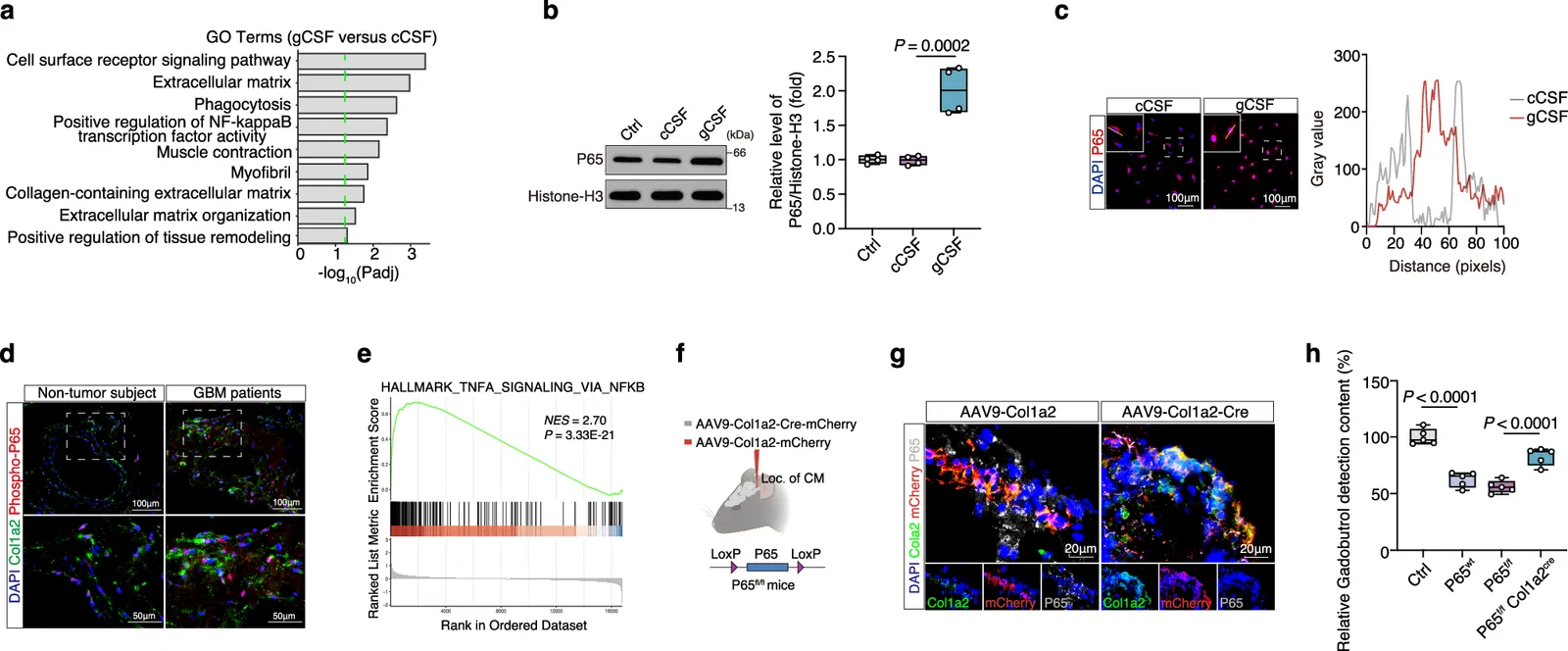
NF-κB/P65 pathway analysis and perturbation in leptomeningeal fibroblasts. **a** Gene Ontology (GO) enrichment analysis of differentially expressed genes in hPLMFs treated with cCSF or gCSF; two‑sided hypergeometric tests with Benjamini-Hochberg FDR correction. **b** Immunoblot analysis of nuclear P65 abundance in hPLMFs treated with cCSF or gCSF (*n* = 4 independent cell-culture experiments per group); *P* values, one-way ANOVA with Tukey’s test. **c** Subcellular localization of P65 in fibroblasts after treatment with cCSF or gCSF. Left, representative immunofluorescence images (representative of independent cell-culture experiments). Scale bar, 100 μm. Right, quantification of nuclear P65 localization. **d** Representative immunofluorescence images of phosphorylated P65 in fibroblast nuclei within leptomeningeal tissues from non-tumor controls and patients with GBM (representative of 5 human tissue samples; quantification in Supplementary Fig. 3d). Scale bar, 50 μm. **e** GSEA for HALLMARK_TNFA_SIGNALING_VIA_NFKB in leptomeningeal fibroblasts from patients with GBM versus non-tumor controls; two‑sided hypergeometric tests with Benjamini-Hochberg FDR correction. **f** Schematic of the strategy used for conditional deletion of P65 in Col1a2-targeted leptomeningeal fibroblast-like cells. AAV9 vectors expressing Cre-mCherry under the Col1a2 promoter (pAAV9-Col1a2-Cre-mCherry) or control mCherry vectors (pAAV9-Col1a2-mCherry) were injected into the cisterna magna (cm.) of P65^f/f^ mice. Figure created with BioRender.com. **g** Representative multiplex immunofluorescence images showing co-staining of P65, AAV9-mCherry signal and fibroblast markers in leptomeningeal tissues from mice receiving pAAV9-Col1a2-Cre-mCherry or pAAV9-Col1a2-mCherry (representative of independent mouse tissue samples). Scale bar, 20 μm. **h** Relative gadobutrol content detected in CSF from GBM tumor-bearing mice of the indicated groups (*n* = 5 mice per group); *P* values, one-way ANOVA with Tukey’s test. Box plots display the median (center line), 25 and 75th percentiles (box limits), minimum and maximum values (whiskers), with all individual data points shown for Fig. 3b, h. Source data are provided as a Source Data file.

As the liquid environment of the CNS in direct contact with the leptomeninges, CSF is intricately linked to leptomeningeal physiology^36^. Given the characteristic secretion of tumor-derived components^27^, we hypothesized that GBM patient-derived CSF (gCSF) directly promotes leptomeningeal fibroblast activation. To test this possibility, we used human embryonic fibroblasts (HEbF) as a tractable in vitro fibroblast model and exposed them to CSF from non-tumor controls (cCSF) or gCSF. gCSF stimulation induced marked morphological remodeling in HEbF cells, including cytoplasmic expansion, transition from a spindle-shaped to a stellate morphology, and acquisition of features consistent with fibroblast activation and myofibroblast-like remodeling^46^ (Fig. 2j). These changes were accompanied by elevated COL1A1 protein levels (Fig. 2k), indicating that gCSF promotes a profibrotic activation-like state in HEbF cells characterized by increased collagen production. To assess whether similar responses occur in leptomeningeal fibroblasts, we next performed bulk RNA sequencing in primary human leptomeningeal fibroblasts (hPLMFs) treated with cCSF or gCSF. Gene set enrichment analysis (GO) revealed significant enrichment of upregulated differentially expressed genes in pathways including extracellular matrix organization, phagocytosis, and positive regulation of tissue remodeling (Fig. 3a), providing transcriptomic evidence that gCSF induces profibrotic phenotypic changes in leptomeningeal fibroblasts.

To investigate how GBM modulates leptomeningeal fibroblast function via CSF remodeling, we performed quantitative proteomic profiling of CSF samples from 20 GBM patients and 10 non-tumor controls (Fig. 2l, m). This analysis identified 475 differentially expressed proteins (*fold change [FC]* ≥ 1.2; *false discovery rate [FDR]* < 0.05) (Fig. 2n and Supplementary Dataset 2). Notably, gCSF exhibited significant enrichment of secreted factors originating from tumor tissues, including platelet-derived growth factor C (PDGFC), platelet-derived growth factor D (PDGFD), and galectin-9 (LGALS9)-all of which possess chemotactic and profibrotic properties^47^(Fig. 2n and Supplementary Fig. 2f). PDGFC and PDGFD, which signal through the canonical PDGFRβ receptor abundantly expressed on perivascular fibroblasts, hint at a functional link between gCSF and leptomeningeal fibroblasts. Pathway enrichment analysis further revealed that these dysregulated proteins are predominantly implicated in acute-phase responses, G1/S phase cell cycle transitions, interleukin-6 (IL-6) production, and neutrophil extracellular trap (NET) formation (Fig. 2o), consistent with a pro-inflammatory and tissue-remodeling CSF milieu. Collectively, these data indicate that gCSF contains bioactive molecules capable of promoting profibrotic fibroblast activation, and nominate PDGFC, PDGFD and LGALS9 as candidate mediators of this response.

### NF-κB/P65 signaling contributes to profibrotic activation of leptomeningeal fibroblasts

Consistent with the profibrotic phenotypic changes induced by GBM-associated CSF in fibroblast models (Fig. 2j, k), gCSF stimulation of hPLMFs enriched transcriptional programs related to NF-κB activation (Fig. 3a and Supplementary Dataset 3). Consistently, immunoblotting showed that gCSF significantly increased nuclear abundance of P65 (RELA), a central transcriptional effector of canonical NF-κB, in hPLMFs (Fig. 3b). Immunofluorescence further revealed gCSF-induced translocation of P65 from the cytoplasm to the nucleus (Fig. 3c), supporting activation of NF-κB signaling in these cells. Given the established involvement of NF-κB in fibrosis^48,49^, we next assessed NF-κB activation in leptomeningeal fibroblast-like stromal cells from GBM patients. Compared with non-tumor controls, GBM leptomeningeal tissues exhibited markedly increased nuclear phospho-P65 within fibroblast cells (Fig. 3d and Supplementary Fig. 3a). This finding was further supported by gene set enrichment analysis of fibroblast-like cells extracted from the single-cell RNA-sequencing dataset of GBM and control leptomeningeal samples, in which HALLMARK_TNFA_SIGNALING_VIA_NFKB emerged as a representative enriched program (Fig. 3e), further supporting NF-κB pathway activation in GBM-associated leptomeningeal fibroblast-like cells.

To test whether NF-κB/P65 is required for gCSF-induced profibrotic fibroblast responses, we inhibited P65 using two complementary approaches. Pharmacological inhibition with the NF-κB/P65 inhibitor dehydroxymethylepoxyquinomicin (DHMEQ) attenuated gCSF-induced profibrotic responses, as indicated by reduced COL1A1 and COL3A1 protein levels (Supplementary Fig. 3b). In parallel, siRNA-mediated P65 knockdown (versus siNC) reduced P65 mRNA and phosphorylated P65 protein levels in gCSF-stimulated hPLMFs, without affecting cell viability (Supplementary Fig. 3c–e), and concomitantly reduced COL1A1 and COL3A1 expression (Supplementary Fig. 3f). Together, these results support a role for NF-κB/P65 signaling in mediating the profibrotic fibroblast response induced by GBM-associated CSF.

To examine the in vivo relevance of fibroblast NF-κB/P65 signaling, we combined P65-floxed mice with AAV9 vectors encoding Col1a2-driven Cre recombinase (pAAV9-Col1a2-Cre-mCherry) or a matched control lacking Cre (pAAV9-Col1a2-mCherry). Intracisternal administration of pAAV9-Col1a2-Cre-mCherry enabled conditional P65 deletion with preferential targeting of leptomeningeal Col1a2^+^ fibroblast-like cells (Fig. 3f). We first assessed the targeting pattern of this approach and found that mCherry signal was observed mainly in Col1a2^+^ leptomeningeal fibroblast-like cells, with little or no detectable overlap with endothelial, pericyte or immune-cell markers in the regions examined (Supplementary Fig. 3g). Multiplex immunofluorescence confirmed efficient reduction of P65 signal in leptomeningeal fibroblast-like cells in the pAAV9-Col1a2-Cre-mCherry group relative to the control virus group (Fig. 3g). Using this Col1a2-targeted conditional P65 deletion strategy, we then generated GBM-bearing mice for in vivo analysis. In GBM-bearing leptomeninges, P65^f/f^ mice receiving pAAV9-Col1a2-Cre-mCherry showed reduced type I/III collagen accumulation (COL1A1/COL3A1) compared with both control groups, namely P65^f/f^ mice receiving the control virus and WT mice receiving pAAV9-Col1a2-Cre-mCherry (Supplementary Fig. 3h). Functionally, the CSF clearance-associated readout was significantly improved in P65^f/f^ mice receiving pAAV9-Col1a2-Cre-mCherry compared with P65^f/f^ groups (Fig. 3h). Targeted P65 deletion in Col1a2^+^ cells did not alter the CSF clearance-associated readout in healthy mice (Supplementary Fig. 3i).

Collectively, these data support NF-κB/P65 signaling in leptomeningeal fibroblast-like cells as an important contributor to GBM-associated leptomeningeal fibrosis and suggest that this pathway participates in CSF transport and clearance dysfunction. Targeted disruption of this pathway in Col1a2^+^ leptomeningeal fibroblast-like cells reduced collagen deposition and improved the CSF clearance-associated readout.

### Improvement in the CSF clearance-associated phenotype is accompanied by delayed GBM progression and immune activation

**Fig. 4:**
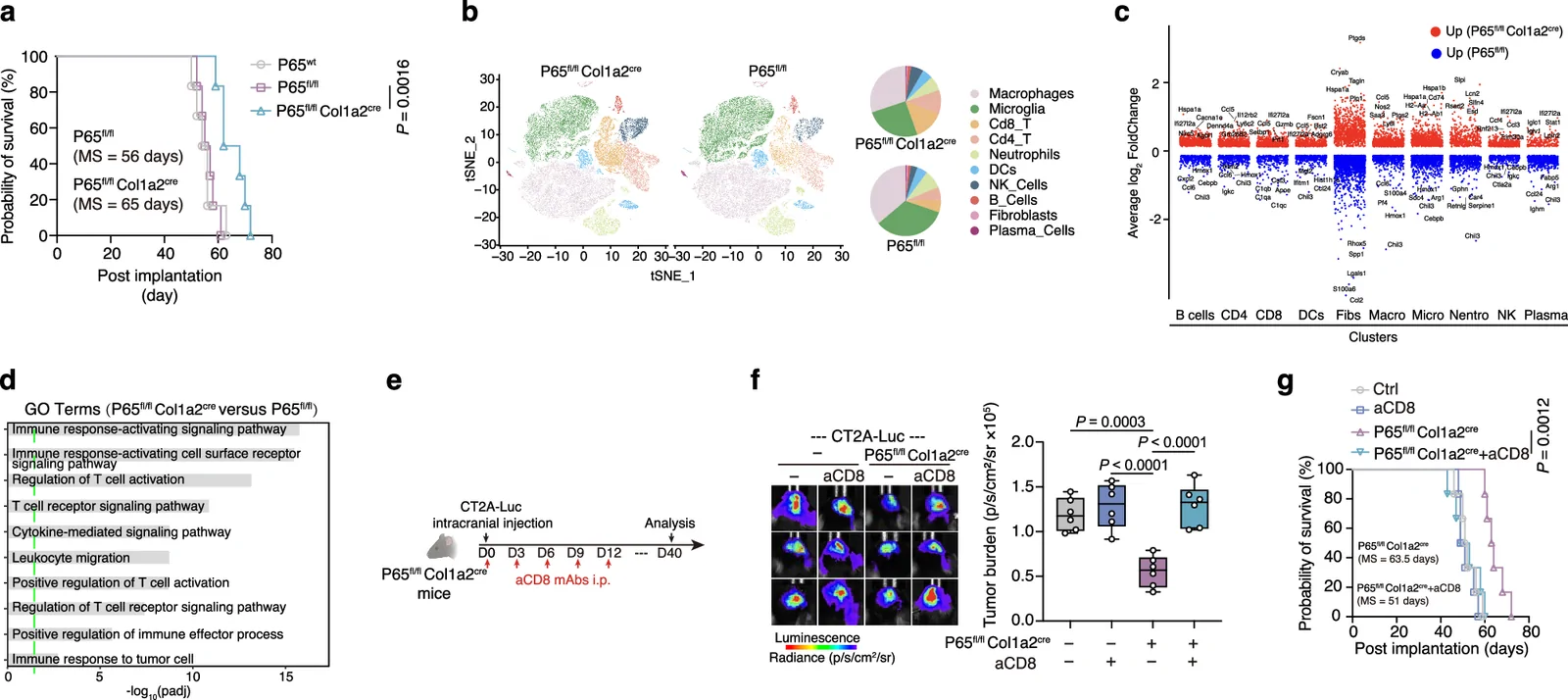
Tumor progression and immune profiling in GBM-bearing mice with fibroblast P65 deletion. **a** Kaplan-Meier survival curves of CT2A-Luc tumor-bearing mice in the P65^wt^, P65^f/f^, and P65^f/f^ + pAAV9-Col1a2-Cre-mCherry groups (*n* = 6 mice per group); Kaplan-Meier curves, and group differences were assessed with Log-rank (Mantel-Cox) tests. **b** Left: single-cell clustering analysis of CD45⁺ cells in CT2A tumor tissues of tumor-bearing mice from the P65^f/f^ and P65^f/f^ + pAAV9-Col1a2-Cre-mCherry groups, identifying ten immune cell populations annotated by canonical markers. Right: proportion of cell populations in tumor tissues of the indicated groups (*n* = 5 mice per group). **c** Gene signatures upregulated in different cell populations (B Cells, CD4_T Cells, CD8_T Cells, DC Cells, Fibroblasts, Macrophages, Microglia, Neutrophils, NK Cells, and Plasma Cells) in single-cell RNA-sequencing data from the P65^f/f^ and P65^f/f^ + pAAV9-Col1a2-Cre-mCherry groups. **d** GO enrichment analysis of differentially expressed genes in CD8 + T cells from the P65^f/f^ and P65^f/f^ + pAAV9-Col1a2-Cre-mCherry groups; two‑sided hypergeometric tests with Benjamini-Hochberg FDR correction. **e** Schematic of IgG isotype control or anti-CD8α antibody treatment in P65^f/f^ mice receiving pAAV9Col1a2-Cre-mCherry. Figure created with BioRender.com. ip: intraperitoneal injection. **f** Left, representative bioluminescence images of CT2A-Luc tumor-bearing mice in the indicated groups treated with IgG isotype control or anti-CD8α antibody. Right, quantification of tumor burden (*n* = 6 mice per group); *P* values, one-way ANOVA with Tukey’s test. Box plots display the median (center line), 25 and 75th percentiles (box limits), minimum and maximum values (whiskers), with all individual data points. **g** Kaplan-Meier survival curves of CT2A-Luc tumor-bearing P65^f/f^ mice receiving pAAV9-Col1a2-Cre-mCherry and treated with IgG isotype control or anti-CD8α antibody (*n* = 6 mice per group); Kaplan-Meier curves, and group differences were assessed with Log-rank (Mantel-Cox) tests. Source data are provided as a Source Data file.

Previous studies have demonstrated that impaired CSF clearance is associated with brain dysfunction in neurodegenerative diseases^50^, whereas its relationship to GBM progression remains less clear. We evaluated tumor progression in GBM-bearing mice across three experimental groups: WT mice, P65^f/f^ mice receiving control virus, and P65^f/f^ mice receiving pAAV9-Col1a2-Cre-mCherry. Compared with both control groups, P65^f/f^ mice receiving pAAV9-Col1a2-Cre-mCherry exhibited reduced tumor burden and significantly prolonged survival in both the CT2A and GL261 GBM models (Fig. 4a and Supplementary Fig. 4a–c). These findings indicate that targeted disruption of NF-κB/P65 in Col1a2^+^ leptomeningeal fibroblast-like cells, accompanied by improvement in the CSF clearance-associated readout, is associated with delayed GBM progression.

To explore potential mechanisms associated with the delayed GBM progression observed following targeted P65 deletion in Col1a2^+^ cells, we next examined the tumor microenvironment, given that CSF flow can influence solute trafficking, antigen drainage and immune cell priming^15,51,52^. We performed magnetic-bead sorting of CD45⁺ cells followed by single-cell RNA sequencing of tumor tissues from CT2A-bearing mice. Cells were clustered into 10 populations based on canonical markers (Supplementary Fig. 4d). Notably, the proportion of T cells was significantly increased in P65^f/f^ mice receiving pAAV9-Col1a2-Cre-mCherry (Fig. 4b). To determine whether the increased CD8⁺ T-cell signal reflected preferential vascular retention or increased accumulation within tumor tissue, we performed intravascular anti-CD45-PE labeling immediately before sacrifice. After tumor dissociation, leukocytes were defined by CD45-FITC and stratified by intravenous CD45-PE into intravascular (CD45-FITC^+^CD45-PE^+^) and extravascular/tissue (CD45-FITC^+^CD45-PE^−^) compartments. Compared with P65^f/f^ mice receiving control virus, P65^f/f^ mice receiving pAAV9-Col1a2-Cre-mCherry showed increased numbers of both intravascular and extravascular/tissue CD8⁺ T cells, with a more pronounced increase in the extravascular/tissue compartment (Supplementary Fig. 4e, f). This pattern is consistent with enhanced recruitment to the tumor vasculature together with increased accumulation within tumor tissue.

To assess whether local expansion might also contribute to the enlarged intratumoral CD8^+^ T-cell pool, we quantified Ki67 expression in tumor-infiltrating CD8^+^ T cells. We observed an increased Ki67^+^ fraction in the P65^f/f^ mice receiving pAAV9-Col1a2-Cre-mCherry group (Supplementary Fig. 4g), suggesting that local proliferation may contribute to the expanded intratumoral CD8^+^ compartment. Taken together, these data support contributions from both enhanced recruitment/entry and local expansion to the increased intratumoral CD8^+^ T-cell population.

Comparative transcriptional analysis of T cells from P65^f/f^ mice receiving control virus and P65^f/f^ mice receiving pAAV9-Col1a2-Cre-mCherry revealed increased expression of immune effector genes, including Gzmb and Ccl5, in CD8⁺ T cells from the latter group, whereas genes associated with a more immunoregulatory state, including Apoe, were relatively enriched in the control group (Fig. 4c). In addition, several immune-associated genes, including Ccl3, Ccl5 and Cd74, were elevated in other immune-cell populations in the P65^f/f^ mice receiving pAAV9-Col1a2-Cre-mCherry group (Fig. 4c).

These findings suggest that targeted P65 deletion in Col1a2⁺ cells, together with reduced leptomeningeal fibrosis and improvement in the CSF clearance-associated readout, is accompanied by a more activated immune state in the tumor region, which may contribute to delayed tumor progression. Gene set enrichment analysis of differentially expressed genes in CD8⁺ T cells further showed enrichment of pathways related to immune-response-activating cell surface receptor signaling, immune-response-activating signaling, and positive regulation of immune effector processes in the P65^f/f^ mice receiving pAAV9-Col1a2-Cre-mCherry group (Fig. 4d). These results are consistent with remodeling of the tumor immune microenvironment in association with the improved CSF clearance-associated phenotype and may help explain the delayed GBM progression.

To assess the functional contribution of T cells, we administered CD8-neutralizing antibodies to deplete CD8⁺ T cells in vivo. The survival advantage observed in P65^f/f^ mice receiving pAAV9-Col1a2-Cre-mCherry was largely abolished by CD8⁺ T-cell depletion (Fig. 4e–g and Supplementary Fig. 4h, i). Collectively, these data indicate that targeted NF-κB/P65 disruption in Col1a2⁺ leptomeningeal fibroblast-like cells, together with reduced leptomeningeal fibrosis and improvement in the CSF clearance-associated readout, is associated with enhanced antitumour immunity, including increased T-cell accumulation and activation of immune effector programs.

### A pre-established CSF clearance-associated deficit is accompanied by accelerated GBM progression and CD8^+^ T-cell dysfunction

**Fig. 5:**
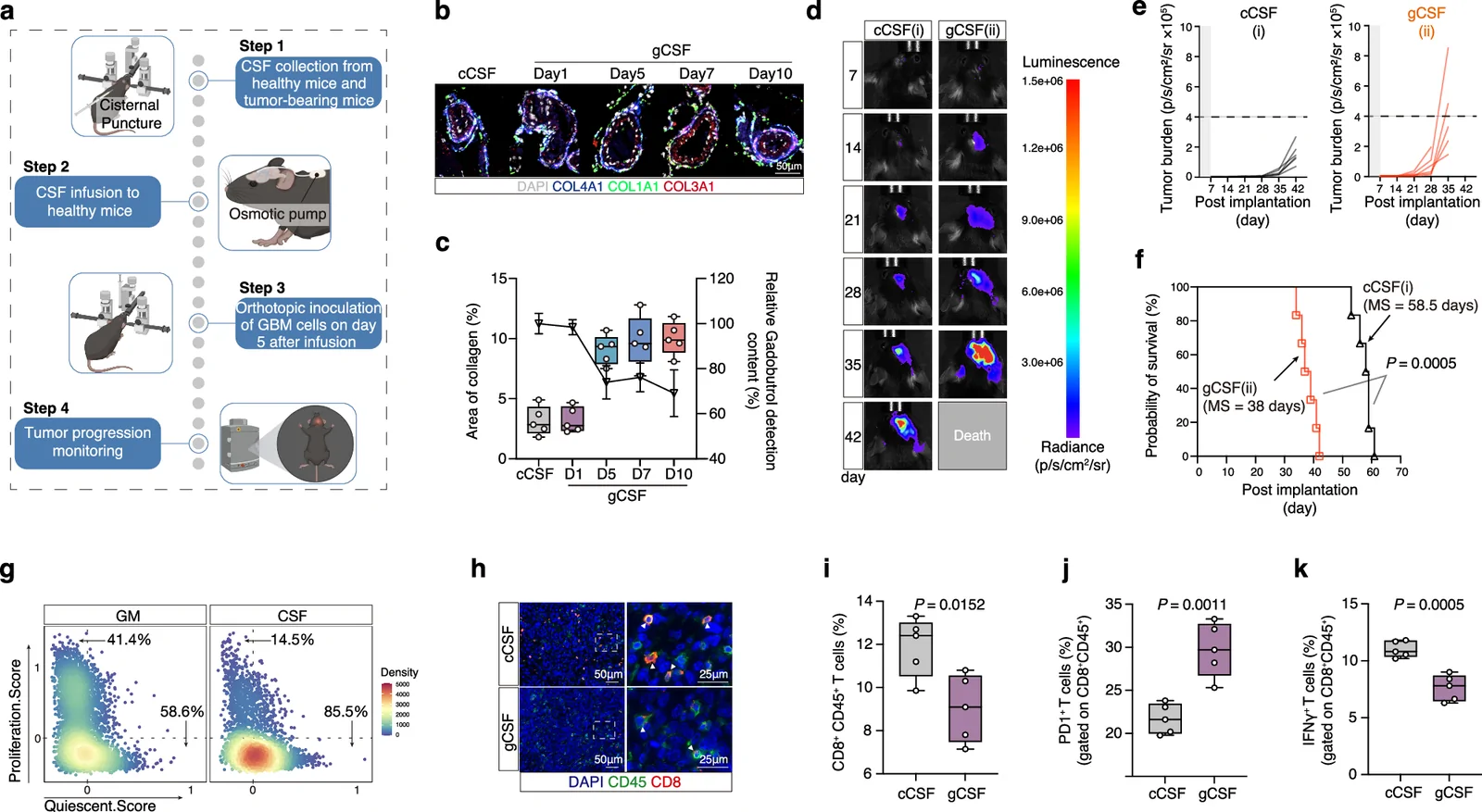
Pre-established CSF clearance impairment is associated with accelerated GBM progression and altered CD8⁺ T-cell states. **a** Schematic of the CSF infusion experiment. Osmotic pumps were used to infuse cCSF or gCSF into the lateral ventricles of healthy mice at a rate of 0.5 μL h⁻¹ (total volume: 100 μL). Orthotopic CT2A-Luc and GL261-Luc tumor models were established 5 days after completion of CSF infusion. Figure created with BioRender.com. **b** Representative fluorescence images of leptomeningeal type I/III collagen (COL1A1^+^/COL3A1^+^) in mice after completion of cCSF or gCSF infusion. Scale bar, 20 μm. **c** Quantification of perivascular leptomeningeal COL1A1^+^/COL3A1^+^ collagen area and relative gadobutrol content after completion of cCSF or gCSF infusion in mice (the right y‑axis shows mean ± s.d.; the left y‑axis uses the box‑plot convention defined, *n* = 5 mice per group). **d** Representative bioluminescence images of CT2A-Luc tumor-bearing mice in the cCSF and gCSF groups. **e** Bar graphs showing tumor burden in CT2A-Luc tumor-bearing mice of the cCSF and gCSF groups (*n* = 6 mice per group). **f** Kaplan-Meier survival curves of CT2A-Luc tumor-bearing mice in the cCSF and gCSF groups (*n* = 6 mice per group); Kaplan-Meier curves, and group differences were assessed with Log-rank (Mantel-Cox) tests. **g** Quiescent.Score and Proliferation.Score analysis of tumor cells using the GSE243501 dataset. **h** Representative fluorescence staining images of CD45⁺CD8⁺ cells in the tumor tissues of CT2A-Luc tumor-bearing mice in the cCSF and gCSF groups (representative of independent mouse tumor samples). Scale bar, 50 μm (zoom-ins, 25 μm). **i–k** Flow-cytometric quantification of CD45⁺CD8⁺ cells, PD1⁺ cells gated on CD45⁺CD8⁺ cells, and IFN-γ⁺ cells gated on CD45⁺CD8⁺ cells in tumor tissues (*n* = 5 mice per group); *P* values, unpaired two-tailed Student t-test. Box plots display the median (center line), 25th and 75th percentiles (box limits), minimum and maximum values (whiskers), with all individual data points shown for Fig. 5c, i–k. Source data are provided as a Source Data file.

To further interrogate the relationship between a pre-established CSF clearance-associated deficit and tumor progression, we established an intracerebroventricular CSF infusion model to test whether exposure to gCSF is sufficient to induce leptomeningeal fibrotic remodeling accompanied by impairment of the CSF clearance-associated readout. To determine whether the proteomic alterations identified in human GBM-associated CSF were also represented in murine GBM-associated CSF, we first examined representative proteins in CSF collected from CT2A-bearing mice. Several secreted proteins enriched in human gCSF, including Gdf15, Pdgfc, Pdgfd, Tnc, Postn and Lgals9, were similarly elevated in CSF from tumor-bearing mice (Supplementary Fig. 5a). Using osmotic pumps, we then infused either cCSF or gCSF (100 μL at 0.5 μL/h) into the lateral ventricles of healthy mice (Fig. 5a and Supplementary Fig. 5b). Mice were analyzed at the indicated time points (days 1–10) after completion of the continuous infusion. Immunofluorescence analysis showed significant expansion of COL1A1- and COL3A1-positive collagen areas in the leptomeninges of the gCSF group by day 5 after completion of infusion (Fig. 5b, c), while tracer-based analysis at the same time point demonstrated impairment of the CSF clearance-associated readout (Fig. 5c). Because this CSF clearance-associated deficit was detectable by day 5 and remained evident through day 10, day 5 was selected for subsequent tumor implantation experiments.

Using this model, we next implanted orthotopic CT2A or GL261 tumors after completion of CSF infusion to evaluate the impact of a pre-established CSF clearance deficit on tumor growth and survival (Fig. 5a). In the CT2A model, mice receiving gCSF infusion exhibited significantly accelerated tumor progression, with median survival decreasing from 58.5 days to 38 days (*P* = 0.0005) (Fig. 5d–f). Similarly, in the GL261 model, median survival decreased from 33 days in the cCSF group to 26 days in the gCSF group (*P* = 0.0012) (Supplementary Fig. 5c–e). We next asked whether gCSF exposure directly altered the proliferative state of tumor cells. Using a previously published single-cell RNA-sequencing dataset^17^, we compared quiescence- and proliferation-related transcriptional program scores in malignant cells across groups and found no evidence for higher proliferation-associated scores in the gCSF-associated condition (Fig. 5g). To further assess this in vivo, we performed a separate experiment using the CSF infusion model and analyzed tumor cells at days 3 and 21 after implantation. Proliferation was assessed at both the protein and mRNA levels by flow cytometry and qPCR for Ki-67 and representative proliferation markers (Mki67 and Top2a) (Supplementary Fig. 5f–i). No significant increase in proliferation markers was detected in the gCSF group at day 3. By day 21, however, these markers were significantly elevated in the gCSF group (Supplementary Fig. 5h, i), consistent with the increased tumor burden observed at later stages. Together, these findings suggest that, in this gCSF infusion model, accelerated tumor progression is unlikely to be explained by an immediate increase in tumor-cell proliferation and is instead more consistent with early changes in the non-tumor microenvironment, with increased tumor-cell proliferation emerging at later stages.

Building on the association between the CSF clearance-associated phenotype and immune microenvironment alterations observed in Fig. 4b–d, we next analyzed the functional state of intratumoral CD8⁺ T cells in CT2A-bearing mice from the cCSF and gCSF infusion groups. The gCSF group showed a significant reduction in CD45⁺CD8⁺ T-cell numbers, an increased proportion of CD8⁺PD1⁺ T cells with features consistent with dysfunction/exhaustion, and a decreased population of CD8⁺IFN-γ⁺ effector T cells (Fig. 5h–k and Supplementary Fig. 5j). Similar changes in T-cell state were observed in the GL261 model (Supplementary Fig. 5k, l). Collectively, these findings support a model in which a gCSF-induced state characterized by leptomeningeal fibrotic remodeling and impaired CSF clearance-associated readouts is associated with accelerated GBM progression, reduced CD8⁺ T-cell abundance, increased features of T-cell dysfunction/exhaustion, and diminished effector function.

### Targeting the NF-κB/P65 signaling pathway in leptomeningeal fibroblasts augments efficacy of immune checkpoint blockade therapy

**Fig. 6:**
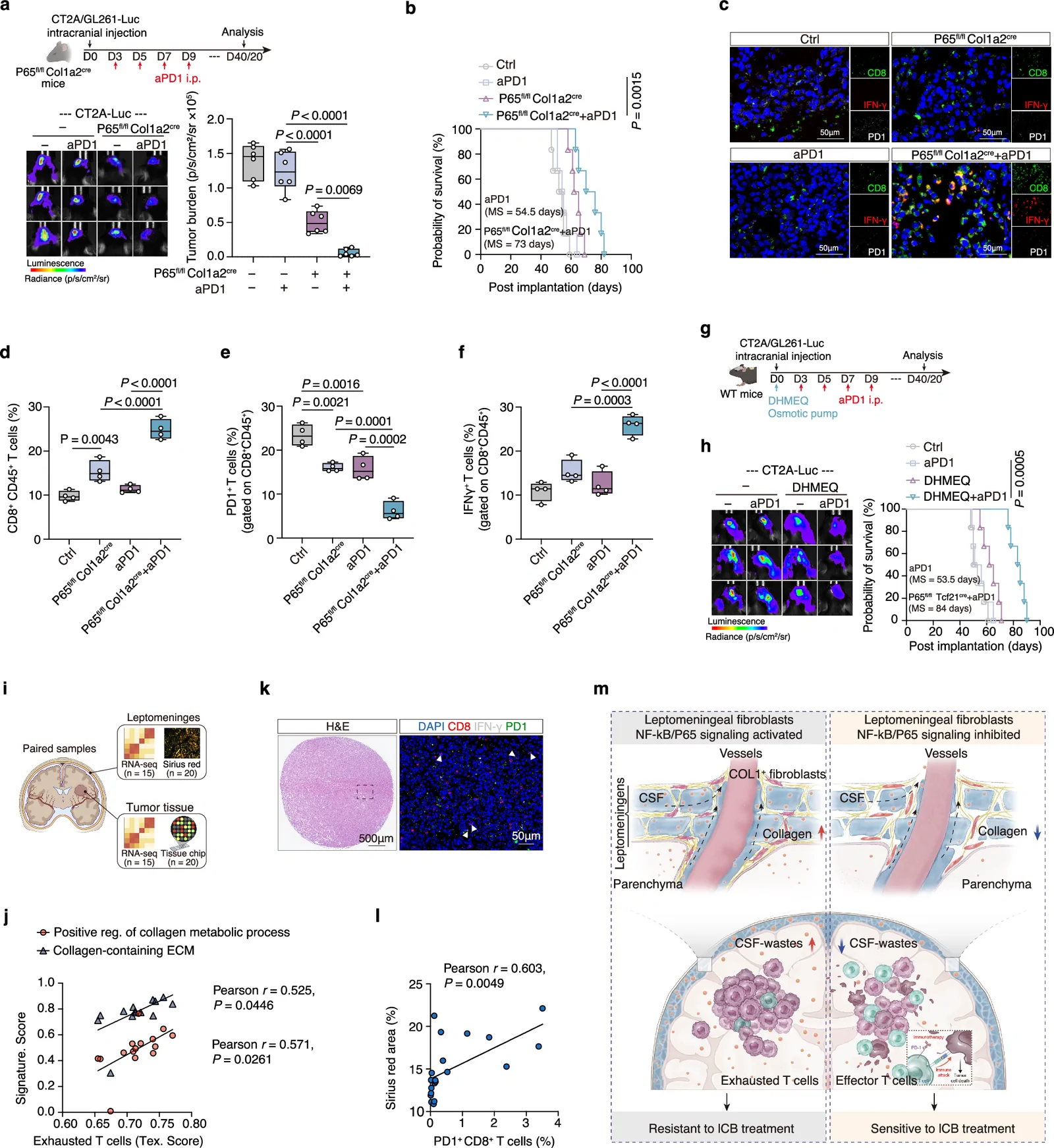
Genetic and pharmacological targeting of NF-κB/P65 signaling in GBM models and patient samples. **a**,**b** Representative bioluminescence images, tumor burden (*P* values, one-way ANOVA with Tukey’s test) and Kaplan-Meier survival (Log-rank tests) of CT2A-Luc tumor-bearing mice in the control (Ctrl), anti-PD-1 monotherapy (aPD1), P65^f/f^ + pAAV9-Col1a2-Cre-mCherry, and aPD1 + P65^f/f^ + pAAV9-Col1a2-Cre-mCherry groups (*n* = 6 mice per group). ip: intraperitoneal injection. **c** Representative fluorescence images of CD8⁺IFN-γ⁺PD1⁺ cells in the tumor tissues of the Ctrl, aPD1, P65^f/f^ + pAAV9-Col1a2-Cre-mCherry, and aPD1 + P65^f/f^ + pAAV9-Col1a2-Cre-mCherry groups (GL261-Luc model). Scale bar, 50 μm. **d–f** Quantification of CD45⁺CD8⁺ cells, PD1⁺ cells among CD45⁺CD8⁺ T cells, and IFN-γ⁺ cells among CD45⁺CD8⁺ T cells in the tumor tissues of CT2A-Luc tumor-bearing mice in the Ctrl, P65^f/f^ + pAAV9-Col1a2-Cre-mCherry, aPD1, and aPD1 + P65^f/f^ + pAAV9-Col1a2-Cre-mCherry groups (*n* = 4 mice per group); *P* values, one-way ANOVA with Tukey’s test. **g** Schematic of DHMEQ, anti-PD-1, or combination treatment in CT2A-Luc and GL261-Luc tumor-bearing mice. **h** Representative bioluminescence images and survival analysis of CT2A-Luc tumor-bearing mice treated with Ctrl, DHMEQ, aPD1, or aPD1 + DHMEQ (*n* = 6 mice per group); Kaplan-Meier curves, and group differences were assessed with Log-rank (Mantel-Cox) tests. **i** Schematic of bulk RNA-sequencing, histological and immunofluorescence analyses of leptomeningeal and tumor tissues from newly diagnosed GBM patients. Figure created with BioRender.com. **j** Correlation between exhausted T-cell signature scores and collagen-associated programs in GBM samples (*n* = 15 patients); *P* values, two‑tailed Pearson’s correlation coefficient. **k** Representative H&E and immunofluorescence images of tumor tissues from newly diagnosed GBM patients (representative of independent patient samples; quantification in Fig. 6l). Scale bars, 500 μm; zoom-ins, 50 μm. **l** Correlation between the proportion of PD1⁺CD8⁺ T cells in tumors and the Sirius Red-positive area in the leptomeninges (*n* = 20 patients); *P* values, two‑tailed Pearson’s correlation coefficient. **m** Schematic summary of the study. Box plots display the median (center line), 25 and 75th percentiles (box limits), minimum and maximum values (whiskers), with all individual data points shown for Fig. 6a, d–f. Source data are provided as a Source Data file.

Immune checkpoint blockade (ICB) has shown limited efficacy in GBM, at least in part because of the TIME^53,54^. To investigate whether targeting the pathological alterations of leptomeningeal fibroblasts to reverse the TIME can enhance the responsiveness to ICB, we evaluated anti-PD-1 treatment in combination with targeted P65 deletion in Col1a2^+^ cells in P65^f/f^ mice receiving pAAV9-Col1a2-Cre-mCherry in the CT2A-luc model. Compared with anti-PD-1 monotherapy and the corresponding control groups, this combination showed greater antitumour efficacy, as reflected by reduced tumor burden and prolonged survival (Fig. 6a, b). Similar results were observed in the GL261-luc model (Supplementary Fig. 6a, b). Analysis of the TIME further showed that, in both murine GBM models, P65^f/f^ mice receiving pAAV9-Col1a2-Cre-mCherry together with anti-PD-1 treatment exhibited increased intratumoral CD8^+^ T-cell abundance (Fig. 6c, d and Supplementary Fig. 6c–e), reduced PD-1 expression on intratumoral CD8^+^ T cells (Fig. 6e and Supplementary Fig. 6f), and increased frequencies of IFN-γ^+^ CD8^+^ T cells compared with the other groups (Fig. 6f and Supplementary Fig. 6g).

To begin to explore the translational potential of NF-κB/P65 inhibition in this pathological context, we next used DHMEQ, a pharmacological inhibitor of NF-κB/P65 (Fig. 6g). DHMEQ treatment improved the CSF clearance-associated readout in both CT2A- and GL261-bearing mice (Supplementary Fig. 6h, i). Although anti-PD-1 monotherapy showed limited efficacy in both models, the combination of anti-PD-1 and DHMEQ significantly reduced tumor burden (Fig. 6h and Supplementary Fig. 6j, k) and prolonged survival (Fig. 6i and Supplementary Fig. 6l). Together, these data suggest that genetic or pharmacological inhibition of NF-κB/P65 is associated with improved responsiveness to anti-PD-1 therapy in GBM models.

To assess the clinical association between leptomeningeal fibrosis and the tumor immune microenvironment in GBM, we collected paired leptomeningeal and tumor tissues from treatment-naive patients with GBM and performed bulk RNA sequencing together with histological analyses (Fig. 6i). Tex-signature scores were positively correlated with collagen-associated gene sets, including positive regulation of collagen metabolic process (Pearson *r* = 0.571, *P* = 0.0261) and collagen-containing extracellular matrix (Pearson *r* = 0.525, *P* = 0.0446) (Fig. 6j). In addition, the proportion of CD8⁺PD1⁺ T cells in tumors was positively correlated with Sirius Red-positive area in the leptomeninges (Pearson *r* = 0.603, *P* = 0.0049) (Fig. 6k, l). Collectively, the murine and patient data support an association between leptomeningeal fibrosis and CD8⁺ T-cell dysfunction in GBM, with the murine models further linking this state to impaired CSF clearance-associated phenotypes and reduced responsiveness to anti-PD-1 therapy (Fig. 6m).

## Discussion

Our study identifies a pathological mechanism by which GBM perturbs CSF clearance through leptomeningeal fibrosis and associates this disturbance with a more immunosuppressive tumor microenvironment. These findings support a pathological axis linking the leptomeninges, CSF dynamics and T cell accumulation, and provide a broader framework for understanding GBM progression and potential therapeutic intervention.

As both a mechanical protective barrier and a metabolic waste clearance system of the CNS^55^, CSF homeostasis is essential for maintaining neurological function^56^. Previous studies have demonstrated that reduced CSF secretion or impaired clearance is closely associated with brain dysfunction in aging and neurodegenerative diseases such as Alzheimer’s disease^50,57^. The glymphatic system, including the arterial paravascular pathway in the subarachnoid space and the venous paravascular/meningeal lymphatic pathway, serves as a major route for CSF clearance^32,34^. Under physiological conditions, CSF enters the brain parenchyma and exchanges with interstitial fluid via the arterial paravascular pathway, subsequently draining into the deep cervical lymph nodes and ultimately entering the systemic circulation through venous paravascular and meningeal lymphatic pathways^32,34^. However, CSF clearance is mediated by multiple partially parallel routes, including perivascular/glymphatic transport, meningeal lymphatic drainage and arachnoid/villous absorption. Accordingly, impaired CSF clearance in GBM is likely multifactorial. While our data support leptomeningeal fibrosis as an important gatekeeping mechanism that can restrict CSF outflow, they do not exclude additional contributors such as altered vascular pulsatility, meningeal lymphatic dysfunction, or broader CSF hydrodynamic disturbances. We also note that the apparent dissociation between increased ventricular CSF velocity and reduced tracer-based clearance should be interpreted cautiously. PC-MRI-derived velocity reflects local, plane-specific pulsatile motion and does not directly quantify net CSF turnover, effective solute transport, downstream outflow resistance, craniospinal compliance, or intracranial pressure. Therefore, although inefficient oscillatory flow, altered compliance, increased outflow resistance, or ICP-related changes could potentially contribute to this dissociation, our data do not directly test these mechanisms, and these possibilities remain speculative. Accordingly, in the current study, increased ventricular CSF velocity and reduced clearance should be regarded as an observed association rather than evidence for a defined hydrodynamic mechanism. Future studies incorporating direct ICP measurements and more comprehensive PC-MRI metrics, including net flow, stroke volume, forward and reverse flow volumes, pulsatility index, or regurgitant fraction, will be needed to resolve the underlying physiology.

In this study, we observed significantly reduced CSF tracer signals in the cervical lymph nodes of GBM-bearing mice, consistent with prior reports^24^. Although GBM patients exhibited compensatory increases in CSF flow velocity before and after surgery, the leptomeninges-which serve as critical gatekeepers for CSF entry into meningeal lymphatic vessels, with their perivenous spaces functioning as pivotal components of the paravascular pathway^34,35^ underwent profound pathological remodeling during GBM progression. Histological analyses uncovered extensive deposition of type I/III collagen within the leptomeningeal PVS distant from the tumor, forming a physical barrier that disrupts physiological CSF drainage. Both in vitro and in vivo experiments showed that gCSF activates the NF-κB signaling pathway, driving aberrant activation of leptomeningeal fibroblasts and excessive collagen secretion. Proteomic analyses further identified gCSF as enriched with profibrotic secretory proteins such as PDGFC, PDGFD and LGALS9, which may contribute to the formation of pathological fibroblasts^47^. Nevertheless, although PDGFC, PDGFD and LGALS9 are enriched in gCSF and PDGFRβ is expressed on perivascular fibroblasts, these data remain correlative. Establishing a causal PDGF-PDGFRβ- or LGALS9-dependent mechanism will require targeted perturbation studies. More broadly, the CSF-induced fibroblast phenotype is likely driven by the synergy of multiple CSF-borne factors rather than by a single dominant mediator. Additionally, CSF contains brain metabolic waste products including sphingomyelin, lactate, and tryptophan metabolites^58,59^. Among these, sphingomyelin has been reported to reprogram tumor-associated macrophages toward an antitumor phenotype^18^ suggesting that the complex composition of CSF may influence leptomeningeal function through multiple pathways. While our current study highlights the central role of secretory proteins, the contribution of other CSF metabolites to fibroblast activation and leptomeningeal remodeling warrants further investigation.

Previous studies have predominantly focused on intratumoral immunosuppression (e.g., TAMs/MDSCs) or localized CSF abnormalities (e.g., cytokine concentration changes)^60–64^. In contrast, our study, through conditional gene knockout and CSF infusion experiments, supports the concept that CSF clearance impairment can indirectly promote the accumulation of exhausted T cells in tumor regions in association with leptomeningeal structural disruption. Further analysis of paired leptomeningeal and tumor samples from GBM patients demonstrated a positive correlation between leptomeningeal fibrosis severity and the number of exhausted T cells in tumor regions, suggesting that CSF clearance impairment may contribute to immunosuppression by influencing immune cell infiltration and/or functional state. Due to clinical limitations, we used leptomeningeal fibrosis severity as a surrogate marker for CSF clearance impairment; future larger cohort studies incorporating direct assessment of CSF clearance will be required to further define the relationship between CSF dysfunction, immune contexture and clinical outcome.

Our scRNA-seq analysis of P65^f/f^Col1a2^Cre^ tumors also revealed increased expression of antigen-presentation and immune-activation programs across immune compartments, including macrophage/microglia populations, consistent with a more inflammatory and antigen-presenting myeloid state^65^. Thus, in addition to effects on exhausted T cells, altered CSF clearance and meningeal drainage may influence antigen availability or APC trafficking, thereby shaping antitumor immunity and response to anti-PD-1 therapy. These interpretations remain correlative and will require dedicated functional studies. Recent leptomeningeal single-cell and single-nucleus atlases further support the view that the leptomeninges are a heterogeneous CNS-border niche containing specialized fibroblast/barrier programs, tissue-resident-like lymphocytes and BAM populations distinct from parenchymal microglia, indicating that immune regulation may occur locally within CSF-exposed meningeal microenvironments.

Our study confirms that targeting the core NF-κB transcription factor P65 in leptomeningeal fibroblasts, either by pharmacological inhibition or conditional knockout, significantly improves CSF clearance and enhances the efficacy of ICB. This provides insights for GBM treatment: maintaining CSF homeostasis, for example by preventing leptomeningeal fibrosis, may help reverse the immunosuppressive microenvironment and improve ICB response. At the same time, because NF-κB/P65 is ubiquitously expressed, the fibroblast-specific P65 genetic experiments presented here are intended to establish mechanistic necessity rather than an immediately translatable therapy. Future studies will need to develop cell-targeted delivery approaches or identify fibroblast-selective downstream effectors. In addition, although this study focuses on leptomeningeal fibroblasts, other meningeal-resident cell types, including immune, vascular-associated and meningeal epithelial-like cells, may also sense CSF-derived signals and contribute to CSF-related pathology. The NF-κB pathway contains multiple regulatory elements, such as IKKβ^66^, and leptomeningeal fibroblasts express markers such as PDGFRβ, raising the possibility of more selective intervention strategies. Additionally, VP shunt placement for CSF diversion has been associated with overall survival in subsets of GBM patients; however, whether modulating CSF dynamics benefits unselected newly diagnosed patients remains unknown and requires prospective study.

Several limitations should also be acknowledged. First, non-tumor leptomeningeal control tissues were obtained from epilepsy surgeries. Because epilepsy can be associated with chronic neuroinflammation, some baseline immune or extracellular matrix alterations in these leptomeningeal samples cannot be completely excluded, although neoplastic disease was excluded radiographically and tumor infiltration was excluded pathologically. Future studies incorporating additional non-epilepsy controls and quantitative assessment of inflammatory infiltrates will be valuable. Second, our mechanistic evidence is derived primarily from the GL261 and CT-2A models together with correlative human datasets; therefore, the extent to which this CSF-leptomeningeal fibrosis axis generalizes across molecular GBM subtypes remains to be established in larger, subtype-stratified cohorts and additional models. Importantly, these syngeneic mouse models do not fully recapitulate the genetic, cellular and immunological heterogeneity of human GBM, and validation in less immunogenic models in the current study remains limited. Accordingly, the human analyses presented here should be interpreted cautiously, as they are associative, based on relatively limited clinical and pathological readouts, and not sufficient to establish causality. Although we controlled for age and sex, our clinical analyses remain susceptible to residual confounding by molecular subtype and treatment-related factors. Third, all mechanistic mouse experiments in the current study were performed in male mice. Therefore, potential sex-dependent differences in CSF dynamics, leptomeningeal remodeling, fibroblast activation and antitumor immunity were not addressed here. Future studies incorporating both male and female animals will be required to determine the generalizability of these findings across sex. Fourth, the mechanism described here is supported in a deep-seated orthotopic model, but tumor location may modulate the extent and pattern of leptomeningeal/PVS remodeling and CSF clearance impairment. Future studies should systematically compare superficial versus deep tumor models, stratified by tumor-to-cortical-surface distance and tumor burden.

In summary, our study provides structural and mechanistic evidence that GBM is associated with impaired CSF clearance and identifies leptomeningeal fibrosis as an important contributor to this process. By incorporating CSF kinetic abnormalities as a regulatory component of GBM progression, this work extends beyond models focused solely on the local tumor microenvironment. These findings broaden our understanding of GBM pathogenesis and point to the leptomeningeal-CSF dysfunction-T cell accumulation axis as a potentially actionable therapeutic framework for improving clinical outcomes (Fig. 6m). Future studies should further clarify the roles of additional CSF metabolites and secreted factors in fibroblast regulation, refine precision targeting of fibroblast-associated signaling pathways, and determine the clinical utility of CSF clearance assessment and intervention in GBM management.

## Methods

### Ethics statement

All animal procedures were conducted in accordance with the Fudan University Guidelines for the Care and Use of Laboratory Animals and were approved by the Institutional Animal Care and Use Committee of Fudan University under protocol FLYP-FE23207R. For orthotopic GBM models, the maximal permitted tumor burden was defined by the humane endpoints approved in the animal protocol, including severe neurological symptoms, body-weight loss exceeding 20% of the pre-implantation baseline, moribund appearance, or other signs of distress requiring euthanasia. Because intracranial tumors cannot be reliably monitored by caliper-based volume measurement, tumor burden was monitored longitudinally by bioluminescence imaging and by daily clinical assessment after tumor implantation. Animals were euthanized immediately when predefined humane endpoints were reached. The maximal tumor burden permitted by the approved animal protocol was not exceeded in this study. The use of human specimens was approved by the Ethics Review Committee of Huashan Hospital, Fudan University, and the study was conducted in accordance with the Declaration of Helsinki. Surgical specimens and cerebrospinal fluid samples were obtained from treatment-naïve GBM patients and non-tumor control individuals after written informed consent was obtained from all participants. No financial compensation was provided for sample donation. Human samples included tumor tissues, tumor-distal leptomeningeal tissues, non-tumor control leptomeningeal tissues, and intraoperatively collected cerebrospinal fluid.

### Clinical samples

The clinical samples used in this study included surgical specimens from treatment‑naïve GBM patients and non‑tumor control individuals. Participants comprised adult males and females, with ages ranging from 18 to 76 years; sex and age distributions for the cerebrospinal fluid dynamics cohort are summarized and disaggregated by experiment in the Source Data. Sex was assigned at birth based on medical records; gender identity was not collected. Sex was considered in the study design and analyzed specifically for cerebrospinal fluid flow dynamics; no sex‑based analysis was performed for other endpoints. Samples were categorized into three types: (1) Leptomeningeal tissues: obtained from tumor‑distal regions of GBM patients (confirmed intraoperatively by pathology to be free of tumor cell infiltration) and non‑tumor control leptomeningeal tissues from individuals undergoing neurosurgical procedures for epilepsy; (2) Tumor tissues: primary tumor masses resected from GBM patients during surgery; (3) CSF: including GBM‑derived CSF (gCSF) and non‑tumor control CSF (cCSF), collected from cisternal fluids intraoperatively (free of hemorrhagic contamination). All CSF samples were processed within 30 min of collection: cellular debris was removed by centrifugation at 400 g for 7 min at 4 °C, and supernatants were aliquoted and stored at −80 °C; some samples were used immediately after centrifugation. Preoperative brain MRI (T1‑weighted pre‑ and post‑gadolinium, T2‑FLAIR) was reviewed for all GBM cases to document tumor location and exclude radiological evidence of leptomeningeal dissemination (defined as leptomeningeal contrast enhancement or sulcal FLAIR hyperintensity).

### Animals

#### Experimental animals and housing conditions

All in vivo experiments were performed using male mice (6-8 weeks old), including wild-type C57BL/6 J mice and genetically modified (offspring) mice. Mouse strains used in this study included C57BL/6 J mice and the following strains purchased from GemPharmatech: P65^f/f^, Col1a2-P2A-CreERT2-PolyA, and Cspg4-CreERT2. Rosa-LSL-tdTomato mice were purchased from Shanghai Model Organisms Center. All mice were housed under specific pathogen-free (SPF) conditions with controlled temperature (22 °C ± 1 °C), humidity (50 ± 10%), and a 12-hour light/dark cycle, with ad libitum access to food and water.

#### Orthotopic GBM tumor model

Male C57BL/6 J mice (6–8 weeks old) were anesthetized with isoflurane (RWD Life Science) and positioned in a stereotaxic apparatus (RWD Life Science). Luciferase-expressing glioma cells (GL261-luc or CT2A-luc; 2 × 10⁵ or 5 × 10⁴ cells in 5 μL) were implanted intracranially at the following coordinates relative to bregma: AP, 1.0 mm; ML, 2.0 mm; DV, 3.0 mm. Tumor progression was followed by bioluminescence imaging (BLI) using an AniView100 Multimode Live Animal Imaging System (Boluteng Biological Technology Co., Ltd., Guangzhou, China). D‑luciferin sodium (Macklin, D812647) was administered intraperitoneally (150 mg/kg). Images were acquired 14 min after luciferin injection with a 90‑s exposure time; the imaging time point was chosen based on pilot testing to capture a stable bioluminescent signal. BLI data were processed with AniView software. Total photon flux (photons/s) was quantified within a manually drawn ROI covering the cranial tumor signal, with background subtraction performed using a standardized ROI placed in a non-tumor area. All animals were imaged using the same acquisition and analysis settings. Photon flux was used as a relative longitudinal indicator of tumor burden for within-study comparisons, rather than as an absolute volumetric measurement. Animals were euthanized when they reached predefined humane endpoints, including severe neurological symptoms, marked weight loss, or moribund appearance.

#### Orthotopic GBM tumor resection model

At 12 days post-inoculation of the orthotopic GBM tumor model, visible tumor masses around the original injection site were carefully dissected under a ×20 microscope, with maximal removal of all tumor tissue. Mice were allowed to recover for 7 days before subsequent experiments.

#### Lineage tracing

C57BL/6J-background Col1a2^CreERT2^ and Cspg4^CreERT2^ mice were generated by CRISPR-Cas9. Rosa‑LSL‑tdTomato mice were created by knocking the CAG promoter-loxP‑Stop‑loxP-tdTomato cassette into the Rosa26 safe-harbor locus. Rosa‑LSL‑tdTomato homozygotes were crossed with Col1a2^CreERT2^ heterozygotes or Cspg4^CreERT2^ heterozygotes, and double-heterozygous mice (Rosa‑LSL‑tdTomato/Col1a2^CreERT2^ and Rosa‑LSL‑tdTomato/Cspg4^CreERT2^) were confirmed by PCR genotyping. At 6 weeks of age, mice received tamoxifen (100 mg/kg; dissolved in sterile corn oil) by intraperitoneal injection for five consecutive days to induce tdTomato expression. An orthotopic GBM model was established at 8 weeks, and brains were collected at 10 weeks for Collagen I staining. ImageJ was used to calculate total collagen area and to quantify tdTomato⁺ reporter cells within the collagen-positive regions.

#### Conditional deletion of P65 in Col1a2-targeted cells

P65^f/f^ mice were anesthetized, shaved and disinfected on the neck, and fixed in a stereotactic frame. A midline incision was made to expose the muscle layer, which was bluntly dissected to reveal the cisterna magna. A microsyringe was used to inject adeno-associated virus (AAV9-Col1a2-Cre-(mCherry) or control virus AAV9-Col1a2-(mCherry), purchased from Cyagen, titer: 1 × 10¹¹ vg/mL) into the cisterna magna (dose: 1 × 10¹¹ vg per mouse, completed within 2 min). Viral infection efficacy was validated at 3 weeks post-injection (fluorescence staining procedures are detailed in the Histology and Morphology Analysis section).

#### CSF infusion experiment

An osmotic pump (RWD Life Science, 1001 W) was used to model CSF clearance impairment induced by gCSF. CSF was collected from the cisterna magna of healthy mice (control cCSF) and GBM-bearing mice, centrifuged to remove cellular debris, and stored until use. gCSF (or cCSF) was continuously infused into the lateral ventricle of recipient mice (stereotaxic coordinates: AP + 0.5 mm; ML + 1.0 mm; DV −2.5 mm) at a constant rate of 0.5 μL/hr using the osmotic pump implanted subcutaneously on the back. The pump reservoir (nominal volume 100 μL) provides ~8.3 days of infusion at this rate. Leptomeningeal fibrosis was assessed at indicated time points after completion of infusion (fluorescence staining procedures are described in Histology and Morphology Analysis). On day 5 after completion of infusion, an orthotopic CT2A-Luc/GL261-Luc tumor model was established for subsequent experiments.

#### Antibody/drug interventions

For CT2A-Luc tumor-bearing mice, an osmotic pump (model 1001 W, RWD Life Science) was implanted on day 7 post-tumor establishment to infuse pirfenidone (Yeasen, 53179-13-8) into the CSF at 12 mg/mL via a fixed delivery rate of 0.5 μL/hr for 3 days. Leptomeningeal fibrosis (fluorescence staining procedures are detailed in the Histology and Morphology Analysis section) and CSF clearance rate (see the Gadobutrol CSF Tracer section) were assessed post-intervention. In the CD8 antibody neutralization experiment, anti-CD8α monoclonal antibody (BioXCell, BE0061, 100 μg per mouse) was administered intraperitoneally every 3 days starting from day 0 of CT2A-Luc/GL261-Luc cell inoculation to deplete CD8⁺ T cells; the control group received an equal volume of IgG isotype antibody (100 μg per mouse). For immune checkpoint inhibitor experiments, anti-PD-1 monoclonal antibody (BioXCell, BE0146, 200 μg per mouse) was injected intraperitoneally on day 3 post-inoculation, followed by administration every 2 days until the end of the experiment. DHMEQ (Medchemexpress, HY-14645) was dissolved in 10% DMSO and 90% saline and continuously delivered via the CSF at a dose of 10 mg/kg/day using an osmotic pump (RWD Life Science, 1001 W).

#### Intravascular CD45 labeling and Ki67 analysis

To distinguish intravascular from extravascular (tissue) leukocytes, tumor-bearing mice were injected intravenously with anti-mouse CD45-PE (BioLegend, 147711; 3 μg in 100 μl PBS per mouse) 3 min before sacrifice. Tumors were rapidly harvested and dissociated into single-cell suspensions. Cells were stained for surface markers. Leukocytes were defined as CD45-FITC^+^ events and stratified by i.v. CD45-PE labeling into intravascular (CD45-FITC^+^CD45-PE^+^) and extravascular/tissue (CD45-FITC^+^CD45-PE^−^) compartments, within which CD8^+^ T cells were quantified by flow cytometry. For local proliferation analysis, cells were fixed and permeabilized and stained intracellularly with anti-Ki67; the Ki67^+^ fraction among intratumoral CD8^+^ T cells was quantified by flow cytometry.

#### Cell lines and primary cell culture

The mouse GBM cell lines GL261-Luc (purchased from Mingzhoubio, MZ8070) and CT2A (purchased from NOBLEBIO, nobcell0435) were cultured in high-glucose Dulbecco’s modified Eagle’s medium (DMEM, Gibco) supplemented with 10% fetal bovine serum (FBS, Gibco) and 1% penicillin-streptomycin. The CT2A-Luc cell line was generated by transducing the CT2A cell line with a lentiviral vector carrying a luciferase reporter gene and a puromycin resistance gene. Human embryonic fibroblasts (HEbF, purchased from otwobiotech, HTX3968) were cultured in fibroblast-specific medium (otwobiotech). hPLMFs (Primary Human Leptomeningeal Fibroblasts) : Surgically resected leptomeningeal tissues were immersed in 4 °C PBS and dissociated in tissue digestion buffer (bioGenous, K601008) at 37 °C for 15 min. The dissociated mixture was filtered through a 70 μm cell strainer, and the filtrate was centrifuged at 200 *g* for 3 minutes at 4 °C to collect the cells. LAMA2-positive fibroblasts (hPLMFs) were isolated via flow cytometry: cells were incubated with a LAMA2 antibody (Sigma, L0663) for 2 h at 4 °C, followed by incubation with an AF647-conjugated goat anti-mouse IgG (H + L) secondary antibody (Beyotime, A0473) for 1 h. The sorted LAMA2-positive fibroblasts were cultured in DMEM (Gibco) supplemented with 10% FBS (Gibco) under adherent conditions, with gentle pipetting during handling to maintain cell integrity. All cell lines were routinely tested for mycoplasma contamination and were negative. Cell identity was confirmed by supplier authentication and maintained according to the manufacturer’s recommendations.

### Histology and morphological analysis

#### Tissue fixation and sectioning

Mouse and human tissue samples were fixed in 4% paraformaldehyde (PFA) overnight at 4 °C, followed by gradient dehydration and paraffin embedding (for hematoxylin-eosin [HE] staining, immunohistochemistry [IHC], Sirius Red staining, and immunofluorescence [IF]) or optimal cutting temperature (OCT) compound embedding (for frozen sections and IF). Fresh tissues were directly used for single-cell suspension preparation, flow cytometry sorting, or molecular experiments.

#### Scanning electron microscopy (SEM)

Leptomeningeal tissues were fixed in 2.5% glutaraldehyde at 4 °C, dehydrated through a graded ethanol series, and subjected to critical point drying (CO₂ critical point dryer). The dried samples were sputter-coated with gold (ion sputter coater, 10 nm thickness) and examined using a scanning electron microscope (Zeiss Gemini 300) to observe surface ultrastructure at an accelerating voltage of 10 kV.

#### HE and Sirius red staining

Paraffin-embedded tissue samples were fixed in 4% PFA, followed by gradient ethanol dehydration (70%-100%), xylene clearing, and paraffin embedding. Continuous sections (4-5 μm thick) were cut and baked at 60 °C for 2 h. Sections were routinely deparaffinized to water (xylene ×2, gradient ethanol to water) and stained as follows: HE staining: Nuclei were stained with hematoxylin for 5–10 min, followed by tap water blueing; cytoplasm was stained with eosin for 1–3 min. Sections were then dehydrated through gradient ethanol, cleared with xylene, and mounted with neutral resin. Sirius Red staining: Sections were stained with 0.1% Sirius Red dissolved in saturated picric acid solution for 1 h in the dark, rinsed briefly with running water, dehydrated through gradient ethanol, cleared with xylene, and mounted with neutral resin. HE staining was used to observe tissue architecture, while Sirius Red-stained sections were examined under polarized light microscopy to visualize type I/III collagen fibers.

#### Immunohistochemistry (IHC) and Immunofluorescence (IF)

Paraffin sections were deparaffinized (xylene ×3, gradient ethanol rehydration), subjected to antigen retrieval (Tris-EDTA buffer, pH 9.0, microwave repair for 15 minutes), and blocked with 10% goat serum at room temperature for 30 minutes. Multiplex fluorescence staining was performed using tyramide signal amplification (TSA) technology. Primary antibodies were incubated overnight at 4 °C (commonly used antibodies included: anti-α-SMA [Servicebio, GB12045], anti-COL4A1 [Proteintech, 30850-1-AP], anti-COL1A1 [Servicebio, GB154197], anti-COL1A2 [Abcam, AB322963], anti-COL3A1 [Servicebio, GB111629], anti-VWF [Abcam, AB287962], anti-FAP [Proteintech, 11779-1-AP], anti-PDGFRB [Proteintech, 13449-1-AP], anti-CD31 [Abcam, AB182981], anti-PDGFRA [Abcam, AB203491], anti-COL11A1 [Abcam, AB270946], anti-MYH11 [Abcam, AB224804], anti-CD45 [CST, 70257], anti-CD8 [Servicebio, GB15068], anti-CD8 [ZSGB-BIO, ZA-0508], anti-PD1 [PTG, 18106-1-AP], anti-IFN-γ [Affinity, DF6045], anti-p-P65 [Beyotime, AF5875], anti-Ki67 [Abcam, AB16667], and anti-P65 [Servicebio, GB12997]. After each primary antibody incubation, the corresponding Alexa Fluor-series fluorescent secondary antibodies (HRP-labeled, matched to the primary antibody host) were incubated at room temperature for 30 minutes. Subsequently, different fluorophore-labeled tyramides (Alexa Fluor 488/555/647) were added for TSA signal amplification (incubated in the dark for 5–10 min with 0.001–-0.003% H₂O₂). After completing TSA staining for each target, residual HRP activity was quenched with 0.01 M HCl or glycine solution (2 mg/mL), followed by staining for the next target. After all target staining was completed, nuclei were counterstained with DAPI (Beyotime, C1006) for 5 min. Fluorescence signals were acquired using a confocal microscope (Zeiss LSM 880), and quantitative analysis was performed with ImageJ software (measuring positive area proportion/positive cell count).

#### scRNA-seq for leptomeningeal tissue

Leptomeningeal tissue was obtained from three non‑tumor control human participants and 3 GBM patients, under institutional ethics approval and written informed consent. Fresh tissue was washed in ice‑cold PBS and enzymatically dissociated using a commercial tissue digestion buffer (BioGenous, K601008) at 37 °C for 15 min with gentle agitation. The cell suspension (supernatant and remaining tissue fraction) was pooled and filtered through a 70‑µm strainer (BD Falcon, 352350), centrifuged at 500 g for 5 min, and subjected to an additional digestion step in 1 mL trypsin for 15 min. All fractions were pooled, filtered through a 35‑µm strainer (BD Falcon, 352235), centrifuged at 500 *g* for 5 min, and resuspended in PBS + 0.01% BSA. Cells were processed on the 10x Genomics Chromium Next GEM platform, and 3′ scRNA‑seq libraries were generated using the Chromium Next GEM Single Cell 3′ Kit v3.1 according to the manufacturer’s instructions. Libraries were sequenced on an Illumina NovaSeq 6000 using paired‑end 150‑bp reads.

Base call files were processed using Cell Ranger (v9.0.1) and aligned to the human reference genome (GRCh38; Cell Ranger reference), generating a filtered feature‑barcode matrix for each sample. Downstream analysis was performed in R (v4.3.0) using Seurat (v4.4.4) unless noted. Putative doublets were identified and removed for each sample using DoubletFinder_v3. The expected doublet rate was estimated from the total number of recovered cells, assuming a linear increase of 0.5% per 1000 cells; across samples, the expected rate ranged from 3% to 7%. To determine the optimal neighborhood size parameter (pK), parameter sweep analysis was performed using paramSweep_v3 and summarizeSweep with pN set to 0.25 and the first 20 principal components (PCs) as input. The optimal pK value for each donor was selected using find.pK based on the peak of the mean-variance normalized BCmetric profile. DoubletFinder_v3 was then run using the donor-specific optimal pK and the calculated expected doublet number, and predicted doublets were removed prior to downstream analysis. Cells were excluded if they met any of the following criteria: (i) detected genes (nFeature_RNA) outside median ± 3 SD, calculated within each sample, or (ii) high mitochondrial transcript fraction (*percent.mt* > 30%). Data were log-normalized using NormalizeData (default settings) and scaled using ScaleData. Highly variable genes were selected using FindVariableFeatures (top 3000 HVGs). Patient-level batch effects were corrected using Harmony, and the Harmony-corrected embedding was used for downstream dimensionality reduction, clustering, and visualization.

Principal component analysis (PCA) was performed on highly variable genes. For unsupervised clustering, a shared nearest-neighbor (SNN) graph was constructed in the Harmony-integrated space using FindNeighbors with the first 30 dimensions (dims = 1:30). Clusters were identified using the Louvain algorithm implemented in FindClusters with a resolution of 0.6 and a random seed of 16. UMAP was computed for visualization based on the same Harmony-corrected dimensions. We emphasize that UMAP was used for visualization only; quantitative comparisons are based on differential expression and gene‑set/module scoring. Major cell classes were annotated using canonical marker genes and supported by dot plots. Markers included: endothelial cells (PECAM1/CD31, VWF, CLDN5), fibroblasts (COL1A1, COL1A2, PDGFRA), mural cells (ACTA2, TAGLN, MYH11, CNN1; COL4A1), and immune cells (PTPRC). Clusters lacking clear lineage markers were annotated as other. To increase resolution within fibroblasts, fibroblast‑annotated cells were subset and reprocessed using the same workflow. Fibroblast subclusters were identified at resolution 0.1 and annotated using a curated marker panel including ECM genes (e.g., COL1A1/COL1A2, LUM, DCN), activation markers (e.g., FAP, CTHRC1), and smooth muscle/myofibroblast‑associated markers (e.g., ACTA2), while excluding pericyte‑dominant signatures (e.g., CSPG4/NG2). Differentially expressed genes were identified using Seurat FindAllMarkers with thresholds adjusted *P* < 0.05 and *avg_log*_*2*_*FC* > 0.25. GO/KEGG enrichment and GSEA were performed using clusterProfiler (v4.10.1), using all detected genes as background. Enrichment significance was defined as nominal *P* < 0.05 and *FDR (q)* < 0.05. Gene sets were obtained from MSigDB (https://www.gsea-msigdb.org). Fibrosis/ECM module scores (e.g., COLLAGEN_FIBRIL_ORGANIZATION, COLLAGEN_CONTAINING_EXTRACELLULAR_MATRIX) were calculated using Seurat AddModuleScore and compared across fibroblast subsets and conditions. These quantitative scores were used to support statements regarding ECM‑remodeling/fibrotic activation states. Fibroblast differentiation trajectories were reconstructed using Monocle2 (v2.30.0). To balance cluster representation, up to 1000 cells per fibroblast subcluster were randomly retained. Genes used for ordering were selected by differentialGeneTest (*q‑value* < 0.01), excluding mitochondrial, ribosomal, and hemoglobin genes. Dimensionality reduction was performed with DDRTree, and trajectories were visualized using plot_cell_trajectory. We note that pseudotime analyses infer transcriptional state relationships and do not establish causality.

#### scRNA-seq of tumor tissue of CT2A GBM-bearing mice

Tumor tissues were collected from CT2A tumor-bearing P65^f/f^ mice receiving pAAV9-Col1a2-Cre-mCherry and from corresponding P65^f/f^ control mice receiving control vector. Tumor tissues were dissociated using tumor tissue digestion buffer (BioGenous, K601003) at 37 °C with constant shaking for 20 min. The dissociated cell suspension was filtered through a 70 μm cell strainer (BD Falcon, 352350) to obtain a single-cell suspension. CD45⁺ immune cells were enriched via CD45 MicroBeads separation (Miltenyi Biotec, 130-049-501) according to the manufacturer’s protocol. Single-cell RNA-Seq libraries were prepared using the SeekOne® DD Single Cell 3’ library preparation kit (SeekGene Catalog No. K00202). The indexed sequencing libraries were cleaned up with VAHTS DNA Clean Beads (Vazyme N411-01), analyzed by Qubit (Thermo Fisher Scientific Q33226) and Bio-Fragment Analyzer (Bioptic Qsep400). The libraries were then sequenced on the Illumina NovaSeq Plus with PE150 read length. Single-cell RNA sequencing data were processed and quality-controlled using Seurat v4.4.0. Cells were filtered based on the following criteria: gene count per cell (nFeature_RNA) between 300 and 6000, UMI count per cell (nCount_RNA) between 600 and 60,000, and mitochondrial gene percentage <7%. The integrated dataset from both groups (P65^f/f^Col1a2^Cre^ and P65^f/f^) was then clustered using the standard Seurat workflow including canonical normalization, principal component analysis (PCA), t-distributed stochastic neighbor embedding (t-SNE) dimensionality reduction, and Louvain clustering, resolving distinct immune cell clusters.

Cell type annotation was based on the expression of canonical marker genes (Plasma cells: Irf4, Prdm1, Jchain; Fibroblasts: Col1a1, Col3a1, Serpinh1, Tm4sf1; Neutrophils: S100a8, S100a9, Csf3r, Cxcr2; B cells: Cd79a, Cd79b, Ms4a1, Cd22; NK cells: Nkg7, Gzma, Klrd1, Klrb1c; Dendritic cells (DCs): Flt3, Zbtb46, Itgax, Clec9a; CD4⁺ T cells: Cd4, Il7r, Tcf7; CD8⁺ T cells: Cd3e, Cd8a, Cd8b1, Gzmb; Microglia: Tmem119, P2ry12, Hexb, Cx3cr1; Macrophages: Cd68, Ccr2, Msr1, Ly6c2). Cell proportion plots were generated based on group stratification (P65^f/f^Col1a2^Cre^ and P65^f/f^). Differential gene expression analysis between the two groups was performed for each cluster using Seurat’s FindMarkers function (Wilcoxon rank-sum test, *min.pct* = 0.1, *logfc.threshold* = 0.1, *adjusted P* < 0.05). For CD8⁺ T cells, differentially expressed genes (DEGs) between P65^f/f^Col1a2^Cre^ and P65^f/f^ groups were subjected to functional enrichment analysis using the clusterProfiler v4.10.0 package in R. Gene Ontology (GO) enrichment analysis was conducted using the enrichGO function with hypergeometric distribution test for significantly differentially expressed genes (*adjusted P* < 0.05), with parameters: *ont* = ALL, *pAdjustMethod* = BH, *pvalueCutoff* = 0.1, *qvalueCutoff* = 0.2. Significantly enriched pathways and GO terms were visualized to identify functional alterations in CD8⁺ T cell subsets.

### Bulk RNA sequencing

Total RNA was extracted from leptomeningeal tissue or tumor tissues samples of 15 GBM patients using Trizol reagent (Invitrogen) following the manufacturer’s protocol. RNA quality was assessed using an Agilent 2100 Bioanalyzer and RNase-free agarose gel electrophoresis. mRNA was enriched using Oligo(dT) magnetic beads, and Illumina sequencing libraries were constructed with the NEBNext Ultra RNA Library Prep Kit (NEB, 7530) through fragmentation, reverse transcription, end repair, A-tailing, adapter ligation, and PCR amplification. Paired-end sequencing was performed on the Illumina NovaSeq platform.

We integrated publicly available RNA-seq data from 44 non-tumor patients (Synapse platform, accession: syn34512705). All raw sequencing data underwent standardized quality control, alignment, and gene expression quantification. Batch effects across datasets were corrected using the ComBat algorithm to ensure data reliability. Based on the corrected expression matrix, differentially expressed genes (DEGs) between GBM patient leptomeningeal tissues and control samples (non-tumor leptomeningeal tissues from public data) were identified using the limma package (criteria: *|log*_*2*_*FC* | > 1.5 and *FDR* < 0.05). Functional enrichment analysis of these DEGs was performed using the clusterProfiler R package to explore key biological process alterations. Additionally, using the paired RNA-seq data of tumor tissues from the same 15 GBM patients, we calculated exhausted T-cell enrichment scores in each tumor sample via single-sample gene set enrichment analysis (ssGSEA) based on a validated set of 19 T-cell exhaustion signature genes (including ADGRG1, CD8A, PDCD1, LAG3, CTLA4, etc.). For leptomeningeal tissue RNA-seq data, two fibrosis-related GO gene sets-positive regulation of collagen metabolic process, collagen containing extracellular matrix-were analyzed using ssGSEA to generate sample-specific scores. Pearson correlation coefficients were then computed to assess potential associations between exhausted T-cell scores and fibrosis-related gene set scores in paired samples.

For RNA-seq data of hPLMFs co-cultured with cCSF or gCSF, differential expression analysis was performed using DESeq2 (for inter-group comparisons) and edgeR (for inter-sample comparisons) with criteria of *FDR* < 0.05 and *|fold change* | ≥ 2. GO functional annotations of differentially expressed genes were based on the standard GO classification system, and significantly enriched GO terms (*FDR* ≤ 0.05) were identified to investigate functional changes in fibroblasts under co-culture conditions.

### Proteomics

For each CSF sample (non‑tumor controls: *n* = 10 human participants; GBM patients: *n* = 20 human participants), 40 μL was used. Protein enrichment was performed using commercial magnetic nanoparticles. Briefly, beads were washed, resuspended in diluent, mixed with CSF, and incubated at 37 °C with shaking (1500 rpm). After magnetic separation and three washes, enriched proteins were digested in‑solution with trypsin at 37 °C (1500 rpm, 3 h). Peptides were desalted on a C18 desalting column, eluted in 25 μL elution buffer, dried, reconstituted in 8 μL mobile phase A (0.1% FA in water), and quantified by Nanodrop. Each biological sample was processed once (technical replicate); a pooled QC sample was included for quality control.

Peptide separation was performed on a Bruker nanoElute2 UHPLC coupled to a Bruker timsTOF HT mass spectrometer. For DIA analysis, iRT standard peptides (Biognosys) were spiked in at 1:20 (iRT:sample). Chromatography used a C18 column (15 cm × 100 μm, 1.9 μm) with buffer A (0.1% FA in water) and B (0.1% FA in ACN): 0–7.5 min, 6–28% B (0.7 μL/min); 7.5–9.0 min, 28–80% B (0.4 μL/min); 9.0–10.0 min, 80% B (0.7 μL/min). MS was in DIA‑TIMS mode: capillary voltage 1.6 kV; dry gas 180 °C, 3.2 L/min; m/z300–1500; ion mobility window 0.7–-1.3 V·s/cm²; collision energy 20–59 eV (ramp 50 ms). DIA windows covered m/z 300–1500 with 32 overlapping windows. Raw DIA data were processed with Spectronaut Pulsar(v18.7, Biognosys) against the UniProt human reference proteome (2025‑02 release). Search: Trypsin/P, max 2 missed cleavages; fixed Carbamidomethyl (C); variable Oxidation (M), Acetylation (Protein N‑term); precursor 10 ppm, fragment 20 ppm; min peptide length 7 aa; protein/peptide *FDR* < 1% *(q* < 0.01). Global normalization applied. PCA was performed using prcompin R. Differential expression used limma (Bioconductor, empirical Bayes; *fold change* > 1.2, *FDR* < 0.05). GSEA used clusterProfiler. QC: *CV* < 15% for pooled QC and technical replicates.

### Flow cytometry

Mouse tumor tissues were mechanically dissociated (minced with scissors) and enzymatically digested in tumor tissue digestion buffer (bioGenous, K601003) at 37 °C for 30 min. The digested mixture was filtered through a 70 μm cell strainer, washed with PBS, and treated with ACK Lysing Buffer to remove red blood cells. The final cell suspension was resuspended in PBS and adjusted to a concentration of 1 × 10⁷ cells/mL. For flow cytometry staining, the cell suspension was first incubated with 5% FBS/PBS blocking buffer at room temperature for 15 min in the dark to reduce nonspecific binding. Staining was performed in two steps: 1) Surface marker and viability staining: The cell suspension was incubated with fluorescently labeled antibodies against CD45 (BioLegend, 157213), CD45 (BioLegend, 147711), CD8a (BioLegend, 100711), CD8a (BioLegend, 100733) and PD1 (BioLegend, 135224) at 4 °C for 30 min in the dark, together with a viability dye (Thermo Fisher, L34989). After PBS washing, cells were fixed and permeabilized. 2) Intracellular marker staining: Permeabilized cells were incubated with fluorescently labeled antibodies against IFN-γ (BioLegend, 505807) at 4 °C for 30 min in the dark. After PBS washing, cells were analyzed using a flow cytometer (BD FACS Vantage SE), and data were processed with FlowJo software.

### Molecular and cellular biology experiments

#### Protein extraction and Western blot

To assess the effects of gCSF and non-tumor control CSF (cCSF) on fibroblasts, HEbF were seeded at 2 × 10⁴ cells/well in 6-well plates with 10% FBS-DMEM, incubated overnight at 37 °C/5% CO₂ for adherence. Cells were co-cultured with serum-free DMEM (FBS-free), cCSF, or gCSF for 36 h. After PBS washing, cells were lysed with RIPA buffer (containing protease/phosphatase inhibitors), centrifuged at 12,000 g for 15 mins at 4 °C, and supernatants were collected. Protein concentration was measured by BCA assay (Beyotime, P0010S). Samples were separated by SDS-PAGE, transferred to PVDF membranes, blocked with 5% skim milk (RT, 1 hur), and incubated overnight at 4 °C with primary antibodies (COL1A1, Servicebio, GB11022; β-ACTIN, Servicebio, GB15003). Membranes were washed with TBST, incubated with HRP-conjugated goat anti-rabbit IgG (Servicebio, GB23303) at RT for 1 h, and visualized by ECL chemiluminescence (Thermo Fisher). Grayscale intensities were quantified via ImageJ to determine protein expression.

hPLMFs were seeded at 1 × 10⁴ cells/well in 6-well plates with 10% FBS-DMEM, incubated overnight for adherence, and co-cultured with serum-free DMEM, cCSF, or gCSF for 36 h. After PBS washing, nuclear proteins were extracted using the kit (Thermo Fisher, 78833), with subsequent steps identical to total protein analysis. Membranes were incubated overnight at 4 °C with anti-P65 (Servicebio, GB11997) and at RT for 1 h with anti-histone H3 (Servicebio, GB11102) (HRP-conjugated), followed by ECL detection. hPLMFs were seeded and incubated overnight, treated with 10 μM DHMEQ (MCE, 287194-40-5) for 24 h, washed with PBS, and co-cultured with serum-free DMEM, cCSF, or gCSF for 36 h. Total proteins were extracted, and fibrosis-related proteins (COL1A1, COL3A1, β-ACTIN) were analyzed by Western blot as above. hPLMFs were transfected with siRNA (siRNA#1: GCUGCAGUUUGAUGAUGAATT; siRNA#2: GAGUCAGAUCAGCUCCUAATT; siRNA#3: CUUCCAAGUUCCUAUAGAATT) (siRNA-001-01, D-Nano) using CALNP RNAi transfection reagent (DN001-10, D-Nano). A negative control siRNA (siNC: UUCUCCGAACGUGUCACGUTT) (si003-10, D-Nano) was used. 48 h post-transfection, P65 knockdown efficiency was assessed. Cells were then co-cultured with serum-free DMEM, cCSF, or gCSF for 36 h, and fibrosis-related proteins (p-P65, P65, COL1A1, COL3A1, β-ACTIN) were analyzed by Western blot.

#### Quantitative PCR (qPCR)

After siRNA transfection, total RNA was extracted from hPLMFs treated with cCSF/gCSF. RNA concentration and purity (A260/A280 1.8–2.0, *A260/A230* > 2.0) were measured using a Nanodrop, and integrity was verified by 1% agarose gel electrophoresis. RNA was reverse-transcribed into cDNA using PrimeScript™ RT Master Mix (Takara) under the following conditions: 37 °C for 15 min, 85 °C for 5 s (20 μL reaction system). qPCR was performed with SYBR® Premix Ex Taq™ II (Takara) in a 20 μL reaction system containing 2 μL cDNA template (10-fold diluted), 10 μM forward (GAPDH: H-GAPDH-S 5’-GGAAGCTTGTCATCAATGGAAATC-3’; P65: H-P65(1)-S 5’-ACCGGATTGAGGAGAAACGTA-3’) (NM_002046, Servicebio) and reverse primers (H-GAPDH-A: 5’-TGATGACCCTTTTGGCTCCC-3’; H-P65(1)-A: 5’-TCTGCCCAGAAGGAAACACC-3’) (NM_001145138.2, Servicebio), 10 μL 2×SYBR premix, and RNase-free water. GAPDH was used as an internal control. The thermal cycling conditions were: 95 °C for 30 s (pre-denaturation), followed by 40 cycles (95 °C for 5 s, 60 °C for 30 s). Melting curve analysis was performed to confirm specificity, and P65 relative expression was calculated using the 2⁻ΔΔCt method.

#### Cell fluorescence staining

hPLMFs were seeded at 5 × 10⁴ cells/well in confocal dishes containing 10% FBS-DMEM and cultured overnight for adherence. After co-culture with serum-free DMEM, cCSF, or gCSF for 36 h, cells were washed three times with PBS. To observe P65 nuclear translocation, cells were fixed and blocked, followed by incubation with NF-κB/P65 antibody (Beyotime, SN368) overnight at 4 °C. After incubation with Cy3-conjugated anti-rabbit IgG (Beyotime, SN368) at room temperature for 1 hour, cells were stained for nuclei and observed by confocal microscopy. Red fluorescence signals were localized and analyzed using ImageJ.

#### Enzyme-linked immunosorbent assay (ELISA)

ELISA kits were used to measure the relative expression levels of representative secretory proteins in the CSF of healthy mice and CT2A-Luc tumor-bearing mice, including Gdf15 (Elabscience, E-EL-M0604), PDGFC (CUSABIO, CSB-E13404m), PDGFD (CUSABIO, CSB-EL017711MO), TNC (JONLNBO, JL24309-48T), POSTN (CUSABIO, CSB-EL018381MO), and LGALS9 (CUSABIO, CSB-EL012895MO). All procedures strictly followed the manufacturer’s instructions: Mouse CSF samples were collected, centrifuged to remove impurities, and the supernatant was diluted at ratios. Samples were added to ELISA plates precoated with capture antibodies, followed by incubation, washing, and sequential addition of detection antibodies, enzyme-conjugated secondary antibodies, and substrates for color development. Absorbance (OD values) was measured using a microplate reader (Thermo Fisher), and protein concentrations were calculated based on standard curves to determine relative expression levels.

### CSF tracing and clearance function assessment

#### Gadobutrol CSF tracing

Gadobutrol (MCE, ZK135079, 1 mmol/L, 5 μL) was injected into the lateral ventricle of different model mice at stereotactic coordinates (AP: 0.5 mm, ML: 1.0 mm, DV: 2.0 mm). At 10 minutes (initial concentration) and 3 hours post-injection, 10 μL of cisternal CSF was collected using a Hamilton syringe connected to a 33-gauge needle. Gadolinium (Gd) concentrations were measured by inductively coupled plasma mass spectrometry (ICP-MS) using a Perkin Elmer NexION 300D ICP-MS (Perkin Elmer, USA). The measurements were performed in peak hopping mode at a mass-to-charge ratio (m/z) of 157, utilizing an RF power of 1550 W and a nebulizer gas flow rate of 1.05 L/min. Raw counts (cps) were background-subtracted and calibrated against multi-point external standards (*R*^*2*^ > 0.999). Quality control included procedural blanks and duplicates (*RSD* < 5%). Internal standardscorrected for signal drift. The clearance rate was calculated as: *Clearance rate* = [(Cisternal Gd concentration at 10 min - Cisternal Gd concentration at 3 h) / Cisternal Gd concentration at 10 min] × 100%. To assess parenchyma-to-CSF efflux, gadobutrol (1 mmol/L, 5 μL) was injected intraparenchymally. Cisterna magna CSF (10 μL) was collected 4 h after injection with a Hamilton syringe fitted with a 33-gauge needle, and relative Gd levels were measured as described above.

#### Intravital two-photon imaging of leptomeningeal PVS transport

Mice were anaesthetized and placed on a custom head holder. Fur from the head to the upper neck was removed, and the skin was disinfected with povidone-iodine followed by 75% ethanol. Ovalbumin-AF647 (1 mg/mL; Fisher Scientific; O34784; 2 μL) was injected intraparenchymally using a Hamilton syringe over 5 min. The needle was left in place for an additional 5 min before slow withdrawal to minimize backflow and facilitate tracer dispersion. At 2 h after tracer injection, the head was fixed in a custom stereotaxic frame and a cranial window was prepared by removing the skull over the imaging area. FITC-dextran was administered intravenously via the tail vein to label the vasculature. At 3 h after ovalbumin-AF647 injection, leptomeningeal PVS were imaged using a two-photon microscope (A1R MP; Nikon) with a 25× water-immersion objective (NA 1.1, WD 2.0 mm). Image stacks were acquired at 512 × 512 pixels with a z-step of 2 μm.

#### Cervical lymph node drainage assay

Mice were anaesthetized, shaved and disinfected with povidone–iodine followed by 75% ethanol, and then placed in a stereotaxic frame. After a midline scalp incision, a small cranial burr hole was made above the injection site. Ovalbumin-AF647 (1 mg/mL; Fisher Scientific; O34784; 2 μL) was injected intraparenchymally using a Hamilton syringe fitted with a 33-gauge needle. The tracer was delivered slowly over 2 min; the needle was kept in place for an additional 5 min before withdrawal to minimize reflux. Mice were euthanized 1 h after injection. Deep cervical lymph nodes were collected, processed as cryosections, and scanned for fluorescence using a slide scanner (3DHISTECH; Pannoramic MIDI).

### Phase-contrast magnetic resonance imaging

Non-tumor controls and GBM patients underwent phase-contrast MRI (3.0 T MRI, Siemens) at admission and on postoperative day 21 to assess CSF flow velocities in the third, fourth, and lateral ventricles. Flow velocities were calculated from velocity-encoded maps. Non-tumor controls and GBM patients were strictly matched for age and sex.

### Statistical analysis

Quantitative data are expressed as mean ± standard deviation (mean   s.d.). For box plots, the center line represents the median, the box limits denote the 25th and 75th percentiles, and the whiskers extend to the minimum and maximum values; individual data points are plotted alongside. Comparisons between two groups were performed using unpaired two-tailed Student’s *t*-tests. For multiple group comparisons, one-way analysis of variance (ANOVA) followed by Tukey’s multiple comparison test was used. Survival analysis was conducted using Kaplan-Meier curves, and group differences were assessed with Log-rank (Mantel-Cox) tests. Correlation analyses were performed using Pearson correlation co-efficients (linear relationships), with *P* < 0.05 considered statistically significant. Missing values arising from variable follow‑up (e.g., humane endpoints) were treated as missing without imputation; analyses used all available measurements up to the last valid time point, and n per time point is reported in the source data. For each family of related endpoints, multiple-comparison correction was additionally applied using the Holm-Šidák method. Statistical analyses were performed using GraphPad Prism (v10.0.2) and R-Studio. No statistical methods were used to predetermine sample size. Animals were randomly assigned to experimental groups where applicable. Investigators were blinded to group allocation during image acquisition or quantification whenever feasible. All representative micrographs are supported by independent experimental repeats. Unless otherwise indicated, n refers to biologically independent animals, patient samples, or cell preparations.

### Reporting summary

Further information on research design is available in the Nature Portfolio Reporting Summary linked to this article.

## Supplementary information

Supplementary Information (pdf)

Description of Additional Supplementary Files

Supplementary Dataset 1

Supplementary Dataset 2

Supplementary Dataset 3

Reporting Summary

Transparent Peer Review file

## Source data

Source Data

## Data Availability

Raw mass spectrometry proteomics data of CSF samples from non‑tumor controls and GBM patients have been deposited in the iProX database, which serves as a core member of the ProteomeXchange Consortium, under accession IPX0017901000 (https://www.iprox.cn/page/project.html?id=IPX0017901000). These proteomics data are publicly accessible without restriction. The bulk RNA‑seq data from non‑neoplastic human leptomeninges used in this study were obtained as controlled‑access third‑party data from the Synapse platform (syn34512705; https://www.synapse.org) and are not publicly redistributable. The sequencing data generated in this study have been deposited in the National Genomics Data Center (NGDC) under BioProject PRJCA061682 (https://ngdc.cncb.ac.cn). The associated OMIX accession numbers are as follows: Single‑cell RNA‑seq data of tumor tissues from CT2A tumor‑bearing mice: OMIX016098 - publicly open; Bulk RNA‑seq data of human leptomeningeal tissues from GBM patients: OMIX017887 - controlled access; RNA‑seq data of human pia‑arachnoid leptomeningeal fibroblast‑like cells (hPLMFs) co‑cultured with control CSF (cCSF) or GBM‑derived CSF (gCSF): OMIX017890 (https://ngdc.cncb.ac.cn/omix/release/OMIX017890) - controlled access; Single‑cell RNA‑seq data of leptomeningeal tissue from non‑tumor controls and GBM patients: OMIX017990 - controlled access. Consistent with Chinese human genetic resource regulations, the human RNA-seq datasets are deposited under controlled access. Access will be granted to qualified researchers who contact the corresponding author (shizhifeng@fudan.edu.cn) with a brief description of the intended use. Approval by the data owners and execution of a Data Use Agreement (provided as Supplementary Information) are required prior to data transfer. To ensure full transparency and reproducibility, all processed summary data required to support the main conclusions, including differentially expressed genes (Supplementary Dataset 1 and 3) and differential protein expression (Supplementary Dataset 2), are provided as Supplementary Information and constitute an independently verifiable resource. Source data underlying the figures are provided as a Source Data file. Additional data supporting the findings are available in the Article and Supplementary Information. Source data are provided with this paper.

## Code availability

Custom analysis code for RNA‑seq, based on established bioinformatics packages, is publicly available at https://github.com/maoyuansun/szf_lab_2026.6.
