# Supplementary material for: Leptomeningeal fibroblasts promote glioblastoma progression by regulating cerebrospinal fluid dynamics
Source: Nat Commun. 2026 Aug 26;17:9049. doi: 10.1038/s41467-026-76032-2 (PMC13518310; doi:10.1038/s41467-026-76032-2)
Supplement: Supplementary file 1 [file 41467_2026_76032_MOESM1_ESM.pdf]

Supplementary Information

Leptomeningeal fibroblasts promote glioblastoma progression by regulating cerebrospinal fluid dynamics

Maoyuan Sun[1,2,3#], Shan Jiang[1,2,3#], Wenli Zhang[4#], Zhen Fan[1,2,3#], Beining Liu[1,2,3], Yue Wang[1,2,3], Zhen Li[1,2,3], Yun Guan[1,2,3], Yulai Zeng[1,2,3], Jiawen Chen[1,2,3], Ruize Zhu[1,2,3], Weiqiu Ping[1,2,3], Yanlin Teng[1,2,3], Songlin Yan[1,2,3], Qisheng Tang[1,2,3], Yudan Chi[1], Zhiyong Qin[1,2,3], Houshi Xu[5]*, Chong Liu[6]*, Ying Mao[1,2,3]*, Zhifeng Shi[1,2,3]*

[1]Department of Neurosurgery, Huashan Hospital, Fudan University, Shanghai, China

[2]National Center for Neurological Disorders, Shanghai, China

[3]Neurosurgical Institute, Fudan University, Shanghai, China

[4]Department of Radiology, Second Affiliated Hospital of Chongqing Medical University, Chongqing, China

[5]Department of Neurosurgery, Shanghai General Hospital, Shanghai Jiaotong University, Shanghai, China

[6]Department of Neurosurgery of Second Affiliated Hospital, Zhejiang University School of Medicine, Hangzhou, China

[#]These authors contribute equally.

*Correspondence: Houshi Xu (houshi1996@gmail.com); Chong Liu (chongliu77@zju.edu.cn); Ying Mao (maoying@fudan.edu.cn); Zhifeng Shi (shizhifeng@fudan.edu.cn).

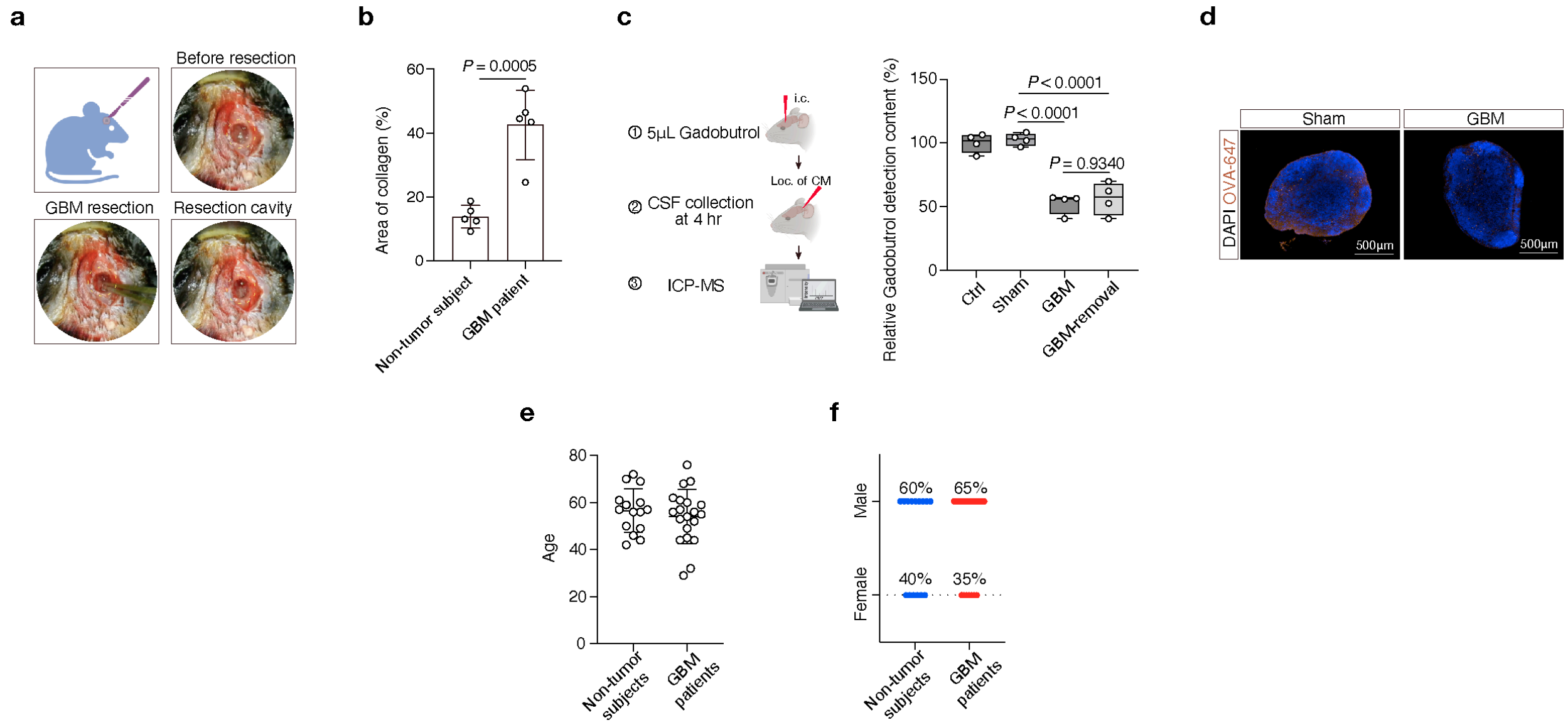

Supplementary Figure. 1 | CSF transport assays in GBM-bearing mice.

a, Schematic of tumour resection in the orthotopic GBM mouse model under microscopy. **b,** Representative images and quantification of PVS and type I/III collagen fibres in leptomeninges from non-tumor subjects and GBM patients (mean ± s.d., *n* = 5 human participants per group); *P* values, unpaired two-tailed Student t-test. **c,** Left, schematic of intraparenchymal gadobutrol injection and cisterna magna CSF collection. Right, relative abundance of gadobutrol in cisterna magna CSF measured 3 h after intraparenchymal tracer injection (*n* = 4 mice per group); *P* values, one-way ANOVA with Tukey's test. Box plots display the median (centre line), 25th and 75th percentiles (box limits), minimum and maximum values (whiskers), with all individual data points. Figure created with BioRender.com. **d,** Representative fluorescence images of deep cervical lymph nodes from sham-operated and GBM tumour-bearing mice after intraparenchymal injection of ovalbumin-AF647. Scale bar, 500 µm. **e-f,** Age (mean ± s.d.) **(e)** and sex **(f)** distribution of non-tumour controls and patients with GBM included in the phase-contrast MRI (PC-MRI) analysis. Source data are provided as a Source Data file.

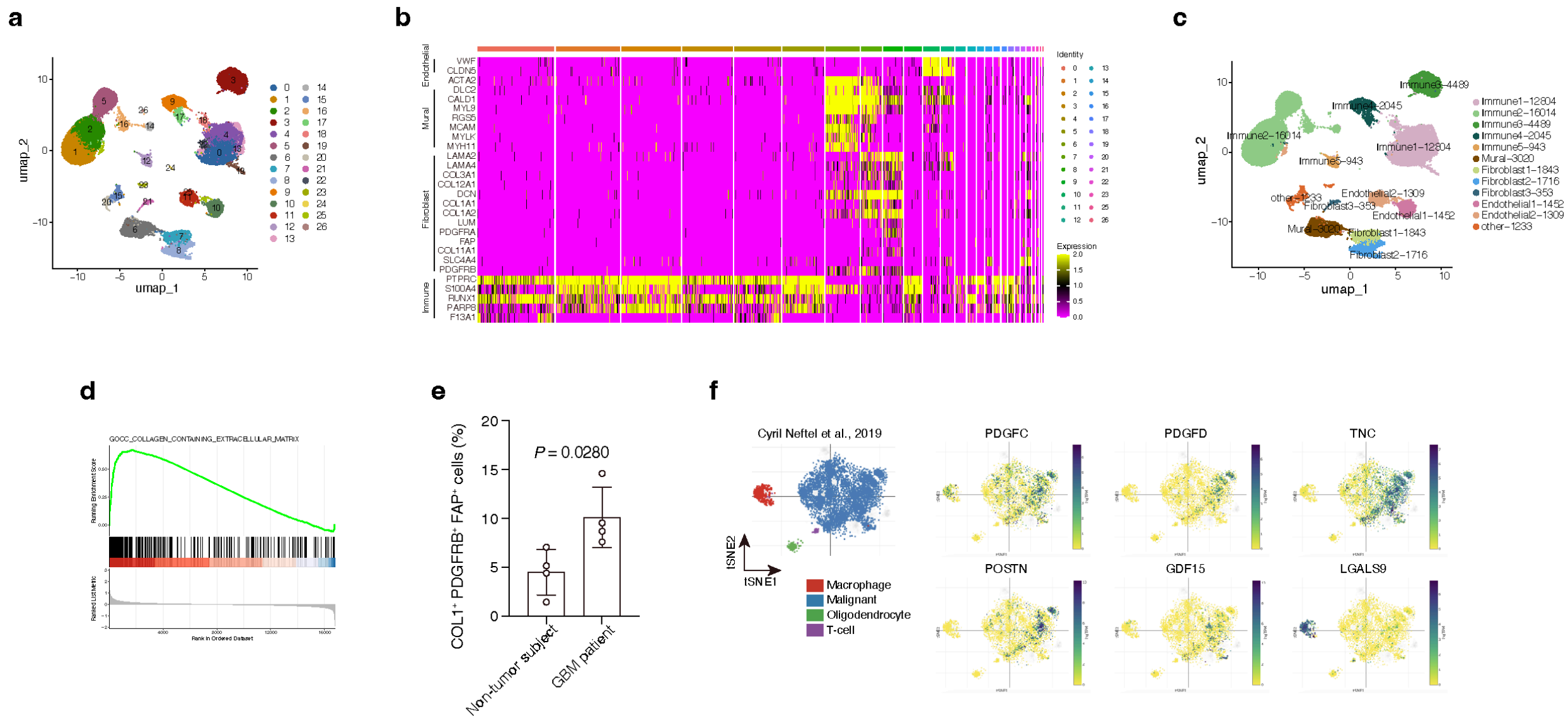

Supplementary Figure. 2 | Single-cell RNA-seq profiling of leptomeningeal tissues and CSF proteomic associations.

a, UMAP of single-cell transcriptomes from leptomeningeal tissues of non-tumour controls (n = 3 human participants) and patients with GBM (n = 3 patients). **b,** Heat map showing expression of canonical marker genes used for annotation of major cell types. **c,** UMAP of single-cell transcriptomes from leptomeningeal tissues of non-tumour controls (n = 3 human participants) and patients with GBM (n = 3 patients), with clusters annotated into distinct cell populations based on canonical marker gene expression. **d,** Gene set enrichment analysis (GSEA) comparing non-tumour control and GBM-associated cell clusters for the GO cellular component term COLLAGEN-CONTAINING EXTRACELLULAR MATRIX; two-sided hypergeometric tests with Benjamini-Hochberg FDR correction. **e,** Proportion of $COL1^{+}PDGFRB^{+}FAP^{+}$ cells in non-tumour subjects and patients with GBM (mean ± s.d., n = 4 human participants per group); P values, unpaired two-tailed Student t-test. **f,** t-SNE plot of single-cell transcriptomes from GBM tumours showing expression of genes encoding CSF proteins upregulated in GBM patient CSF relative to non-tumour control CSF. Source data are provided as a Source Data file.

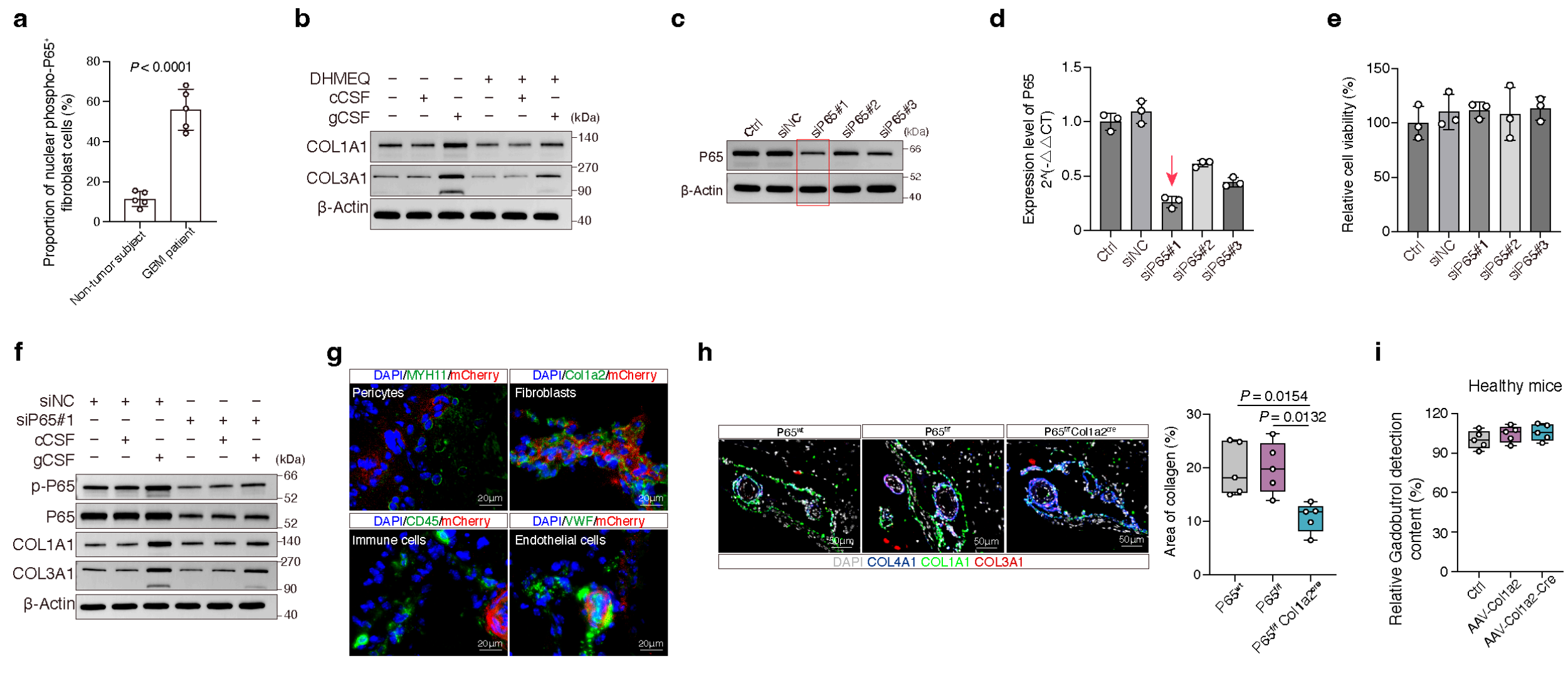

Supplementary Figure. 3 | NF-κB/P65 pathway perturbation in fibroblasts and validation of Col1a2-targeted AAV delivery.

a, Proportion of nuclear phospho-P65+ fibroblast cells in non-tumour subjects and patients with GBM (mean ± s.d., *n* = 5 human participants per group); *P* values, unpaired two-tailed Student t-test. **b,** Immunoblot analysis of COL1A1 and COL3A1 protein in hPLMFs treated with cCSF or gCSF with or without DHMEQ treatment. **c-d,** P65 protein expression **(c)** and RELA mRNA levels **(d)** in hPLMFs after siRNA transfection (mean ± s.d., *n* = 3 independent cell-culture experiments per group). **e,** Cell viability of hPLMFs after siP65 transfection (mean ± s.d., *n* = 3 independent cell-culture experiments per group). **f,** Immunoblot analysis of phosphorylated P65, total P65, COL1A1 and COL3A1 in hPLMFs treated with cCSF or gCSF after siRNA-mediated P65 silencing. **g,** Representative images assessing AAV9-mCherry tropism in leptomeningeal tissues by co-staining with fibroblast, endothelial, mural and immune cell markers. Scale bar, 20 µm. **h,** Left, representative images of type I/III collagen fibres in the leptomeninges of GBM tumour-bearing mice in the indicated groups. Scale bar, 50 µm. Right, quantification of type I/III collagen fibre area in the leptomeninges (*n* = 5 mice per group); *P* values, one-way ANOVA with Tukey's test. **i,** CSF clearance assay in healthy mice from the indicated groups (*n* = 5 mice per group). Box plots display the median (centre line), 25th and 75th percentiles (box limits), minimum and maximum values (whiskers), with all individual data points for Supplementary Figure. 3h, i. Source data are provided as a Source Data file.

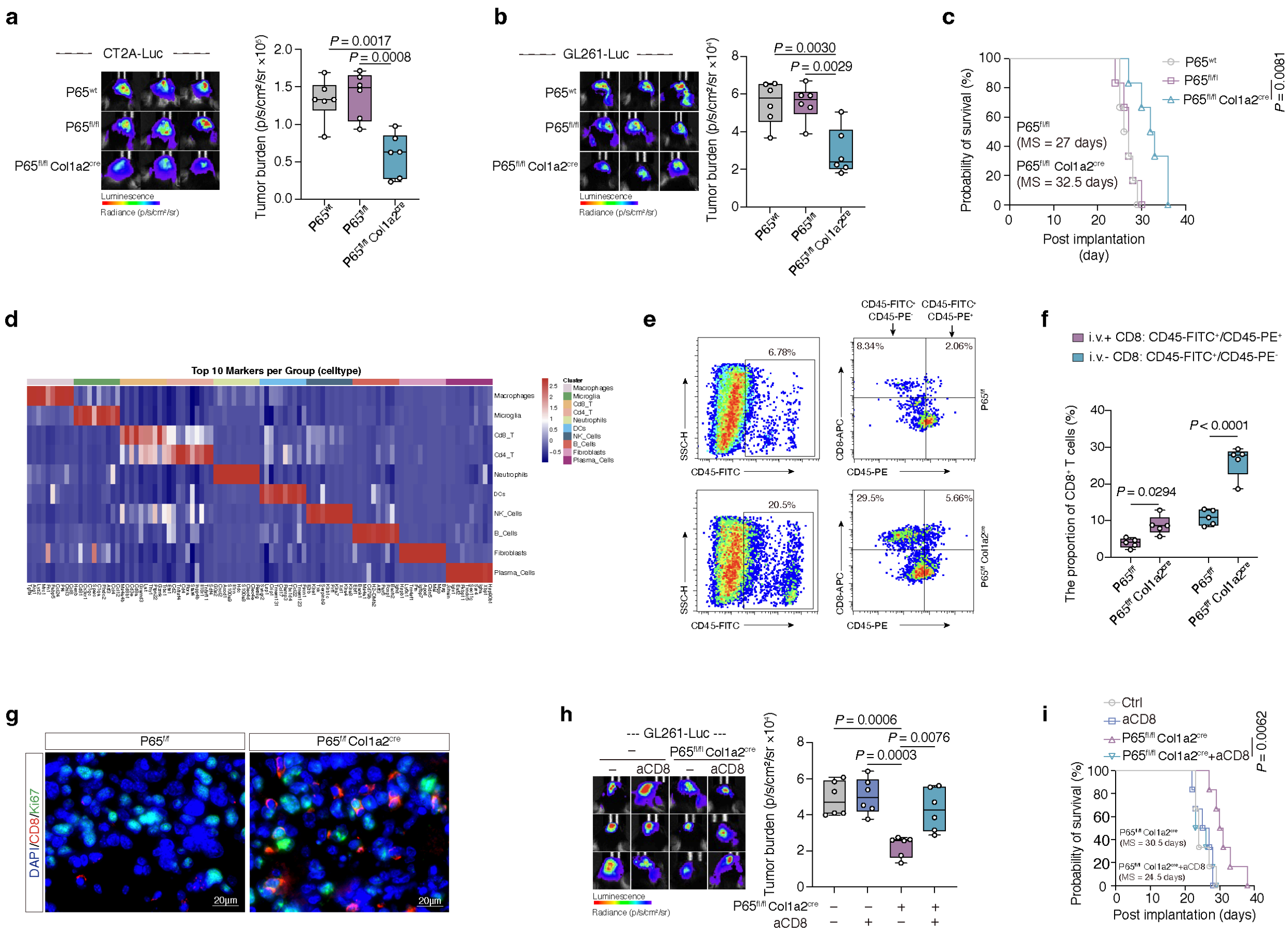

Supplementary Figure. 4 | Complementary tumour and immune analyses in CT2A and GL261 GBM models.

a, Left: representative bioluminescence images of CT2A-Luc tumour-bearing mice in the P65wt, P65$^{f/f}$, and P65$^{f/f}$ + pAAV9-Col1a2-Cre-mCherry groups. Right: quantification of tumour burden (n = 6 mice per group); P values, one-way ANOVA with Tukey's test. **b,** Left, representative bioluminescence images of GL261-Luc tumour-bearing mice in the P65^{wt}, P65$^{f/f}$ and P65$^{f/f}$ + pAAV9-Col1a2-Cre-mCherry groups. Right, quantification of tumour burden (n = 6 mice per group); P values, one-way ANOVA with Tukey's test. **c,** Kaplan-Meier survival curves of GL261-Luc tumour-bearing mice in the P65^{wt}, P65$^{f/f}$, and P65$^{f/f}$ + pAAV9-Col1a2-Cre-mCherry groups (n = 6 mice per group, log-rank test). **d,** Single-cell RNA-sequencing data of CD45^{+} cells in tumour tissues of CT2A-Luc tumour-bearing mice in the P65$^{f/f}$ and P65$^{f/f}$ + pAAV9-Col1a2-Cre-mCherry groups, showing annotation of 10 immune cell populations based on canonical marker genes. **e,** Representative flow cytometry plots for i.v.+ (CD45-FITC^{+}CD45-PE^{+}) and i.v.- (CD45-FITC^{+}CD45-PE^{-}) groups. **f,** Quantification of numbers of both i.v.+ (CD45-FITC^{+}CD45-PE^{+}) and i.v.- (CD45-FITC^{+}CD45-PE^{-}) CD8^{+} T cells (n = 5 mice per group); P values, one-way ANOVA with Tukey's test. **g,** Immunofluorescence staining of Ki67 in intratumoral CD8^{+} T cells in CT2A GBM mice. **h,** Left, representative bioluminescence images of GL261-Luc tumour-bearing mice in the indicated groups treated with IgG isotype control or anti-CD8α antibody. Right, quantification of tumour burden (n = 6 mice per group); P values, one-way ANOVA with Tukey's test. **i,** Kaplan-Meier survival curves of GL261-Luc tumour-bearing P65$^{f/f}$ mice receiving pAAV9-

Col1a2-Cre-mCherry and treated with IgG isotype control or anti-CD8α antibody (n = 6 mice per group, log-rank test). Box plots display the median (centre line), 25th and 75th percentiles (box limits), minimum and maximum values (whiskers), with all individual data points shown for Supplementary Figure. 4a, b, f, h. Source data are provided as a Source Data file.

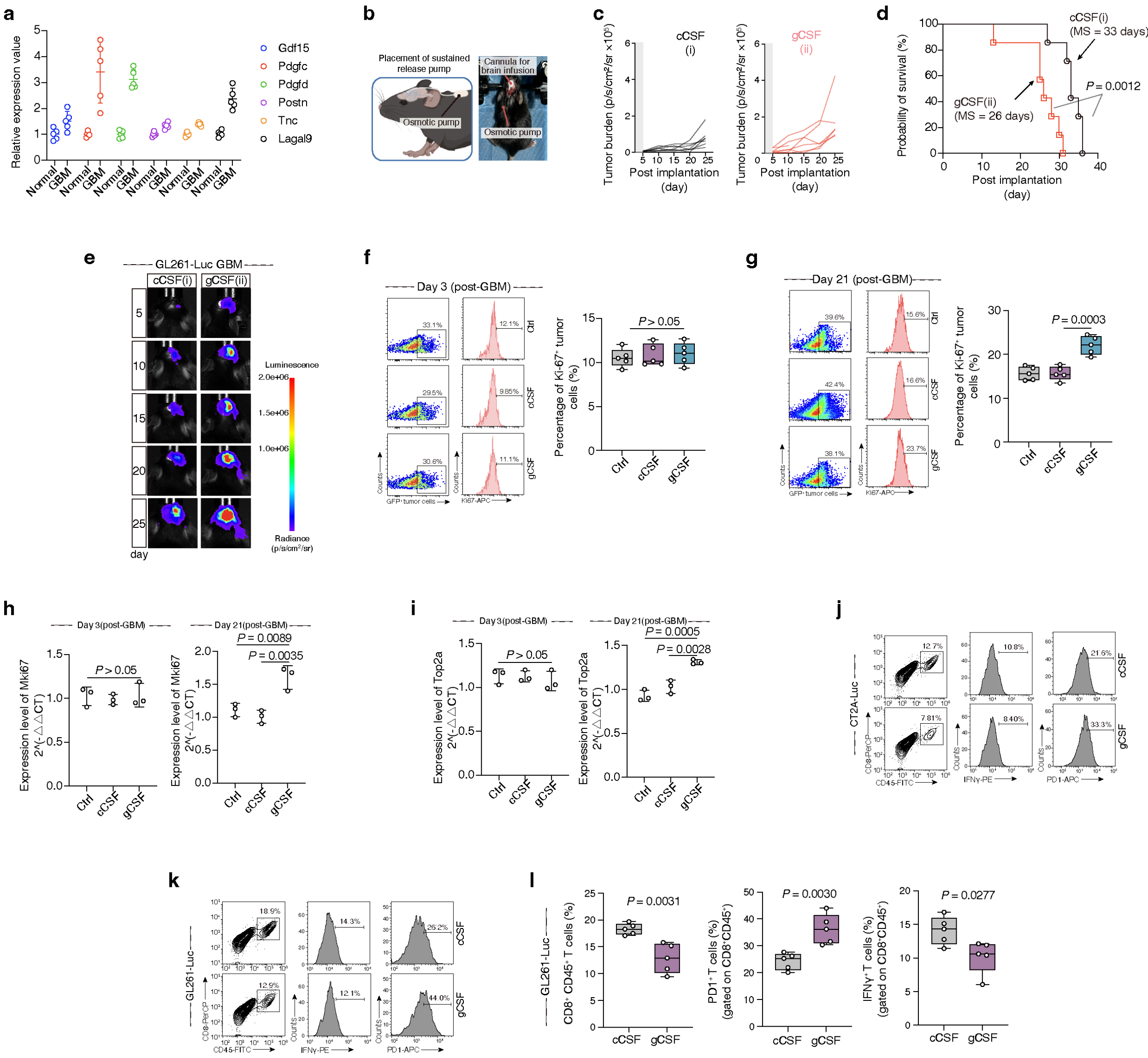

Supplementary Figure. 5 | Additional characterization of the CSF infusion model and associated GBM phenotypes.

a, Expression levels of representative secreted proteins (Gdf15, Pdgfc, Pdgfd, Tnc, Postn and Lgals9) in the CSF of CT2A-Luc tumour-bearing mice (mean ± s.d., *n* = 5 mice per group). **b,** Schematic of the CSF infusion experiment and implantation of osmotic pumps and injection cannulas in the CSF replacement experiment. Figure created with BioRender.com. **c,** Tumour burden in GL261-Luc tumour-bearing mice of the cCSF and gCSF groups (mean ± s.d., *n* = 7 mice per group). **d,** Kaplan-Meier survival curves of GL261-Luc tumour-bearing mice in the cCSF and gCSF groups (*n* = 7 mice per group, log-rank test). **e,** Representative bioluminescence images of GL261-Luc tumour-bearing mice in the cCSF and gCSF groups. **f,** Representative flow cytometry plots and quantification of GFP⁺Ki-67⁺ tumour cells at day 3 after tumour implantation (*n* = 5 mice per group); *P* values, one-way ANOVA with Tukey's test. **g,** Representative flow cytometry plots and quantification of GFP⁺Ki-67⁺ tumour cells at day 21 after tumour implantation (*n* = 5 mice per

group); P values, one-way ANOVA with Tukey's test. **h,** qPCR analysis of Mki67 expression in isolated tumour cells at day 3 and day 21 post-implantation (mean ± s.d.; $n = 3$ mice per group); P values, one-way ANOVA with Tukey's test. **i,** qPCR analysis of Top2a expression in isolated tumour cells at day 3 and day 21 post-implantation (mean ± s.d.; $n = 3$ mice per group); P values, one-way ANOVA with Tukey's test. **j,** Representative flow cytometry plots of $CD45^+CD8^+$ cells, IFN-γ^+ cells (gated on $CD45^+CD8^+$), and $PD1^+$ cells (gated on $CD45^+CD8^+$) in tumour tissues of CT2A-Luc tumour-bearing mice in the cCSF and gCSF groups. **k,** Representative flow cytometry plots of $CD45^+CD8^+$ cells, IFN-γ^+ cells (gated on $CD45^+CD8^+$), and $PD1^+$ cells (gated on $CD45^+CD8^+$) in tumour tissues of GL261-Luc tumour-bearing mice in the cCSF and gCSF groups. **l,** Bar graphs showing the proportions of $CD45^+CD8^+$ cells, IFN-γ^+ cells (gated on $CD45^+CD8^+$), and $PD1^+$ cells (gated on $CD45^+CD8^+$) in tumour tissues of GL261-Luc tumour-bearing mice ($n = 5$ mice per group); P values, one-way ANOVA with Tukey's test. Box plots display the median (centre line), 25th and 75th percentiles (box limits), minimum and maximum values (whiskers), with all individual data points shown for Supplementary Figure. 5f, g, i. Source data are provided as a Source Data file.

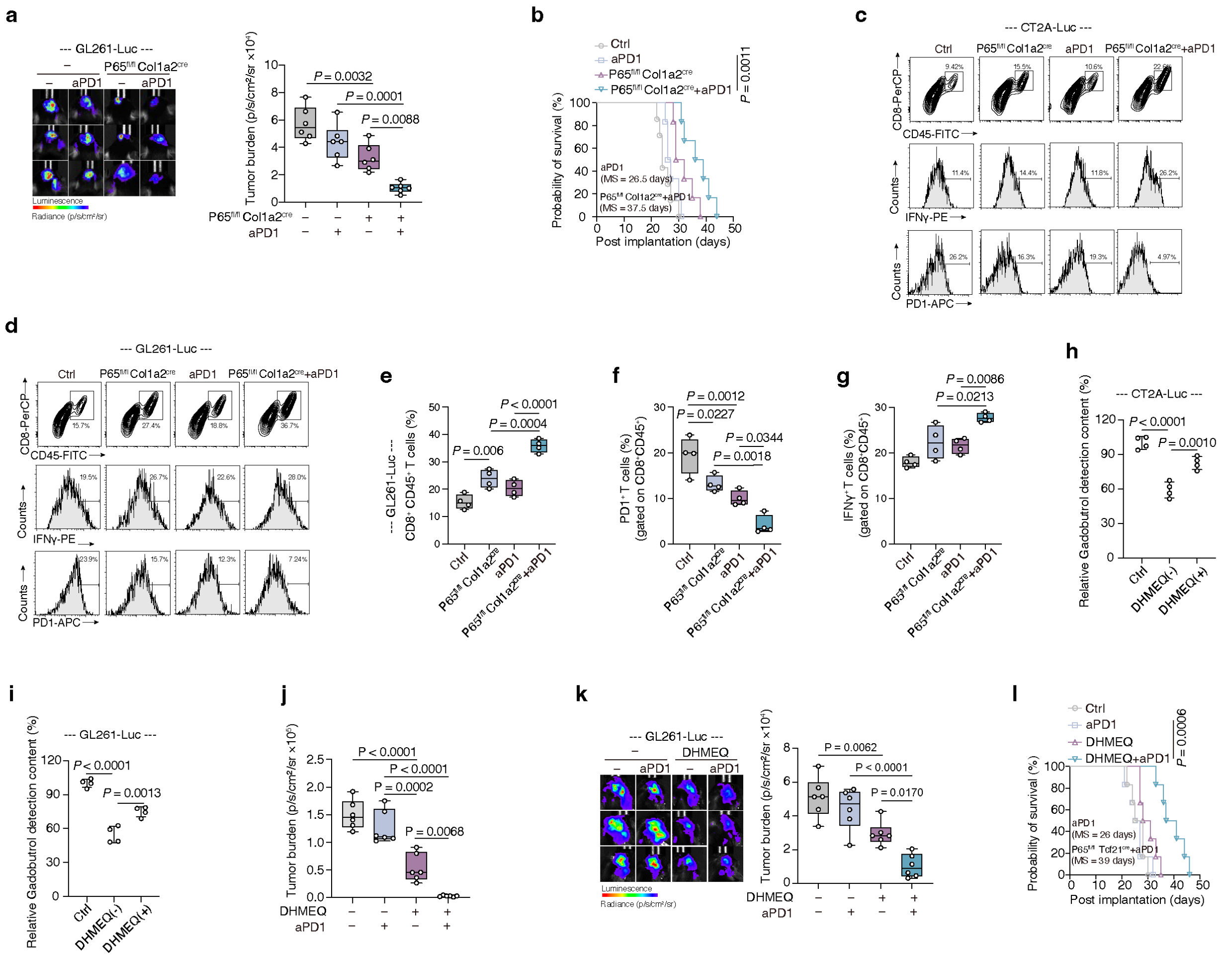

Supplementary Figure. 6 | Additional analyses of genetic and pharmacological NF-κB/P65 targeting in GBM models.

a, Left: representative bioluminescence images of GL261-Luc tumour-bearing mice in the control (Ctrl), aPD1, P65$^{f/f}$ + pAAV9-Col1a2-Cre-mCherry, and aPD1 + P65$^{f/f}$ + pAAV9-Col1a2-Cre-mCherry groups. Right: bar graphs showing tumour burden in these mice (*n* = 6 mice per group); *P* values, one-way ANOVA with Tukey's test. **b,** Kaplan-Meier survival curves of GL261-Luc tumour-bearing mice in the control (Ctrl), aPD1, P65$^{f/f}$ + pAAV9-Col1a2-Cre-mCherry, and aPD1 + P65$^{f/f}$ + pAAV9-Col1a2-Cre-mCherry groups (*n* = 6 mice per group, log-rank test). **c-d,** Representative flow cytometry plots of CD45$^+$CD8$^+$ cells, IFN-γ$^+$ cells (gated on CD45$^+$CD8$^+$), and PD1$^+$ cells (gated on CD45$^+$CD8$^+$) in tumour tissues of CT2A/GL261-Luc tumour-bearing mice in the control (Ctrl), anti-PD-1 monotherapy (aPD1), P65$^{f/f}$ + pAAV9-Col1a2-Cre-mCherry, and aPD1 + P65$^{f/f}$ + pAAV9-Col1a2-Cre-mCherry groups. **e-g,** Quantification of CD45$^+$CD8$^+$ cells, PD1$^+$ cells (gated on CD45$^+$CD8$^+$), and IFN-γ$^+$ cells (gated on CD45$^+$CD8$^+$) in tumour tissues of GL261-Luc tumour-bearing mice in the control (Ctrl), P65$^{f/f}$ + pAAV9-Col1a2-Cre-mCherry, anti-PD-1 monotherapy (aPD1), and aPD1 + P65$^{f/f}$ + pAAV9-Col1a2-Cre-mCherry groups (*n* = 4 mice per group); *P* values, one-way ANOVA with Tukey's test. **h-i,** Quantification of relative gadobutrol content in CT2A-Luc and GL261-Luc tumour-bearing mice treated with vehicle or DHMEQ (mean ± s.d., *n* = 4 mice per group); *P* values, one-way ANOVA with Tukey's test. **j,**

Quantification of CT2A-Luc tumour burden in the Ctrl, DHMEQ, aPD1 and aPD1 + DHMEQ groups (*n* = 6 mice per group); *P* values, one-way ANOVA with Tukey's test. **k,** Left, representative bioluminescence images of GL261-Luc tumour-bearing mice in the control (Ctrl), DHMEQ monotherapy, anti-PD-1 monotherapy (aPD1), and aPD1 + DHMEQ groups. Right, tumour burden in each group (*n* = 6 mice per group); *P* values, one-way ANOVA with Tukey's test. **l,** Kaplan-Meier survival curves of GL261-Luc tumour-bearing mice in the control (Ctrl), DHMEQ monotherapy, anti-PD-1 monotherapy (aPD1), and aPD1 + DHMEQ groups (*n* = 6 mice per group, log-rank test). Box plots display the median (centre line), 25th and 75th percentiles (box limits), minimum and maximum values (whiskers), with all individual data points shown for Supplementary Figure. 6a, e-g, j, k. Source data are provided as a Source Data file.
