# Supplementary material for: Leptomeningeal fibroblasts promote glioblastoma progression by regulating cerebrospinal fluid dynamics
Source: Nat Commun. 2026 Aug 26;17:9049. doi: 10.1038/s41467-026-76032-2 (PMC13518310; doi:10.1038/s41467-026-76032-2)
Supplement: Supplementary file 2 [file 41467_2026_76032_MOESM2_ESM.pdf]

Description of Additional Supplementary Files

Supplementary Dataset 1: Differentially expressed genes (DEGs) between leptomeningeal tissues from glioblastoma (GBM) patients and non-neoplastic human leptomeninges.

Supplementary Dataset 2: Differential protein expression profiling of cerebrospinal fluid (CSF) between non-tumour controls and GBM patients.

Supplementary Dataset 3: RNA-sequencing analysis of human leptomeningeal fibroblast-like cells (hPLMFs) co-cultured with control CSF (cCSF) or GBM-derived CSF (gCSF), highlighting DEGs between the two groups.
