# Supplementary material for: Leptomeningeal fibroblasts promote glioblastoma progression by regulating cerebrospinal fluid dynamics
Source: Nat Commun. 2026 Aug 26;17:9049. doi: 10.1038/s41467-026-76032-2 (PMC13518310; doi:10.1038/s41467-026-76032-2)
Supplement: Supplementary file 3 [file 41467_2026_76032_MOESM6_ESM.pdf]

nature portfolio

Corresponding author(s): Zhifeng Shi

Last updated by author(s): Jun 25, 2026

# Reporting Summary

Nature Portfolio wishes to improve the reproducibility of the work that we publish. This form provides structure for consistency and transparency in reporting. For further information on Nature Portfolio policies, see our Editorial Policies and the Editorial Policy Checklist.

## Statistics

For all statistical analyses, confirm that the following items are present in the figure legend, table legend, main text, or Methods section.

| n/a | Confirmed | |
|---|---|---|
| ☐ | ☒ | The exact sample size (*n*) for each experimental group/condition, given as a discrete number and unit of measurement |
| ☐ | ☒ | A statement on whether measurements were taken from distinct samples or whether the same sample was measured repeatedly |
| ☐ | ☒ | The statistical test(s) used AND whether they are one- or two-sided<br>*Only common tests should be described solely by name; describe more complex techniques in the Methods section.* |
| ☒ | ☐ | A description of all covariates tested |
| ☐ | ☒ | A description of any assumptions or corrections, such as tests of normality and adjustment for multiple comparisons |
| ☐ | ☒ | A full description of the statistical parameters including central tendency (e.g. means) or other basic estimates (e.g. regression coefficient) AND variation (e.g. standard deviation) or associated estimates of uncertainty (e.g. confidence intervals) |
| ☐ | ☒ | For null hypothesis testing, the test statistic (e.g. *F*, *t*, *r*) with confidence intervals, effect sizes, degrees of freedom and *P* value noted<br>*Give P values as exact values whenever suitable.* |
| ☒ | ☐ | For Bayesian analysis, information on the choice of priors and Markov chain Monte Carlo settings |
| ☒ | ☐ | For hierarchical and complex designs, identification of the appropriate level for tests and full reporting of outcomes |
| ☐ | ☒ | Estimates of effect sizes (e.g. Cohen's *d*, Pearson's *r*), indicating how they were calculated |

*Our web collection on statistics for biologists contains articles on many of the points above.*

## Software and code

Policy information about availability of computer code

| | |
|---|---|
| Data collection | BD FACS Diva, v9.0<br>Zen (Zeiss), v3.4<br>NIS-Elements (Nikon), v5.2<br>Spectronaut Pulsar, 18.7<br>Cell Ranger, 9.0.1<br>Illumina NovaSeq Control Software, v4.2<br>BD FACSDiva, v9.0 |
| Data analysis | ImageJ / Fiji Fiji, 2.14.0 / ImageJ, 1.54f<br>FlowJo, v10.9.0<br>GraphPad Prism, v10.0.2<br>R 4.3.0<br>R-Studio, 2023.09.1+494<br>Seurat, 4.4<br>DoubletFinder, v3 (0.2.7)<br>Harmony 0.1.1, (R package)<br>Monocle2, 2.30.0<br>clusterProfiler, 4.10.1<br>limma, 3.58.1(Bioconductor 3.18)<br>edgeR, 4.0.1 (Bioconductor 3.18)<br>DESeq2, 1.42.1 (Bioconductor 3.18) |

sva (ComBat), 3.50.0 (Bioconductor 3.18)
GSVA / ssGSEA, 1.50.0 (Bioconductor 3.18)
BioRender Web-based (accessed 2025–2026)
SnapGene, 7.0.3 / 8.2.2
SeekOne® DD Analysis Software, v1.0
Qubit dsDNA HS Assay, v4.0 (Thermo Fisher)
Bioptic Qsep400 Fragment Analyzer, v2.1

For manuscripts utilizing custom algorithms or software that are central to the research but not yet described in published literature, software must be made available to editors and reviewers. We strongly encourage code deposition in a community repository (e.g. GitHub). See the Nature Portfolio guidelines for submitting code & software for further information.

## Data

Policy information about availability of data

All manuscripts must include a data availability statement. This statement should provide the following information, where applicable:

- Accession codes, unique identifiers, or web links for publicly available datasets
- A description of any restrictions on data availability
- For clinical datasets or third party data, please ensure that the statement adheres to our policy

All de-identified patient clinical information and sample data are presented in the main text and supplementary materials. Raw mass spectrometry proteomics data of CSF samples from non tumour controls and GBM patients have been deposited in the iProX database, which serves as a core member of the ProteomeXchange Consortium, under accession IPX0017901000 (https://www.iprox.cn/page/project.html?id=IPX0017901000). These proteomics data are publicly accessible without restriction.
The bulk RNA seq data from non neoplastic human leptomeninges used in this study were obtained as controlled access third party data from the Synapse platform (syn34512705; https://www.synapse.org) and are not publicly redistributable. The sequencing data generated in this study have been deposited in the National Genomics Data Center (NGDC) under BioProject PRJCA061682 (https://ngdc.cncb.ac.cn). The associated OMIX accession numbers are as follows:
Single cell RNA seq data of tumor tissues from CT2A tumour bearing mice: OMIX016098 (https://ngdc.cncb.ac.cn/omix/release/OMIX016098) - publicly open;
Bulk RNA seq data of human leptomeningeal tissues from GBM patients: OMIX017887 (https://ngdc.cncb.ac.cn/omix/release/OMIX017887) - controlled access;
RNA seq data of human pia arachnoid leptomeningeal fibroblast like cells (hPLMFs) co cultured with control CSF (cCSF) or GBM derived CSF (gCSF): OMIX017890 (https://ngdc.cncb.ac.cn/omix/release/OMIX017890) - controlled access;
Single cell RNA seq data of leptomeningeal tissue from non tumour controls and GBM patients: OMIX017990 (https://ngdc.cncb.ac.cn/omix/release/OMIX017990) - controlled access.
Consistent with Chinese human genetic resource regulations, the human RNA seq datasets are deposited under controlled access. Access will be granted to qualified researchers who contact the corresponding author (shizhifeng@fudan.edu.cn) with a brief description of the intended use. Approval by the data owners and execution of a Data Use Agreement (provided as Supplementary Information) are required prior to data transfer.
To ensure full transparency and reproducibility, all processed summary data required to support the main conclusions, including differentially expressed genes (Supplementary Tables 1 and 3) and differential protein expression (Supplementary Table 2), are provided as Supplementary Information and constitute an independently verifiable resource.
Source data underlying the figures are provided as a Source Data file. Additional data supporting the findings are available in the Article and Supplementary Information.

## Research involving human participants, their data, or biological material

Policy information about studies with human participants or human data. See also policy information about sex, gender (identity/presentation), and sexual orientation and race, ethnicity and racism.

| | |
|---|---|
| Reporting on sex and gender | Human participants included both male and female adults. Sex was assigned at birth and is reported for each participant in the manuscript. Due to the limited cohort size, sex was not used as a biological variable for stratified statistical analysis, but demographic information is provided for transparency. |
| Reporting on race, ethnicity, or other socially relevant groupings | All human participants (patients and non tumor controls) in this study were of Chinese ethnicity. Race and ethnicity were not used as biological variables in the analysis due to the homogeneity of the cohort. No other socially relevant groupings (such as socioeconomic status, disability, or migration status) were collected or analyzed in this study. |
| Population characteristics | The study included adult patients diagnosed with glioblastoma (GBM) who underwent surgical resection at Huashan Hospital, Fudan University. Non tumor control samples were obtained from individuals without malignant brain tumors (e.g., epilepsy surgery patients with normal leptomeninges). Age and sex distributions are provided in the manuscript. All participants were of Chinese ethnicity; no specific population stratification was performed. |
| Recruitment | Patients were recruited from the Department of Neurosurgery at Huashan Hospital, Fudan University.<br>Inclusion criteria: histologically confirmed GBM; age ≥18 years; availability of fresh leptomeningeal tissue and/or tumor tissue; provision of written informed consent. Exclusion criteria: prior radiotherapy or chemotherapy; active systemic infection; severe comorbidities preventing surgery.<br>Non tumor controls were recruited from patients undergoing surgery for non malignant conditions. Recruitment was consecutive and non selective within the inclusion/exclusion criteria. |
| Ethics oversight | The clinical samples used in this study included surgical specimens from treatment naïve GBM patients and non tumour control individuals. The study protocol was approved by the Ethics Review Committee of Huashan Hospital, Fudan University. |

Note that full information on the approval of the study protocol must also be provided in the manuscript.

# Field-specific reporting

Please select the one below that is the best fit for your research. If you are not sure, read the appropriate sections before making your selection.

☒ Life sciences ☐ Behavioural & social sciences ☐ Ecological, evolutionary & environmental sciences

For a reference copy of the document with all sections, see nature.com/documents/nr-reporting-summary-flat.pdf

# Life sciences study design

All studies must disclose on these points even when the disclosure is negative.

| | |
|---|---|
| Sample size | Sample sizes were chosen based on previously published studies in the field and our preliminary data to ensure adequate power for detecting biologically meaningful differences. The number of biologically independent samples (mice, patients, or independent cell preparations) for each experiment is explicitly stated in the figure legends, Source Data, or corresponding Method sections (e.g., n = 10 non-tumor controls and n = 20 GBM patients for CSF proteomics; n = 3 per group for scRNA-seq; specific n values per group for in vivo experiments) |
| Data exclusions | For single-cell RNA sequencing, cells were excluded based on standard quality control criteria: detected genes (nFeature_RNA) outside median ± 3 standard deviations within each sample, or high mitochondrial transcript fraction (percent.mt > 30%). For flow cytometry, single cells were gated based on FSC/SSC characteristics, and only live singlets were included for analysis. No animals or samples were excluded from the analysis post-randomization unless specified (e.g., technical failure of tracer injection or death unrelated to the experimental condition). |
| Replication | All experiments were replicated using biologically independent samples. In vivo experiments were performed with independent biological replicates (number of mice per group stated per experiment); key therapeutic and phenotypic findings were replicated in at least two independent cohorts. In vitro cellular experiments (fibroblast cultures, co-culture, Western blot, qPCR, ELISA) were performed using independently isolated or passaged cell preparations, with each condition repeated in at least three independent biological replicates. Single-cell and bulk RNA-seq, as well as proteomics, were derived from independently collected biological samples (patients or mice). Representative images for Western blots, immunostaining, and flow cytometry are shown from ≥3 independent experimental repeats with similar results.vitro findings replicated in ≥3 independent biological replicates. |
| Randomization | Animals were randomly assigned to experimental groups where applicable (e.g., CSF infusion, osmotic pump implantation, drug intervention, and survival studies). For lineage tracing and conditional knockout studies, littermate controls were used and allocated randomly to experimental arms. Human patient samples were collected consecutively and analyzed based on availability and inclusion criteria. |
| Blinding | Investigators were blinded to group allocation during image acquisition and quantitative analysis (histology, immunofluorescence, confocal microscopy, Western blotting, flow cytometry quantification) whenever feasible. Blinding was not possible during in vivo surgical procedures, stereotactic injections, or osmotic pump implantation, as the operator required real-time identification of genotype, viral construct, or treatment condition to perform the procedure correctly. Similarly, bioinformatic analyses (scRNA-seq, bulk RNA-seq, proteomics) were performed on coded samples, but complete blinding was not possible due to the inherent biological differences between experimental groups. Outcome assessment relied on predefined, objective quantitative metrics. |

# Reporting for specific materials, systems and methods

We require information from authors about some types of materials, experimental systems and methods used in many studies. Here, indicate whether each material, system or method listed is relevant to your study. If you are not sure if a list item applies to your research, read the appropriate section before selecting a response.

## Materials & experimental systems

| n/a | Involved in the study | |
|---|---|---|
| ☐ | ☒ | Antibodies |
| ☐ | ☒ | Eukaryotic cell lines |
| ☒ | ☐ | Palaeontology and archaeology |
| ☐ | ☒ | Animals and other organisms |
| ☐ | ☒ | Clinical data |
| ☒ | ☐ | Dual use research of concern |
| ☒ | ☐ | Plants |

## Methods

| n/a | Involved in the study | |
|---|---|---|
| ☒ | ☐ | ChIP-seq |
| ☐ | ☒ | Flow cytometry |
| ☐ | ☒ | MRI-based neuroimaging |

## Antibodies

| | |
|---|---|
| Antibodies used | Immunofluorescence (IF) / Immunohistochemistry (IHC)<br>Anti-α-SMA, Supplier: Servicebio, Catalog #: GB12045, Dilution: 1:200,<br>Anti-COL4A1, Supplier: Proteintech, Catalog #: 30850-1-AP, Dilution: 1:200,<br>Anti-COL1A1, Supplier: Servicebio, Catalog #: GB154197, Dilution: 1:200,<br>Anti-COL1A2, Supplier: Abcam, Catalog #: AB322963, Dilution: 1:200,<br>Anti-COL3A1, Supplier: Servicebio, Catalog #: GB111629, Dilution: 1:200,<br>Anti-VWF, Supplier: Abcam, Catalog #: AB287962, Dilution: 1:200, |

Anti-FAP, Supplier: Proteintech, Catalog #: 11779-1-AP, Dilution: 1:200,
Anti-PDGFRB, Supplier: Proteintech, Catalog #: 13449-1-AP, Dilution: 1:200,
Anti-CD31, Supplier: Abcam, Catalog #: AB182981, Dilution: 1:200,
Anti-PDGFRA, Supplier: Abcam, Catalog #: AB203491, Dilution: 1:200,
Anti-COL11A1, Supplier: Abcam, Catalog #: AB270946, Dilution: 1:200,
Anti-MYH11, Supplier: Abcam, Catalog #: AB224804, Dilution: 1:200,
Anti-CD45, Supplier: CST, Catalog #: 70257, Dilution: 1:200,
Anti-CD8, Supplier: Servicebio, Catalog #: GB15068, Dilution: 1:200,
Anti-CD8, Supplier: ZSGB-BIO, Catalog #: ZA-0508, Dilution: 1:200,
Anti-PD1, Supplier: PTG, Catalog #: 18106-1-AP, Dilution: 1:200,
Anti-IFN-γ, Supplier: Affinity, Catalog #: DF6045, Dilution: 1:200,
Anti-p-P65, Supplier: Beyotime, Catalog #: AF5875, Dilution: 1:200,
Anti-Ki67, Supplier: Abcam, Catalog #: AB16667, Dilution: 1:200,
Anti-P65, Supplier: Servicebio, Catalog #: GB12997, Dilution: 1:200,
Anti-LAMA2, Supplier: Sigma, Catalog #: L0663, Dilution: 1:100,
Alexa Fluor 488/555/647 Tyramide Reagents, Supplier: Invitrogen/Thermo,
AF647-conjugated Goat Anti-Mouse IgG (H+L), Supplier: Beyotime, Catalog #: A0473, Dilution: 1:500,

Flow Cytometry (FC)
Anti-CD45, Supplier: BioLegend, Catalog #: 157213, Dilution: 1:100,
Anti-CD45, Supplier: BioLegend, Catalog #: 147711, Dilution: 1:100,
Anti-CD8a, Supplier: BioLegend, Catalog #: 100711, Dilution: 1:100,
Anti-CD8a, Supplier: BioLegend, Catalog #: 100733, Dilution: 1:100,
Anti-PD1, Supplier: BioLegend, Catalog #: 135224, Dilution: 1:100,
Live/Dead Viability Dye, Supplier: Thermo Fisher, Catalog #: L34989, Dilution: 1:1000,
Anti-IFN-γ, Supplier: BioLegend, Catalog #: 505807, Dilution: 1:100,

Western Blot (WB)
Anti-COL1A1, Supplier: Servicebio, Catalog #: GB11022, Dilution: 1:1000,
Anti-β-ACTIN, Supplier: Servicebio, Catalog #: GB15003, Dilution: 1:5000,
Anti-P65, Supplier: Servicebio, Catalog #: GB11997, Dilution: 1:1000,
Anti-Histone H3, Supplier: Servicebio, Catalog #: GB11102, Dilution: 1:1000,
Anti-p-P65, Supplier: Beyotime, Catalog #: AF5875, Dilution: 1:200,
HRP-conjugated Goat Anti-Rabbit IgG, Supplier: Servicebio, Catalog #: GB23303, Dilution: 1:5000,

Validation

Commercial antibodies validated by suppliers for WB/IF/FC in mouse/human (manufacturer websites). WB: correct MW bands, uncropped blots in Source Data. IF/IHC: expected cellular localization, isotype/Secondary only controls. FC: positive/negative populations, isotype/FMO gates. Cross validated by independent antibodies (e.g., CD8, CD45) and siRNA knockdown (P65). Secondary antibodies validated by supplier.

## Eukaryotic cell lines

Policy information about cell lines and Sex and Gender in Research

Cell line source(s)

Commercial cell lines: GL261-Luc (Mingzhoubio, MZ8070), CT2A (NOBLEBIO, nobcell0435), and HEbF (otwobiotech, HTX3968) were purchased from commercial suppliers. Authentication was performed by the suppliers using Short Tandem Repeat (STR) profiling, and certificates of analysis were provided.
Derived cell lines: The CT2A-Luc cell line was generated in-house by transducing CT2A cells with a luciferase-expressing lentivirus. Authentication was performed by confirming stable luciferase expression and morphological consistency with the parental CT2A line.
Primary cells: Human primary leptomeningeal fibroblasts (hPLMFs) were isolated from surgically resected human leptomeningeal tissues. Authentication was performed by flow cytometry sorting and verifying fibroblast morphology and marker expression (e.g., COL1A1, Vimentin) during culture.

Authentication

Authentication methods varied by cell type:
STR profiling: Used for all commercial cell lines (GL261-Luc, CT2A, HEbF) to confirm species origin and identity.
Morphological analysis: Used for all cell lines to monitor epithelial/fibroblast-like morphology and growth patterns.
Functional assay: For CT2A-Luc, luciferase activity was confirmed using bioluminescence imaging.
Flow cytometry and immunofluorescence: For hPLMFs, surface marker expression (LAMA2, COL1A1) was used to confirm fibroblast identity.
Mycoplasma testing: All cell lines were routinely tested negative for mycoplasma contamination using PCR-based detection kits.

Mycoplasma contamination

All cell lines used in this study were checked against the International Cell Line Authentication Committee (ICLAC) database of commonly misidentified cell lines (https://iclac.org/databases/cross-contaminations/). None of the cell lines used (GL261-Luc, CT2A, CT2A-Luc, HEbF, hPLMFs) are listed as misidentified or known contaminated cell lines. No cross-contamination issues were identified.

Commonly misidentified lines
(See ICLAC register)

None of the cell lines used in this study (GL261-Luc, CT2A, CT2A-Luc, HEbF, hPLMFs) are listed in the ICLAC register of commonly misidentified cell lines.

## Animals and other research organisms

Policy information about studies involving animals; ARRIVE guidelines recommended for reporting animal research, and Sex and Gender in Research

| | |
|---|---|
| Laboratory animals | The following mouse strains (Mus musculus) were used:<br>Wild type C57BL/6J mice: 6–8 weeks old, sourced from GemPharmatech.<br>P65f/f mice: 6–8 weeks old, C57BL/6J background, sourced from GemPharmatech (floxed P65 allele).<br>Col1a2 P2A CreERT2 PolyA mice: C57BL/6J background, sourced from GemPharmatech.<br>Cspg4 CreERT2 mice: C57BL/6J background, sourced from GemPharmatech.<br>Rosa LSL tdTomato mice: C57BL/6J background, sourced from Shanghai Model Organisms Center (Rosa26 LSL tdTomato reporter).<br>Rosa LSL tdTomato / Col1a2 CreERT2 double heterozygous mice: Generated by crossing Rosa LSL tdTomato homozygotes with Col1a2 CreERT2 heterozygotes; tamoxifen (100 mg/kg i.p.) administered at 6 weeks of age.<br>Rosa LSL tdTomato / Cspg4 CreERT2 double heterozygous mice: Generated by crossing Rosa LSL tdTomato homozygotes with Cspg4 CreERT2 heterozygotes; tamoxifen administered at 6 weeks of age.<br>GBM bearing C57BL/6J mice: Male, 6–8 weeks old, generated by intracranial implantation of GL261 Luc or CT2A Luc cells.<br>P65f/f mice receiving AAV9 Col1a2 Cre mCherry: Male, 6–8 weeks old, generated by cisterna magna delivery of AAV9 Col1a2 Cre mCherry.<br>P65f/f mice receiving control AAV: 6–8 weeks old, generated by cisterna magna delivery of control AAV.<br>Healthy donor mice for cCSF: 6–8 weeks old, non tumour bearing, used for CSF collection.<br>GBM bearing donor mice for gCSF: 6–8 weeks old, tumour bearing, used for CSF collection.<br>All mice were housed under specific pathogen free (SPF) conditions with controlled temperature (22±1°C) and a 12 hour light/dark cycle. |
| Wild animals | N/A. |
| Reporting on sex | All laboratory mice used were male (C57BL/6J background). Sex was not considered in the study design because only male mice were used to avoid hormonal variability; sex specific data disaggregation was therefore not applicable. |
| Field-collected samples | No field-collected samples were used. |
| Ethics oversight | All animal experiments were carried out in accordance with the Health Guide for the Care and Use of Laboratory Animals of Fudan University. Animal protocols were reviewed and approved by the Institutional Animal Care and Use Committee of Fudan University (FLYP-FE23207R). |

Note that full information on the approval of the study protocol must also be provided in the manuscript.

## Clinical data

Policy information about clinical studies
All manuscripts should comply with the ICMJE guidelines for publication of clinical research and a completed CONSORT checklist must be included with all submissions.

| | |
|---|---|
| Clinical trial registration | No. This study did not involve a prospective interventional clinical trial. It is a retrospective observational study using human biospecimens (leptomeningeal tissue, tumor tissue, cerebrospinal fluid) and imaging data collected from patients with glioblastoma (GBM) and non tumor controls under institutional ethics approval. |
| Study protocol | The study protocol is not publicly available due to its retrospective, non clinical trial nature. The general study design is described in the Methods section (Human participants, tissue collection, CSF sampling, imaging acquisition). |
| Data collection | Human biospecimens: Fresh leptomeningeal and tumor tissues were collected during standard surgical resection, immediately processed for single cell suspension, RNA/protein extraction, or fixed for histology.<br>Imaging data: Phase contrast MRI (3.0 T Siemens) was used to acquire velocity encoded maps; CSF flow velocities in third, fourth, and lateral ventricles were calculated.<br>Clinical metadata: Age, sex, diagnosis, and basic demographic information were extracted from electronic medical records.<br>Omics data: scRNA seq , bulk RNA seq , and DIA proteomics were generated from collected samples as described in Methods. |
| Outcomes | As this is a retrospective observational study (not a clinical trial), formal clinical outcomes (e.g., OS/PFS) were not predefined endpoints. |

## Plants

| | |
|---|---|
| Seed stocks | N/A. |
| Novel plant genotypes | N/A. |
| Authentication | N/A. |

## Flow Cytometry

### Plots

Confirm that:

☒ The axis labels state the marker and fluorochrome used (e.g. CD4-FITC).

☒ The axis scales are clearly visible. Include numbers along axes only for bottom left plot of group (a 'group' is an analysis of identical markers).

☒ All plots are contour plots with outliers or pseudocolor plots.

☒ A numerical value for number of cells or percentage (with statistics) is provided.

### Methodology

| | |
|---|---|
| Sample preparation | Mouse tumor tissues were mechanically dissociated (minced with scissors) and enzymatically digested in tumor tissue digestion buffer (bioGenous, K601003) at 37°C for 30 minutes. The digested mixture was filtered through a 70 µm cell strainer, washed with PBS, and treated with ACK Lysing Buffer to remove red blood cells. The final cell suspension was resuspended in PBS and adjusted to a concentration of $1\times10^7$ cells/mL. For flow cytometry staining, the cell suspension was first incubated with 5% FBS/PBS blocking buffer at room temperature for 15 minutes in the dark to reduce nonspecific binding. Staining was performed in two steps: 1) Surface marker and viability staining: The cell suspension was incubated with fluorescently labeled antibodies against CD45 (BioLegend, 157213), CD45 (BioLegend, 147711), CD8a (BioLegend, 100711), CD8a (BioLegend, 100733) and PD1 (BioLegend, 135224) at 4°C for 30 minutes in the dark, together with a viability dye (Thermo Fisher, L34989). After PBS washing, cells were fixed and permeabilized. 2) Intracellular marker staining: Permeabilized cells were incubated with fluorescently labeled antibodies against IFN-γ (BioLegend, 505807) at 4°C for 30 minutes in the dark. After PBS washing, cells were analyzed using a flow cytometer (BD FACS Vantage SE), and data were processed with FlowJo software. |
| Instrument | Data were acquired on a BD FACSCanto II or BD LSRFortessa X-20 flow cytometer equipped with 405 nm, 488 nm, and 640 nm lasers. For sorting, a BD FACSAria III cell sorter was used. |
| Software | Data acquisition was performed using BD FACSDiva software (v9.0). Data analysis was performed using FlowJo software (v10.9.0). |
| Cell population abundance | Leptomeningeal immune profiling typically yielded 50,000–200,000 viable CD45+ cells per mouse. Tumor-infiltrating leukocytes ranged from 100,000–500,000 cells per tumor. For fibroblast sorting (LAMA2+ hPLMFs), approximately 1×10^6 cells were collected per patient sample. Cell viability post-preparation was consistently >85% as determined by viability dye exclusion. |
| Gating strategy | Cells were first gated on the main cell population by FSC-A versus SSC-A to exclude debris, followed by singlet gating. Live CD45+ immune cells were then selected, and CD8a+ T cells were identified within the CD45+ population. PD-1, IFN-γ and Ki67 expression was subsequently analyzed in the CD8a+ cell population. Appropriate isotype/unstained controls were used for gate setting where applicable. |

☒ Tick this box to confirm that a figure exemplifying the gating strategy is provided in the Supplementary Information.

## Magnetic resonance imaging

### Experimental design

| | |
|---|---|
| Design type | Phase-Contrast Magnetic Resonance Imaging (Sagittal Fast PC Cine) |
| Design specifications | Non-tumor controls and GBM patients underwent phase-contrast MRI (3.0 T MRI, Siemens) at admission and on postoperative day 21 to assess CSF flow velocities in the third, fourth, and lateral ventricles. |

| | |
|---|---|
| Behavioral performance measures | N/A. |

## Acquisition

| | |
|---|---|
| Imaging type(s) | Functional MRI |
| Field strength | 3.0 T |
| Sequence & imaging parameters | Flow velocities were calculated from velocity-encoded maps (mean velocity) |
| Area of acquisition | N/A. |

Diffusion MRI ☐ Used ☒ Not used

## Preprocessing

| | |
|---|---|
| Preprocessing software | 3.0 T MRI, Siemens |
| Normalization | N/A. |
| Normalization template | N/A. |
| Noise and artifact removal | N/A. |
| Volume censoring | N/A. |

## Statistical modeling & inference

| | |
|---|---|
| Model type and settings | N/A. |
| Effect(s) tested | N/A. |

Specify type of analysis: ☐ Whole brain ☒ ROI-based ☐ Both

Anatomical location(s): Third, fourth, lateral ventricle.

| | |
|---|---|
| Statistic type for inference (See Eklund et al. 2016) | For multiple group comparisons, one-way analysis of variance (ANOVA) followed by Tukey's multiple comparison test was used. |
| Correction | N/A. |

## Models & analysis

| n/a | Involved in the study | |
|---|---|---|
| ☒ | ☐ | Functional and/or effective connectivity |
| ☐ | ☒ | Graph analysis |
| ☒ | ☐ | Multivariate modeling or predictive analysis |

| | |
|---|---|
| Graph analysis | Graphs were generated and statistical analyses were performed using GraphPad Prism. Final figures were assembled in Adobe Illustrator. |
