# Supplementary material for: Leptomeningeal fibroblasts promote glioblastoma progression by regulating cerebrospinal fluid dynamics
Source: Nat Commun. 2026 Aug 26;17:9049. doi: 10.1038/s41467-026-76032-2 (PMC13518310; doi:10.1038/s41467-026-76032-2)
Supplement: Supplementary file 4 [file 41467_2026_76032_MOESM7_ESM.pdf]

nature portfolio

Peer Review File

Leptomeningeal fibroblasts promote glioblastoma progression by regulating cerebrospinal fluid dynamics

Corresponding Author: Dr Maoyuan Sun

This file contains all reviewer reports in order by version, followed by all author rebuttals in order by version.

Version 0:

Reviewer comments:

Reviewer #1

(Remarks to the Author)
This study investigates the impact of GBM on cerebrospinal fluid (CSF) dynamics and clearance, and proposes that leptomeningeal fibroblasts, via NF-κB/P65 signaling, can modulate the immune microenvironment. This suggests a potential axis of "leptomeninges - CSF - immunosuppression." Overall, the topic is of potential significance, but there are several gaps in the evidence chain, and some key conclusions lack direct support. Below, I outline the major and minor concerns.

1.Evidence for the conclusion that "CSF clearance impairment is independent of tumor mass effect and associated with non-tumoral regions"
The authors report that CSF clearance impairment persists in mice even after surgical tumor resection, and based on this observation, they propose that GBM pathology disrupts leptomeningeal-mediated CSF drainage in non-tumoral regions independently of tumor mass effect. However, several concerns arise:
1)Methodology does not specifically indicate which CSF pathway is affected.
The currently recognized CSF clearance pathways include arachnoid villi absorption, meningeal lymphatics, perineural routes, glymphatic pathways, and drainage to deep cervical lymph nodes. The authors measured CSF clearance by injecting gadolinium into the lateral ventricles and sampling from the cisterna magna, which mainly reflects classical ventricular–subarachnoid flow. GBM, especially when not compressing the ventricular system, primarily affects glymphatic and leptomeningeal outflow pathways. Attributing the observed impairment to leptomeningeal or glymphatic dysfunction lacks methodological justification. The authors need to clarify:
a. Which specific CSF clearance pathway does the reported "CSF clearance impairment" correspond to?
b.Can the current experimental method distinguish between ventricular pathway, leptomeningeal pathway, and glymphatic pathway?
c. Why is this method appropriate to infer leptomeningeal clearance impairment?
2) Persistence of post-resection abnormality does not directly indicate origin from non-tumoral regions
GBM is highly infiltrative, and tumor boundaries are generally indistinct. It is unclear whether peri-tumoral tissues post-resection are free of residual tumor cells. How can the authors exclude the influence of residual tumor cells or secondary pathological changes (e.g., inflammation, microenvironment remodeling) on CSF pathways? More direct evidence is needed to support that the observed abnormality indeed originates from "uninfiltrated normal regions."
3)Does leptomeningeal structural alteration depend on tumor location?
The authors propose that GBM disrupts leptomeningeal drainage structures but do not address whether this effect depends on tumor location. For superficial cortical tumors, direct perturbation of the leptomeningeal structures and pial perivascular spaces is plausible. However, for deep-seated tumors (e.g., thalamus or basal ganglia), it is unclear whether similar leptomeningeal remodeling, ECM deposition, or fibroblast activation can occur. The authors are encouraged to clarify:
a.Does tumor location influence the proposed mechanism of leptomeningeal structural damage?
b.If deep-seated tumors can also induce leptomeningeal changes, what are the potential biological pathways (e.g., diffusion of inflammatory factors, CSF-mediated exosome transport, PVS–leptomeningeal continuity remodeling)?
c.Provide MRI images showing tumor implantation sites to clarify spatial relationships between tumor foci and leptomeninges, supporting inferences about leptomeningeal drainage impairment.
d.If all models involve cortical/superficial implantation, the discussion should specify whether this mechanism is generalizable to deep-seated tumors.

2. Inadequate explanation of the dissociation between increased CSF flow velocity and reduced clearance

The authors report that GBM patients show significantly elevated CSF flow velocities in the third and fourth ventricles, but not in the lateral ventricles; post-resection, only the fourth ventricle remains elevated. They suggest that CSF flow velocity may be "decoupled" from functional clearance. This raises several questions:
1)Why is only part of the ventricular system affected? Why does lateral ventricular flow remain unchanged while third and fourth ventricles increase? Could this reflect tumor location, ventricular anatomy, imaging angle, or pulsatile flow propagation differences? Moreover, post-resection, lateral and third ventricles normalize, but fourth ventricle remains elevated. Is this single-ventricle abnormality sufficient to claim "physiological dysfunction"?
2)Does a single ventricle's flow change reflect overall CSF dynamics? Can the authors demonstrate that flow changes in a single ventricle represent global CSF dysfunction? If not, overinterpretation should be avoided.
3)Mechanistic discussion and validation of the "dissociation" phenomenon:
The observed increase in CSF flow velocity concurrent with decreased clearance is interesting but seemingly contradictory. The discussion lacks mechanistic exploration. For instance, could this reflect "ineffective turbulence" in CSF circulation or obstructive high-pressure in outflow pathways? Based on the findings, increased flow with slowed clearance might lead to CSF accumulation and elevated intracranial pressure (ICP). Do the authors have ventricular enlargement or direct ICP measurements to support this? Speculative discussion and supporting data would substantially strengthen the manuscript.

3. Abbreviations
1) The abbreviations P65wt, P65f/f, P65f/fTcf21Cre appear in lines 247–249 but should be defined at first occurrence (lines 235–236).
2) CSF (Cerebrospinal Fluid) should be defined at first mention in the text (line 296 is too late).

4.Consistency and statistical rationale of animal data
1)Incomplete methodological description:
Methods lack specific details for bioluminescence imaging of tumor size (e.g., instrument model, luciferin dose and timing, image analysis). Please provide these details.
2)Data inconsistencies:
In the GL261 model, Extended Data Fig. 4e showed no tumor size difference between P65f/fTcf21Cre + anti-CD8 groups and P65f/fTcf21Cre groups, yet Extended Data Fig. 4f showed a survival difference. Clarify whether this is from the same cohort of animals. If yes, how can tumor size remain unchanged while survival differs significantly? Are there non-tumor causes of death? If not, the lack of synchronization should be noted.
3)Impact of outlier time points on statistics:
In Fig. 5e and Extended Data Fig. 5c, individual animals have measurement timepoints significantly earlier than others (e.g., one CT2A mouse in Fig. 5e measured only until day 28; one GL261 mouse in Extended Data Fig. 5c measured only until day 10). The authors should clarify:
a.Are these due to early death or technical failure?
b.Were they handled in the statistical analysis (e.g., excluded or noted)?
c.Do they affect the robustness of the overall statistical conclusions?

5. Unclear rationale for gCSF infusion time points
1) Ambiguity of day 5 vs. day 10 and lack of explanation for day 10. In Fig. 5b–c, the authors chose to assess collagen and CSF clearance rate on day 5 post-gCSF infusion initiation, but the figures include data for day 10 without corresponding explanation in the main text. It must be clarified whether "day 10" refers to observation after 10 days of continuous infusion, or observation until day 10 after tumor implantation performed on day 5 of continuous infusion (i.e., 5 days post-implantation). Current description is unclear.
2)Rationale for choosing day 5 for assessment. Infusion of 100µL at 0.5µL/hr takes approximately 8 days to complete. Why was assessment performed on day 5, before pump exhaustion? The CSF clearance rate is reported as identical on days 5 and 10. When did the difference emerge between days 1 and 5? Is there dynamic observation data supporting day 5 as a key timepoint? The rationale for selecting this specific timepoint needs to be explicitly stated.

6.Experimental validation of database analysis
The authors utilize a published single-cell database to conclude that gCSF does not enhance tumor cell proliferative capacity. This is a valuable finding; however, validating this using the study's own animal model samples (e.g., by performing RNA sequencing or qPCR on key genes related to "Quiescent.Score" and "Proliferation.Score" in tumor cells isolated from mice infused with cCSF or gCSF) would significantly strengthen the originality and persuasiveness of this conclusion. The authors are encouraged to consider supplementing the manuscript with such experimental data.

Reviewer #2

(Remarks to the Author)
This manuscript by Sun et al. establishes a causal link between GBM progression and NF κB/p65-dependent leptomeningeal perivascular fibrosis, which leads to CSF clearance defects, $CD8^{+}$ T cell exhaustion, and reduced anti PD 1 efficacy. The authors support these findings with evidence from orthotopic GBM mouse models, human CSF proteomics, leptomeningeal transcriptomics, and paired patient leptomeningeal–tumor analyses. Key findings include:
• Mice with orthotopic GBM tumors, or following tumor resection, exhibit reduced CSF clearance and drainage to lymph nodes and blood, as well as increased leptomeningeal collagen and α-SMA+ fibroblasts.
• GBM patient CSF displays a pro fibrotic, NF κB–dependent protein signature that drives human leptomeningeal fibroblasts toward $CTHRC1^{+}\alpha\ SMA^{+}$ perivascular myofibroblasts in vitro. Similar molecular signatures are observed in the CSF of GBM-bearing mice, supporting the use of these mouse models in testing therapies targeting the CSF–leptomeningeal–immune axis.

• Tcf21 specific p65 ablation reduces leptomeningeal collagen, restores CSF clearance, increases intratumoral $CD8^+$ T cell infiltration, and immune effector signatures. This loss of p65 attenuates GBM growth and improves survival, and this effect is dependent on T cell engagement, as CD8 cell depletion eliminates the survival benefit.
• Conditional p65 knockout and CSF DHMEQ enhance anti-PD-1 therapy and increase functional CD8+ T cells in mice. In patients, leptomeningeal fibrosis correlates with exhausted T-cell signatures.

Strengths:
These findings are significant for GBM, CSF biology, and CNS barrier and immune research, as they demonstrate a role for lymphatic dynamics in GBM tumor immunology and establish leptomeningeal fibroblasts as key regulators of brain tumor immunity. They also identify NF κB /p65 as a potential therapeutic target to remodel the immunosuppressive GBM microenvironment and enhance sensitivity to immune checkpoint inhibition.
The experimental design is mechanistically sound and robust. The authors' major claims are supported by data from multiple independent and complementary approaches, and there is sufficient detail in the methodology to support the reproducibility of the reported findings. Statistical analysis methods are clearly specified and consistently described throughout.
The use of Tcf21 fibroblast specific p65 deletion, as well as a newly established CSF replacement model to mimic CSF-induced clearance impairment, enables the authors to distinguish tumor intrinsic from CSF/meningeal mediated effects on GBM progression and provides greater mechanistic clarity vs. prior (bulk) analyses of GBM lymphatics or the tumor microenvironment.
Overall, these studies and the conceptual advances they present advance mechanistic insight and therapeutic potential in GBM neuro-oncology, immuno-oncology, and CNS barrier research.

Despite these noted strengths, several issues in the manuscript should be addressed:
• The data support a causal link between this axis and GBM progression and immune checkpoint inhibitor resistance. However, claims regarding generalizability across GBM subtypes and potential clinical shunting strategies are less well supported and overstated.
• The authors show that p65 loss in leptomeningeal fibroblasts does not affect healthy mice. However, off target effects due to recombination in other $Tcf21^+$ populations cannot be completely excluded. Additional marker co staining or lineage tracing data are needed to confirm that the phenotype is fibroblast specific.
• MRI data from patients show increased ventricular flow velocities, but CSF clearance is not directly measured. Therefore, the authors should avoid overinterpreting the data to conclude that flow velocity causally impacts CSF clearance.
• Although the patient cohorts studied are modest in size, the authors control for age and sex and use external controls for RNA seq analysis. However, they do not adequately address potential confounders, such as GBM molecular subtypes and the effects of treatment (e.g., steroids) on immune and CSF phenotypes. A more in-depth clinical analysis and/or a discussion are recommended.
• Proteomics data suggest a role for PDGFC, PDGFD, and LGALS9, and PDGFRβ is expressed on perivascular fibroblasts. However, functional analyses (e.g., experiments using PDGFR inhibition or neutralizing antibodies) would be needed to validate these factors as molecular drivers. In the absence of additional mechanistic data, referring to these newly identified proteins as markers, rather than drivers, would be more accurate and avoid overstating conclusions.
• In their statistical analyses, the authors use simple unpaired t tests or ANOVA across many imaging and flow cytometry results that have multiple endpoints (e.g., different collagen metrics, immune cell subsets, activation markers). Without any global correction applied across all panels or families of related tests, this raises a multiple-comparisons issue that can potentially inflate type I error (rejecting a true null hypothesis and concluding there is a real effect or difference when, in fact, there is none). As such, to enhance rigor, detailed explanations of corrections beyond Tukey's post tests (e.g., Holm–Šidák, Benjamini–Hochberg FDR) are needed, along with specifying exact sample sizes and effect sizes in each panel.
• A prior publication demonstrated that the therapeutic effect of anti-PD-1 blockade in GBM is mediated by macrophage/microglia M1 polarization, rather than by CD8 T cells (Rao et al., 2020, PMID: 32554515). The authors' findings and potential involvement of the innate immune system should be discussed and interpreted in this context.
• Further discussion and integration of the findings with recent single-cell leptomeningeal analyses and studies of meningeal lymphatic modulation would greatly strengthen the manuscript.
In summary, this study is sound and mechanistically detailed, and its main conclusions are well supported by the data. The primary concerns relate to interpretation and conclusions (e.g., regarding specificity) rather than critical flaws. Revisions should clarify the specificity of fibroblast targeting and address potential clinical confounders, such as tumor subtype and adjuvant steroid therapy that could affect results and/or data interpretation. Further experiments or more cautious language are also needed for discussing specific CSF factors, and the interpretation of the relationship between ventricular flow velocity and CSF clearance in patients.

Reviewer #3

(Remarks to the Author)
The manuscript by Sun, Jiang, Zhang, Fan et al. introduces a concept in which the progression of GBM, a particularly aggressive brain tumor type, is regulated by leptomeningeal fibroblasts. These fibroblasts respond to the tumor, forming fibrosis that alters CSF clearance dynamics. While experimentally compelling, the manuscript in its current form has several conceptual gaps that I recommend addressing.

Major comments:
1. Origin of fibrosis: The authors invoke leptomeningeal and perivascular fibrosis several times, interchangeably. These two pathologies are distinct, and they should perform formal lineage tracing to delineate the origin of the fibrotic tissue deposited

in response to GBM. This is a critical point for the submitted manuscript. See examples of such tracing in PMID: 33526922.

2. Critical fibrosis mediator: The authors use GBM-derived CSF, human or mouse, with a somewhat delineated composition, to induce the phenotype in most experiments. They call out several proteins that can be responsible for this pathophysiological behavior, such as PDGFs or galectins. Which of these (or which minimal combination and at what concentration) mediates this phenotypic switch in vitro and in vivo? Also, the authors refer to these factors as “aberrant substances”, which is not necessarily true, as they have clear roles in physiology. Better phrasing, such as tumor-derived (if correct), would be appreciated.

3. Sham controls: What is the situation regarding clearance and fibrosis in non-tumor-bearing mice that underwent surgery?

4. Core drivers of fibrosis: The authors suggest that fibroblasts are the core drivers of fibrosis. While technically correct, this process is instructed and orchestrated by the immune cells. Is this phenotype reproducible in immunocompromised (i.e., NSG) animals? And human xenografts? How do resident immune cell types, such as macrophages, contribute to or regulate this process?

5. GBM models: This brings me to another vital issue – the choice of the correct model. Both model lines used in this study elicit a severe immune response, atypical for human GBM. Is this phenomenon reproducible in less immunogenic models? Such as xenografts or other murine lines, as asked above.

6. “Pathological fibroblasts” and single-cell analysis: What evidence do the authors have for CTHRC1/SMA fibroblasts to be pathological, besides their position in UMAP? UMAP does not allow quantitative measurements. If these are assumptions, they should be experimentally proven (i.e., by CTHRC1/SMA fibroblast-lineage depletion). In addition, the single-cell analyses do not meet contemporary standards. Cell type annotations are insufficient, methods do not allow full reproducibility, filtering steps are unclear, quality metrics are not provided, the number of retained cells cannot be found…

7. Cre reporter: What is the reasoning behind the Cre reporter choice? Tcf21 has been described to be expressed in other types of fibroblasts (fibroblasts in choroid plexus and parenchymal perivascular fibroblasts), not leptomeningeal fibroblasts. How do the authors explain their findings, considering this article PMID: 37776854? This suggests that these fibrotic lesions may not be of leptomeningeal origin. Again, tracing here is necessary.

8. c/gCSF infusions: These infusions take several days. Is c/gCSF stable for such a long time at body temperature? Please provide quality metrics (i.e., proteomics and stability measurements for Dgf15, Pdgfc, Pdgfd, Tnc, Postn, and Lgals9) for these experiments.

9. Impact on CD8 T cells: The performed experiments are somewhat correlative. What is the reason for increased CD8 T-cell activity? Is it more local T cell expansion, more infiltration, or better antibody penetrance? The mechanism has to be uncovered. It does not appear to be direct immunosuppression but rather T cell exclusion, a different phenomenon. The causal axis “leptomeninges – CSF – immunosuppression” is therefore incorrect, plus there would be no such causal axis with only these terms. This should be worded more precisely, focusing on the actual results rather than hypothetical mechanisms. And again, this must be repeated with at least one less immunogenic mouse GBM model.

Minor comments:
1. The reference number for local ethical approval (clinical samples) is missing, as is the mouse study approval.
2. Did the authors use male or female mice? The methods are confusing in this regard.
3. How was the speed of c/gCSF infusion decided?
4. How was the dose of infusion pirfenidone calculated?
5. Which of the procedures (and how) directly interfere with the studied phenomena? I would assume that injections/collections/infusions perturb the normal clearance rates. How was this controlled for or corrected?

Reviewer #4

(Remarks to the Author)
General strengths
This study addresses an important unmet need and clinical problem.
The causal logic for the study is clear and compelling and was tested at various levels (human → mouse → cellular → molecular → pharmacologic → therapeutic).
There were multiple types of readouts of CSF clearance (ICP-MS, imaging, lymph node drainage).
The genetic specificity by fibroblast targeting was a major strength.
The immune checkpoint inhibitor and NF-κB subunit P65 inhibitor synergy was extremely compelling.

General areas for improvement
The Discussion should address:
CSF clearance might occur through multiple routes. Fibrosis might be one gatekeeper, but not the only one.
Other meninges resident cells may be responding to CSF, in addition to fibroblasts.
NF-κB subunit P65 is ubiquitous. In clinical practice, it may be difficult to target the NF-κB subunit P65 inhibitor to only GBM fibroblasts because the drug is not fibroblast-specific.

+ Strengths
- Areas for improvement

Introduction
+
The mechanistic logic is strong and goes from a clinical observation, to structural explanation, to molecular changes, and finally therapeutic relevance.
-
Add one sentence with epidemiologic context.Firm statistics would strengthen the magnitude of the public health burden. For example, “most common” could be supplanted with incidence or prevalence, and “... the median survival of GBM patients remains severely limited” could be strengthened with a statistic. Also, it’s not clear if these statements are based on global or national data.

Results

Figure 1: Human Studies + Mouse Experiments
+ Three individual CSF tracers were used to measure CSF clearance rates (gadobutrol, FITC-dextran, and ovalbumin-AF647).
+ The reduced CSF clearance data was observed even after tumor resection, suggesting a structural change (and not the tumor) may be the contributing factor.
+ The degree of fibrosis was negatively correlated with CSF clearance rates in their mouse models.
+ The treatment with an anti-fibrotic drug (i.e. pirfenidone) improved CSF clearance rates.
- Line 121: The bulk RNA-seq data reflects transcriptomic changes, and does not provide direct genetic variation or mutational evidence.

Figure 2: Human Studies
+ Pathological fibroblasts (CTHRC1$^+$and SMA$^+$) were higher in GBM patients.
+ Single-cell RNA-seq demonstrated higher expression of fibrotic markers and fibrosis-related pathways.
+ GBM-derived CSF induced a myofibroblast phenotype and pathological changes driven by reprogramming molecules (i.e. secreted and dysregulated proteins).

Figure 3: Human Studies + Mouse Experiments
+ The drug inhibition, siRNA knockdown, and conditional knockout of the NF-κB subunit P65NF-κB experiments supported the pathway induction.
+ The NF-κB subunit P65 knockout in fibroblasts did not affect CSF clearance in healthy mice, but CSF clearance improved in the conditional knockout mice.

Figures 4 + 5 + 6: Mouse Experiments
+ The fibroblast-specific P65 conditional knockout mice had reduced tumor burden and prolonged survival.
+ The above changes were associated with increased T cell infiltration and activation in the tumor microenvironment.
+ The replacement model recapitulated that gCSF caused reduced CSF clearance, exhibited faster tumor progression, and lower survival.
+ Reduced CSF clearance induces CD8$^+$ T cell exhaustion.
+ The anti-PD-1 and P65 inhibitor combination treatment dramatically reduced tumor size and prolonged survival.

Figure 6: Human Studies
+ CD8$^+$ T cell exhaustion was positively correlated with collagen-related pathways.

Discussion
+ The non-tumor leptomeninges come from epilepsy patients. While the authors acknowledge the inflammatory features of epilepsy, a brief discussion of how this might bias fibrosis or immune signatures should be added.

Methods
+
The fibroblast-specific P65 conditional knockout mice experiment was particularly strong.

Version 1:

Reviewer comments:

Reviewer #1

(Remarks to the Author)
I appreciate the authors'efforts to revise the manuscript. Several of my previous concerns have been addressed in a constructive manner. In particular, the authors now more clearly acknowledge that their tracer-based assays do not isolate a single CSF clearance route, and the revised interpretation of the post-resection data is more appropriate. The added

clarification regarding tumor location and the distinction between local PC-MRI velocity metrics and global CSF clearance also improves the manuscript.
However, one key concern remains only partially addressed: the mechanistic interpretation of the apparent dissociation between increased ventricular CSF velocity and reduced clearance.
The authors'explanation that PC-MRI-derived velocity reflects local, plane-specific pulsatile motion rather than net CSF turnover is conceptually reasonable. However, the proposed mechanisms, including inefficient oscillatory flow, increased downstream outflow resistance, altered craniospinal compliance, and possible ICP-related changes, remain hypotheses rather than validated mechanisms. The current revision does not provide direct experimental or quantitative physiological evidence to support these interpretations.
The added ventricular size analysis is useful but insufficient to validate an ICP-related or obstructive high-pressure mechanism. A mild increase in third-ventricle width, in the absence of significant changes in the Huckman or Evans indices and without direct ICP measurements, does not establish CSF accumulation, hydrocephalus, elevated ICP, or ineffective oscillatory flow.
Therefore, I recommend that the authors either provide additional evidence supporting this interpretation or further temper the mechanistic language. Useful supporting analyses could include direct ICP measurements, quantitative PC-MRI parameters such as net flow, stroke volume, forward/reverse flow volumes, pulsatility index, or regurgitant fraction, or correlation analyses linking ventricular CSF velocity with tracer-based clearance, ventricular indices, tumor location, tumor volume, or ventricular proximity.
If such experiments or analyses are beyond the scope of the current study, the manuscript should explicitly state that the velocity-clearance dissociation is an observed association, and that ineffective oscillatory flow, altered compliance, increased outflow resistance, and ICP-related changes remain speculative explanations requiring future validation.
Overall, the revised manuscript is improved, but this mechanistic issue remains incompletely resolved. I would consider this point only partially addressed unless the authors either provide additional supporting evidence or substantially weaken the related mechanistic claims.

Reviewer #2

(Remarks to the Author)
The authors have thoroughly addressed my concerns, now including acknowledging limitations of the study (e.g., generalizability, tumor subtypes), adding functional data for proteomics to demonstrate causality (PDGFR inhibition), including controls for fibroblast specificity, discussing potential clinical confounders, and clarifying statistical analyses.

Reviewer #3

(Remarks to the Author)
I appreciate the effort the authors put into revising their manuscript. The mechanistic links are now much stronger, and I am happy to recommend it for publication, provided that the additional comments, limited to the scope of this revision, are addressed.

I have several additional comments:

1. All newly generated datasets must be deposited in public repositories, such as NCBI GEO, and released by the publication date at the latest, without restrictions, as is standard in the field. This includes single-cell transcriptomics in human and mouse, bulk RNA-seq, proteomics, etc.

2. Human analyses are still quite correlative and minimalistic. These results should be interpreted with extreme caution, especially given that the main models used do not faithfully recapitulate that of human GBM, and only limited validation with less immunogenic lines was provided during the revision. These should be outlined in the study limitations section. Similarly, for the sex-specific findings, they used only male mice.

3. Bioluminescent measurements, quantified here as radiance, are not directly representative of tumor size per se but rather of tumor burden.

4. The current gating strategy seems to overestimate the CD45 infiltrate as well as the other immune fractions. Data has to be reanalyzed and replotted. Moreover, PD1 alone is not a marker of exhaustion. Intracellular TOX staining on top of PD1 would be more suitable.

Open Access This Peer Review File is licensed under a Creative Commons Attribution 4.0 International License, which permits use, sharing, adaptation, distribution and reproduction in any medium or format, as long as you give appropriate credit to the original author(s) and the source, provide a link to the Creative Commons license, and indicate if changes were made.
In cases where reviewers are anonymous, credit should be given to 'Anonymous Referee' and the source.
The images or other third party material in this Peer Review File are included in the article's Creative Commons license, unless indicated otherwise in a credit line to the material. If material is not included in the article's Creative Commons license and your intended use is not permitted by statutory regulation or exceeds the permitted use, you will need to obtain permission directly from the copyright holder.
To view a copy of this license, visit https://creativecommons.org/licenses/by/4.0/

Response to Reviewers

1. Summary of major comments from Reviewers.

Based on the comments from the reviewers, we have performed additional experiments and analyses. The details of our revision are summarized in the following **Table 1**.

Table 1. Revision for major comments from the reviewers

Key Questions	Reviewers	New experiments	New supporting data
What CSF clearance route is measured by the current assays?	#1	Added assay-specific clarification and cross-validation, together with integrated interpretation of the tracer-based CSF readouts.	We now clarify that these assays report impaired CSF clearance relevant to leptomeningeal perivascular dysfunction. **(Response Fig. 1a-g; revised Fig.1g-n)** Overinterpretation of route specificity has been removed.
What is the cellular origin of the fibrosis, and is it truly leptomeningeal perivascular?	#2, #3	Added lineage-tracing analyses and a Col1a2-specific conditional model.	New data support $Col1a2^{+}$ perivascular fibroblasts as major contributors to leptomeningeal perivascular fibrosis **(Response Fig. 10a-c, 13a-c; revised Fig.2a-c)** Terminology was revised for greater anatomical precision.
Is the fibroblast-targeting strategy sufficiently specific?	#2, #3	Added lineage-tracing and co-staining analyses and validated key findings using a Col1a2-Cre conditional strategy.	These data reduce concern regarding off-target recombination in the original Tcf21-Cre approach and support fibroblast-specific involvement in the phenotype **(Response Fig. 10a-e, 19a-e; revised Fig.2a-c; Extended Data Fig. 3f)**
Are the mechanistic claims overstated?	#1, #2, #4	Revised the Results and Discussion to remove linear causal overstatement.	The phrase "leptomeninges-CSF-immunosuppression axis" has been removed. The manuscript now states that improved CSF clearance is associated with altered $CD8^{+}$ T-cell accumulation and activation-related changes **(revised Results and Discussion)**
How should the human MRI findings be interpreted?	#1, #2	Reframed the MRI analysis to distinguish local flow-related signals from net clearance efficiency.	We now avoid equating increased flow velocity with improved clearance and discuss possible compensatory ventricular changes more cautiously **(revised Results and Discussion)**

Do the CSF proteomics data identify causal drivers?	#2, #3	Revised the proteomics interpretation and clarified the GBM-associated CSF experimental framework.	PDGF-related ligands, galectins, and related factors are now described as candidate mediators rather than validated drivers **(Response Fig. 11, 14; revised Discussion)**
What is the immune mechanism for impact on CD8 T cells?	#3	We incorporated gene-expression analyses of multiple immune-cell populations and performed in vivo intravascular CD45 labelling with Ki67 staining to assess $CD8^+$ T-cell localization and proliferation.	Increased extravascular (tissue) $CD8^+$ T cells and a higher $Ki67^+$ fraction in CSF-clearance-improved mice, supporting enhanced infiltration and local proliferation. **(Response Fig. 21, Extended Data Fig. 4e-f**)
Are the findings generalizable beyond one GBM model?	#2, #4	Repeated key analyses in the less immunogenic SB28 model.	SB28 tumors showed consistent leptomeningeal PVS fibrosis and CSF-clearance changes, supporting broader model relevance **(Response Fig. 17a-b)**
Are the single-cell analyses and fibroblast-state annotations sufficiently rigorous?	#3	Revised scRNA-seq reporting and redefined fibroblast-state terminology.	Single-cell RNA sequencing of GBM patient leptomeninges revealed a fibrogenic trajectory in fibroblasts, with enhanced collagen synthesis programs distinguishing disease-associated activation. **(Response Fig. 18; revised Fig.2a-h; Extended Data Fig. 2a-c)**
Could human clinical confounders affect interpretation?	#2, #4	Expanded analysis and discussion of subtype heterogeneity and glucocorticoid exposure.	The revised manuscript explicitly discusses these variables as potential confounders and limits translational claims accordingly **(revised Discussion)**
Can statistical rigor and transparency be improved?	#2	Added detailed response tables for major quantitative datasets.	Exact n, means, effect sizes, and multiplicity-adjusted P values are now reported for representative figure families **(Response Tables 2-4; Source Data)**

2. REVIEWER COMMENTS

Reviewer #1 (Remarks to the Author):
This study investigates the impact of GBM on cerebrospinal fluid (CSF) dynamics and clearance, and proposes that leptomeningeal fibroblasts, via NF-κB/P65 signaling, can modulate the immune microenvironment. This suggests a potential axis of "leptomeninges - CSF - immunosuppression." Overall, the topic is of potential significance, but there are several gaps in the evidence chain, and some key conclusions lack direct support. Below, I outline the major and minor concerns.

Response: We sincerely thank Reviewer for the time and care devoted to reviewing our manuscript. Your comments were specific and technically helpful, and they prompted us to re-examine several parts of the study more critically. In response, we have gone point-by-point to (i) tighten the logic and ensure that each conclusion is directly supported by the data, (ii) revise wording where our original text could be read as over-claiming, and (iii) add the additional experiments you suggested where feasible. We believe these changes have made the manuscript more rigorous, clearer in its interpretation, and stronger overall.

1.Evidence for the conclusion that "CSF clearance impairment is independent of tumor mass effect and associated with non-tumoral regions" The authors report that CSF clearance impairment persists in mice even after surgical tumor resection, and based on this observation, they propose that GBM pathology disrupts leptomeningeal-mediated CSF drainage in non-tumoral regions independently of tumor mass effect. However, several concerns arise:
1)Methodology does not specifically indicate which CSF pathway is affected.
The currently recognized CSF clearance pathways include arachnoid villi absorption, meningeal lymphatics, perineural routes, glymphatic pathways, and drainage to deep cervical lymph nodes. The authors measured CSF clearance by injecting gadolinium into the lateral ventricles and sampling from the cisterna magna, which mainly reflects classical ventricular–subarachnoid flow. GBM, especially when not compressing the ventricular system, primarily affects glymphatic and leptomeningeal outflow pathways. Attributing the observed impairment to leptomeningeal or glymphatic dysfunction lacks methodological justification. The authors need to clarify:

a. Which specific CSF clearance pathway does the reported "CSF clearance impairment" correspond to?

Response: We appreciate the reviewer's point and agree that CSF clearance occurs through multiple parallel and interconnected routes (arachnoid granulations, meningeal lymphatics to dCLNs, perineural pathways, and glymphatic/perivascular transport), and that our original wording could be interpreted as implying pathway specificity that is not supported by a single ventriculo-cisternal readout.

In this manuscript, we use "CSF clearance impairment" as an umbrella term for slower CSF-space turnover and reduced brain-to-CSF tracer transport, as captured by our tracer-based assays. Specifically, lateral-ventricle gadolinium injection with cisterna magna sampling primarily reports ventriculo-cisternal transit/CSF-compartment kinetics, and does not allow us to distinguish the

relative contribution of downstream terminal efflux routes. **We have revised the text throughout to avoid over-attribution.** To better localize the affected steps along the CSF-brain exchange continuum, **we added complementary experiments that probe upstream transport processes preceding terminal drainage**:

1) Parenchyma-to-CSF efflux: following intraparenchymal tracer delivery in non-tumoral cortex, GBM mice showed reduced tracer recovery in cisterna magna CSF at 4 h (Response Fig. 1a), indicating impaired solute efflux from brain parenchyma into CSF.
2) Leptomeningeal perivascular transport: using in vivo two-photon imaging, we quantified longitudinal tracer displacement along leptomeningeal perivascular spaces, and observed significantly slower net displacement in GBM mice (Response Fig. 1b), consistent with impaired perivascular transport at the brain surface.
3) Downstream drainage: tracer signal in dCLNs was reduced (Response Fig. 1c). As dCLN accumulation integrates multiple routes, we interpret this as reduced net downstream drainage rather than a meningeal-lymphatic-exclusive defect.
4) Mechanistic reversibility: genetic/pharmacologic targeting of leptomeningeal fibroblast NF-κB/P65 reduced PVS fibrosis and concordantly improved these transport-related readouts (Response Fig. 1d-g), supporting a causal link between leptomeningeal fibrosis and impaired brain-to-CSF turnover.

Collectively, our revised conclusion is that GBM is associated with impaired CSF-space turnover and impaired parenchyma-to-CSF efflux with slowed leptomeningeal perivascular transport, consistent with **dysfunction along the leptomeningeal-perivascular (glymphatic-associated) continuum**.

Page 2, Lines 46-52 (**Results**): In this study, we first used the clinical contrast agent gadobutrol as a tracer to assess an overall CSF tracer clearance readout in mice after intracerebroventricular injection, by quantifying its relative abundance in cisterna magna CSF 3 h later (Fig. 1a). Relative to sham animals, tumor-bearing GBM mice exhibited a markedly reduced gadobutrol clearance-associated readout (from 84.67% to 31.47%, $P < 0.0001$) (Fig. 1a), consistent with prior studies. Notably, this abnormality persisted after surgical tumor resection (Fig. 1a and Extended Data Fig. 1a), suggesting that the defect is not solely attributable to acute tumor mass effect.

Page 3, Lines 75-88 (**Results**):We next asked whether these structural changes were accompanied by functional abnormalities in brain fluid and solute drainage. To do so, we used a set of complementary assays designed to interrogate distinct but related steps of transport. Following intraparenchymal tracer injection, tracer recovery in cisterna magna CSF at 4 h, used here as a surrogate readout of parenchyma-to-CSF efflux, was significantly reduced in GBM mice (Extended Data Fig. 1b). Intravital two-photon imaging further revealed slower tracer movement within leptomeningeal PVS (Fig. 1k), consistent with impeded transport along the brain-surface perivascular compartment. Finally, after intraparenchymal injection of ovalbumin-AF647, GBM mice showed reduced tracer coverage in cervical lymph nodes compared with sham controls ($P = 0.0083$; Fig. 1l and Extended Data Fig. 1c), consistent with impaired downstream drainage. To functionally test whether fibrosis contributes to this phenotype, we administered the anti-fibrotic drug pirfenidone into the CSF of tumor-bearing mice via osmotic pump. Pirfenidone reduced leptomeningeal fibrosis (Fig. 1m) and

significantly improved the parenchyma-to-CSF tracer efflux readout (Fig. 1n). Together, these data support leptomeningeal perivascular fibrosis as an important contributor to GBM-associated impairment of CSF transport and clearance.

Page 9, Lines 363-366 (**Discussion**): Accordingly, impaired CSF clearance in GBM is likely multifactorial. While our data support leptomeningeal fibrosis as an important gatekeeping mechanism that can restrict CSF outflow, they do not exclude additional contributors such as altered vascular pulsatility, meningeal lymphatic dysfunction or broader CSF hydrodynamic disturbances.

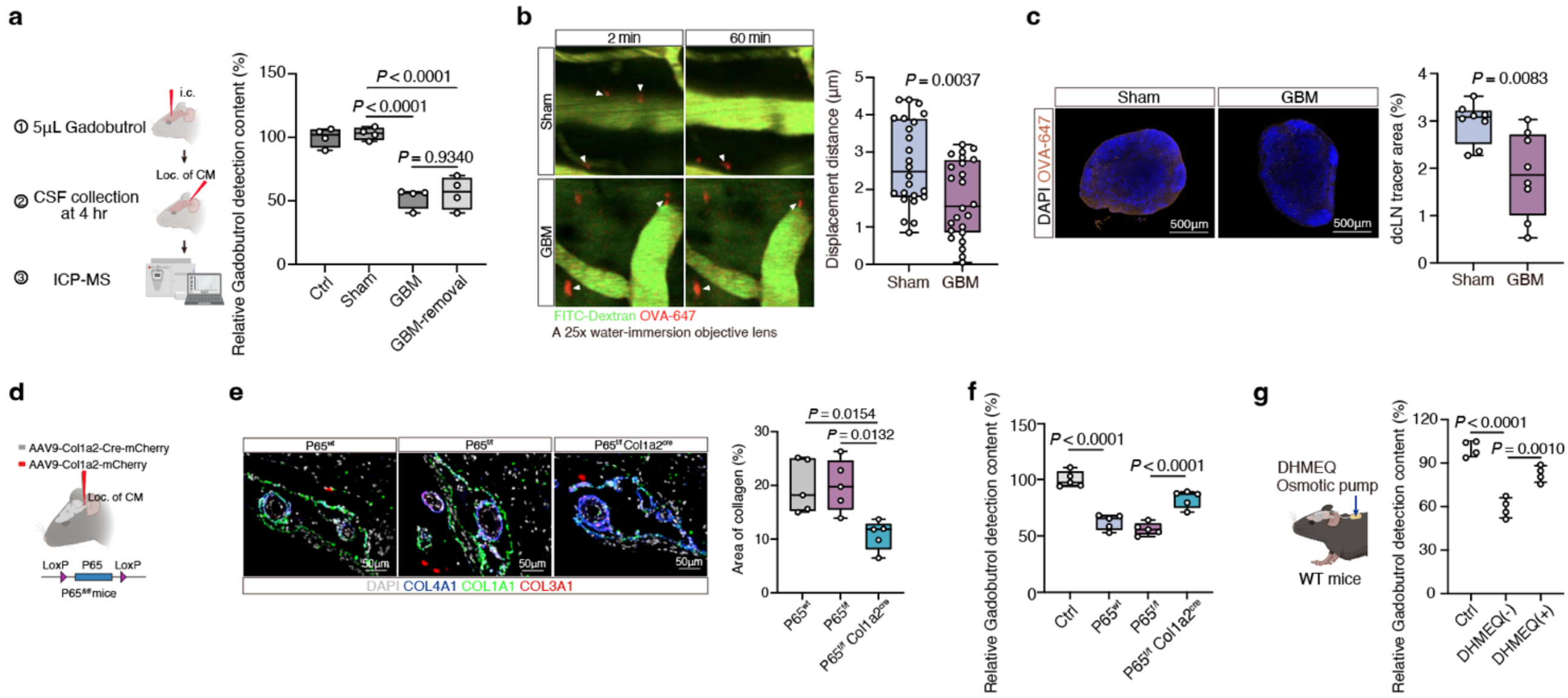

Response Figure. 1 | a, Left, Schematic of the parenchyma-to-CSF efflux assay: intraparenchymal injection of the gadobutrol tracer followed by cisterna magna CSF collection for ICP-MS quantification. Right, Quantification of gadobutrol levels in cisterna magna CSF (mean ± s.d.; n = 4 mice per group). **b,** Left, Representative in vivo two-photon images of leptomeningeal perivascular spaces (PVS) in sham and GBM tumor-bearing mice following intraparenchymal injection of the fluorescent tracer ovalbumin-AF647. Right, Quantification of CSF tracer displacement distance along leptomeningeal PVS (mean ± s.d.; n = 24 vessels per group from 3 mice per group). **c,** Left, Representative fluorescence images of dCLNs from sham and GBM tumor-bearing mice after intraparenchymal injection of ovalbumin-AF647. Scale bar, 500 μm. Right, Quantification of fluorescent coverage area in dCLNs (mean ± s.d.; n = 8 mice per group). **d,** Workflow for generating a conditional P65 knockout mouse model in leptomeningeal fibroblasts. **e,** Left, representative images of type I/III collagen fibres in the leptomeninges of GBM tumour-bearing mice in the indicated groups. Scale bar, 50 μm. Right, quantification of type I/III collagen fibre area in the leptomeninges (mean ± s.d., n = 5 mice per group). **f,** Relative gadobutrol content detected in CSF from GBM tumour-bearing mice of the indicated genotypes (mean ± s.d., n = 5 mice per group). **g,** Quantification of relative gadobutrol content in CT2A-Luc tumour-bearing mice treated with vehicle or DHMEQ (mean ± s.d., n = 4 per group).

b.Can the current experimental method distinguish between ventricular pathway, leptomeningeal pathway, and glymphatic pathway?

Response: We appreciate this important methodological question. **We agree that our current approaches cannot unambiguously disentangle the ventricular-subarachnoid CSF transit, leptomeningeal outflow, and glymphatic/perivascular transport as fully separable**, independently quantifiable pathways. CSF circulation and solute transport are highly interconnected: parenchymal CSF-ISF exchange (glymphatic transport) interfaces with perivascular spaces at the brain surface and the meningeal CSF compartment, and downstream drainage can converge toward meningeal lymphatics and dCLNs. Therefore, tracer-kinetic readouts alone are inherently limited in assigning an observed deficit to a single exclusive anatomical route.

To calibrate the scope of inference, we now explicitly state in the revised manuscript what our data do and do not demonstrate. **Our experiments support that GBM is associated with slower CSF/solute transport kinetics, including reduced parenchyma-to-CSF efflux and slower tracer movement along leptomeningeal surface perivascular spaces**, and that these abnormalities are mechanistically supported by leptomeningeal fibroblast-driven fibrosis, given the concordant and reversible improvement with fibroblast-targeted genetic and pharmacological interventions. However, with the present methods we cannot provide an exclusive quantitative partitioning among terminal outflow routes (arachnoid granulations vs meningeal lymphatics vs perineural pathways). Accordingly, we describe **our findings as implicating the leptomeningeal perivascular segment of the clearance/transport network** without claiming pathway exclusivity.

c. Why is this method appropriate to infer leptomeningeal clearance impairment?

Response: We appreciate this important question. We agree that no single assay in our study can uniquely and pathway-specifically quantify "leptomeningeal clearance." Accordingly, in the revised manuscript we frame our conclusion more conservatively as **leptomeningeal/surface-perivascular–associated transport or outflow impairment, supported by convergent evidence from complementary readouts**:
1) Functional readout: The parenchyma-to-CSF efflux assay (standardized intraparenchymal tracer injection in non-tumoral cortex followed by early cisterna magna CSF sampling) provides a functional readout of brain-to-CSF solute efflux. Reduced recovery indicates impaired upstream outflow/transport steps toward CSF compartments, while not specifying the final terminal exit route.
2) Spatial readout: In vivo two-photon imaging demonstrates slower tracer transport within leptomeningeal surface PVS, anatomically aligning with the functional efflux deficit and supporting involvement of the surface PVS/leptomeningeal segment rather than being explained solely by a generic change in bulk CSF circulation.
3) Downstream readout: Reduced tracer signal in dCLNs supports decreased net downstream drainage, while we acknowledge that dCLN accumulation integrates multiple upstream routes.
4) Rescue: Targeting leptomeningeal fibroblasts reduces leptomeningeal fibrosis and in parallel improves the efflux and downstream drainage readouts. This coordinated rescue supports a mechanistic link between leptomeningeal fibrosis and impaired surface-associated transport.

Taken together, while we cannot isolate an exclusive pathway, the combined functional, spatial, downstream, and rescue evidence supports our inference of leptomeningeal PVS-associated

transport impairment in the GBM condition. We have revised the text accordingly to avoid over-attribution to a single anatomical route.

Page 9, Lines 363-366 (**Discussion**): Accordingly, impaired CSF clearance in GBM is likely multifactorial. While our data support leptomeningeal fibrosis as an important gatekeeping mechanism that can restrict CSF outflow, they do not exclude additional contributors such as altered vascular pulsatility, meningeal lymphatic dysfunction or broader CSF hydrodynamic disturbances.

2) Persistence of post-resection abnormality does not directly indicate origin from non-tumoral regions. GBM is highly infiltrative, and tumor boundaries are generally indistinct. It is unclear whether peri-tumoral tissues post-resection are free of residual tumor cells. How can the authors exclude the influence of residual tumor cells or secondary pathological changes (e.g., inflammation, microenvironment remodeling) on CSF pathways? More direct evidence is needed to support that the observed abnormality indeed originates from "uninfiltrated normal regions."

Response: We appreciate this important point. We agree that GBM is highly infiltrative and that persistence of an abnormality after resection does not demonstrate that the abnormality originates from truly uninfiltrated normal regions. Residual tumor cells as well as postoperative/secondary processes (inflammation, wound response, and microenvironmental remodeling) could contribute.

Accordingly, in the revised manuscript we have softened any wording implying that post-resection persistence proves a non-tumoral origin. **The post-resection experiment is now interpreted more narrowly as evidence that the clearance abnormality cannot be explained solely by gross tumor mass effect/ventricular compression.**

Regarding "non-tumoral" regions, we define these areas operationally as regions with no radiographic and no routine histopathologic evidence of tumor involvement (e.g., absence of leptomeningeal enhancement on contrast MRI; negative screening on serial sections using standard glioma-associated markers such as Olig2 and GFAP) (Response Fig. 2a-b). We explicitly acknowledge that these criteria do not exclude microscopic infiltration below detection limits, and we have added this limitation to the Discussion.

Overall, our revised conclusion is that the observed clearance impairment persists beyond removal of gross mass effect and is associated with leptomeningeal/perivascular alterations.

Page 2, Lines 46-52 (**Results**): Notably, this abnormality persisted after surgical tumor resection (Fig. 1a and Extended Data Fig. 1a), suggesting that the defect is not solely attributable to acute tumor mass effect.

Page 3, Lines 90-95 (**Results**): We measured ventricular CSF velocity metrics in 15 non-tumor controls, 20 GBM patients (temporal, frontal, or thalamic tumors), and 6 post-operative GBM patients (Fig. 1o and Extended Data Fig. 1d-e). GBM patients exhibited higher CSF flow velocities in the third ($P = 0.0095$) and fourth ventricles ($P = 0.0058$), with no significant change in the lateral

ventricles (Fig. 1o-r). Elevated fourth-ventricle velocities persisted after resection (P = 0.0248; Fig. 1q).

Page 11, Lines 434-436 (**Discussion**): ...some baseline immune or extracellular matrix alterations in these leptomeningeal samples cannot be completely excluded, although neoplastic disease was excluded radiographically and tumor infiltration was excluded pathologically.

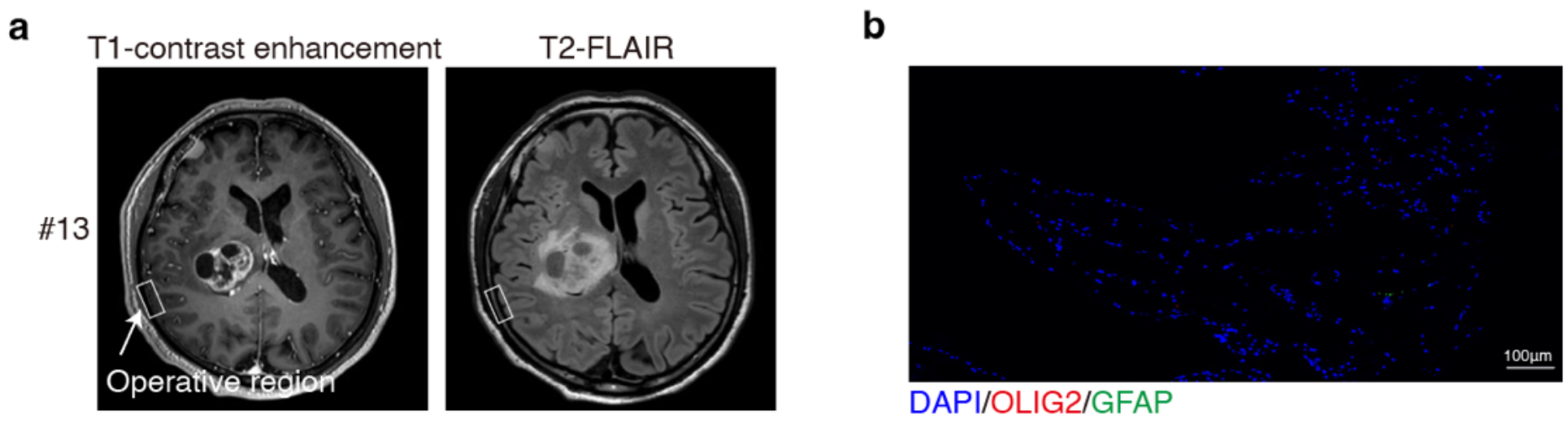

Response Figure. 2 | a, Representative T1-weighted contrast-enhanced and T2-FLAIR MRI images from a glioma patient. **b,** Representative immunofluorescence staining for Olig2 and cytoplasmic GFAP. Scale bar, 100 μm.

3)Does leptomeningeal structural alteration depend on tumor location?
The authors propose that GBM disrupts leptomeningeal drainage structures but do not address whether this effect depends on tumor location. For superficial cortical tumors, direct perturbation of the leptomeningeal structures and pial perivascular spaces is plausible. However, for deep-seated tumors (e.g., thalamus or basal ganglia), it is unclear whether similar leptomeningeal remodeling, ECM deposition, or fibroblast activation can occur. The authors are encouraged to clarify:
a.Does tumor location influence the proposed mechanism of leptomeningeal structural damage?

Response: We thank the reviewer for raising this important point. We agree that for superficially located cortical tumors, direct perturbation of the leptomeningeal perivascular spaces via local mechanical distortion or focal inflammatory responses is plausible. **Based on our current data, our interpretation is that leptomeningeal remodeling can occur even without direct tumor-leptomeningeal contact, and therefore may not be restricted to superficial tumors**. We nonetheless acknowledge that tumor location could modulate the magnitude and spatial distribution of these changes, which will require systematic stratified comparisons.

In our animal experiments, we used an orthotopic GBM implantation model in the deep caudate nucleus (Response Fig. 3a), which is not adjacent to the cortical surface. Despite the absence of direct tumor-meningeal contact, we observed leptomeningeal/surface-PVS abnormalities, including fibrosis and increased collagen/ECM deposition (Response Fig. 3b), which were concordant with reduced CSF/solute transport readouts in our clearance assays (Response Fig. 3c). These findings support that deep-seated tumors can be sufficient to elicit leptomeningeal-associated remodeling at a distance, at least in this model.

In our clinical cohort, we included primary GBM cases from different anatomical locations and did not observe imaging or intraoperative evidence of overt leptomeningeal invasion/dissemination

(Response Fig. 3d-e). Leptomeningeal-associated regions showed ECM deposition and structural features qualitatively consistent with the mouse findings (Response Fig. 3f). While not powered to formally test location-dependence, these observations are consistent with the notion that leptomeningeal alterations are not exclusive to superficial cortical tumors.

We have added to the Discussion that future studies should compare superficial versus deep tumor models (e.g., stratified by tumor-to-surface distance and tumor burden) to quantitatively assess potential location dependence.

Page 11, Lines 442-446 (**Discussion**): Third, the mechanism described here is supported in a deep-seated orthotopic model, but tumor location may modulate the extent and pattern of leptomeningeal/PVS remodeling and CSF clearance impairment. Future studies should systematically compare superficial versus deep tumor models, stratified by tumor-to-cortical-surface distance and tumor burden.

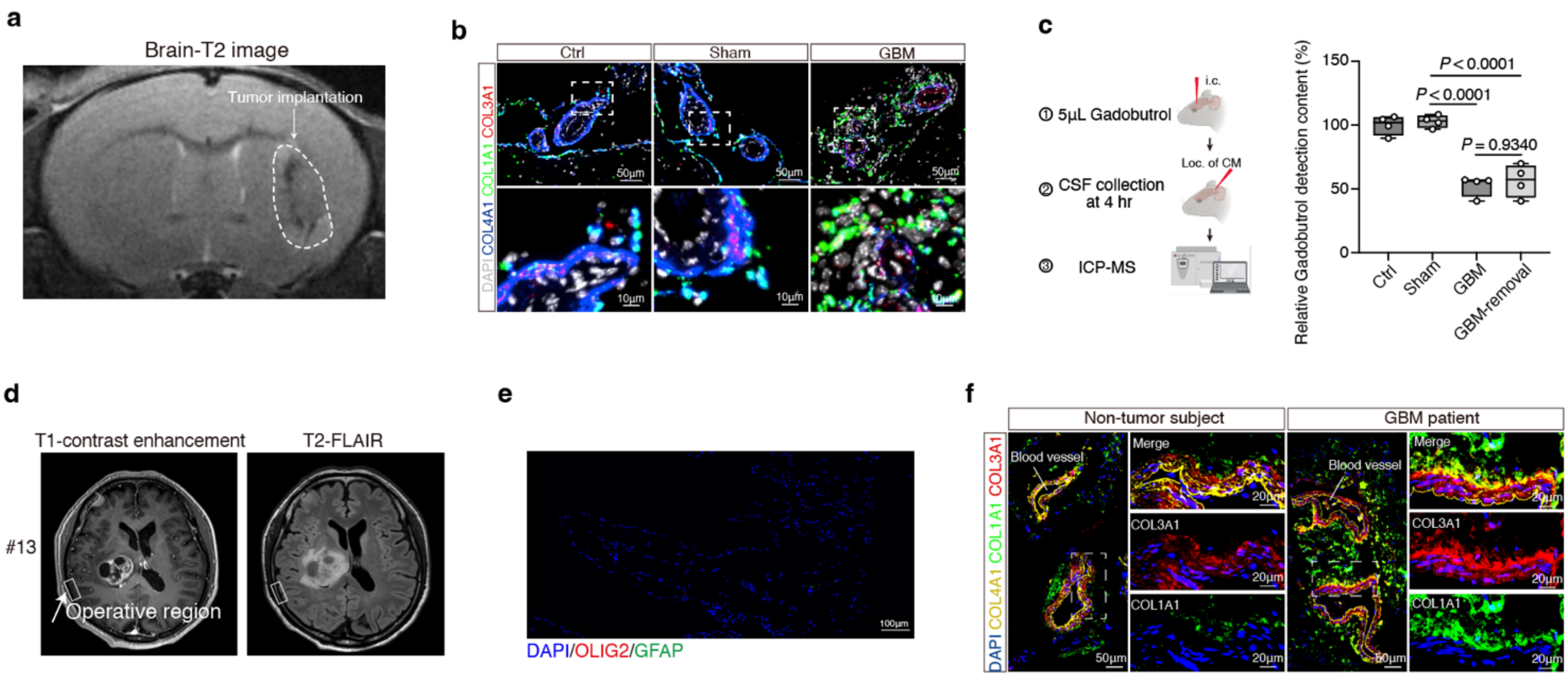

Response Figure. 3 | a, Representative T2-weighted MRI images from GBM-bearing mice. **b,** Representative images of perivascular spaces (COL4A1^{+}) and type I/III collagen fibres (COL1A1^{+}/COL3A1^{+}) in the leptomeninges of control mice, sham-operated mice and GBM tumour-bearing mice. Scale bar, 50 µm; zoom-in, 10 µm. **c,** Left, Schematic of the parenchyma-to-CSF efflux assay: intraparenchymal injection of the gadobutrol tracer followed by cisterna magna CSF collection for ICP-MS quantification. Right, Quantification of gadobutrol levels in cisterna magna CSF (mean ± s.d.; n = 4 mice per group). **d,** Representative T1-weighted contrast-enhanced and T2-FLAIR MRI images from a glioma patient. **e,** Representative immunofluorescence staining for Olig2 and cytoplasmic GFAP. Scale bar, 100 µm. **f,** Representative multiplex immunofluorescence images of perivascular spaces in transverse leptomeningeal sections from non-tumour controls and patients with GBM. Scale bar, 50 µm; zoom-in, 20 µm.

b.If deep-seated tumors can also induce leptomeningeal changes, what are the potential biological pathways (e.g., diffusion of inflammatory factors, CSF-mediated exosome transport, PVS–leptomeningeal continuity remodeling)?

Response: We thank the reviewer for this insightful question. We agree that if deep-seated GBM induces leptomeningeal alterations, the relevant signals likely act at a distance rather than through direct physical contact. **We consider several plausible, non-mutually exclusive mechanisms:**

1) CSF/ISF-borne inflammatory mediators. Inflammatory cytokines/chemokines are reported to be elevated in the CSF of glioma patients (e.g., IL-6, CCL2 and related CXCL chemokines), consistent with tumor-associated inflammation[1,2]. Such soluble mediators could distribute via ISF–CSF exchange and CSF circulation to meningeal compartments, where they may activate leptomeningeal fibroblasts and promote ECM remodeling/fibrosis.

2) CSF compositional changes observed in our dataset. Our CSF proteomics in tumor-bearing mice revealed significant shifts in CSF protein composition, including factors linked to ECM organization and fibroblast activation/fibrotic programs (e.g., PDGFC, PDGFD, LGALS9; Fig. 2k). **To provide functional evidence for PDGF pathway involvement, we performed additional perturbation experiments targeting PDGFRβ in leptomeningeal fibroblasts.** PDGFRβ inhibition significantly attenuated the gCSF-induced profibrotic activation phenotype, reducing the αSMA/Col1a1 double-positive population from 52.2517 to 25.4867 (approximately 51.2% reduction) (Response Figure. 4a-b). These data support that PDGFRβ signaling contributes, at least in part, to the CSF-driven phenotypic switch.

3) CSF/PVS-mediated transport of tumor-derived extracellular vesicles. Tumor-derived extracellular vesicles (including exosomes) and other CSF/PVS-borne signals could reach the cortical surface/meninges and modulate leptomeningeal cell states. In addition, because perivascular spaces form a continuous network connecting deep parenchyma to surface compartments, tumor-driven changes in PVS composition and mechanics could propagate toward leptomeningeal segments.

We have revised the Discussion to present these as data-supported, testable hypotheses.

Page 9-10, Lines 384-389 (**Discussion**): Nevertheless, although PDGFC, PDGFD and LGALS9 are enriched in gCSF and PDGFRβ is expressed on perivascular fibroblasts, these data remain correlative. Establishing a causal PDGF-PDGFRβ- or LGALS9-dependent mechanism will require targeted perturbation studies. More broadly, the CSF-induced fibroblast phenotype is likely driven by the synergy of multiple CSF-borne factors rather than by a single dominant mediator.

Page 10, Lines 392-394 (**Discussion**):…While our current study highlights the central role of secretory proteins, the contribution of other CSF metabolites to fibroblast activation and leptomeningeal remodeling warrants further investigation.

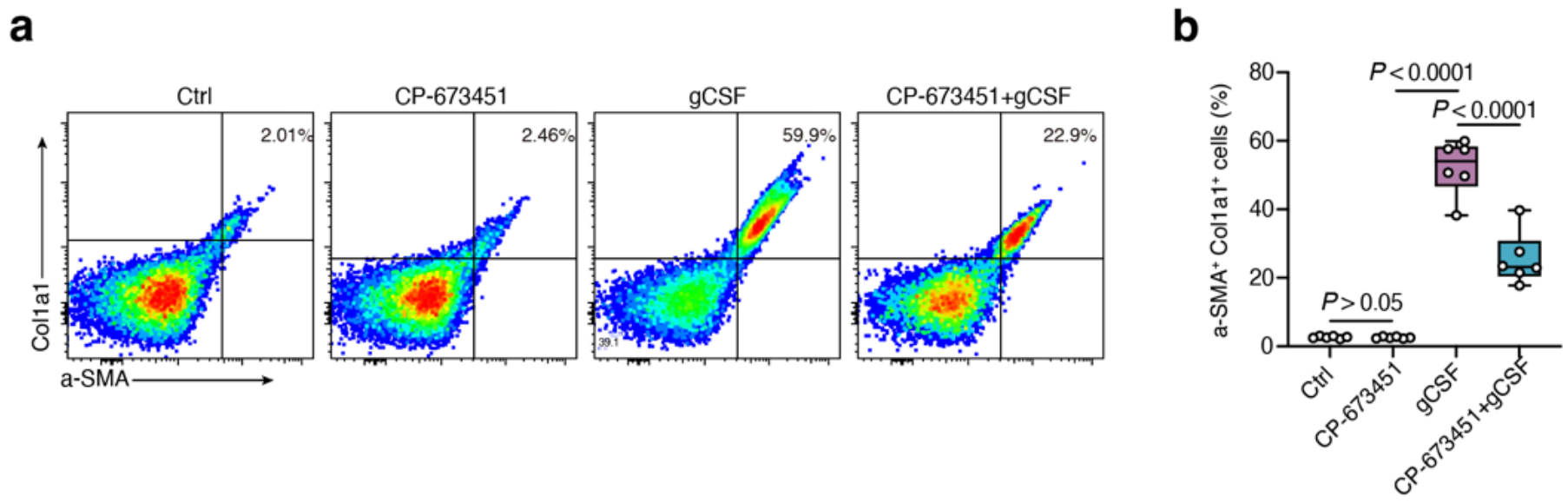

Response Figure. 4 | a, Representative flow cytometry plots showing αSMA and Col1a1 staining in leptomeningeal fibroblasts treated with control medium (Ctrl), CP-673451 (200 nM), GBM-derived CSF (gCSF), or gCSF+CP-673451. **b,** Quantification of $\alpha SMA^{+}Col1a1^{+}$ cells (mean ± s.d., n = 6).

Reference:

1. Koper, O. et al. Cerebrospinal fluid and serum IL-8, CCL2, and ICAM-1 concentrations in astrocytic brain tumor patients. Irish Journal of Medical Science (1971-) 187, 767-775 (2018).

2. Kim, J. W., Wu, S.-Y., Tyagi, A. & Watabe, K. The immune landscape of cerebrospinal fluid across brain malignancies. Neuro-Oncology Advances, vdag058 (2026).

c.Provide MRI images showing tumor implantation sites to clarify spatial relationships between tumor foci and leptomeninges, supporting inferences about leptomeningeal drainage impairment.

Response: We thank the reviewer for this important suggestion. We agree that MRI documentation of tumor location is essential to clarify the spatial relationship between tumor foci and the leptomeningeal compartment and to aid interpretation of leptomeningeal structural changes and CSF drainage alterations.

Accordingly, **we have added representative MRI images from both human patients and our mouse GBM model** (Response Fig. 5a). In the human cohort, we now include representative post-gadolinium T1-weighted and T2-FLAIR images illustrating lesion location (Response Fig. 5b). These sequences were also used to exclude cases with MRI features suggestive of overt leptomeningeal disease (e.g., leptomeningeal contrast enhancement; sulcal FLAIR signal changes in conjunction with enhancement).

In the mouse model, we include representative T2-weighted MRI demonstrating that tumors are localized to deep parenchymal regions (e.g., striatum) and remain spatially separated from the cortical surface/leptomeninges.

We emphasize that MRI is used here as an anatomical localization and exclusion tool rather than a functional readout of CSF dynamics. Restricting analyses to subjects without MRI evidence of overt leptomeningeal involvement strengthens the interpretation that the observed leptomeningeal remodeling and CSF clearance impairment are not simply attributable to direct leptomeningeal invasion detectable on MRI. We have added the corresponding description to the Methods.

Page 11, Lines 470-473 (**Methods**): Preoperative brain MRI was reviewed for all human GBM cases, including T1-weighted images before and after gadolinium administration and T2-FLAIR sequences, to document tumor location and to exclude radiological evidence of leptomeningeal dissemination (defined as leptomeningeal contrast enhancement or sulcal FLAIR hyperintensity).

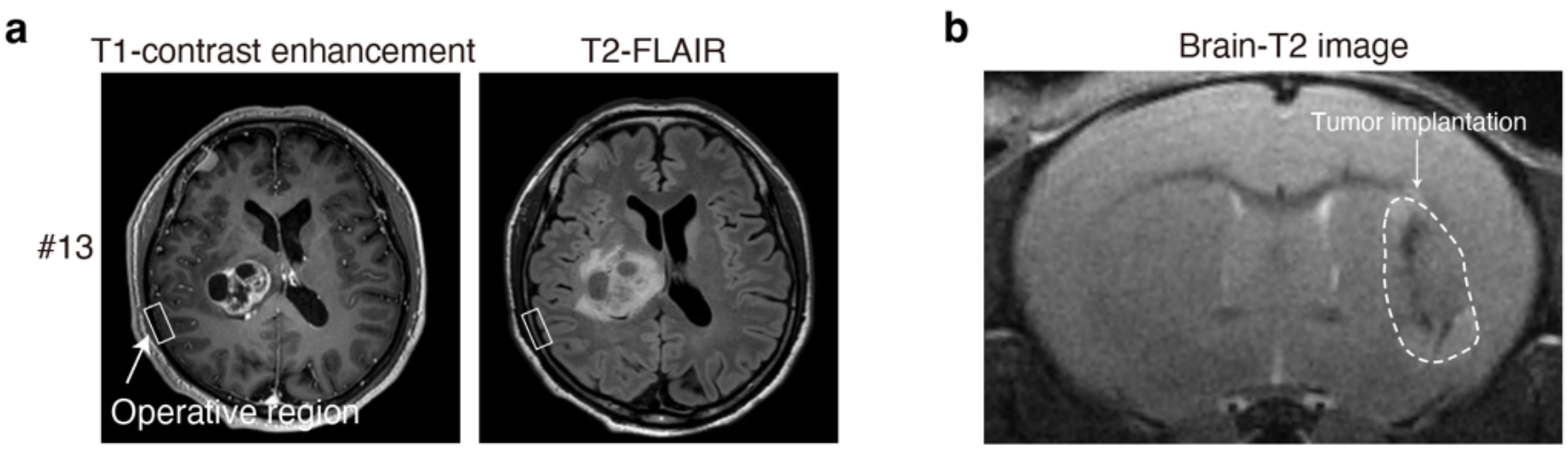

Response Figure. 5 | a, Representative T1-weighted contrast-enhanced and T2-FLAIR MRI images from a glioma patient. **b,** Representative T2-weighted MRI images from GBM-bearing mice.

d. If all models involve cortical/superficial implantation, the discussion should specify whether this mechanism is generalizable to deep-seated tumors.

Response: We thank the reviewer for this important point. **We would like to clarify that our study is not limited to cortical/superficial implantation.** In our mouse GBM model, tumor cells were implanted into a deep parenchymal site (caudate nucleus/striatum; confirmed by MRI), without direct anatomical contact with the leptomeninges. Despite this deep location, we still observed leptomeningeal remodeling together with impaired CSF/solute outflow. **We have revised the Discussion to state that these findings indicate the proposed mechanism is not restricted to superficial tumors**, while also noting that tumor location may modulate the magnitude and spatial pattern of these changes and warrants systematic comparison in future work.

Page 11, Lines 442-446 (**Discussion**): Third, the mechanism described here is supported in a deep-seated orthotopic model, but tumor location may modulate the extent and pattern of leptomeningeal/PVS remodeling and CSF clearance impairment. Future studies should systematically compare superficial versus deep tumor models, stratified by tumor-to-cortical-surface distance and tumor burden.

2. Inadequate explanation of the dissociation between increased CSF flow velocity and reduced clearance

The authors report that GBM patients show significantly elevated CSF flow velocities in the third and fourth ventricles, but not in the lateral ventricles; post-resection, only the fourth ventricle remains elevated. They suggest that CSF flow velocity may be "decoupled" from functional clearance. This raises several questions:

1)Why is only part of the ventricular system affected? Why does lateral ventricular flow remain unchanged while third and fourth ventricles increase? Could this reflect tumor location, ventricular anatomy, imaging angle, or pulsatile flow propagation differences? Moreover, post-resection, lateral

and third ventricles normalize, but fourth ventricle remains elevated. Is this single-ventricle abnormality sufficient to claim "physiological dysfunction"?

Response: We thank the reviewer for the careful attention to the spatial heterogeneity of ventricular CSF velocity changes. We agree that our results reflect region-specific differences in phase-contrast MRI-derived local velocity metrics, rather than a uniform change across the entire ventricular system. In the revised manuscript, **we have toned down the wording and clarified that local CSF velocity measured by PC-MRI is a kinematic metric and does not directly quantify CSF-ISF exchange or solute clearance efficiency.**

1) Why are the third/fourth ventricles increased while the lateral ventricles are unchanged?
We interpret this pattern primarily in the context of ventricular anatomy/flow physics and measurement sensitivity. CSF motion in the lateral ventricles is typically low-velocity and strongly oscillatory with local recirculation; consequently, summary metrics (e.g., peak/mean velocity) can be more sensitive to ROI definition and imaging plane. In contrast, the third and fourth ventricles are midline compartments hydraulically coupled to narrow connecting/outflow segments (e.g., the cerebral aqueduct and fourth-ventricle outlets), where pulsatile driving can manifest as higher detectable peak velocities[1-3]. Importantly, we caution that "no significant change in the lateral ventricles" should not be interpreted as "no involvement," but rather as limited sensitivity of this metric to lateral-ventricle flow complexity. We have added this caveat to the Results.

2) Why does the third ventricle normalize after resection whereas the fourth ventricle remains elevated?
We consider several non-mutually exclusive possibilities. Resection may partially relieve upstream pressure constraints affecting third-ventricle kinematics, whereas fourth-ventricle velocities-being influenced by posterior fossa anatomy, craniospinal compliance, and strong brainstem-driven pulsatility-may remain more sensitive to postoperative changes or downstream outflow resistance[4,5]. We now present this as persistent spatially heterogeneous kinematic dysregulation, rather than evidence for a single global mechanism.

3) Is a single-ventricle abnormality sufficient to claim "physiological dysfunction"?
We agree it is not. We have revised the text to avoid using fourth-ventricle velocity changes alone to infer impaired clearance. Our conclusions regarding clearance impairment are based on independent multimodal clearance readout, while ventricular velocity measurements are presented as supportive observations with explicit interpretational limits.

Page 3, Lines 89-97 (**Results**): To determine whether GBM is also associated with altered regional CSF hydrodynamics in patients, we next assessed ventricular CSF flow by phase-contrast MRI (PC-MRI). We measured ventricular CSF velocity metrics in 15 non-tumor controls, 20 GBM patients (temporal, frontal, or thalamic tumors), and 6 post-operative GBM patients (Fig. 1o and Extended Data Fig. 1d-e). GBM patients exhibited higher CSF flow velocities in the third ($P = 0.0095$) and fourth ventricles ($P = 0.0058$), with no significant change in the lateral ventricles (Fig. 1o-r). Elevated fourth-ventricle velocities persisted after resection ($P = 0.0248$; Fig. 1q). Because PC-MRI primarily reports local kinematics at selected imaging planes rather than whole-system CSF turnover, we

interpret these findings as evidence of regionally altered ventricular CSF dynamics, which may not directly track global CSF clearance.

Reference:
1. Zhu, D. C., Xenos, M., Linninger, A. A. & Penn, R. D. Dynamics of lateral ventricle and cerebrospinal fluid in normal and hydrocephalic brains. Journal of Magnetic Resonance Imaging: An Official Journal of the International Society for Magnetic Resonance in Medicine 24, 756-770 (2006).
2. Sweetman, B. & Linninger, A. A. Cerebrospinal fluid flow dynamics in the central nervous system. Annals of biomedical engineering 39, 484-496 (2011).
3. Wada, T. et al. Visualization of cerebrospinal fluid dynamics using multi-spin echo acquisition cine imaging (MUSACI). Magnetic Resonance in Medicine 81, 331-341 (2019).
4. Tseng, J.-S., Lee, Y.-C., Pan, H.-C. & Chang, M.-H. Motor neuron disease-like syndrome secondary to trapped fourth ventricle and obstruction of cerebrospinal fluid pathway. Clinical neurology and neurosurgery 109, 383-387 (2007).
5. Tyagi, G. et al. Trapped fourth ventricle—treatment options and the role of open posterior fenestration in the surgical management. Acta Neurochirurgica 162, 2441-2449 (2020).

2)Does a single ventricle's flow change reflect overall CSF dynamics? Can the authors demonstrate that flow changes in a single ventricle represent global CSF dysfunction? If not, overinterpretation should be avoided.

Response: We thank the reviewer for this important point. **We fully agree that a flow change measured in a single ventricle by PC-MRI cannot, on its own, demonstrate global CSF dysfunction.**

In the revised manuscript, we explicitly distinguish between (i) local PC-MRI velocity metrics measured at specific ventricular planes, which primarily reflect regional pulsatile hydrodynamics and are influenced by ventricular geometry, compliance, and imaging-plane/ROI selection, and (ii) CSF clearance readouts, which reflect integrated processes including CSF-ISF exchange, perivascular transport, and downstream efflux pathways. Accordingly, we no longer present ventricular velocity as a surrogate for whole-system clearance.

Consistent with the reviewer's concern, **we have tempered our language and revised the Results/Discussion to describe these findings as spatially heterogeneous alterations in ventricular CSF kinematics**, rather than "global dysfunction," and we avoid implying that a single-ventricle abnormality is sufficient to define a system-wide failure.

We appreciate the reviewer's comment, which helped us sharpen the conceptual boundary between local PC-MRI velocity metrics and clearance/outflow and improve the precision of our conclusions.

Page 3, Lines 95-97 (**Results**): Because PC-MRI primarily reports local kinematics at selected imaging planes rather than whole-system CSF turnover, we interpret these findings as evidence of regionally altered ventricular CSF dynamics, which may not directly track global CSF clearance.

3)Mechanistic discussion and validation of the “dissociation” phenomenon:
The observed increase in CSF flow velocity concurrent with decreased clearance is interesting but seemingly contradictory. The discussion lacks mechanistic exploration. For instance, could this reflect “ineffective turbulence” in CSF circulation or obstructive high-pressure in outflow pathways? Based on the findings, increased flow with slowed clearance might lead to CSF accumulation and elevated intracranial pressure (ICP). Do the authors have ventricular enlargement or direct ICP measurements to support this? Speculative discussion and supporting data would substantially strengthen the manuscript.

Response: We thank the reviewer for this insightful comment. **We agree that the concurrent increase in ventricular CSF velocity together with reduced clearance appears counterintuitive and warrants a clearer mechanistic discussion.** Importantly, PC-MRI-derived velocity is a local, plane-specific kinematic metric that predominantly captures pulsatile (oscillatory) motion and does not necessarily reflect effective CSF turnover, net throughput, or solute clearance.

In the revised Discussion, we now outline several non-mutually exclusive mechanisms that could produce this “dissociation.” **We have avoided referring to “turbulence” and instead describe these patterns as inefficient oscillatory flow.**

To address the reviewer’s suggestion regarding CSF accumulation/ICP, we quantified ventricular size on MRI using three established indices (third-ventricle width, Huckman index, and Evans index) (Response Fig. 6). GBM patients showed a mild increase in third-ventricle width ($P = 0.0267$), whereas Huckman and Evans indices were not significantly different from controls ($P > 0.05$ for both) and did not indicate overt hydrocephalus in this cohort. We emphasize that ventricular indices provide anatomical context but do not substitute for direct ICP measurements; accordingly, we have tempered the text and do not equate the velocity-clearance dissociation with elevated ICP.

Overall, we have revised the Discussion to more clearly separate MRI-based kinematic observations from tracer-based clearance readouts and to frame mechanistic interpretations as testable hypotheses.

Page 9, Lines 366-373 (**Discussion**): In addition, GBM may increase downstream outflow resistance and/or alter craniospinal compliance, which could amplify pulsatile, oscillatory (“to-and-fro”) CSF motion and thereby increase PC-MRI peak velocities measured in the third/fourth ventricular pathway. At the same time, increased resistance and reduced effective compliance may diminish the net exchange and efflux required for solute clearance. Under this framework, higher local velocities can coexist with poorer clearance because oscillatory motion increases while net transport efficiency decreases, consistent with greater recirculation rather than effective throughput.

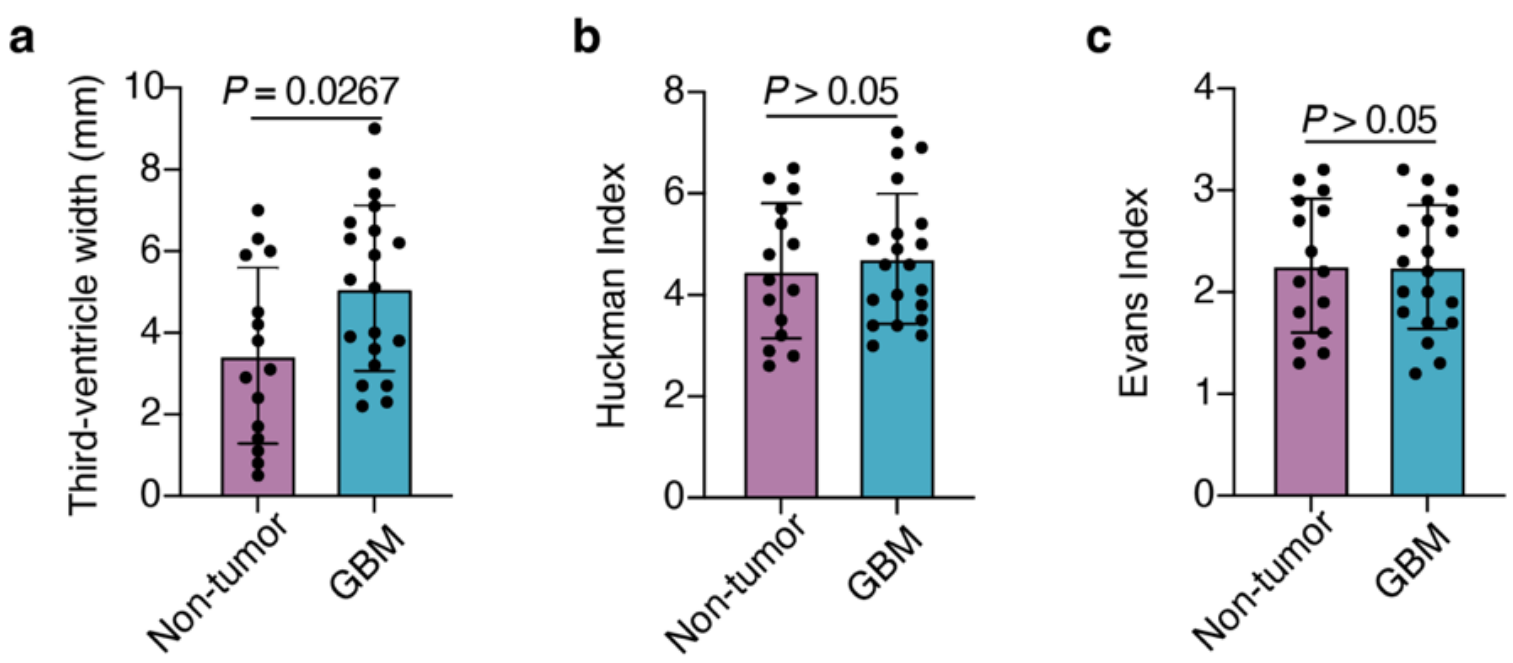

Response Figure. 6 | Ventricular size was quantified on axial MR images using three established indices: **a,** third-ventricle width (maximum transverse diameter of the third ventricle), **b,** Huckman index (sum of the maximal widths of the frontal horns of the lateral ventricles), and **c,** Evans index (ratio of the maximal frontal horn width to the maximal internal skull diameter at the same level). (mean ± s.d.).

3. Abbreviations
1) The abbreviations P65wt, P65f/f, P65f/fTcf21Cre appear in lines 247–249 but should be defined at first occurrence (lines 235–236).
2) CSF (Cerebrospinal Fluid) should be defined at first mention in the text (line 296 is too late).

Response: We thank the reviewer for pointing out these oversights. **The abbreviations have now been defined at their first occurrence in the text.**

4.Consistency and statistical rationale of animal data
1)Incomplete methodological description:
Methods lack specific details for bioluminescence imaging of tumor size (e.g., instrument model, luciferin dose and timing, image analysis). Please provide these details.

Response: We thank the reviewer for pointing out that the description of the bioluminescence imaging (BLI) methodology was incomplete and agree that these details are essential for reproducibility.

In the revised manuscript, **these methodological clarifications have been incorporated into the revised Methods section** to improve transparency and reproducibility.

Page 12, Lines 489-500 (**Methods**): Tumor progression was followed by bioluminescence imaging (BLI) using an AniView100 Multimode Live Animal Imaging System (Boluteng Biological Technology Co., Ltd., Guangzhou, China). D-luciferin sodium (Macklin, D812647) was administered intraperitoneally (150 mg/kg). Images were acquired 14 min after luciferin injection with a 90-s exposure time; the imaging time point was chosen based on pilot testing to capture a stable bioluminescent signal. BLI data were processed with AniView software. Total photon flux (photons/s) was quantified within a manually drawn ROI covering the cranial tumor signal, with background subtraction performed using a standardized ROI placed in a non-tumor area. All animals were imaged using the same acquisition and analysis settings. Photon flux was used as a relative longitudinal

indicator of tumor burden for within-study comparisons, rather than as an absolute volumetric measurement.

2)Data inconsistencies:
In the GL261 model, Extended Data Fig. 4e showed no tumor size difference between P65f/fTcf21Cre + anti-CD8 groups and P65f/fTcf21Cre groups, yet Extended Data Fig. 4f showed a survival difference. Clarify whether this is from the same cohort of animals. If yes, how can tumor size remain unchanged while survival differs significantly? Are there non-tumor causes of death? If not, the lack of synchronization should be noted.

Response: We thank the reviewer for this careful observation. Extended Data Fig. 4e and Extended Data Fig. 4f were generated from the same cohort of GL261-Luc–bearing mice. Tumor burden in Extended Data Fig. 4e was assessed non-terminally by bioluminescence imaging (BLI) at a predefined time point, after which the same animals were followed longitudinally for survival until predefined humane endpoints.

At this single imaging time point, tumor burden showed a trend but did not reach statistical significance between the $P65^{f/f}Tcf21^{Cre}$ + anti-CD8 and $P65^{f/f}Tcf21^{Cre}$ groups (P = 0.0689), whereas overall survival differed significantly (log-rank P = 0.0023). **These findings are not mutually exclusive because a single "snapshot" BLI measurement may not capture subsequent divergence in growth kinetics or clinically relevant features (e.g., invasiveness, edema, mass effect, and microenvironmental changes) that accumulate over time and influence survival.** Mice were euthanized strictly according to predefined tumor-related humane criteria, and no unexpected non–tumor-related deaths were observed in this cohort.

To further reduce potential confusion and address concerns about Cre specificity, **we have additionally performed the GL261-Luc experiments using a more fibroblast-specific Cre driver ($P65^{f/f}Col1a2^{Cre}$) in the revised manuscript.** In this synchronized paradigm, tumor burden and survival were assessed in a matched design and show concordant trends (Response Fig. 7).

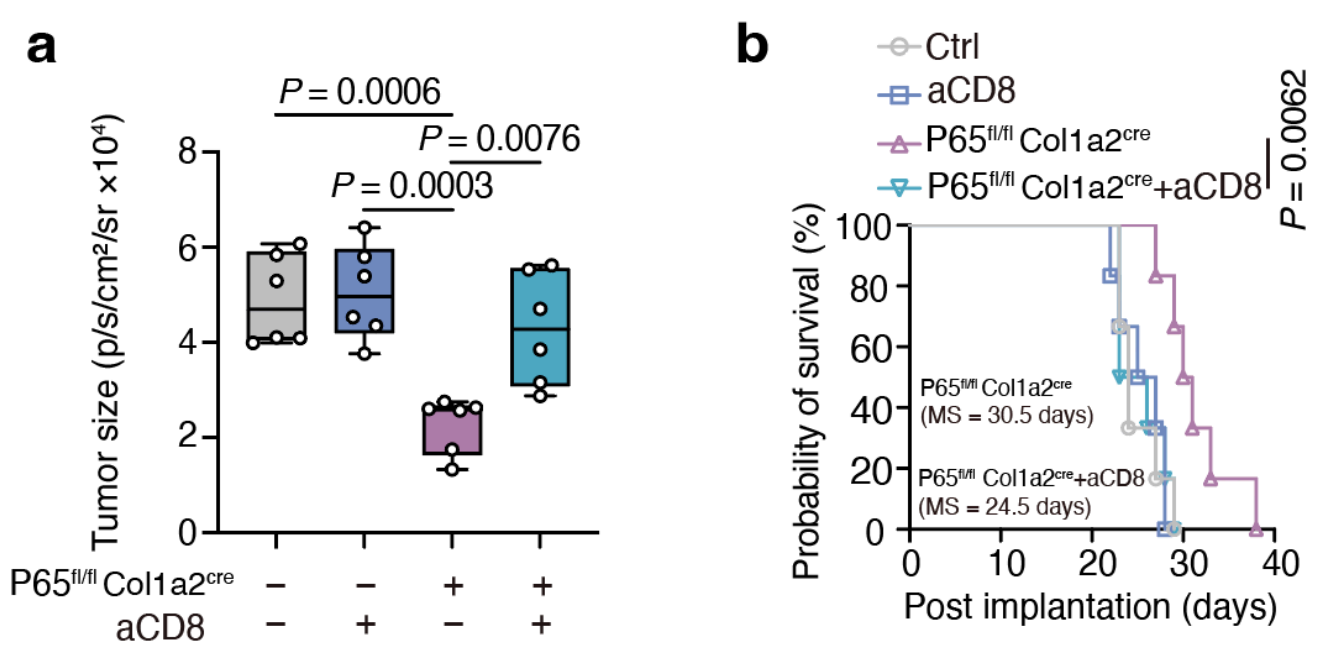

Response Figure. 7 | a, Quantification of tumour burden of GL261-Luc tumour-bearing mice in the $P65^{wt}$, $P65^{f/f}$ and $P65^{f/f}$ $Col1a2^{Cre}$ groups (mean ± s.d., n = 6 mice per group). **b,** Kaplan-Meier survival curves of GL261-Luc tumour-bearing mice in the $P65^{wt}$, $P65^{f/f}$, and $P65^{f/f}$ $Col1a2^{Cre}$ groups (n = 6 per group, Log-rank test).

3)Impact of outlier time points on statistics:
In Fig. 5e and Extended Data Fig. 5c, individual animals have measurement timepoints significantly earlier than others (e.g., one CT2A mouse in Fig. 5e measured only until day 28; one GL261 mouse in Extended Data Fig. 5c measured only until day 10). The authors should clarify:
a.Are these due to early death or technical failure?

Response: We thank the reviewer for raising this important point. **The early termination of measurements in Fig. 5e and Extended Data Fig. 5c was due to animals reaching predefined humane endpoints related to tumor progression, not technical or acquisition failures.**

Imaging was performed on prespecified days (including day 35 for CT2A and day 15 for GL261). The CT2A mouse in Fig. 5e was euthanized at day 34, and therefore its last available imaging time point was day 28 (the next scheduled scan at day 35 could not be performed). Similarly, the GL261 mouse in Extended Data Fig. 5c was euthanized at day 13, with the last available imaging time point at day 10 (the next scheduled scan at day 15 could not be performed).

b.Were they handled in the statistical analysis (e.g., excluded or noted)?

Response: These animals were included in the longitudinal analyses up to their last valid measurement and treated as missing thereafter; **no imputation or extrapolation was performed**. We have clarified this in the Methods, and we report the n per time point (Source Data) to make the variable follow-up explicit.

Page 21, Lines 876-879 (**Methods**): Missing values arising from variable follow-up (e.g., humane endpoints) were treated as missing without imputation; analyses used all available measurements up to the last valid time point, and n per time point is reported in the source data.

c.Do they affect the robustness of the overall statistical conclusions?

Response: To assess robustness, we performed sensitivity analyses excluding these individual animals (one per model). **The overall conclusions were unchanged.** Specifically, after exclusion, survival differences remained significant (GL261: median survival in the gCSF group = 27 days, log-rank $P = 0.002$; CT2A: median survival in the gCSF group = 39 days, log-rank $P = 0.0007$). These sensitivity analyses are provided in the Source Data (Fig.5; Extended Data Fig. 5; sheet: [*Sensitivity analyses*]).

5. Unclear rationale for gCSF infusion time points
1) Ambiguity of day 5 vs. day 10 and lack of explanation for day 10. In Fig. 5b–c, the authors chose to assess collagen and CSF clearance rate on day 5 post-gCSF infusion initiation, but the figures include data for day 10 without corresponding explanation in the main text. It must be clarified whether "day 10" refers to observation after 10 days of continuous infusion, or observation until day 10 after tumor implantation performed on day 5 of continuous infusion (i.e., 5 days post-implantation). Current description is unclear.

Response: We thank the reviewer for pointing out this ambiguity, and we apologize for the insufficient description of the experimental timeline in the original manuscript. In our study, gCSF was delivered continuously for 8 days using an osmotic minipump. Importantly, the time points in Fig. 5b-c are referenced to completion of the continuous gCSF infusion: **"day 5" and "day 10" denote 5 and 10 days after infusion completion, respectively.** We agree that the original text could be misread as "post-initiation," and we have corrected this to prevent confusion.

To eliminate ambiguity, **we revised the Results and Methods section** to explicitly define the time reference.

We appreciate the reviewer's comment, which has substantially improved the clarity of the experimental design and data presentation.

Page 7, Lines 278-287 (**Results**): Mice were analysed at the indicated time points (days 1-10) after completion of the continuous infusion. Immunofluorescence analysis showed significant expansion of COL1A1- and COL3A1-positive collagen areas in the leptomeninges of the gCSF group by day 5 after completion of infusio (Fig. 5b,c), while tracer-based analysis at the same time point demonstrated impairment of the CSF clearance-associated readout (Fig. 5c). Because this CSF clearance-associated deficit was detectable by day 5 and remained evident through day 10, day 5 was selected for subsequent tumour implantation experiment...Using this model, we next implanted orthotopic CT2A or GL261 tumours after completion of CSF infusion to evaluate the impact of a pre-established CSF clearance deficit on tumour growth and survival (Fig. 5a).

Page 13, Lines 525-534 (**Methods**): An osmotic pump (RWD Life Science, 1001W) was used to model CSF clearance impairment induced by gCSF. CSF was collected from the cisterna magna of healthy mice (control cCSF) and GBM-bearing mice, centrifuged to remove cellular debris, and stored until use. gCSF (or cCSF) was continuously infused into the lateral ventricle of recipient mice (stereotaxic coordinates: AP +0.5 mm; ML +1.0 mm; DV −2.5 mm) at a constant rate of 0.5 μL/hr using the osmotic pump implanted subcutaneously on the back. The pump reservoir (nominal volume 100 μL) provides ~8.3 days of infusion at this rate. Leptomeningeal fibrosis was assessed at indicated time points after completion of infusion (fluorescence staining procedures are described in "Histology and Morphology Analysis"). On day 5 after completion of infusion, an orthotopic CT2A-Luc/GL261-Luc tumor model was established for subsequent experiments.

2)Rationale for choosing day 5 for assessment. Infusion of 100μL at 0.5μL/hr takes approximately 8 days to complete. Why was assessment performed on day 5, before pump exhaustion? The CSF clearance rate is reported as identical on days 5 and 10. When did the difference emerge between days 1 and 5? Is there dynamic observation data supporting day 5 as a key timepoint? The rationale for selecting this specific timepoint needs to be explicitly stated.

Response: We thank the reviewer for this important question regarding the rationale for selecting day 5. To determine when gCSF-induced changes emerge and whether they persist, we performed a post-infusion time-course at day 1, 5, 7, and 10 after infusion completion (Response Fig. 8a-c). **At day 1, we did not observe a significant difference in CSF tracer clearance or leptomeningeal**

collagen readouts compared with controls. In contrast, by day 5, CSF clearance impairment and collagen deposition were clearly detectable. Importantly, the magnitude of these changes was similar at day 7 and day 10, indicating that the phenotype had reached a plateau rather than progressively worsening over this interval.

Therefore, we selected day 5 as the earliest post-infusion time point at which the phenotype is robust and reproducible and remains stable thereafter, enabling tumor implantation under conditions of an established (but not excessively delayed) CSF clearance deficit.

We appreciate the reviewer's constructive suggestion, which substantially improved the clarity of the experimental design and the interpretation of time-dependent effects.

Page 7, Lines 279-284 (**Results**): Immunofluorescence analysis showed significant expansion of COL1A1- and COL3A1-positive collagen areas in the leptomeninges of the gCSF group by day 5 after completion of infusion (Fig. 5b,c), while tracer-based analysis at the same time point demonstrated impairment of the CSF clearance-associated readout (Fig. 5c). Because this CSF clearance-associated deficit was detectable by day 5 and remained evident through day 10, day 5 was selected for subsequent tumour implantation experiments.

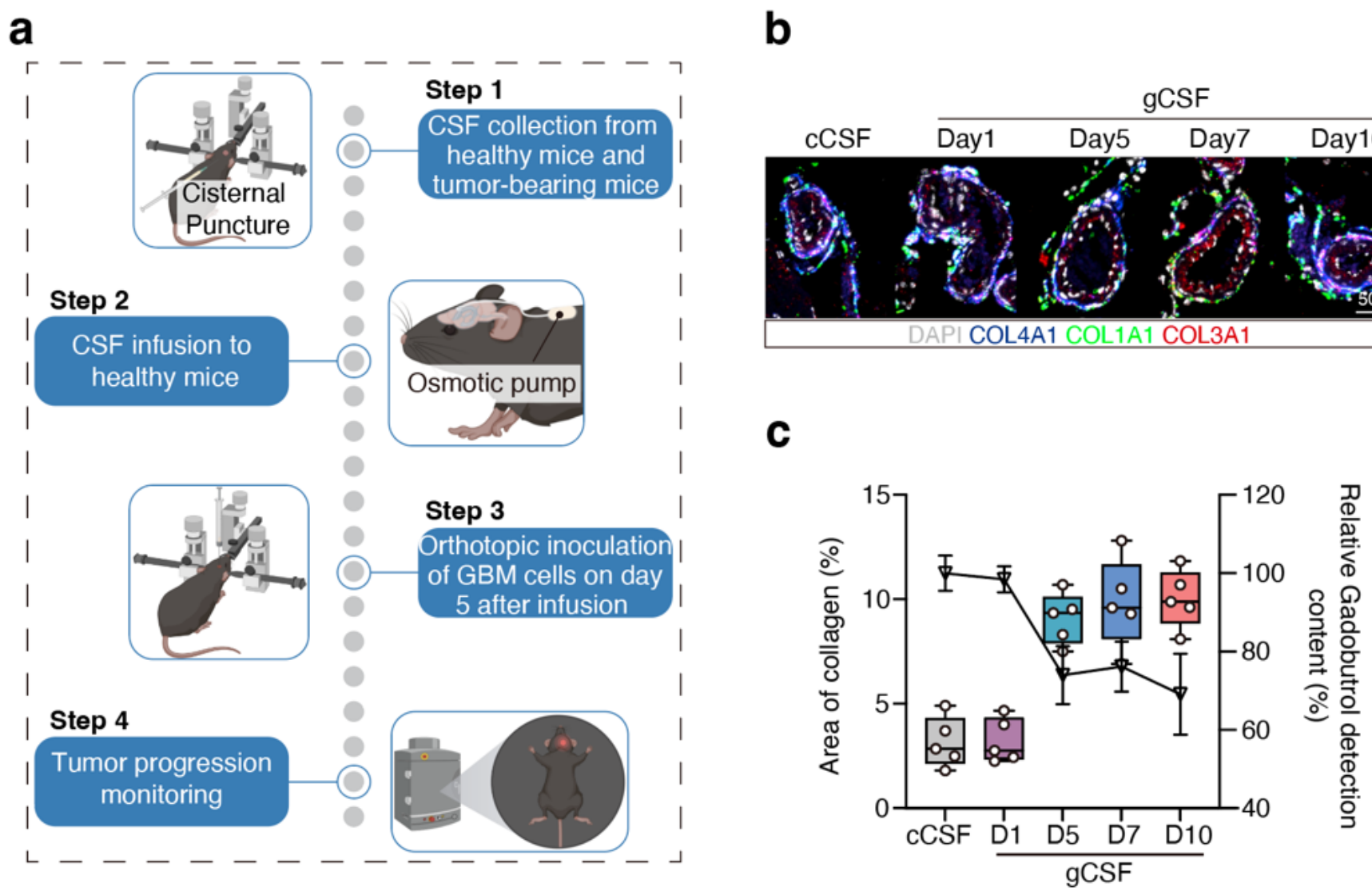

Response Figure. 8 | a, Schematic of the CSF infusion experiment. Osmotic pumps were used to infuse CSF from cCSF or gCSF into the lateral ventricles of healthy mice at a rate of 0.5 µL/hr (total volume: 100 µL). Orthotopic CT2A-Luc and GL261-Luc tumour models were established 5 days after completion of CSF infusion. Figure created with BioRender.com. **b,** Representative fluorescence images of leptomeningeal I/III collagen (COL1A1^{+}/COL3A1^{+}) in mice after completion of cCSF or gCSF infusion. Scale bar, 20 µm. **c,** Quantification of perivascular leptomeningeal COL1A1^{+}/COL3A1^{+} collagen area and relative gadobutrol content after completion of cCSF or gCSF infusion in mice (mean ± s.d., n = 5 per group).

6.Experimental validation of database analysis

The authors utilize a published single-cell database to conclude that gCSF does not enhance tumor cell proliferative capacity. This is a valuable finding; however, validating this using the study's own animal model samples (e.g., by performing RNA sequencing or qPCR on key genes related to "Quiescent.Score" and "Proliferation.Score" in tumor cells isolated from mice infused with cCSF or gCSF) would significantly strengthen the originality and persuasiveness of this conclusion. The authors are encouraged to consider supplementing the manuscript with such experimental data.

Response: We thank the reviewer for recognizing the value of single-cell database analysis and for the constructive suggestion to validate this conclusion using our own in vivo samples.

First, we have revised the manuscript to avoid overstatement. The published scRNA-seq dataset we analyzed compares tumor cells cultured in a CSF-like environment versus standard culture conditions; it was not designed to directly compare cCSF versus gCSF. We therefore now describe this analysis as evidence that a CSF-like environment does not upregulate, and may dampen, proliferation-associated transcriptional programs while showing relatively higher low-proliferation/quiescence features.

In line with the reviewer's recommendation, we performed additional experimental validation in our CSF infusion mouse model. We repeated cCSF or gCSF infusion and isolated tumor cells at day 3 (early) and day 21 (later) post-implantation. We assessed tumor-cell proliferation by (i) flow cytometry for Ki-67 in gated tumor cells (GFP^{+}) and (ii) qPCR for representative proliferation markers (Mki67, Top2a) (Response Fig. 9).

At day 3, we did not detect an increase in Ki-67 positivity or proliferation-marker mRNA in the gCSF group relative to controls, supporting the conclusion that gCSF does not exert an immediate, direct pro-proliferative effect on tumor cells in vivo. At day 21, proliferation markers were higher in the gCSF group, consistent with the increased tumor burden and accelerated progression observed under gCSF; we interpret this later increase as a feature of disease progression effects rather than evidence of an early cell-intrinsic proliferative switch. We have now incorporated these additional validation data and the corresponding rationale into the revised Results section.

We sincerely thank the reviewer for the thoughtful and constructive comments throughout the review. These suggestions have substantially improved the clarity and overall quality of the manuscript.

Page 7-8, Lines 291-303 (**Results**): Using a previously published single-cell RNA-sequencing dataset, we compared quiescence- and proliferation-related transcriptional programme scores in malignant cells across groups and found no evidence for higher proliferation-associated scores in the gCSF-associated condition (Fig. 5g). To further assess this in vivo, we performed a separate experiment using the CSF infusion model and analysed tumour cells at days 3 and 21 after implantation. Proliferation was assessed at both the protein and mRNA levels by flow cytometry and qPCR for Ki-67 and representative proliferation markers (Mki67 and Top2a) (Extended Data Fig. 5f-i). No significant increase in proliferation markers was detected in the gCSF group at day 3. By day 21,

however, these markers were significantly elevated in the gCSF group (Extended Data Fig. 5h,i), consistent with the increased tumour burden observed at later stages. Together, these findings suggest that, in this gCSF infusion model, accelerated tumour progression is unlikely to be explained by an immediate increase in tumour-cell proliferation and is instead more consistent with early changes in the non-tumour microenvironment, with increased tumour-cell proliferation emerging at later stages.

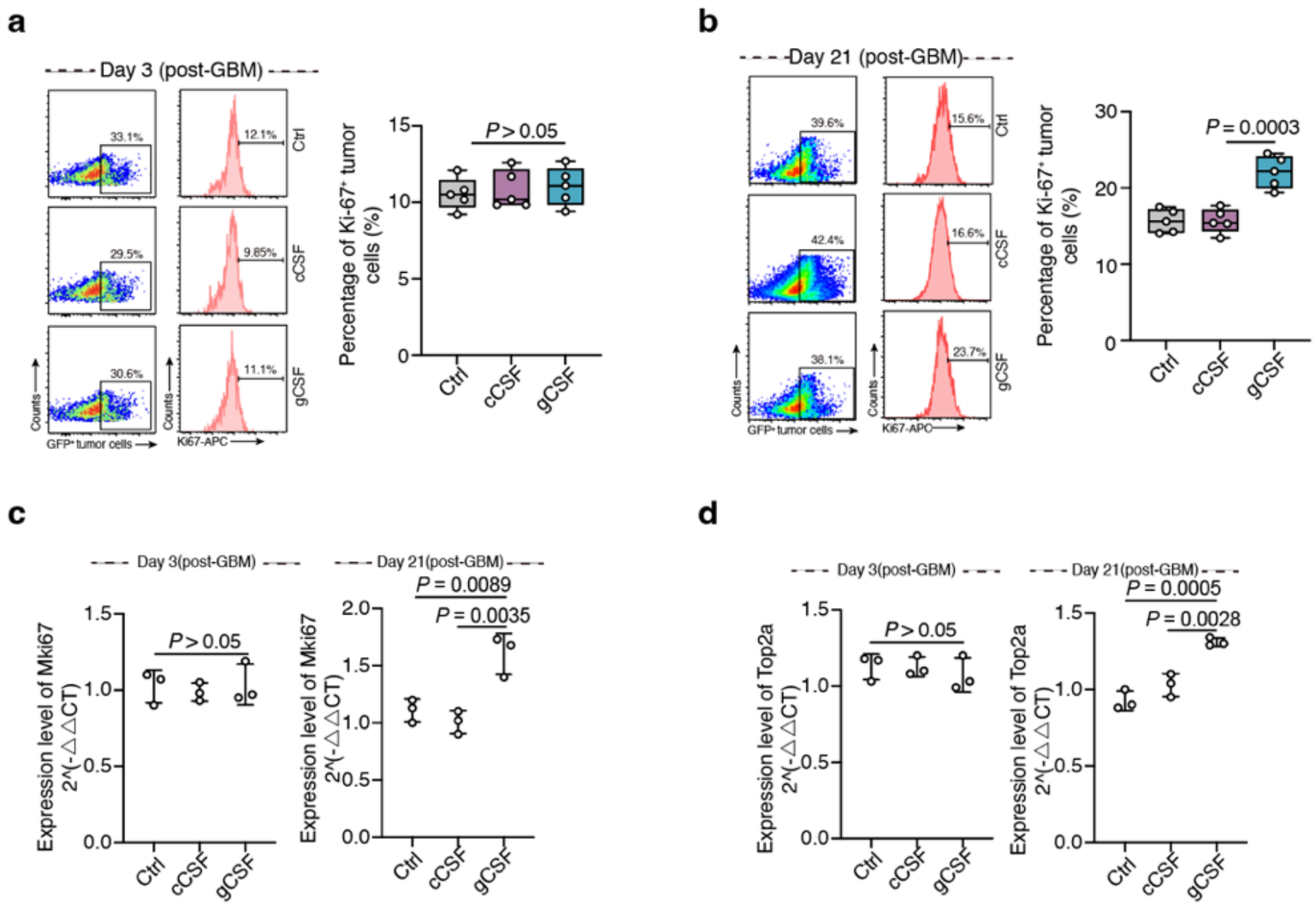

Response Figure. 9 | a, Representative flow cytometry plots and quantification of GFP⁺Ki-67⁺ tumor cells at day 3 post-implantation following cCSF or gCSF infusion. **b,** Representative flow cytometry plots and quantification of GFP⁺Ki-67⁺ tumor cells at day 21 post-implantation following cCSF or gCSF infusion. (mean ± s.d.; n = 5 mice per group) **c,** qPCR analysis of Mki67 expression in isolated tumor cells at day 3 and day 21 post-implantation. **d,** qPCR analysis of Top2a expression in isolated tumor cells at day 3 and day 21 post-implantation. (mean ± s.d.; n = 3 mice per group)

Reviewer #2 (Remarks to the Author):

This manuscript by Sun et al. establishes a causal link between GBM progression and NF κB/p65-dependent leptomeningeal perivascular fibrosis, which leads to CSF clearance defects, $CD8^{+}$ T cell exhaustion, and reduced anti PD 1 efficacy. The authors support these findings with evidence from orthotopic GBM mouse models, human CSF proteomics, leptomeningeal transcriptomics, and paired patient leptomeningeal–tumor analyses. Key findings include:

• Mice with orthotopic GBM tumors, or following tumor resection, exhibit reduced CSF clearance and drainage to lymph nodes and blood, as well as increased leptomeningeal collagen and α-SMA+ fibroblasts.

• GBM patient CSF displays a pro fibrotic, NF κB–dependent protein signature that drives human leptomeningeal fibroblasts toward $CTHRC1^{+}\alpha\ SMA^{+}$ perivascular myofibroblasts in vitro. Similar molecular signatures are observed in the CSF of GBM-bearing mice, supporting the use of these mouse models in testing therapies targeting the CSF–leptomeningeal–immune axis.

• Tcf21 specific p65 ablation reduces leptomeningeal collagen, restores CSF clearance, increases intratumoral $CD8^{+}$ T cell infiltration, and immune effector signatures. This loss of p65 attenuates GBM growth and improves survival, and this effect is dependent on T cell engagement, as CD8 cell depletion eliminates the survival benefit.

• Conditional p65 knockout and CSF DHMEQ enhance anti-PD-1 therapy and increase functional CD8+ T cells in mice. In patients, leptomeningeal fibrosis correlates with exhausted T-cell signatures.

Strengths:

These findings are significant for GBM, CSF biology, and CNS barrier and immune research, as they demonstrate a role for lymphatic dynamics in GBM tumor immunology and establish leptomeningeal fibroblasts as key regulators of brain tumor immunity. They also identify NF κB /p65 as a potential therapeutic target to remodel the immunosuppressive GBM microenvironment and enhance sensitivity to immune checkpoint inhibition.

The experimental design is mechanistically sound and robust. The authors' major claims are supported by data from multiple independent and complementary approaches, and there is sufficient detail in the methodology to support the reproducibility of the reported findings. Statistical analysis methods are clearly specified and consistently described throughout.

The use of Tcf21 fibroblast specific p65 deletion, as well as a newly established CSF replacement model to mimic CSF-induced clearance impairment, enables the authors to distinguish tumor intrinsic from CSF/meningeal mediated effects on GBM progression and provides greater mechanistic clarity vs. prior (bulk) analyses of GBM lymphatics or the tumor microenvironment.

Overall, these studies and the conceptual advances they present advance mechanistic insight and therapeutic potential in GBM neuro-oncology, immuno-oncology, and CNS barrier research.

Response: We sincerely thank you for the thoughtful and constructive assessment of our manuscript. We greatly appreciate the reviewer's recognition of the significance of our findings, the rigor of the experimental design, and the strength of the multi-model and multi-omics evidence. The reviewer's positive feedback and insights are truly valuable and have helped us improve the clarity and presentation of the work.

Despite these noted strengths, several issues in the manuscript should be addressed:

• The data support a causal link between this axis and GBM progression and immune checkpoint inhibitor resistance. However, claims regarding generalizability across GBM subtypes and potential clinical shunting strategies are less well supported and overstated.

Response: We thank the reviewer for this important and constructive comment. **We agree that our original wording could be interpreted as overstating the generalizability of our findings across GBM molecular subtypes and the near-term clinical implications regarding CSF shunting.**

Accordingly, we revised the Discussion to more explicitly delimit the scope of our conclusions. **We now state that the mechanistic evidence is derived from the GL261 and CT-2A models**, and that the human data provide correlative consistency rather than definitive subtype-wide validation. We also added an explicit limitation noting that applicability across GBM subtypes will require confirmation in larger, subtype-stratified patient cohorts and additional preclinical models.

Finally, **we revised the section discussing VP shunting** to describe the reported survival association as observational and hypothesis-generating, removed language that could be read as a therapeutic recommendation, and emphasized that any clinical benefit, safety, and patient selection would need to be evaluated prospectively.

Page 11, Lines 437-442 (**Discussion**): Second, our mechanistic evidence is derived primarily from the GL261 and CT-2A models together with correlative human datasets; therefore, the extent to which this CSF-leptomeningeal fibrosis axis generalizes across molecular GBM subtypes remains to be established in larger, subtype-stratified cohorts and additional models. Although we controlled for age and sex, our clinical analyses remain susceptible to residual confounding by molecular subtype and treatment-related factors.

Page 10-11, Lines 428-431 (**Discussion**): Additionally, VP shunt placement for CSF diversion has been associated with overall survival in subsets of GBM patients; however, whether modulating CSF dynamics benefits unselected newly diagnosed patients remains unknown and requires prospective study.

• The authors show that p65 loss in leptomeningeal fibroblasts does not affect healthy mice. However, off target effects due to recombination in other $Tcf21^{+}$ populations cannot be completely excluded. Additional marker co staining or lineage tracing data are needed to confirm that the phenotype is fibroblast specific.

Response: We thank Reviewer for this important point regarding the specificity of the Tcf21-Cre system. We agree that recombination in other $Tcf21^{+}$ populations cannot be completely excluded, and that Tcf21-Cre alone is therefore insufficient to definitively attribute the phenotype exclusively to leptomeningeal fibroblasts.

To directly address this, we added additional validation experiments and revised the manuscript to more carefully define the targeted compartment and to avoid overstatement.

First, **we performed lineage-tracing experiments using Rosa26-LSL-tdTomato reporter mice crossed to tamoxifen-inducible Cre lines,** with recombination induced prior to tumor implantation to label pre-existing cell populations. Specifically, we used NG2(Cspg4)-CreERT2 to label mural cells and Col1a2-CreERT2 to label Col1a2-expressing fibroblast-lineage cells at baseline. Mice were administered tamoxifen at 6 weeks of age, implanted with tumor at 8 weeks, and brains were collected at 10 weeks for Col1 staining (Response Fig. 10a). We observed a marked expansion of $tdTomato^+$ cells in the Col1a2-CreERT2 lineage within the leptomeningeal $Col1a1^+$ fibrotic regions, whereas NG2-CreERT2 lineage-labeled cells did not show a comparable increase in these fibrotic areas (Response Fig. 10b-c). These data support a predominantly fibroblast-lineage ($Col1a2^+$) origin of the leptomeningeal fibrotic response and argue against a major pericyte/mural-cell contribution.

Second, to further reduce reliance on Tcf21-Cre specificity, we complemented the Tcf21-Cre model with an independent Col1a2-targeted approach (Col1a2-AAV-Cre-based conditional p65 suppression (Response Fig. 10d). **Using multiplex immunofluorescence, we confirmed that the Col1a2-targeted recombination signal co-localizes with fibroblast-associated markers and does not overlap with markers of mural cells, endothelial cells, or immune cells in the analyzed compartment (Response Fig. 10e).** Functionally, inhibiting NF-κB signaling within $Col1a2^+$ cells attenuated leptomeningeal fibrosis and improved CSF clearance and downstream immune phenotypes (Response Fig. 10f-g), consistent with our central conclusions.

Together, these additions strengthen the cell-type attribution and substantially mitigate concerns regarding off-target recombination.

Page 3, Lines 100-109 (**Results**): In the CNS, collagen-producing stromal cells implicated in fibrosis have been reported to include COL1-expressing fibroblasts and pericytes. To assess the lineage contribution to GBM-associated leptomeningeal perivascular collagen deposition, we performed genetic fate mapping using Col1a2CreERT2; Rosa26-LSL-tdTomato and NG2(Cspg4)CreERT2; Rosa26-LSL-tdTomato mice (Fig. 2a). In GBM-bearing mice, we observed an approximately 3.9-fold increase in Col1a2CreERT2-labelled cells at the leptomeningeal interface that co-localized with COL1+ fibrotic regions, whereas NG2CreERT2-traced cells did not show a comparable increase (Fig. 2b,c). These results suggest that Col1a2-lineage fibroblasts represent a major expanding stromal population associated with leptomeningeal perivascular collagen deposition in this model, whereas contribution from $NG2^+$ mural cells appears comparatively limited.

Page 5, Lines 190-206 (**Results**): To examine the in vivo relevance of fibroblast NF-κB/P65 signalling, we combined P65-floxed mice with AAV9 vectors encoding Col1a2-driven Cre recombinase (pAAV9-Col1a2-Cre-mCherry) or a matched control lacking Cre (pAAV9-Col1a2-mCherry). Intracisternal administration of pAAV9-Col1a2-Cre-mCherry enabled conditional P65 deletion with preferential targeting of leptomeningeal $Col1a2^+$ fibroblast-like cells (Fig. 3f). We first assessed the targeting pattern of this approach and found that mCherry signal was observed mainly in $Col1a2^+$ leptomeningeal fibroblast-like cells, with little or no detectable overlap with endothelial, pericyte or immune-cell markers in the regions examined (Extended Data Fig. 3f). Multiplex immunofluorescence confirmed efficient reduction of P65 signal in leptomeningeal fibroblast-like cells in the pAAV9-Col1a2-Cre-mCherry group relative to the control virus group (Fig. 3g). Using

this Col1a2-targeted conditional P65 deletion strategy, we then generated GBM-bearing mice for in vivo analysis. In GBM-bearing leptomeninges, $P65^{f/f}$ mice receiving pAAV9-Col1a2-Cre-mCherry showed reduced type I/III collagen accumulation (COL1A1/COL3A1) compared with both control groups, namely $P65^{f/f}$ mice receiving the control virus and WT mice receiving pAAV9-Col1a2-Cre-mCherry (Extended Data Fig. 3g). Functionally, the CSF clearance-associated readout was significantly improved in $P65^{f/f}$ mice receiving pAAV9-Col1a2-Cre-mCherry compared with $P65^{f/f}$ groups (Fig. 3h). Targeted P65 deletion in $Col1a2^{+}$ cells did not alter the CSF clearance-associated readout in healthy mice (Extended Data Fig. 3h).

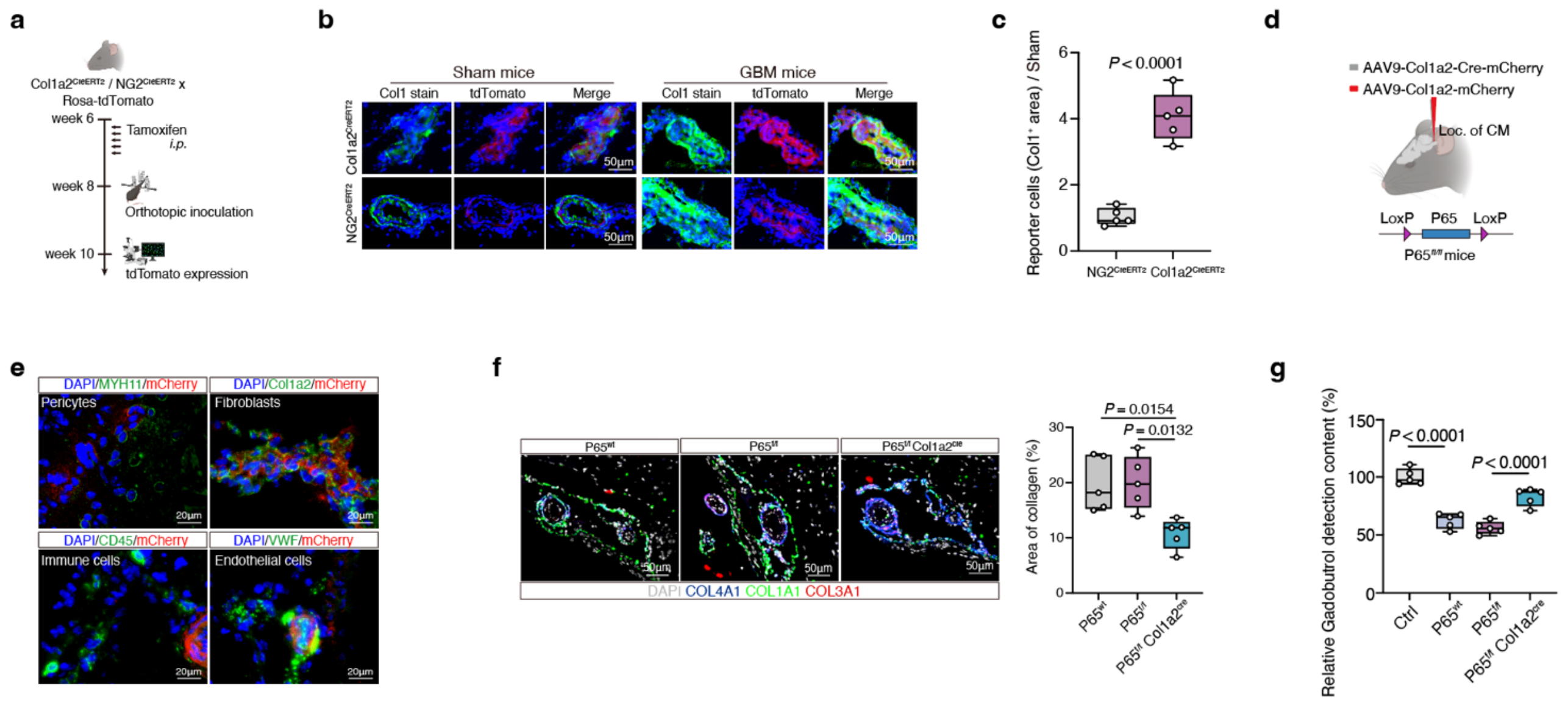

Response Figure. 10 | a, Schematic of inducible genetic fate mapping used to trace COL1-lineage fibroblasts ($Col1a2^{CreERT2}$; Rosa26-LSL-tdTomato) and NG2-lineage mural cells ($NG2^{CreERT2}$; Rosa26-LSL-tdTomato) in mouse leptomeninges under sham and GBM conditions. **b,** Representative images of the leptomeningeal interface showing tdTomato lineage-labelled cells and COL1 immunostaining in sham-operated and GBM tumour-bearing mice. **c,** Quantification of reporter cells at the leptomeningeal interface, normalized to sham-operated controls (mean ± s.d., n = 5 mice per group). **d,** Schematic of the strategy used for conditional deletion of P65 in Col1a2-targeted leptomeningeal fibroblasts. AAV9 vectors expressing Cre-mCherry under the Col1a2 promoter (pAAV9-Col1a2-Cre-mCherry) or control mCherry vectors (pAAV9-Col1a2-mCherry) were injected into the cisterna magna of $P65^{f/f}$ mice. Figure created with BioRender.com. **e,** Representative images assessing AAV9-mCherry tropism in leptomeningeal tissues by co-staining with fibroblast, endothelial, mural and immune cell markers. Scale bar, 20 μm. **f,** Left, representative images of type I/III collagen fibres in the leptomeninges of GBM tumour-bearing mice in the indicated groups. Scale bar, 50 μm. Right, quantification of type I/III collagen fibre area in the leptomeninges (mean ± s.d., n = 5 mice per group). **g,** Relative gadobutrol content detected in CSF from GBM tumour-bearing mice of the indicated genotypes (mean ± s.d., n = 5 mice per group).

• MRI data from patients show increased ventricular flow velocities, but CSF clearance is not directly measured. Therefore, the authors should avoid overinterpreting the data to conclude that flow velocity causally impacts CSF clearance.

Response: We thank the reviewer for this important point and fully agree. In our patient cohort, phase-contrast MRI (PC-MRI) quantifies regional ventricular CSF flow/velocity metrics and does not directly measure CSF clearance. Therefore, these data should be interpreted as evidence of altered CSF hydrodynamics rather than as proof that increased velocity causally impairs clearance.

In the revised manuscript, we removed causal language linking higher ventricular velocity to reduced CSF clearance in patients, andadded an explicit statement in the Results clarifying that CSF clearance was not assessed clinically and cannot be inferred from PC-MRI alone. We now frame impaired CSF clearance in humans as a testable hypothesis that is mechanistically supported by our direct CSF clearance assays in mouse models, which will require dedicated clinical clearance measurements for validation.

Page 3, Lines 95-97 (**Results**): Because PC-MRI primarily reports local kinematics at selected imaging planes rather than whole-system CSF turnover, we interpret these findings as evidence of regionally altered ventricular CSF dynamics, which may not directly track global CSF clearance.

• Although the patient cohorts studied are modest in size, the authors control for age and sex and use external controls for RNA seq analysis. However, they do not adequately address potential confounders, such as GBM molecular subtypes and the effects of treatment (e.g., steroids) on immune and CSF phenotypes. A more in-depth clinical analysis and/or a discussion are recommended.

Response: We thank the reviewer for this important recommendation and agree that, beyond age and sex, additional clinical factors, particularly GBM molecular features and treatment-related variables such as corticosteroid exposure, may confound immune- and CSF-related phenotypes.

To address this, **we expanded the clinical reporting and strengthened the Discussion** in the revised manuscript. First, where molecular information was available (e.g., IDH status, key pathology/IHC markers as reported clinically), we performed exploratory stratified analyses of the main human readouts (PC-MRI ventricular CSF velocity metrics and the RNA-seq-based immune signatures reported in the manuscript). Because the cohort is modest and subgroup sizes are small, these analyses are presented as descriptive and we explicitly avoid overinterpretation. We now report molecular data availability and patient-level clinicopathologic annotations in Response Table 1.

Second, **we reviewed medication records in the peri-imaging/sampling window and added corticosteroid exposure information to the baseline characteristics table.** We also added a limitation statement in the Discussion emphasizing that steroid use and other treatment factors (e.g., prior therapies, anti-edema management) remain important potential confounders and should be systematically recorded and controlled in future prospective, subtype-stratified clinical studies.

We believe these additions improve transparency and better align the strength of our clinical inferences with the available data.

Page 11, Lines 437-442 (**Discussion**): Second, our mechanistic evidence is derived primarily from the GL261 and CT-2A models together with correlative human datasets; therefore, the extent to which

this CSF-leptomeningeal fibrosis axis generalizes across molecular GBM subtypes remains to be established in larger, subtype-stratified cohorts and additional models. Although we controlled for age and sex, our clinical analyses remain susceptible to residual confounding by molecular subtype and treatment-related factors.

Response Table 1. Baseline clinicopathologic characteristics and peri-sampling steroid exposure of the primary GBM patient cohort for RNA-seq analysis. Clinical demographics (sex and age), tumor location, and immunohistochemical/molecular marker status are shown for each patient. Marker annotations follow the original pathology reports ("+", "−", "partial +", "focal +", and "weak +" indicate the reported staining pattern/intensity). NA indicates that a marker was not assessed or not reported.

Sex	Age	Tumor location	GFAP	OLIG2	P53	ATRX	IDH1	H3K27M	H3K27me3	Nestin	NeuN	BRAF V600E	CIC	FUBP1	CD34	H3G34V	CK	Treatment
M	73	Right temporal	+	+	focal +	+	−	−	NA	+	−	NA	NA	NA	NA	NA	NA	NA
M	54	Occipital	+	+	+	+	−	−	NA	+	NA	−	NA	NA	NA	NA	NA	NA
F	54	Right parietal	+	+	+	+	−	−	NA	+	NA	NA	NA	NA	NA	NA	NA	NA
F	67	Right frontal	+	+	+	+	−	−	+	NA	NA	NA	NA	NA	NA	NA	NA	NA
M	28	Right frontal	+	+	+	focal +	−	−	partial +	NA	NA	NA	NA	NA	NA	NA	NA	NA
M	69	Left occipital	+	+	+	+	−	NA	NA	+/−	NA	NA	NA	NA	NA	NA	−	NA
F	38	Left frontal	partial +	+	+	+	−	−	NA	NA	NA	+	NA	NA	NA	NA	NA	NA
M	62	Right thalamic	+	+	+	+	−	−	NA	NA	−	−	+	+	NA	NA	NA	NA
F	38	Left parietal	+	+	focal +	+	−	NA	NA	+	NA	weak +	+	+	vascular +	NA	NA	NA
M	50	Left frontal-corpus callosum	+	+	+/−	+	−	−	NA	+	NA	−	+	+	NA	NA	NA	NA
F	51	Right hemispheric	+	low +	+/−	+	−	−	NA	+	NA	−	+	−	NA	NA	NA	NA
M	40	Left temporal	+	partial +	−	+	−	−	NA	+	NA	+/−	+	+	NA	NA	NA	NA
F	42	Left frontal basal ganglia	+	+	partial +	+	−	−	partial +	NA	NA	−	−/+	+	NA	NA	NA	NA
F	46	Right frontal	+	+	−	partial +	−	−	+	−	NA	−	NA	+	vascular +	−	NA	NA
M	58	Right parietal	+	weak +	+	+	−	−	+	−	NA	focal +	NA	+	partial +	−	NA	NA

• Proteomics data suggest a role for PDGFC, PDGFD, and LGALS9, and PDGFRβ is expressed on perivascular fibroblasts. However, functional analyses (e.g., experiments using PDGFR inhibition or neutralizing antibodies) would be needed to validate these factors as molecular drivers. In the absence of additional mechanistic data, referring to these newly identified proteins as markers, rather than drivers, would be more accurate and avoid overstating conclusions.

Response: We thank the reviewer for this important point and agree. Our proteomic data nominate PDGFC, PDGFD and LGALS9 as CSF-enriched candidate markers associated with the CSF-fibroblast axis, and our expression analyses indicate that PDGFRβ is expressed on perivascular fibroblasts; however, our current study does not include perturbation experiments to establish these factors as molecular drivers.

New functional inhibition experiment: To provide functional evidence for PDGF pathway involvement, we performed additional perturbation experiments targeting PDGFRβ using CP-673451 (200 nM, 12 h) in leptomeningeal fibroblasts. PDGFRβ inhibition significantly attenuated the gCSF-

induced profibrotic activation phenotype, reducing the αSMA/Col1a1 double-positive population from 52.2517 to 25.4867 (approximately 51.2% reduction) (Response Figure. 11). These data support that PDGFRβ signaling contributes, at least in part, to the CSF-driven phenotypic switch.

We acknowledge that our current data do not yet establish a single sufficient molecule or a minimal recombinant factor combination with defined dose ranges that fully recapitulates the phenotype, nor do they yet demonstrate in vivo necessity of PDGFRβ or LGALS9 signaling. **We have explicitly stated this limitation** and outlined follow-up experiments (recombinant add-back across physiologic/pathologic ranges, immunodepletion/neutralization, and in vivo perturbation/validation) in the Discussion

Page 9-10, Lines 384-389 (**Discussion**): Nevertheless, although PDGFC, PDGFD and LGALS9 are enriched in gCSF and PDGFRβ is expressed on perivascular fibroblasts, these data remain correlative. Establishing a causal PDGF-PDGFRβ- or LGALS9-dependent mechanism will require targeted perturbation studies. More broadly, the CSF-induced fibroblast phenotype is likely driven by the synergy of multiple CSF-borne factors rather than by a single dominant mediator.

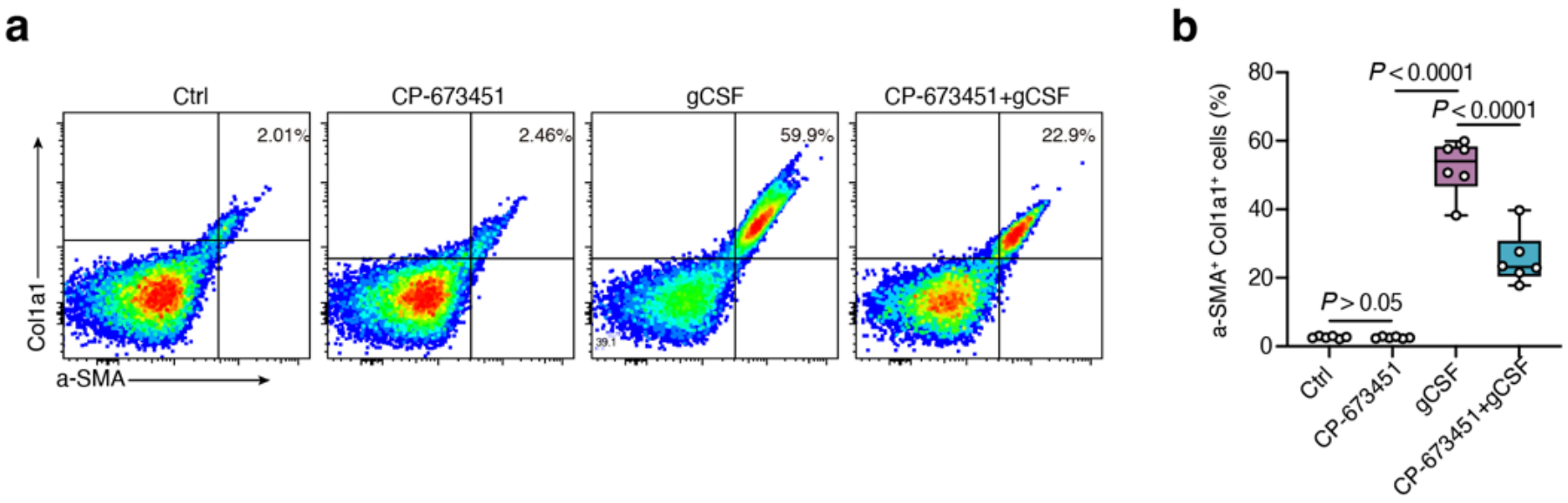

Response Figure. 11 | a, Representative flow cytometry plots showing αSMA and Col1a1 staining in leptomeningeal fibroblasts treated with control medium (Ctrl), CP-673451 (200 nM), GBM-derived CSF (gCSF), or gCSF+CP-673451. **b,** Quantification of $\alpha SMA^{+}Col1a1^{+}$ cells (mean ± s.d., n = 6).

• In their statistical analyses, the authors use simple unpaired t tests or ANOVA across many imaging and flow cytometry results that have multiple endpoints (e.g., different collagen metrics, immune cell subsets, activation markers). Without any global correction applied across all panels or families of related tests, this raises a multiple-comparisons issue that can potentially inflate type I error (rejecting a true null hypothesis and concluding there is a real effect or difference when, in fact, there is none). As such, to enhance rigor, detailed explanations of corrections beyond Tukey's post tests (e.g., Holm–Šidák, Benjamini–Hochberg FDR) are needed, along with specifying exact sample sizes and effect sizes in each panel.

Response: We thank the reviewer for raising this important multiple-comparisons concern. We agree that, for figures reporting multiple related endpoints (e.g., multiple collagen readouts and multiple flow-cytometry cell subsets/activation markers), testing each endpoint independently can inflate the family-wise type I error rate if multiplicity is not controlled across a pre-specified family of hypotheses.

In the revision, **we defined multiplicity "families" as sets of endpoints derived from the same experiment** (same biological sample set, platform, and time point/processing pipeline) and **applied family-level corrections accordingly.** The families are: (i) Figs. 1g-h (collagen imaging endpoints), (ii) Figs. 6d-f (CT2A flow-cytometry endpoints), and (iii) Extended Data Figs. 6e-g (GL261 flow-cytometry endpoints). For each family, we applied the Holm-Šidák procedure to the set of endpoint-level P values to control the family-wise error rate, and we report both raw and adjusted P values in the Source Data. We also now report the exact n for every panel and provide effect sizes (mean differences with 95% CIs for pairwise comparisons for ANOVA-based contrasts) in the Response Table 2-4.

The overall conclusions are unchanged after applying these corrections. We appreciate the reviewer's suggestions, which have improved the statistical rigor and transparency of the study.

Response Table 2. Family#1, Figs. 1g-h.

**Family#1 (collagen metrics)**		
**Figure/Panel**	**Fig.1g**	**Fig.1h**
**Endpoint**	Sirius red area	a-SMA+ cells/hpf
**Comparison**	Non-tumor vs. GBM	
**Sample sizes**	n =10	
**Mean**	5.8026, 14.0074	9.22978, 32.9954
**Effect size（mean difference）**	-8.2048	-23.7656
**Unpaired t test P**	<0.0001	0.0001
**Holm–Šidák adjusted P**	0.0225	<0.0001

Response Table 3. Family#2, Figs. 6d-f.

**Family#2 (immune cell subsets-CT2A)**					
**Figure/Panel**	**Fig. 6d**				
**Endpoint**	**CD8+ CD45+ T cells**				
**Comparison**	Ctrl vs. aPD1	Ctrl vs. P65f/f Col1a2cre	Ctrl vs. P65f/f Col1a2cre+aPD1	aPD1 vs. P65f/f Col1a2cre+aPD1	P65f/f Col1a2cre vs. P65f/f Col1a2cre+aPD1
**Sample sizes**	n = 4				

**Mean**	9.78, 15.3875	9.78, 11.3375	9.78, 24.8625	15.3875, 24.8625	11.3375, 24.8625
**Effect size （mean difference）**	-5.6075	-1.5575	-15.0825	-9.475	-13.525
**Tukey's multiple comparisons adjusted P**	0.0043	0.6278	<0.0001	<0.0001	<0.0001
**Holm–Šidák adjusted P**	0.0024	0.3168	<0.0001	<0.0001	<0.0001
**Figure/Panel**	**Fig. 6e**				
**Endpoint**	**PD1+ T cells**				
**Comparison**	Ctrl vs. aPD1	Ctrl vs. P65f/f Col1a2cre	Ctrl vs. P65f/f Col1a2cre+aPD1	aPD1 vs. P65f/f Col1a2cre+aPD1	P65f/f Col1a2cre vs. P65f/f Col1a2cre+aPD1
**Sample sizes**	n = 4				
**Mean**	23.3750, 16.0500	23.3750, 15.8125	23.3750, 6.16750	16.0500, 6.16750	15.8125, 6.16750
**Effect size （mean difference）**	7.325	7.5625	17.2075	9.8825	9.645
**Tukey's multiple comparisons adjusted P**	0.0021	0.0016	<0.0001	0.0001	0.0002
**Holm–Šidák adjusted P**	<0.0001	<0.0001	<0.0001	<0.0001	<0.0001
**Figure/Panel**	**Fig. 6f**				
**Endpoint**	**IFNγ+ T cells**				
**Comparison**	Ctrl vs. aPD1	Ctrl vs. P65f/f Col1a2cre	Ctrl vs. P65f/f Col1a2cre+aPD1	aPD1 vs. P65f/f Col1a2cre+aPD1	P65f/f Col1a2cre vs. P65f/f Col1a2cre+aPD1
**Sample sizes**	n = 4				
**Mean**	10.8750, 15.3125	10.8750, 12.4000	10.8750, 25.9125	15.3875, 25.9125	12.4000, 25.9125
**Effect size （mean difference）**	-5.6075	-1.5575	-15.0825	-9.475	-13.525

**Tukey's multiple comparisons adjusted P**	0.1078	0.8229	<0.0001	0.0003	<0.0001
**Holm–Šidák adjusted P**	0.0192	0.3268	<0.0001	<0.0001	<0.0001

Response Table 4. Family#3, Extended Data Figs. 6e-g.

**Family#3 (immune cell subsets-GL261)**					
**Figure/Panel**	**Extended Data Fig. 6e**				
**Endpoint**	**CD8+ CD45+ T cells**				
**Comparison**	Ctrl vs. aPD1	Ctrl vs. P65 f/f Col1a2cre	Ctrl vs. P65f/f Col1a2cre+aPD1	aPD1 vs. P65f/f Col1a2cre+aPD1	P65f/f Col1a2cre vs. P65f/f Col1a2cre+aPD1
**Sample sizes**	n = 4				
**Mean**	15.3500,23.8500	15.3500, 20.3500	15.3500, 35.8500	23.8500, 35.8500	20.3500, 35.8500
**Effect size（mean difference）**	-8.5	-5	-20.5	-12	-15.5
**Tukey's multiple comparisons adjusted P**	0.006	0.1181	<0.0001	0.0004	<0.0001
**Holm–Šidák adjusted P**	0.0001	0.0184	<0.0001	<0.0001	<0.0001
**Figure/Panel**	**Extended Data Fig. 6f**				
**Endpoint**	**PD1+ T cells**				
**Comparison**	Ctrl vs. aPD1	Ctrl vs. P65 f/f Col1a2cre	Ctrl vs. P65f/f Col1a2cre+aPD1	aPD1 vs. P65f/f Col1a2cre+aPD1	P65f/f Col1a2cre vs. P65f/f Col1a2cre+aPD1
**Sample sizes**	n = 4				
**Mean**	19.4375,13.1625	19.4375, 10.0000	19.4375, 4.16000	13.1625, 4.16000	10.0000, 4.16000
**Effect size（mean difference）**	6.275	9.4375	15.2775	9.0025	5.84

**Tukey's multiple comparisons adjusted P**	0.0227	0.0012	<0.0001	0.0018	0.0344
**Holm–Šidák adjusted P**	0.0043	<0.0001	<0.0001	<0.0001	0.0055
**Figure/Panel**	**Extended Data Fig. 6g**				
**Endpoint**	**IFNγ+ T cells**				
**Comparison**	Ctrl vs. aPD1	Ctrl vs. P65f/f Col1a2cre	Ctrl vs. P65f/f Col1a2cre+aPD1	aPD1 vs. P65f/f Col1a2cre+aPD1	P65f/f Col1a2cre vs. P65f/f Col1a2cre+aPD1
**Sample sizes**	n = 4				
**Mean**	17.9000,22.3250	17.9000, 21.5250	17.9000, 27.7000	22.3250, 27.7000	21.5250, 27.7000
**Effect size（mean difference）**	-4.425	-3.625	-9.8	-5.375	-6.175
**Tukey's multiple comparisons adjusted P**	0.0616	0.1448	0.0002	0.0213	0.0213
**Holm–Šidák adjusted P**	0.0588	0.1046	<0.0001	0.0086	0.0086

• A prior publication demonstrated that the therapeutic effect of anti-PD-1 blockade in GBM is mediated by macrophage/microglia M1 polarization, rather than by CD8 T cells (Rao et al., 2020, PMID: 32554515). The authors' findings and potential involvement of the innate immune system should be discussed and interpreted in this context.

Response: We thank the reviewer for highlighting the important study by Rao et al[1], which showed that anti-PD-1 efficacy in GBM can be mediated predominantly through macrophage/microglia polarization toward an inflammatory, M1-like state rather than through CD8 T cells. We agree this context is highly relevant for interpreting our findings.

In the revised Discussion, we now cite Rao et al. and explicitly frame our data as consistent with innate immune engagement in our model, while not excluding contributions from T cells. Specifically, in $P65^{f/f}Col1a2^{Cre}$ versus $P65^{f/f}$, our scRNA-seq analyses show increased expression of antigen-presentation-related genes in myeloid populations, including H2-Aa, H2-Ab1, H2-Eb1, Cd74, together with reduced expression of the immunoregulatory marker Arg1 (Response Figure. 12). These transcriptional changes are compatible with a shift toward a more inflammatory, antigen-presenting myeloid state, aligning with the macrophage/microglia-centered mechanism proposed by Rao et al.

We also note as a limitation that our current study is phenotypic/correlative and does not establish the causal effector cell types. Accordingly, we outline future mechanistic experiments (e.g., macrophage/microglia depletion or polarization perturbation alongside CD8 T-cell depletion) to directly test whether innate immune reprogramming is required for the therapeutic effect in our system.

Page 10, Lines 406-415 (**Discussion**): Our scRNA-seq analysis of P65$^{f/f}$Col1a2^{Cre} tumors also revealed increased expression of antigen-presentation and immune-activation programs across immune compartments, including macrophage/microglia populations, consistent with a more inflammatory and antigen-presenting myeloid state. Thus, in addition to effects on exhausted T cells, altered CSF clearance and meningeal drainage may influence antigen availability or APC trafficking, thereby shaping antitumor immunity and response to anti-PD-1 therapy. These interpretations remain correlative and will require dedicated functional studies.

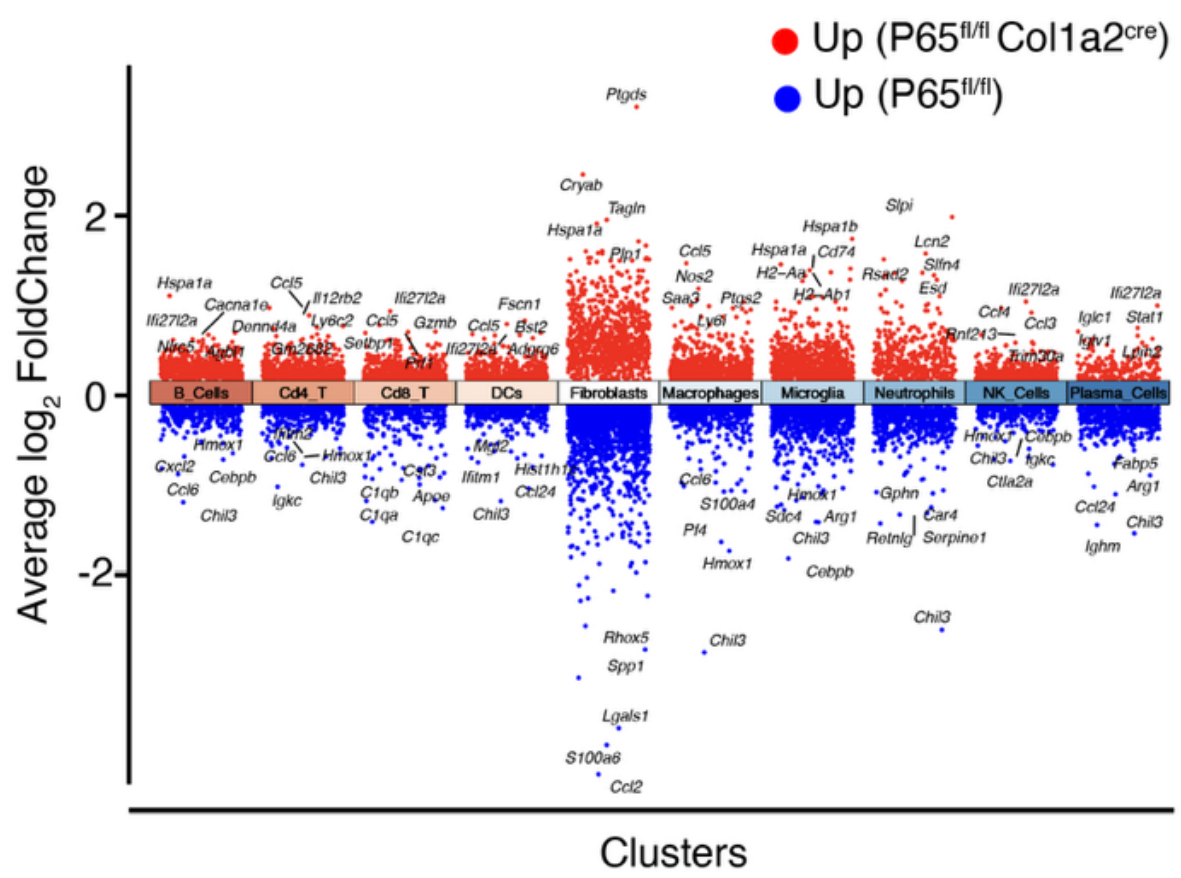

Response Figure. 12 | Gene signatures upregulated in different cell populations on single-cell RNA sequencing data from the P65$^{f/f}$, and P65$^{f/f}$Col1a2^{Cre} groups.

Reference:

1.Rao, G. et al. Anti-PD-1 Induces M1 Polarization in the Glioma Microenvironment and Exerts Therapeutic Efficacy in the Absence of CD8 Cytotoxic T Cells. Clin Cancer Res 26, 4699-4712 (2020).

• Further discussion and integration of the findings with recent single-cell leptomeningeal analyses and studies of meningeal lymphatic modulation would greatly strengthen the manuscript.

Response: We thank the reviewer for this constructive suggestion. In the revised manuscript, **we expanded the Discussion to more explicitly integrate our findings with recent single-cell/single-nucleus transcriptomic atlases of the leptomeninges** and related meningeal niches, **and recent work demonstrating that meningeal/CSF drainage anatomy and meningeal lymphatic function** can shape CSF clearance and downstream immune/homeostatic processes.

First, recent leptomeningeal single-cell/nucleus studies establish the leptomeninges as an immunologically and stromally specialized CNS border with substantial cellular heterogeneity[1,2], including distinct vascular subtypes, multiple fibroblast programs (including arachnoid barrier–associated cells), tissue-resident–like T-cell states, and border-associated macrophage (BAM) populations that are transcriptionally distinct from parenchymal microglia. These data support a framework in which local barrier/ECM programs and CSF-exposed immune interactions (e.g., T-cell-APC crosstalk and BAM/monocyte dynamics) can be regulated at the leptomeninges and may influence CNS immune surveillance in a context-dependent manner.

Second, recent anatomical and functional studies identify conserved leptomeningeal arterio-venous "overlap" regions that can shunt CSF solutes from periarterial to perivenous perivascular spaces and route CSF toward dural efflux pathways[3], highlighting specific control points (e.g., ACE sites) at which impaired outflow can contribute to pathological waste accumulation. Complementing this, studies modulating meningeal lymphatic drainage show that changes in meningeal/dural outflow capacity reshape CSF clearance dynamics and can influence downstream immune and homeostatic states[4]; thus, altered CSF/meningeal drainage in our model could plausibly affect antigen availability and APC-mediated immune responses, although mechanistic experiments will be required to establish causality.

We sincerely thank the reviewer for this constructive suggestion, which helped us strengthen the contextualization of our findings.

Page 10, Lines 412-415 (**Discussion**): Recent leptomeningeal single-cell and single-nucleus atlases further support the view that the leptomeninges are a heterogeneous CNS-border niche containing specialized fibroblast/barrier programs, tissue-resident-like lymphocytes and BAM populations distinct from parenchymal microglia, indicating that immune regulation may occur locally within CSF-exposed meningeal microenvironments.

Reference:

1. Kearns, N. A. et al. Dissecting the human leptomeninges at single-cell resolution. Nature Communications 14, 7036 (2023).

2. Pietilä, R. et al. Molecular anatomy of adult mouse leptomeninges. Neuron 111, 3745-3764. e3747 (2023).

3. Plog, B. A. et al. A route for cerebrospinal fluid flow through leptomeningeal arterial–venous overlaps enables macromolecule and fluid shunting. Nature neuroscience 28, 1436-1445 (2025).

4. Monaghan, K. L. et al. Highly dynamic dural sinuses support meningeal immunity. Nature, 1-10 (2026).

In summary, this study is sound and mechanistically detailed, and its main conclusions are well supported by the data. The primary concerns relate to interpretation and conclusions (e.g., regarding specificity) rather than critical flaws. Revisions should clarify the specificity of fibroblast targeting and address potential clinical confounders, such as tumor subtype and adjuvant steroid therapy that could affect results and/or data interpretation. Further experiments or more cautious language are also

needed for discussing specific CSF factors, and the interpretation of the relationship between ventricular flow velocity and CSF clearance in patients.

Response: We thank the reviewer for the positive and constructive overall assessment. We appreciate the emphasis that the main concerns relate to interpretation (e.g., specificity and clinical confounders) rather than critical flaws, and we have revised the manuscript to improve clarity, rigor, and appropriate caution. Specifically, we have:

1) Clarified fibroblast-targeting specificity by adding additional lineage-tracing analyses and incorporating a Col1a2-specific conditional model (now shown in Fig. 2-3). These data support that Col1a2^{+} perivascular fibroblasts are major contributors to leptomeningeal perivascular fibrosis in our model, and that modulating NF-κB (p65) signaling in these cells reduces fibrosis and is associated with improved CSF tracer clearance and remodeling of the tumor immune microenvironment.

2) Addressed potential clinical confounders by expanding the presentation and Discussion of GBM molecular subtype heterogeneity and glucocorticoid (steroid) exposure as variables that may influence immune states and CSF-related readouts. Where available, we now report steroid exposure metadata (and note limitations in cohort size/annotation that preclude robust subtype-stratified or steroid-stratified inference).

3) Tempered claims about specific CSF factors (e.g., PDGFC, PDGFD, LGALS9) by describing them as CSF-enriched candidate markers/associations and explicitly stating that causal roles will require future mechanistic validation.

4) Refined interpretation of MRI measures by clarifying that ventricular flow velocity reflects CSF dynamics but is not a direct measure of CSF clearance; we therefore restrict conclusions to correlation and note that direct clearance measurements will be needed in future studies.

We are grateful for these insightful suggestions, which have strengthened the specificity, transparency, and clinical interpretability of the revised manuscript.

Reviewer #3 (Remarks to the Author):

The manuscript by Sun, Jiang, Zhang, Fan et al. introduces a concept in which the progression of GBM, a particularly aggressive brain tumor type, is regulated by leptomeningeal fibroblasts. These fibroblasts respond to the tumor, forming fibrosis that alters CSF clearance dynamics. While experimentally compelling, the manuscript in its current form has several conceptual gaps that I recommend addressing.

Response: We sincerely thank the reviewer for the thoughtful and constructive assessment of our manuscript. We agree that several conceptual points required clarification, and we have revised the manuscript accordingly. In response to the reviewer's major concerns, we have also performed additional experiments (including new lineage-tracing analyses) and incorporated these new data and corresponding text revisions to address the identified gaps point-by-point below.

Major comments:

1. Origin of fibrosis: The authors invoke leptomeningeal and perivascular fibrosis several times, interchangeably. These two pathologies are distinct, and they should perform formal lineage tracing to delineate the origin of the fibrotic tissue deposited in response to GBM. This is a critical point for the submitted manuscript. See examples of such tracing in PMID: 33526922.

Response: We thank the reviewer for this important suggestion. We fully agree that leptomeningeal fibrosis and parenchymal perivascular fibrosis represent distinct pathological processes, and **we have revised the manuscript to avoid using these terms interchangeably and to explicitly define "leptomeningeal perivascular fibrosis" as collagen deposition at the leptomeningeal vascular interface**.

To formally delineate the cellular origin of the fibrotic tissue induced by GBM, **we performed adult inducible genetic lineage tracing, guided by the approach in PMID: 33526922**. Specifically, we conducted fate mapping using Col1a2-CreERT2; Rosa26-LSL-tdTomato and NG2(Cspg4)-CreERT2; Rosa26-LSL-tdTomato mice (new data in Fig. 2a-c and Response Figure. 13). In GBM-bearing mice, we observed a ~3.9-fold increase in Col1a2-lineage tdTomato^{+} cells at the leptomeningeal interface, and these cells co-localized with COL1^{+} fibrotic regions. In contrast, NG2-lineage traced cells did not show a comparable expansion in these leptomeningeal collagen-rich areas. These findings indicate that Col1a2-lineage leptomeningeal fibroblasts constitute the predominant expanding stromal population associated with GBM-induced leptomeningeal perivascular collagen deposition, and argue against a major contribution from NG2-lineage mural/pericyte cells under our experimental conditions.

We further note that these fibrotic cells are spatially restricted to the leptomeningeal surface/vascular interface rather than deep parenchymal vessels or the choroid plexus, supporting the conclusion that the fibrosis we quantify in this study is leptomeningeal in origin.

<u>Page 3, Lines 100-109 (**Results**): In the CNS, collagen-producing stromal cells implicated in fibrosis have been reported to include COL1-expressing fibroblasts and pericytes. To assess the lineage</u>

contribution to GBM-associated leptomeningeal perivascular collagen deposition, we performed genetic fate mapping using Col1a2^{CreERT2}; Rosa26-LSL-tdTomato and NG2(Cspg4)^{CreERT2}; Rosa26-LSL-tdTomato mice (Fig. 2a). In GBM-bearing mice, we observed an approximately 3.9-fold increase in Col1a2^{CreERT2}-labelled cells at the leptomeningeal interface that co-localized with COL1+ fibrotic regions, whereas NG2^{CreERT2}-traced cells did not show a comparable increase (Fig. 2b,c). These results suggest that Col1a2-lineage fibroblasts represent a major expanding stromal population associated with leptomeningeal perivascular collagen deposition in this model, whereas contribution from NG2+ mural cells appears comparatively limited.

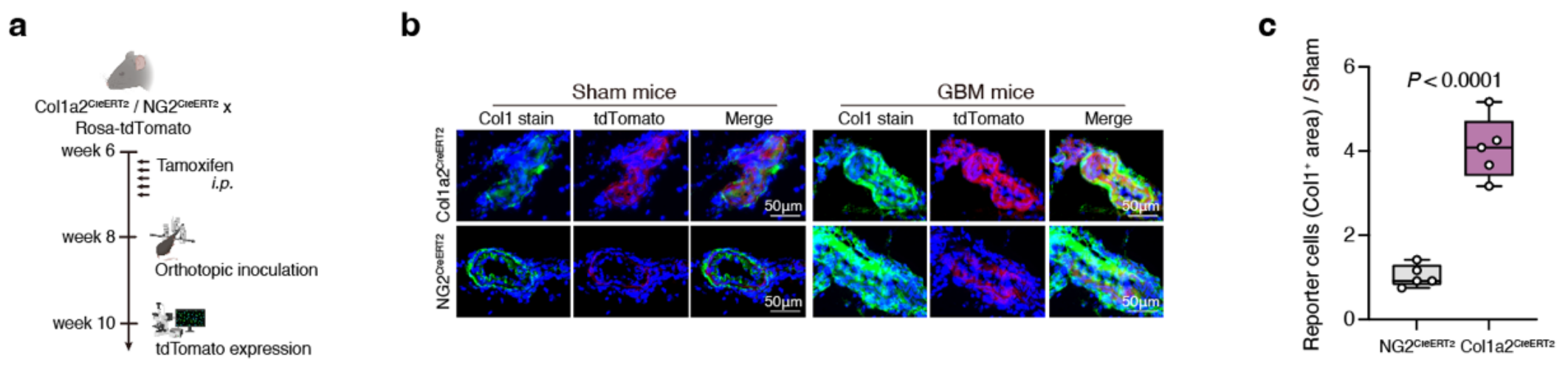

Response Figure. 13 | a, Schematic of inducible genetic fate mapping used to trace COL1-lineage fibroblasts (Col1a2^{CreERT2}; Rosa26-LSL-tdTomato) and NG2-lineage mural cells (NG2^{CreERT2}; Rosa26-LSL-tdTomato) in mouse leptomeninges under sham and GBM conditions. **b,** Representative images of the leptomeningeal interface showing tdTomato lineage-labelled cells and COL1 immunostaining in sham-operated and GBM tumour-bearing mice. **c,** Quantification of reporter cells at the leptomeningeal interface, normalized to sham-operated controls (mean ± s.d., n = 5 mice per group).

2. Critical fibrosis mediator: The authors use GBM-derived CSF, human or mouse, with a somewhat delineated composition, to induce the phenotype in most experiments. They call out several proteins that can be responsible for this pathophysiological behavior, such as PDGFs or galectins. Which of these (or which minimal combination and at what concentration) mediates this phenotypic switch in vitro and in vivo? Also, the authors refer to these factors as "aberrant substances", which is not necessarily true, as they have clear roles in physiology. Better phrasing, such as tumor-derived (if correct), would be appreciated.

Response: We thank the reviewer for this important mechanistic question and for the suggestion regarding terminology. **We agree that, in a GBM-CSF–driven system, identifying causal mediators, and ultimately defining a minimal sufficient combination and effective concentration ranges in vitro and in vivo, would further strengthen the study.**

Our study was designed to test GBM-associated CSF as an integrated tumor-associated fluid microenvironment and to establish its functional impact on leptomeningeal fibroblast activation and fibrosis-like responses. Consistent with this framework, our proteomic profiling revealed enrichment of several candidate ligand families/pathways, including PDGF-related ligands and galectin family members. **To provide functional evidence for PDGF pathway involvement, we performed additional perturbation experiments** targeting PDGFRβ using CP-673451 (200 nM, 12 h) in

leptomeningeal fibroblasts. PDGFRβ inhibition significantly attenuated the gCSF-induced profibrotic activation phenotype, reducing the αSMA/Col1a1 double-positive population from 52.2517 to 25.4867 (approximately 51.2% reduction) (Response Figure. 14). These data support that PDGFRβ signaling contributes, at least in part, to the CSF-driven phenotypic switch.

We acknowledge that our current data do not yet establish a single sufficient molecule or a minimal recombinant factor combination with defined dose ranges that fully recapitulates the phenotype. We have explicitly stated this limitation and outlined follow-up experiments (recombinant add-back across physiologic/pathologic ranges, immunodepletion/neutralization, and in vivo perturbation/validation) in the Discussion.

Regarding wording, we replaced "aberrant substances" with more accurate phrasing and clarified that PDGFs, galectins, and related factors have established physiological roles; our emphasis is on their enrichment and potential pathological activity in the tumor-associated CSF context.

We thank the reviewer for the constructive feedback, which improved the clarity and rigor of the revised manuscript.

Page 9-10, Lines 384-394 (**Discussion**): Nevertheless, although PDGFC, PDGFD and LGALS9 are enriched in gCSF and PDGFRβ is expressed on perivascular fibroblasts, these data remain correlative. Establishing a causal PDGF-PDGFRβ- or LGALS9-dependent mechanism will require targeted perturbation studies. More broadly, the CSF-induced fibroblast phenotype is likely driven by the synergy of multiple CSF-borne factors rather than by a single dominant mediator......While our current study highlights the central role of secretory proteins, the contribution of other CSF metabolites to fibroblast activation and leptomeningeal remodeling warrants further investigation.

Page 5, Lines 163-165 (**Results**): Collectively, these data indicate that gCSF contains bioactive molecules capable of promoting profibrotic fibroblast activation, and nominate PDGFC, PDGFD and LGALS9 as candidate mediators of this response.

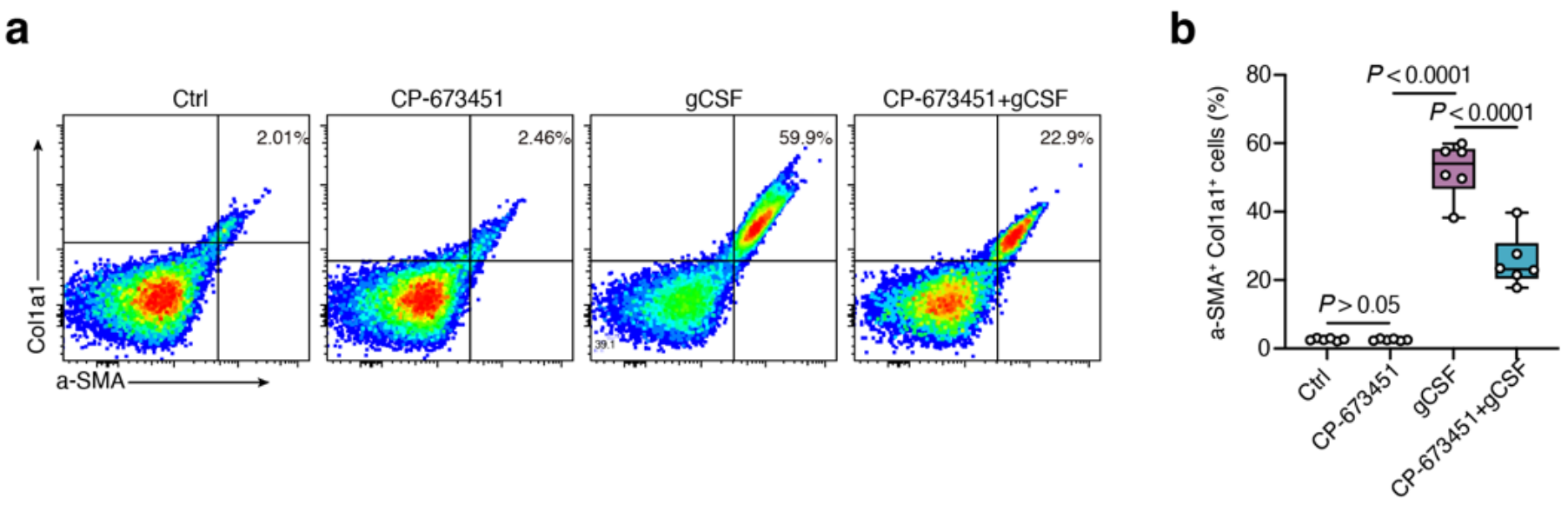

Response Figure. 14 | a, Representative flow cytometry plots showing αSMA and Col1a1 staining in leptomeningeal fibroblasts treated with control medium (Ctrl), CP-673451 (200 nM), GBM-derived CSF (gCSF), or gCSF+CP-673451. **b,** Quantification of $\alpha SMA^+Col1a1^+$ cells (mean ± s.d., n = 6).

3. Sham controls: What is the situation regarding clearance and fibrosis in non-tumor-bearing mice that underwent surgery?

Response: We thank the reviewer for this important suggestion regarding sham controls. We agree that the impact of surgery itself must be defined when interpreting tumor-associated clearance impairment and leptomeningeal fibrosis. Therefore, **we added a sham cohort and compared it with non-operated controls (Ctrl) at the same post-surgical time point used for the tumor-bearing group.** Sham mice underwent identical anesthesia, stereotaxic positioning, craniotomy, and needle insertion, and received an equal volume of sterile saline at the same coordinates (without tumor cells).

COL1A1/COL3A1 immunostaining showed no increase in leptomeningeal collagen deposition in sham mice compared with Ctrl (Response Figure. 15a-b). In the gadobutrol clearance assay, sham mice showed no significant difference compared with Ctrl (Response Figure. 15c).

Together, these data indicate that the surgical procedure alone is insufficient to reproduce the leptomeningeal fibrosis and CSF clearance deficit observed in tumor-bearing mice, supporting a tumor-associated mechanism..

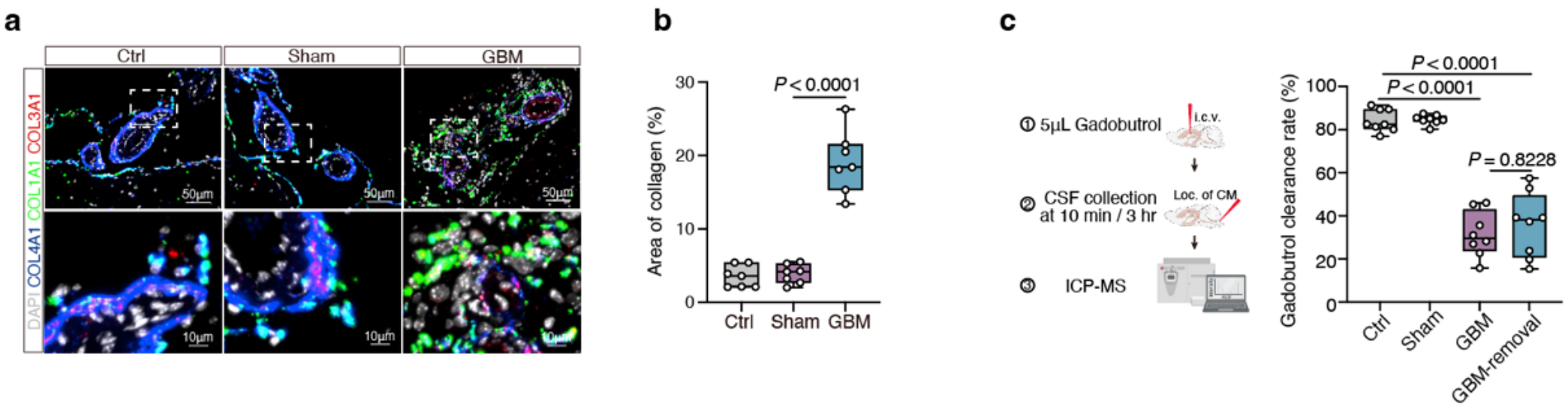

Response Figure. 15 | a, Representative images of perivascular spaces (COL4A1$^+$) and type I/III collagen fibres (COL1A1$^+$/COL3A1$^+$) in the leptomeninges of control mice, sham-operated mice and GBM tumour-bearing mice. Scale bar, 50 µm; zoom-in, 10 µm. **b,** Quantification of leptomeningeal type I/III collagen fibre area (COL1A1$^+$/COL3A1$^+$) in control mice, sham-operated mice and GBM tumour-bearing mice (mean ± s.d., n = 7 mice per group). **c,** CSF clearance assay in the cisterna magna region following intraventricular injection of gadobutrol in sham mice, GBM tumour-bearing mice and tumour-resected GBM mice. Left, schematic of intraventricular gadobutrol injection and CSF collection. Right, quantification of CSF clearance within 3 h after injection (mean ± s.d., n = 8 mice per group). Figure created with BioRender.com.

4. Core drivers of fibrosis: The authors suggest that fibroblasts are the core drivers of fibrosis. While technically correct, this process is instructed and orchestrated by the immune cells. Is this phenotype reproducible in immunocompromised (i.e., NSG) animals? And human xenografts? How do resident immune cell types, such as macrophages, contribute to or regulate this process?

Response: We thank the reviewer for raising this important point. We agree that, while fibroblasts are the principal ECM-producing effector cells in fibrosis, their activation can be instructed and modulated by immune cells in the meningeal niche[1,2].

To address whether the phenotype depends on an intact immune system, we performed additional experiments in immunocompromised NSG mice. NSG mice were intracranially implanted with our CT3-GBM cells[3], and leptomeningeal perivascular fibrosis and CSF tracer clearance were assessed at the same time point used in our immunocompetent tumor-bearing cohort. We observed a significant increase in leptomeningeal perivascular collagen deposition (COL1A1/COL3A1) and impaired gadobutrol clearance in CT3-bearing NSG mice compared with controls (Response Figure. 16a-b).

These data indicate that the **GBM-associated leptomeningeal fibrosis and clearance impairment can occur in the absence of functional adaptive immunity (T/B cells) and NK cells**, suggesting that tumor-associated factors can directly engage fibroblast activation programs. Importantly, NSG mice retain innate immune populations (including macrophage-lineage cells), and we therefore do not exclude a modulatory role for resident meningeal macrophages/microglia in shaping the magnitude and dynamics of the response. We have clarified this point and we will investigate to highlight future directions, including targeted perturbation of macrophage signaling/abundance and single-cell profiling of immune-fibroblast crosstalk.

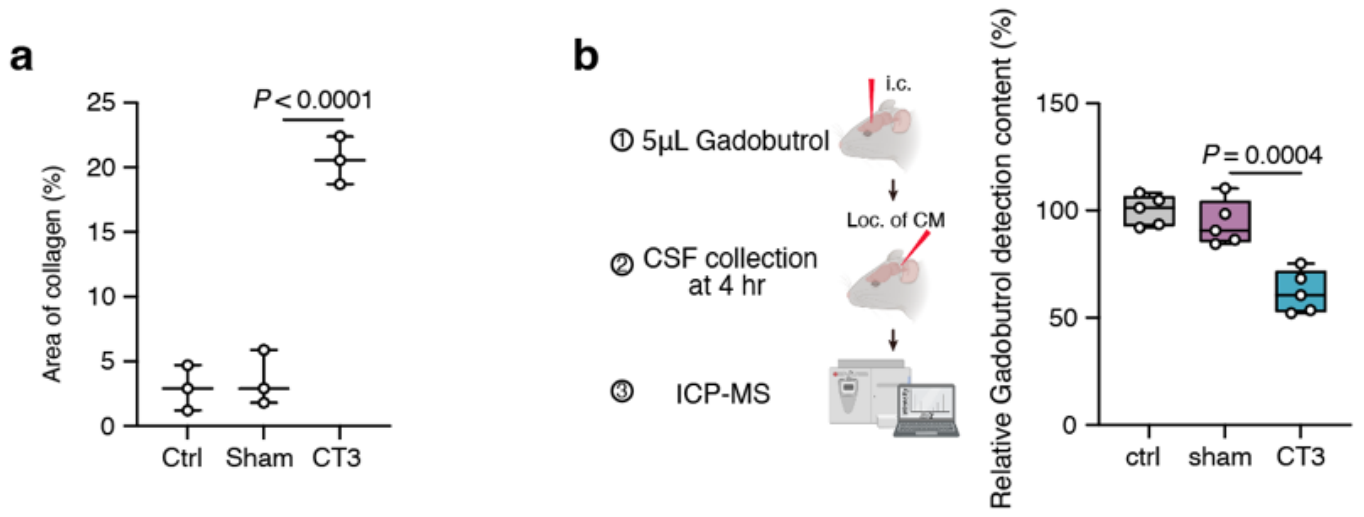

Response Figure. 16 | a, Quantification of leptomeningeal type I/III collagen fibre area ($COL1A1^{+}$/$COL3A1^{+}$) in control mice, sham-operated mice and GBM tumour-bearing mice (mean ± s.d., n = 3 mice per group). **c,** Left, schematic of intraparenchymal gadobutrol injection and cisterna magna CSF collection. Right, relative abundance of gadobutrol in cisterna magna CSF measured 3 h after intraparenchymal tracer injection (mean ± s.d., n = 5 mice per group). Figure created with BioRender.com.

Reference:
1. DeSisto, J. et al. Single-cell transcriptomic analyses of the developing meninges reveal meningeal fibroblast diversity and function. Developmental cell 54, 43-59. e44 (2020).
2. Dorrier, C. E., Jones, H. E., Pintarić, L., Siegenthaler, J. A. & Daneman, R. Emerging roles for CNS fibroblasts in health, injury and disease. Nature Reviews Neuroscience 23, 23-34 (2022).
3. Jiang, S. et al. Interleukin-8-induced tumor self-rampart spatially confines oncolytic virotherapy in glioblastoma. Neuro-Oncology (2025). https://doi.org:10.1093/neuonc/noaf276

5. GBM models: This brings me to another vital issue – the choice of the correct model. Both model lines used in this study elicit a severe immune response, atypical for human GBM. Is this phenomenon reproducible in less immunogenic models? Such as xenografts or other murine lines, as asked above.

Response: We thank the reviewer for highlighting the important issue of model immunogenicity. We agree that some commonly used murine GBM models can elicit strong immune responses that may not fully reflect the immune landscape of human GBM.

To address reproducibility in a less immunogenic setting, **we performed additional experiments using *SB28 cell*, a reported less immunogenic murine GBM model.** In this model, we again observed increased leptomeningeal perivascular collagen deposition (COL1A1/COL3A1) and impaired gadobutrol clearance compared with controls (Response Figure. 17a-b).

Together with the data from immunocompromised NSG mice (Response Figure. 15a-b), these findings support that the leptomeningeal fibrosis and clearance impairment are not restricted to highly immunogenic models and are not solely a byproduct of an exaggerated anti-tumor immune response. **We have clarified that additional meningeal-resident cell types, including immune and vascular-associated populations, may also contribute to CSF-related pathology and warrant further investigation.**

Page 10, Lines 424-426 (**Discussion**): In addition, although this study focuses on leptomeningeal fibroblasts, other meningeal-resident cell types, including immune, vascular-associated and meningeal epithelial-like cells, may also sense CSF-derived signals and contribute to CSF-related pathology.

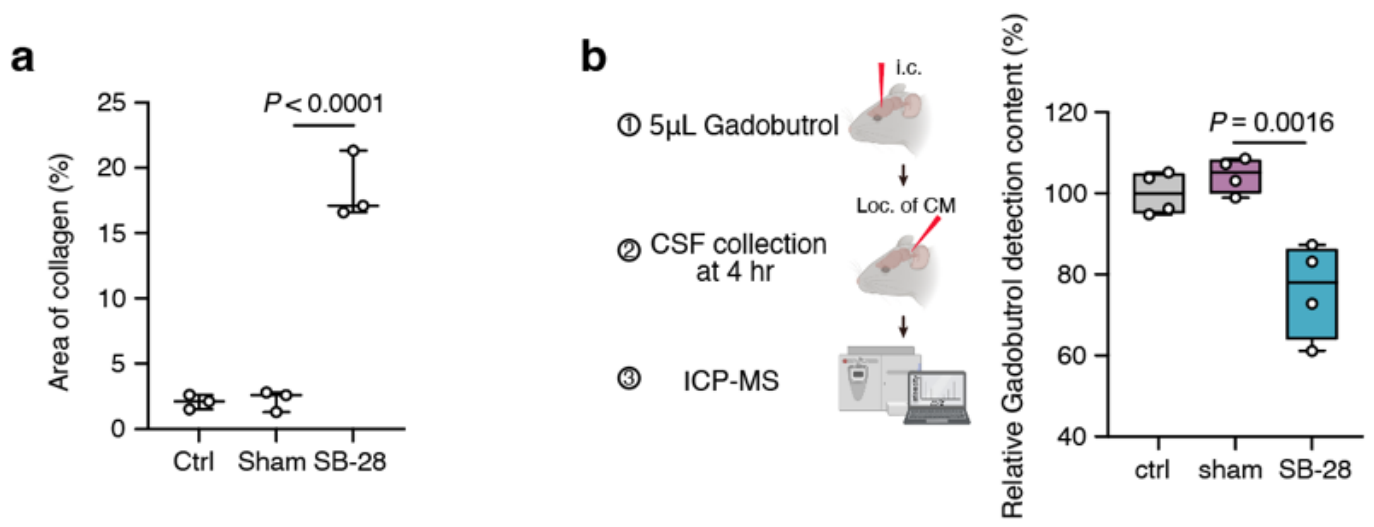

Response Figure. 17 | a, Quantification of leptomeningeal type I/III collagen fibre area ($COL1A1^+/COL3A1^+$) in control mice, sham-operated mice and GBM tumour-bearing mice (mean ± s.d., n = 3 mice per group). **c,** Left, schematic of intraparenchymal gadobutrol injection and cisterna magna CSF collection. Right, relative abundance of gadobutrol in cisterna magna CSF measured 3 h after intraparenchymal tracer injection (mean ± s.d., n = 5 mice per group). Figure created with BioRender.com.

6. "Pathological fibroblasts" and single-cell analysis: What evidence do the authors have for CTHRC1/SMA fibroblasts to be pathological, besides their position in UMAP? UMAP does not allow quantitative measurements. If these are assumptions, they should be experimentally proven (i.e., by CTHRC1/SMA fibroblast-lineage depletion). In addition, the single-cell analyses do not meet contemporary standards. Cell type annotations are insufficient, methods do not allow full reproducibility, filtering steps are unclear, quality metrics are not provided, the number of retained cells cannot be found…

Response： We thank the reviewer for this important critique. **We agree that UMAP location alone is insufficient to label a fibroblast state as "pathological," and our original single-cell reporting lacked several elements expected for contemporary scRNA-seq standards. We have therefore revised both the language and the analyses** to improve rigor, quantification, and reproducibility.

1) "Pathological fibroblasts" (CTHRC1/SMA) - interpretation and evidence.
We now avoid defining CTHRC1/ACTA2(SMA) fibroblasts as "pathological" based solely on marker positivity or UMAP positioning. Throughout the revised manuscript, we refer to this population as an ECM-remodeling/myofibroblast-like fibroblast state and restrict our conclusions to what is supported by quantitative transcriptomic evidence. **Specifically, within the fibroblast compartment we identify three major subsets (clusters 7/8/21; Fig. 2d-e and Response Figure. 18d-e)** that (i) express canonical fibroblast/ECM genes (e.g., COL1A1/COL1A2) and (ii) lack pericyte markers such as CSPG4 (NG2), distinguishing them from mural cells. **We further perform gene-module scoring** (collagen fibril organization and collagen-containing extracellular matrix) and show a graded increase across the inferred fibroblast state transition, consistent with a fibrotic activation trajectory; importantly, these fibrosis-associated scores are consistently higher in GBM leptomeninges than controls (Fig. 2f-g and Response Figure. 18f-g). We also **include a pseudotime analysis** (Monocle2) as an orthogonal, quantitative approach to support state relationships, while explicitly noting that pseudotime infers transcriptional ordering rather than proving causality. These results have been incorporated into the revised manuscript text

2) scRNA-seq standards and reproducibility.
We have reanalyzed the dataset and substantially expanded the Methods and Extended Data to ensure full reproducibility. We now report: (i) the number of biological replicates; (ii) raw and post-QC cell counts for each sample and each cluster; (iii) QC metrics (nUMI, nFeature, and percent.mt) together with the applied thresholds and their rationale; (iv) complete preprocessing, integration, and clustering parameters (normalization, HVGs, number of PCs used, clustering resolution, and UMAP settings); (v) marker-based cell-type annotations supported by feature plots; and (vi) the settings used for differential expression and enrichment analyses. These additions are described in the revised Methods.

We sincerely appreciate the reviewer's guidance, and we believe the revisions described above address these concerns and meaningfully improve the manuscript.

Page 3-4, Lines 110-132 (**Results**): To define the cellular programs associated with GBM-related fibroblast activation, we performed single-cell RNA sequencing on leptomeningeal samples from control individuals and GBM patients. Across 26 clusters, we annotated four major putative compartments, fibroblasts, mural cells (including pericytes), immune cells and endothelial cells, based on canonical markers (Extended Data Fig. 2a-c). Fibroblasts were primarily distributed across clusters 7, 8 and 21 (Fig. 2d). All three clusters expressed fibroblast-associated markers, including COL1A1, COL1A2 and DCN, and lacked the canonical mural-cell marker CSPG4 (NG2) (Fig. 2e). Notably, cluster 7 was enriched for COL1A1 and PDGFRB, whereas cluster 8 preferentially expressed COL1A1 and PDGFRA and showed higher DCN expression (Fig. 2e), suggesting heterogeneous fibroblast-like stromal states with differential PDGFRB and PDGFRA expression that

may be differentially engaged in GBM. We next used Monocle 2 to infer relationships among fibroblast-like states across the three subclusters, identifying three pseudotime-associated states. State 3, composed largely of cluster 7 cells, exhibited a PDGFRB$^+$ PDGFRA$^-$ FAP$^-$ profile, consistent with a less activated fibroblast-like state located near the beginning of the inferred trajectory (Fig. 2e,f). Across the inferred transition from state 3 to state 1, gene set scores for COLLAGEN_FIBRIL_ORGANIZATION and COLLAGEN_CONTAINING_EXTRACELLULAR_MATRIX progressively increased, consistent with acquisition of a more fibrogenic program (Fig. 2g). Importantly, these fibrosis-related scores were consistently higher in the GBM group than in controls (Fig. 2g and Extended Data Fig. 2d), indicating enhanced fibrogenic activation of leptomeningeal fibroblast-like cells in GBM. A COLLAGEN_BIOSYNTHETIC_PROCESS gene set further highlighted clusters 7 and 8 as the fibroblast-enriched populations with the strongest collagen biosynthetic signatures within this inferred fibrotic program (Fig. 2h), whereas cluster 21 showed substantially weaker enrichment of collagen biosynthetic pathways.

Page 15-16, Lines 621-670 (**Methods**): Leptomeningeal tissue was obtained from 3 non-tumor controls and 3 GBM patients (biological replicates), under institutional ethics approval and written informed consent. Fresh tissue was washed in ice-cold PBS and enzymatically dissociated using a commercial tissue digestion buffer (BioGenous, K601008) at 37 °C for 15 min with gentle agitation. The cell suspension (supernatant and remaining tissue fraction) was pooled and filtered through a 70-µm strainer (BD Falcon, 352350), centrifuged at 500 × g for 5 min, and subjected to an additional digestion step in 1 mL trypsin for 15 min. All fractions were pooled, filtered through a 35-µm strainer (BD Falcon, 352235), centrifuged at 500 × g for 5 min, and resuspended in PBS + 0.01% BSA. Cells were processed on the 10x Genomics Chromium Next GEM platform, and 3′ scRNA-seq libraries were generated using the Chromium Next GEM Single Cell 3′ Kit v3.1 according to the manufacturer's instructions. Libraries were sequenced on an Illumina NovaSeq 6000 using paired-end 150-bp reads. Base call files were processed using Cell Ranger (v9.0.1) and aligned to the human reference genome (GRCh38; Cell Ranger reference), generating a filtered feature-barcode matrix for each sample. Downstream analysis was performed in R (v4.3.0) using Seurat (v4.4.4) unless noted. Putative doublets were identified per sample using DoubletFinder (parameters and expected doublet rate reported in Supplementary Methods) and removed prior to integration. Cells were excluded if they met any of the following criteria: (i) detected genes (nFeature_RNA) outside median ± 3 SD (computed within each sample), or (ii) high mitochondrial transcript fraction (percent.mt > 30%). Data were log-normalized (Seurat NormalizeData, default) and scaled (ScaleData). Highly variable genes were selected using FindVariableFeatures (top 3,000 HVGs). Patient-level batch effects were corrected using Harmony. The Harmony-corrected embedding was used for downstream dimensionality reduction, clustering, and visualization. Principal component analysis (PCA) was performed on HVGs. The top 30 PCs were used to build the shared nearest-neighbor graph (FindNeighbors) and to identify clusters (FindClusters, resolution 0.6). UMAP was computed for visualization (RunUMAP; parameters provided in Supplementary Methods). We emphasize that UMAP was used for visualization only; quantitative comparisons are based on differential expression and gene-set/module scoring. Major cell classes were annotated using canonical marker genes and supported by dot plots. Markers included: endothelial cells (PECAM1/CD31, VWF, CLDN5), fibroblasts (COL1A1, COL1A2, PDGFRA), mural cells (ACTA2,

TAGLN, MYH11, CNN1; COL4A1), and immune cells (PTPRC). Clusters lacking clear lineage markers were annotated as "other". To increase resolution within fibroblasts, fibroblast-annotated cells were subset and reprocessed using the same workflow. Fibroblast subclusters were identified at resolution 0.1 and annotated using a curated marker panel including ECM genes (e.g., COL1A1/COL1A2, LUM, DCN), activation markers (e.g., FAP, CTHRC1), and smooth muscle/myofibroblast-associated markers (e.g., ACTA2), while excluding pericyte-dominant signatures (e.g., CSPG4/NG2). Differentially expressed genes were identified using Seurat FindAllMarkers with thresholds adjusted $P < 0.05$ and avg_log2FC > 0.25. GO/KEGG enrichment and GSEA were performed using clusterProfiler (v4.10.1), using all detected genes as background. Enrichment significance was defined as nominal $P < 0.05$ and FDR (q) < 0.05. Gene sets were obtained from MSigDB (https://www.gsea-msigdb.org). Fibrosis/ECM module scores (e.g., COLLAGEN_FIBRIL_ORGANIZATION, COLLAGEN_CONTAINING_EXTRACELLULAR_MATRIX) were calculated using Seurat AddModuleScore and compared across fibroblast subsets and conditions. These quantitative scores were used to support statements regarding ECM-remodeling/fibrotic activation states. Fibroblast differentiation trajectories were reconstructed using Monocle2 (v2.30.0). To balance cluster representation, up to 1,000 cells per fibroblast subcluster were randomly retained. Genes used for ordering were selected by differentialGeneTest (q-value < 0.01), excluding mitochondrial, ribosomal, and hemoglobin genes. Dimensionality reduction was performed with DDRTree, and trajectories were visualized using plot_cell_trajectory. We note that pseudotime analyses infer transcriptional state relationships and do not establish causality.

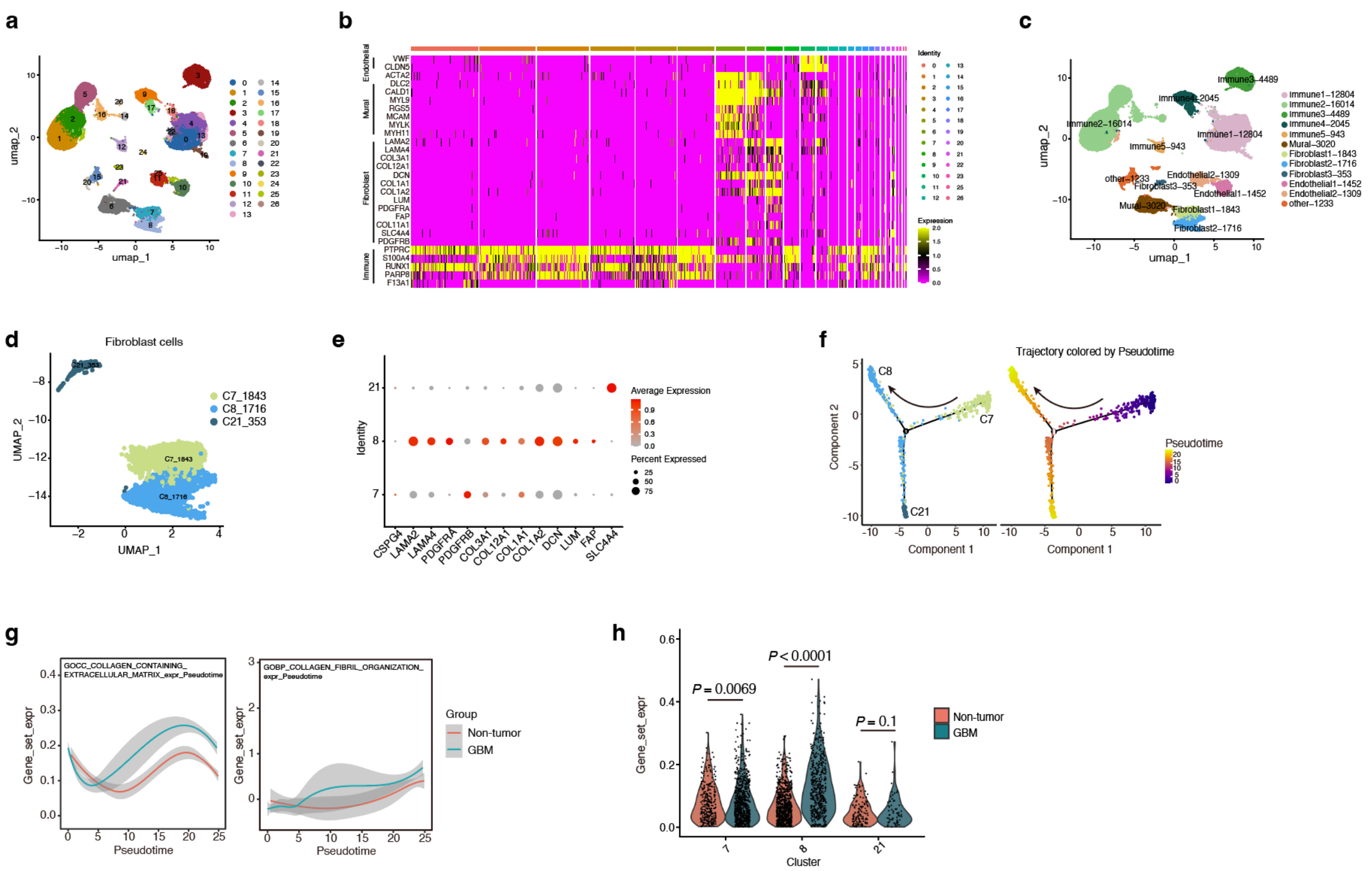

Response Figure. 18 | a, UMAP of single-cell transcriptomes from leptomeningeal tissues of non-tumour controls (n = 3) and patients with GBM (n = 3). **b,** Heat map showing expression of canonical marker genes used for annotation of major cell types. **c,** UMAP of single-cell transcriptomes from leptomeningeal tissues of non-tumour controls (n = 3) and patients with GBM (n = 3), with clusters annotated into distinct cell populations based on canonical marker gene expression. **d,** UMAP embedding of single-cell transcriptomes from leptomeningeal tissues of non-tumour controls and patients with GBM (n = 3 samples per group), highlighting fibroblast clusters. **e,** Feature plots showing expression of representative fibroblast marker genes in leptomeningeal single-cell transcriptomes from non-tumour controls and patients with GBM. **f,** Monocle 2 pseudotime trajectory of fibroblast subclusters (clusters 7, 8 and 21), with inferred transcriptional states. **g,** Gene set scores along pseudotime for *COLLAGEN_FIBRIL_ORGANIZATION* and *COLLAGEN_CONTAINING_EXTRACELLULAR_MATRIX* in control and GBM groups. **h,** Gene set scores for *COLLAGEN_BIOSYNTHETIC_PROCESS* across fibroblast subclusters.

7. Cre reporter: What is the reasoning behind the Cre reporter choice? Tcf21 has been described to be expressed in other types of fibroblasts (fibroblasts in choroid plexus and parenchymal perivascular fibroblasts), not leptomeningeal fibroblasts. How do the authors explain their findings, considering this article PMID: 37776854? This suggests that these fibrotic lesions may not be of leptomeningeal origin. Again, tracing here is necessary.

Response: We thank the reviewer for the thoughtful critique regarding Cre-reporter specificity and cellular origin. **We fully agree that Tcf21 is not a leptomeningeal-fibroblast-specific marker, and recent work (PMID: 37776854) highlights its expression across multiple CNS fibroblast populations (including choroid plexus and parenchymal perivascular fibroblasts).** Accordingly, we have revised the manuscript to avoid using Tcf21-Cre alone to assign the origin of the fibrotic lesions.

To directly address the reviewer's concern and the need for lineage tracing, we conducted fate mapping using Col1a2-CreERT2; Rosa26-LSL-tdTomato and NG2(Cspg4)-CreERT2; Rosa26-LSL-tdTomato mice (new data in Fig. 2a-c and Response Fig. 19a). In GBM-bearing mice, we observed a ~3.9-fold increase in Col1a2-lineage tdTomato+ cells at the leptomeningeal interface, and these cells co-localized with COL1^{+} fibrotic regions. In contrast, NG2-lineage traced cells did not show a comparable expansion in these leptomeningeal collagen-rich areas (Response Fig. 19b-c). These data support that Col1a2-lineage stromal fibroblasts represent the predominant expanding population associated with GBM-induced leptomeningeal perivascular collagen deposition under our experimental conditions, and argue against a major contribution from NG2-lineage mural/pericyte cells.

In addition, to further reduce reliance on Tcf21-Cre specificity, **we complemented the Tcf21-Cre model with an independent Col1a2-targeted approach** (Col1a2-AAV-Cre-based conditional p65 suppression (Response Fig. 19d). Using multiplex immunofluorescence, we confirmed that the Col1a2-targeted recombination signal co-localizes with fibroblast-associated markers and does not overlap with markers of mural cells, endothelial cells, or immune cells in the analyzed compartment (Response Fig. 19e). Functionally, inhibiting NF-κB signaling within Col1a2^{+} cells attenuated

leptomeningeal fibrosis and improved CSF clearance (Response Fig. 19f-g) and downstream immune phenotypes (Response Fig. 19h-m), consistent with our central conclusions.

We note that AAV-Cre delivery has inherent limitations in cell-type specificity, particularly with CSF administration. Therefore, in the revised manuscript, the AAV-Cre experiments are used as a functional perturbation strategy and not as evidence to define cell identity or developmental origin. We thank the reviewer again; these revisions and new lineage-tracing data substantially strengthen the rigor of our conclusions regarding stromal cell sources and Cre-reporter specificity.

Page 3, Lines 110-109 (**Results**): In the CNS, collagen-producing stromal cells implicated in fibrosis have been reported to include COL1-expressing fibroblasts and pericytes. To assess the lineage contribution to GBM-associated leptomeningeal perivascular collagen deposition, we performed genetic fate mapping using Col1a2CreERT2; Rosa26-LSL-tdTomato and NG2(Cspg4)CreERT2; Rosa26-LSL-tdTomato mice (Fig. 2a). In GBM-bearing mice, we observed an approximately 3.9-fold increase in Col1a2CreERT2-labelled cells at the leptomeningeal interface that co-localized with COL1+ fibrotic regions, whereas NG2CreERT2-traced cells did not show a comparable increase (Fig. 2b,c). These results suggest that Col1a2-lineage fibroblasts represent a major expanding stromal population associated with leptomeningeal perivascular collagen deposition in this model, whereas contribution from NG2+ mural cells appears comparatively limited.

Page 3-4, Lines 110-132 (**Results**): To define the cellular programs associated with GBM-related fibroblast activation, we performed single-cell RNA sequencing on leptomeningeal samples from control individuals and GBM patients. Across 26 clusters, we annotated four major putative compartments, fibroblasts, mural cells (including pericytes), immune cells and endothelial cells, based on canonical markers (Extended Data Fig. 2a-c). Fibroblasts were primarily distributed across clusters 7, 8 and 21 (Fig. 2d). All three clusters expressed fibroblast-associated markers, including COL1A1, COL1A2 and DCN, and lacked the canonical mural-cell marker CSPG4 (NG2) (Fig. 2e). Notably, cluster 7 was enriched for COL1A1 and PDGFRB, whereas cluster 8 preferentially expressed COL1A1 and PDGFRA and showed higher DCN expression (Fig. 2e), suggesting heterogeneous fibroblast-like stromal states with differential PDGFRB and PDGFRA expression that may be differentially engaged in GBM. We next used Monocle 2 to infer relationships among fibroblast-like states across the three subclusters, identifying three pseudotime-associated states. State 3, composed largely of cluster 7 cells, exhibited a PDGFRB^{+} PDGFRA^{-} FAP^{-} profile, consistent with a less activated fibroblast-like state located near the beginning of the inferred trajectory (Fig. 2e,f). Across the inferred transition from state 3 to state 1, gene set scores for COLLAGEN_FIBRIL_ORGANIZATION and COLLAGEN_CONTAINING_EXTRACELLULAR_MATRIX progressively increased, consistent with acquisition of a more fibrogenic program (Fig. 2g). Importantly, these fibrosis-related scores were consistently higher in the GBM group than in controls (Fig. 2g and Extended Data Fig. 2d), indicating enhanced fibrogenic activation of leptomeningeal fibroblast-like cells in GBM. A COLLAGEN_BIOSYNTHETIC_PROCESS gene set further highlighted clusters 7 and 8 as the fibroblast-enriched populations with the strongest collagen biosynthetic signatures within this inferred fibrotic program (Fig. 2h), whereas cluster 21 showed substantially weaker enrichment of collagen biosynthetic pathways.

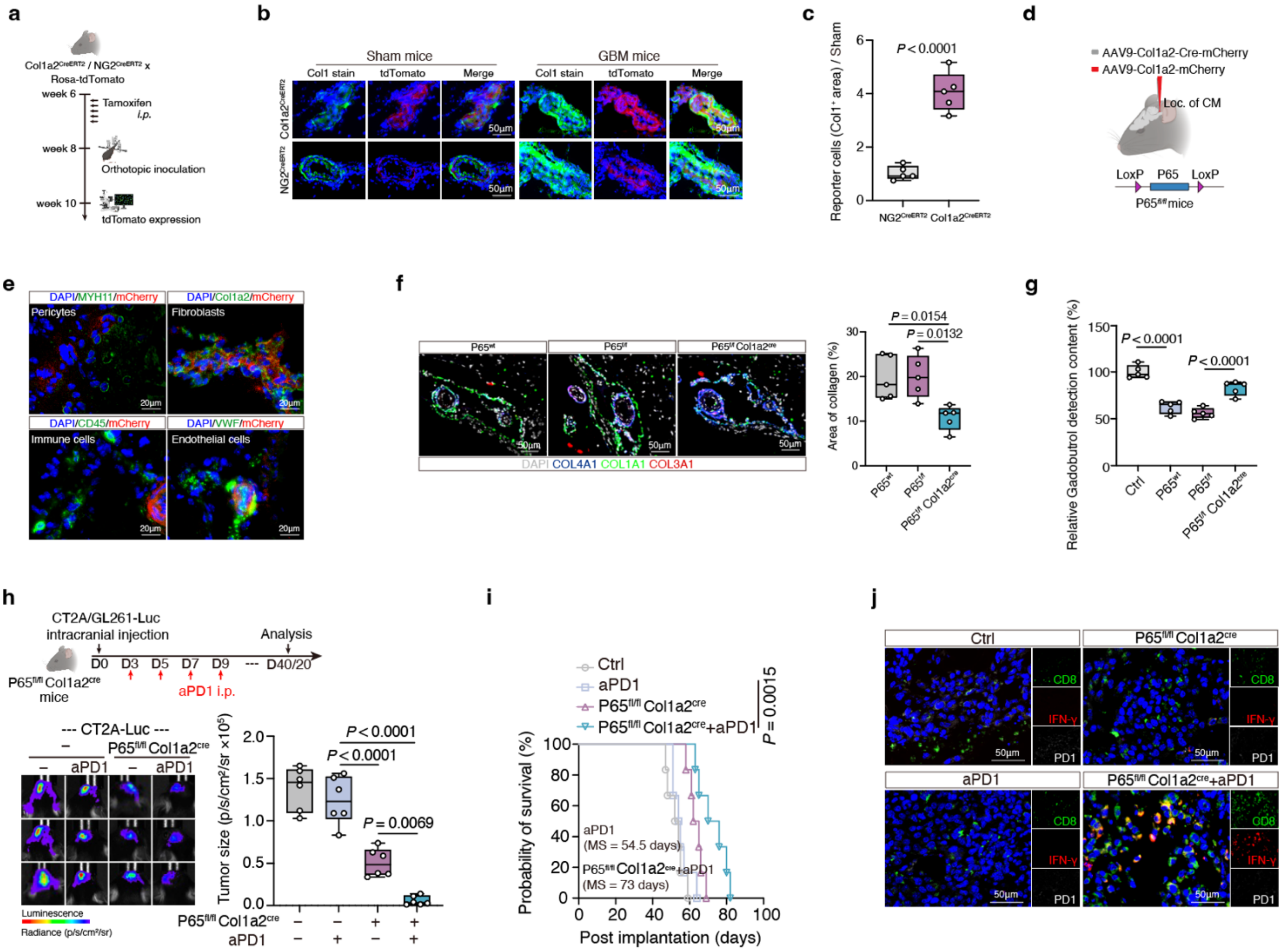

Response Figure. 19 | a, Schematic of inducible genetic fate mapping used to trace COL1-lineage fibroblasts (Col1a2^{CreERT2}; Rosa26-LSL-tdTomato) and NG2-lineage mural cells (NG2^{CreERT2}; Rosa26-LSL-tdTomato) in mouse leptomeninges under sham and GBM conditions. **b,** Representative images of the leptomeningeal interface showing tdTomato lineage-labelled cells and COL1 immunostaining in sham-operated and GBM tumour-bearing mice. **c,** Quantification of reporter cells at the leptomeningeal interface, normalized to sham-operated controls (mean ± s.d., n = 5 mice per group). **d,** Schematic of the strategy used for conditional deletion of P65 in Col1a2-targeted leptomeningeal fibroblasts. AAV9 vectors expressing Cre-mCherry under the Col1a2 promoter (pAAV9-Col1a2-Cre-mCherry) or control mCherry vectors (pAAV9-Col1a2-mCherry) were injected into the cisterna magna of P65$^{f/f}$ mice. Figure created with BioRender.com. **e,** Representative images assessing AAV9-mCherry tropism in leptomeningeal tissues by co-staining with fibroblast, endothelial, mural and immune cell markers. Scale bar, 20 µm. **f,** Left, representative images of type I/III collagen fibres in the leptomeninges of GBM tumour-bearing mice in the indicated groups. Scale bar, 50 µm. Right, quantification of type I/III collagen fibre area in the leptomeninges (mean ± s.d., n = 5 mice per group). **g,** Relative gadobutrol content detected in CSF from GBM tumour-bearing mice of the indicated genotypes (mean ± s.d., n = 5 mice per group). **h,** Left: Representative bioluminescence images of CT2A-Luc tumor-bearing mice in the control (Ctrl), anti-PD-1 monotherapy (aPD1), P65$^{f/f}$ Col1a2^{Cre}, and aPD1 + P65$^{f/f}$ Col1a2^{Cre} treatment groups. Right: Bar graphs showing tumor burden in these groups (mean ± s.d., n = 6 per group). **i,** Kaplan-Meier survival

curves of CT2A-Luc tumor-bearing mice in the Ctrl, aPD1, P65$^{f/f}$ Col1a2^{Cre}, and aPD1 + P65$^{f/f}$ Col1a2^{Cre} groups (n = 6 per group, Log-rank test). **j,** Representative fluorescence images of CD8^{+}IFN-γ^{+}PD1^{+} cells in the tumor tissues of the Ctrl, aPD1, P65$^{f/f}$ Col1a2^{Cre}, and aPD1 + P65$^{f/f}$ Col1a2^{Cre} groups (GL261-Luc model). Scale bar, 50 µm. **k-m,** Quantification of CD45^{+}CD8^{+} cells, PD1^{+} cells among CD45^{+}CD8^{+} T cells, and IFN-γ^{+} cells among CD45^{+}CD8^{+} T cells in the tumor tissues of CT2A-Luc tumor-bearing mice in the Ctrl, P65$^{f/f}$Col1a2^{Cre}, aPD1, and aPD1 + P65$^{f/f}$Col1a2^{Cre} groups (mean ± s.d., n = 4 per group).

8. c/gCSF infusions: These infusions take several days. Is c/gCSF stable for such a long time at body temperature? Please provide quality metrics (i.e., proteomics and stability measurements for Dgf15, Pdgfc, Pdgfd, Tnc, Postn, and Lgals9) for these experiments.

Response: We thank the reviewer for this important and technically well-taken concern.

1) Stability of c/gCSF during multi-day infusion at ~37 °C.
Because c/gCSF was delivered by continuous infusion over several days at ~37 °C, we agree that stability of key components must be supported by direct, time-matched measurements. We therefore performed a stability time course under infusion-relevant conditions (37 °C; sterile, low-binding conditions). We chose ELISA for these six predefined factors because it provides higher sensitivity and more quantitative measurement (via standard curves). **We quantified six representative secreted proteins (Gdf15, Pdgfc, Pdgfd, Tnc, Postn, and Lgals9) by ELISA at D0, D1, D3, D5, and D**7. Across this period, levels remained largely stable without a progressive decrease consistent with substantial degradation during the infusion window (Response Fig. 20a-f).

2) Quality metrics and broader compositional integrity.
We agree that single-analyte assays alone may miss broader compositional changes. As an orthogonal QC readout, **we assessed bulk protein integrity by gel permeation chromatography (GPC/size-exclusion) across the same incubation window**. The molecular-weight distribution profiles were highly similar over time, and the number-average molar mass showed no time-dependent shift (Response Fig. 20g-i), arguing against substantial proteolysis or aggregation during the infusion-relevant period.

We thank the reviewer again for prompting these additional quality control experiments, which strengthen the rigor and interpretability of the infusion studies.

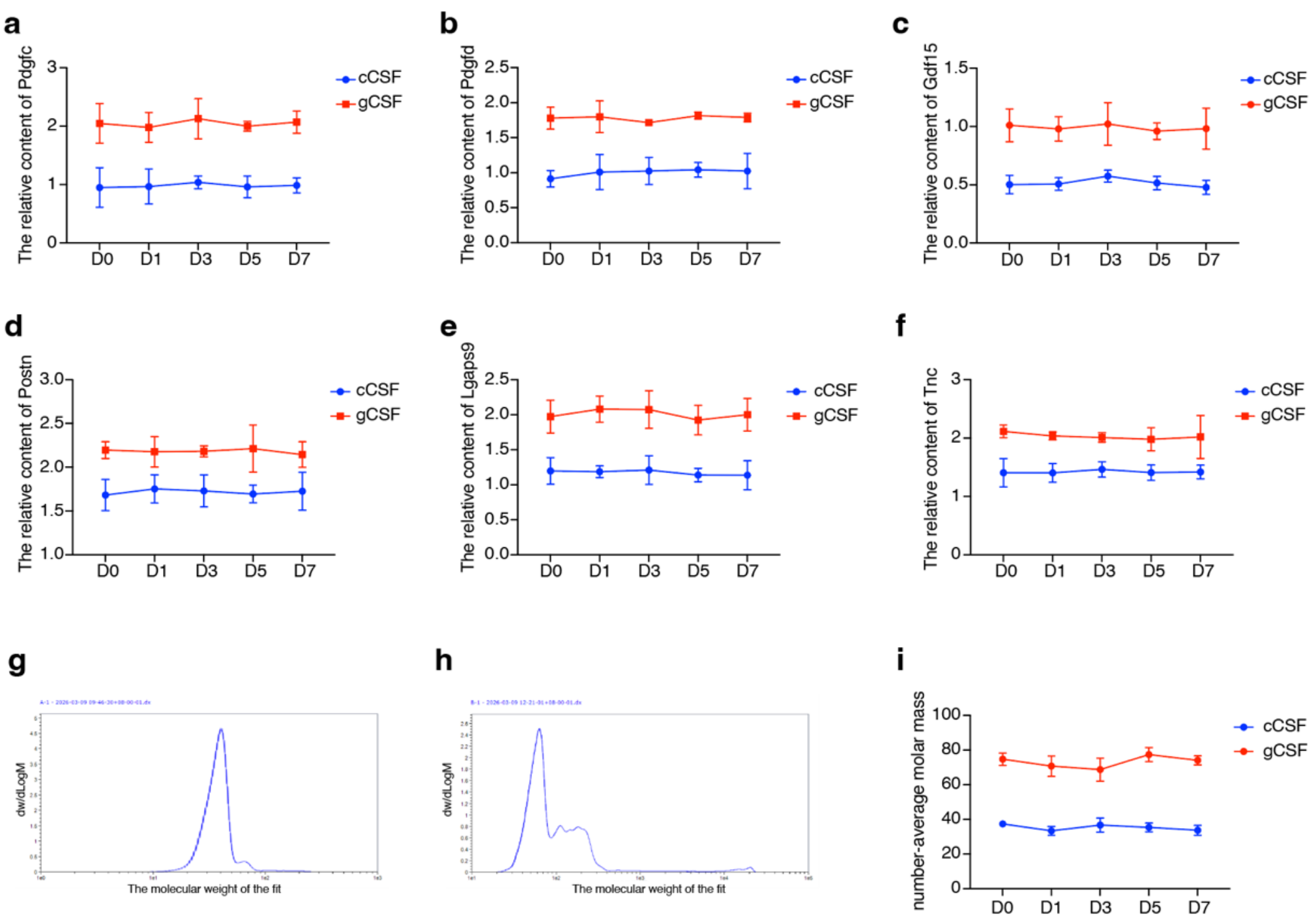

Response Figure. 20 | a-f, relative abundance of six representative secreted proteins in cCSF and gCSF; **g-h,** GPC profiles showing molecular-weight distribution; **i,** number-average molar mass (mean ± s.d.; n = 3 mice per group).

9. Impact on CD8 T cells: The performed experiments are somewhat correlative. What is the reason for increased CD8 T-cell activity? Is it more local T cell expansion, more infiltration, or better antibody penetrance? The mechanism has to be uncovered. It does not appear to be direct immunosuppression but rather T cell exclusion, a different phenomenon. The causal axis "leptomeninges - CSF - immunosuppression" is therefore incorrect, plus there would be no such causal axis with only these terms. This should be worded more precisely, focusing on the actual results rather than hypothetical mechanisms. And again, this must be repeated with at least one less immunogenic mouse GBM model.

Response: We thank the reviewer for this important and constructive critique regarding our $CD8^+$ T-cell conclusions. We agree that our original presentation was largely correlative and that the term "leptomeninges-CSF-immunosuppression axis" was overly causal and too imprecise given the data. In the revised manuscript, we therefore (i) refocus the text on results directly supported by the experiments, (ii) add analyses to better distinguish increased tissue entry from local expansion, (iii) explicitly address the possibility of antibody penetrance as a confounder, and (iv) repeat key findings in a less immunogenic GBM model.

1) **Distinguishing intravascular vs tissue $CD8^+$ T cells**:

To determine whether the increased $CD8^+$ T-cell signal reflects primarily enhanced vascular accumulation versus bona fide entry into tumor tissue, **we performed intravascular (i.v.) anti-CD45-PE labeling immediately prior to sacrifice[1,2]. After tumor dissociation, leukocytes were defined by CD45-FITC and stratified by i.v. CD45-PE into intravascular (i.v.+) and**

extravascular/tissue (i.v.-) compartments. Compared with the $P65^{f/f}$ group, the CSF-clearance–improved group ($P65^{f/f}$ $Col1a2^{cre}$) showed increased numbers of both i.v.+ ($CD45\text{-}FITC^{+}CD45\text{-}PE^{+}$) and i.v.- ($CD45\text{-}FITC^{+}CD45\text{-}PE^{-}$) $CD8^{+}$ T cells, with a more pronounced increase in the i.v.- (tissue) compartment (Response Fig. 21a-b). This pattern argues against a simple interpretation of "more $CD8^{+}$ cells confined to the vasculature" and is instead consistent with enhanced recruitment to the tumor vasculature together with increased extravasation/accumulation within tumor tissue, aligning more closely with reduced T-cell exclusion /a trafficking barrier.

The reviewer's point regarding "better antibody penetrance" is well taken. We clarify in the revised text that our primary $CD8^{+}$ T-cell quantification and activation-related readouts are derived from flow cytometry of dissociated tumor single-cell suspensions, which does not rely on antibody penetration through intact tissue; therefore, improved antibody penetrance is not an alternative explanation for the observed increase in $CD8^{+}$ T cells.

To assess whether local expansion contributes, we also **quantified Ki67 in intratumoral $CD8^{+}$ T cells and observed an increased $Ki67^{+}$ fraction** (Response Fig. 21c), indicating that local proliferation likely contributes to the increased tissue $CD8^{+}$ pool and activity. Importantly, because both i.v.+ and i.v.- compartments increase, we now frame the conclusion conservatively: the data support contributions from both enhanced recruitment/entry and local expansion, rather than a single dominant mechanism.

2) Mechanistic framing and revised wording:
In line with the reviewer's critique, we have replaced the "leptomeninges-CSF-immunosuppression axis" terminology and revised the relevant sections to emphasize the observed results. Specifically, improved CSF clearance is associated with changes in the vascular-versus-tissue partitioning and accumulation of tumor $CD8^{+}$ T cells, together with changes in proliferation/activation-related measures. We also explicitly state that the current study does not establish a single causal chain, and that additional targeted experiments will be required to uncover the underlying mechanisms.

3) Replication in a less immunogenic GBM model:
As requested, we repeated key immunological analyses in the less immunogenic SB28 GBM model. In this model, leptomeningeal PVS fibrosis and CSF clearance changes after tumor development were consistent with those observed in immunocompetent mice (Response Fig. 21d-e). Consistently, the CSF-clearance–improved group ($P65^{f/f}$ $Col1a2^{cre}$) exhibited increased $CD8^{+}$ T cells together with an increased frequency of $Ki67^{+}$ $CD8^{+}$ T cells (Response Fig. 21f-g). This replication supports the robustness of the $CD8^{+}$ T-cell phenotype beyond a single, potentially more immunogenic model.

We again thank the reviewer for these thoughtful and constructive suggestions. The additional experiments and the corresponding revisions in wording have substantially improved the rigor, precision, and interpretability of our conclusions regarding $CD8^{+}$ T-cell changes in this study.

Page 6, Lines 228-244 (**Results**): Notably, the proportion of T cells was significantly increased in P65$^{f/f}$ mice receiving pAAV9-Col1a2-Cre-mCherry (Fig. 4b). To determine whether the increased CD8$^+$ T-cell signal reflected preferential vascular retention or increased accumulation within tumour tissue, we performed intravascular anti-CD45-PE labelling immediately before sacrifice. After tumour dissociation, leukocytes were defined by CD45-FITC and stratified by intravenous CD45-PE into intravascular (CD45-FITC$^+$CD45-PE$^+$) and extravascular/tissue (CD45-FITC$^+$CD45-PE$^-$) compartments. Compared with P65$^{f/f}$ mice receiving control virus, P65$^{f/f}$ mice receiving pAAV9-Col1a2-Cre-mCherry showed increased numbers of both intravascular and extravascular/tissue CD8$^+$ T cells, with a more pronounced increase in the extravascular/tissue compartment (Extended Data Fig. 4e-f). This pattern is consistent with enhanced recruitment to the tumour vasculature together with increased accumulation within tumour tissue. To assess whether local expansion might also contribute to the enlarged intratumoral CD8$^+$ T-cell pool, we quantified Ki67 expression in tumour-infiltrating CD8$^+$ T cells. We observed an increased Ki67$^+$ fraction in the P65$^{f/f}$ mice receiving pAAV9-Col1a2-Cre-mCherry group (Extended Data Fig. 4g), suggesting that local proliferation may contribute to the expanded intratumoral CD8$^+$ compartment. Taken together, these data support contributions from both enhanced recruitment/entry and local expansion to the increased intratumoral CD8$^+$ T-cell population.

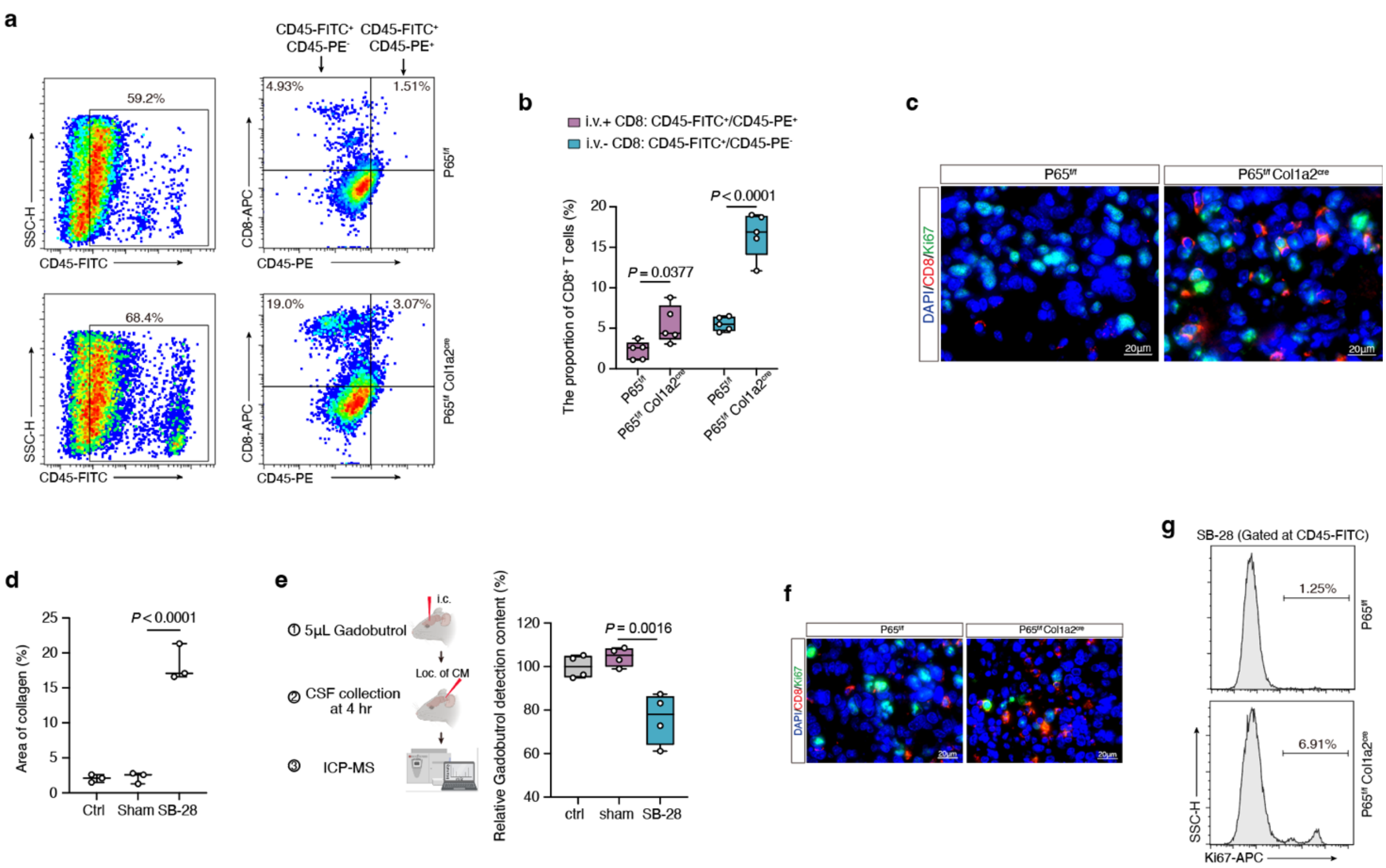

Response Figure. 21 | a, Representative flow cytometry plots. **b,** Quantification of numbers of both i.v.+ (CD45-FITC$^+$CD45-PE$^+$) and i.v.- (CD45-FITC$^+$CD45-PE$^-$) CD8$^+$ T cells (mean ± s.d., n = 5 per group). **c,** Immunofluorescence staining of Ki67 in intratumoral CD8$^+$ T cells in CT-2A GBM mice. **d,** Quantification of leptomeningeal type I/III collagen fibre area (COL1A1$^+$/COL3A1$^+$) in control mice, sham-operated mice and GBM tumour-bearing mice (mean ± s.d., n = 3 mice per group).

e, Left, schematic of intraparenchymal gadobutrol injection and cisterna magna CSF collection. Right, relative abundance of gadobutrol in cisterna magna CSF measured 3 h after intraparenchymal tracer injection (mean ± s.d., n = 5 mice per group). Figure created with BioRender.com. **f,** Immunofluorescence staining of $Ki67^+$ $CD8^+$ T cells in SB-28 GBM mice. Scale bar, 20 µm. **g,** Representative flow cytometry plots of $Ki67^+$ cells (gated at CD45-FITC).

Reference：

1. Anderson, K. G. et al. Intravascular staining for discrimination of vascular and tissue leukocytes. Nature protocols 9, 209-222 (2014).

2. Galletti, G. et al. Two subsets of stem-like CD8+ memory T cell progenitors with distinct fate commitments in humans. Nature immunology 21, 1552-1562 (2020).

Minor comments:

1. The reference number for local ethical approval (clinical samples) is missing, as is the mouse study approval.

Response: We thank the reviewer for noting the missing ethics approval information and apologize for this omission. In the revised manuscript, **we have added the full ethical compliance statements** for both the clinical samples and the animal studies. Specifically, collection and use of all human clinical samples and all mouse experiments were approved by the local ethics committee (Approval No.: FLYP-FE23207R).

2. Did the authors use male or female mice? The methods are confusing in this regard.

Response: We thank the reviewer for pointing out that the description of mouse sex was unclear in the Methods. All in vivo experiments in this study were performed using male mice (6-8 weeks old). This includes wild-type C57BL/6J mice as well as genetically modified (offspring) mice used in the study; only male animals were enrolled in experimental cohorts to minimize potential confounding from sex-related physiological differences and to improve consistency and reproducibility across experiments.

We have now explicitly stated "male mice" in the Abstract (as required by the editor) and **clarified this information in the Methods**, including the sex of both WT C57BL/6J mice and genetically modified mice used for experiments. We thank the reviewer again for this helpful suggestion.

Page 12, Lines 478-479 (**Methods**): All in vivo experiments were performed using male mice (6-8 weeks old), including wild-type C57BL/6J mice and genetically modified (offspring) mice.

3. How was the speed of c/gCSF infusion decided?

Response: We thank the reviewer for raising this point. The infusion rate for cCSF/gCSF was chosen based on (i) the technical specifications and validated performance of the osmotic pump used in this study, and (ii) physiological considerations to maintain slow, continuous ventricular exposure while

minimizing the risk of acute volume/pressure perturbations associated with bolus injections or high flow rates[1,2].

Specifically, we used an implantable osmotic pump (RWD Life Science, model 1001W; nominal reservoir volume 100 μL) designed to deliver a stable, near-constant flow in vivo. According to the manufacturer's specification and common practice for continuous intracranial delivery in mice, we used a fixed infusion rate of 0.5 μL/hr (i.e., 12 μL/day), which corresponds to an approximate 8.3-day continuous infusion when the reservoir is filled to capacity. We additionally verified the pump's release stability over time (Response Fig. 22).

This infusion speed was selected to 1) provide sustained and steady exposure over the experimental window without bolus-associated transient intracranial pressure changes or fluid accumulation; 2) remain within a commonly used range for mouse intracranial/ventricular infusion using osmotic pumps; and 3) in our hands, not produce observable adverse effects attributable to implantation or delivery speed. **We have clarified the pump model, delivery rate, and infusion duration/volume in the revised Methods section.** We appreciate the reviewer's helpful suggestion.

Page 13, Lines 524-534 (**Methods**): An osmotic pump (RWD Life Science, 1001W) was used to model CSF clearance impairment induced by gCSF. CSF was collected from the cisterna magna of healthy mice (control cCSF) and GBM-bearing mice, centrifuged to remove cellular debris, and stored until use. gCSF (or cCSF) was continuously infused into the lateral ventricle of recipient mice (stereotaxic coordinates: AP +0.5 mm; ML +1.0 mm; DV −2.5 mm) at a constant rate of 0.5 μL/hr using the osmotic pump implanted subcutaneously on the back. The pump reservoir (nominal volume 100 μL) provides ~8.3 days of infusion at this rate. Leptomeningeal fibrosis was assessed at indicated time points after completion of infusion (fluorescence staining procedures are described in "Histology and Morphology Analysis"). On day 5 after completion of infusion, an orthotopic CT2A-Luc/GL261-Luc tumor model was established for subsequent experiments.

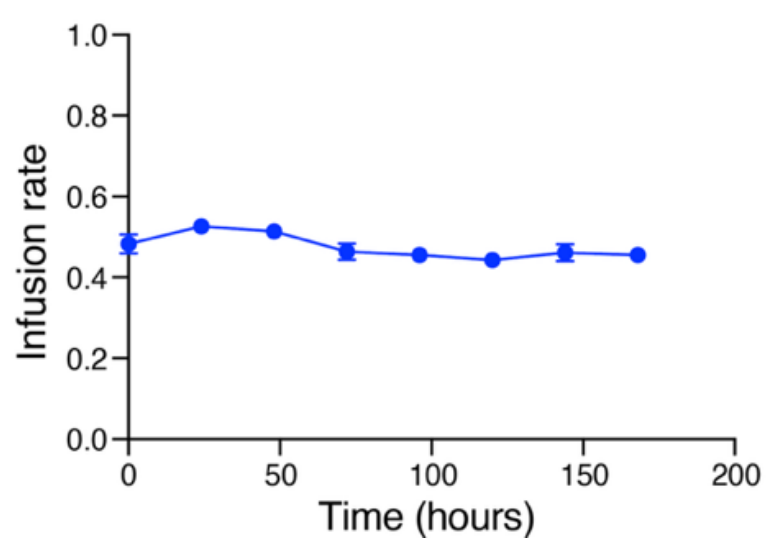

Response Figure. 22 | Stability of the osmotic pump infusion rate over time.

Reference:
1. Iram, T. et al. Young CSF restores oligodendrogenesis and memory in aged mice via Fgf17. Nature 605, 509-515 (2022).
2. Robert, S. M. et al. The choroid plexus links innate immunity to CSF dysregulation in hydrocephalus. Cell 186, 764-785. e721 (2023).

4. How was the dose of infusion pirfenidone calculated?

Response: We thank the reviewer for asking how the pirfenidone infusion dose and regimen were determined. Pirfenidone was delivered using an implantable osmotic pump (RWD Life Science, 1001W) with a fixed delivery rate of 0.5 μL/hr (i.e., 12 μL/day). Because pirfenidone has limited aqueous solubility, we prepared the infusate at 12 mg/mL. At this concentration and pump rate, the delivered amount was 12 mg/mL × 12 μL/day = 0.144 mg/day (144 μg/day), which corresponds to ~5.8 mg/kg/day for an average 25 g mouse.

The pump's nominal total capacity is 100 μL (maximum run time ~8.3 days at 0.5 μL/hr). In our experimental design, pirfenidone was infused for 3 days; therefore, we loaded only the volume required for this period (~36 μL, calculated as 0.5 μL/hr × 24 hr/day × 3 days),. The pump delivers at a constant rate independent of the fill volume, provided the loaded volume is within the rated capacity, ensuring stable and controlled delivery during the defined 3-day intervention window to assess the short-term effect of CSF-delivered pirfenidone on tumor-associated leptomeningeal fibrosis while avoiding unnecessary prolonged exposure.

We have revised the manuscript to report the concentration, infusion rate, and delivered amount/day accordingly. We thank the reviewer for this helpful comment.

Page 13, Lines 536-538 (**Methods**): For CT2A-Luc tumor-bearing mice, an osmotic pump (model 1001W, RWD Life Science) was implanted on day 7 post-tumor establishment to infuse pirfenidone (Yeasen, 53179-13-8) into the CSF at 12 mg/mL via a fixed delivery rate of 0.5 μL/hr for 3 days.

5. Which of the procedures (and how) directly interfere with the studied phenomena? I would assume that injections/collections/infusions perturb the normal clearance rates. How was this controlled for or corrected?

Response: We thank the reviewer for this important point. We agree that invasive procedures, including ventricular cannulation/infusion, osmotic pump implantation, cranial surgery, and CSF sampling, can transiently perturb CSF dynamics and clearance (e.g., via brief changes in intracranial pressure, local tissue irritation, or altered flow resistance).

In our study, the procedures most likely to directly interfere with CSF clearance include: (i) stereotaxic cannulation with osmotic pump implantation and continuous ventricular infusion; (ii) CSF collection; and (iii) associated cranial surgical manipulations. **To minimize and control for these potential confounders, we implemented the following measures:**

1）Matched surgical and infusion controls across groups.
All experimental groups (including controls and treatment groups) underwent the same stereotaxic workflow, cannula placement approach, pump implantation, infusion duration, and identical infusion rate. Control animals received vehicle (or cCSF) infusion with the same volume and rate. Thus, procedure-related effects on CSF dynamics should be systematic and comparable across groups, and are controlled for in between-group comparisons.

2）Slow, continuous infusion rather than bolus delivery.
We used a low-rate, constant infusion (0.5 µL/hr) rather than acute intracranial bolus injections to minimize abrupt intracranial pressure changes and large hydrodynamic disturbances, which are more likely to confound clearance measurements.

3）Use of sham controls and separation of surgical effects from tumor/CSF effects.
Where applicable, sham-operated controls were included to account for surgery-related changes, enabling separation of procedure-associated alterations from tumor-associated effects.

4）Consistency across independent readouts.
Our conclusions are supported by multiple independent endpoints assessed in separate cohorts, reducing the likelihood that the observed effects are driven solely by non-specific, procedure-induced perturbations of CSF clearance.

We are sincerely grateful to the reviewer for these careful, practical questions, they prompted us to clarify key methodological details and strengthen the rigor and transparency of the revised manuscript.

Reviewer #4 (Remarks to the Author):

General strengths
This study addresses an important unmet need and clinical problem.
The causal logic for the study is clear and compelling and was tested at various levels (human → mouse → cellular → molecular → pharmacologic → therapeutic).
There were multiple types of readouts of CSF clearance (ICP-MS, imaging, lymph node drainage).
The genetic specificity by fibroblast targeting was a major strength.
The immune checkpoint inhibitor and NF-κB subunit P65 inhibitor synergy was extremely compelling.

Response: We appreciate the reviewer's positive assessment of the study.

General areas for improvement
The Discussion should address:
CSF clearance might occur through multiple routes. Fibrosis might be one gatekeeper, but not the only one.

Response: We thank the reviewer for this important and forward-looking suggestion. We agree that CSF clearance is governed by multiple routes and regulatory layers, including (but not limited to) ventricular-subarachnoid circulation, perivascular/glymphatic transport, meningeal lymphatic drainage, and additional mechanisms that remain incompletely defined. Accordingly, leptomeningeal fibrosis may represent a relevant "gatekeeper" in this process, but it is unlikely to be the sole determinant of CSF clearance efficiency.

In the revised Discussion, we added text to explicitly place our findings within this broader context. We now clarify that the leptomeningeal fibrosis described in our study likely constitutes one important gatekeeping component that can restrict CSF clearance under GBM conditions, rather than a comprehensive explanation for all clearance deficits. We also discuss additional contributors that may act in parallel or synergistically depending on pathological context, such as changes in vascular pulsatility, alterations in meningeal lymphatic structure/function, and shifts in periventricular or subarachnoid fluid dynamics. Finally, we delimit the scope of our conclusions: our data support a model in which GBM-associated leptomeningeal fibrosis contributes to impaired CSF clearance, while not excluding other routes or mechanisms that may independently influence CSF clearance. We agree that future studies integrating these pathways will be essential for a more complete understanding of CSF homeostasis in disease.

Page 9, Lines 358-366 (**Discussion**): Under physiological conditions, CSF enters the brain parenchyma and exchanges with interstitial fluid via the arterial paravascular pathway, subsequently draining into the deep cervical lymph nodes and ultimately entering the systemic circulation through venous paravascular and meningeal lymphatic pathways. However, CSF clearance is mediated by multiple partially parallel routes, including perivascular/glymphatic transport, meningeal lymphatic drainage and arachnoid/villous absorption. Accordingly, impaired CSF clearance in GBM is likely multifactorial. While our data support leptomeningeal fibrosis as an important gatekeeping

mechanism that can restrict CSF outflow, they do not exclude additional contributors such as altered vascular pulsatility, meningeal lymphatic dysfunction or broader CSF hydrodynamic disturbances.

Other meninges resident cells may be responding to CSF, in addition to fibroblasts.
NF-κB subunit P65 is ubiquitous. In clinical practice, it may be difficult to target the NF-κB subunit P65 inhibitor to only GBM fibroblasts because the drug is not fibroblast-specific.

Response: We thank the reviewer for raising these important and clinically relevant points. We agree that, in addition to fibroblasts, multiple meningeal-resident cell types (e.g., immune cells, vascular-associated cells, and meningeal epithelial-like cells) may also respond to changes in CSF composition. Our study does not exclude contributions from these additional cell populations; rather, we focused on leptomeningeal fibroblasts because our data support their central role in fibrosis formation and the associated impairment of CSF clearance. **We have now made this broader perspective explicit in the Discussion.**

Regarding NF-κB signaling, we fully agree that the NF-κB subunit P65 is broadly expressed across many cell types, and systemic pharmacologic inhibition is therefore unlikely to achieve fibroblast-specific targeting in clinical practice. We have clarified in the revised manuscript that the fibroblast-specific genetic manipulation of P65 is primarily intended to establish mechanistic necessity, rather than to propose an immediately translatable fibroblast-restricted therapeutic strategy.

In the Discussion, we further note that more clinically actionable approaches may include: (i) cell type-targeted delivery platforms to modulate NF-κB signaling in fibroblasts; (ii) targeting downstream effector molecules that are more selectively induced in pathological fibroblasts; or (iii) indirectly attenuating NF-κB activation by modifying CSF-derived cues or the profibrotic microenvironment. While these strategies are beyond the scope of the present study, they outline potential paths for future translational development.

We thank the reviewer for highlighting the clinical feasibility and biological complexity, which helped us better define the scope and boundaries of our conclusions.

Page 10, Lines 420-426 (**Discussion**): At the same time, because NF-κB/P65 is ubiquitously expressed, the fibroblast-specific P65 genetic experiments presented here are intended to establish mechanistic necessity rather than an immediately translatable therapy. Future studies will need to develop cell-targeted delivery approaches or identify fibroblast-selective downstream effectors. In addition, although this study focuses on leptomeningeal fibroblasts, other meningeal-resident cell types, including immune, vascular-associated and meningeal epithelial-like cells, may also sense CSF-derived signals and contribute to CSF-related pathology.

+ Strengths
- Areas for improvement

Introduction
+

The mechanistic logic is strong and goes from a clinical observation, to structural explanation, to molecular changes, and finally therapeutic relevance.

Response: We appreciate the reviewer's constructive comments, which have helped improve the clarity and rigor of the manuscript.

-
Add one sentence with epidemiologic context.Firm statistics would strengthen the magnitude of the public health burden. For example, "most common" could be supplanted with incidence or prevalence, and "... the median survival of GBM patients remains severely limited" could be strengthened with a statistic. Also, it's not clear if these statements are based on global or national data.

Response: We agree that including quantitative epidemiologic data strengthens the rationale and avoids imprecise statements without context. Accordingly, **we revised the Introduction to include incidence and survival statistics** and now explicitly state that these estimates are derived from published datasets.

We thank the reviewer for this helpful suggestion.

Page 1, Lines 14-17 (**Introduction**): Glioblastoma (GBM) is a leading malignant primary central nervous system (CNS) tumor in adults, accounting for ~50% of malignant primary brain tumors in population-based datasets. Despite multimodal treatment including surgical resection, radiotherapy, and chemotherapy, the median overall survival remains ~14-16 months, and the 5-year survival rate remains below 17% worldwide.

Results
Figure 1: Human Studies + Mouse Experiments
+ Three individual CSF tracers were used to measure CSF clearance rates (gadobutrol, FITC-dextran, and ovalbumin-AF647).
+ The reduced CSF clearance data was observed even after tumor resection, suggesting a structural change (and not the tumor) may be the contributing factor.
+ The degree of fibrosis was negatively correlated with CSF clearance rates in their mouse models.
+ The treatment with an anti-fibrotic drug (i.e. pirfenidone) improved CSF clearance rates.
- Line 121: The bulk RNA-seq data reflects transcriptomic changes, and does not provide direct genetic variation or mutational evidence.

Response: We thank the reviewer for this important clarification. We agree that bulk RNA-seq captures gene expression changes (transcriptomic alterations) and does not provide direct evidence for genetic variation or somatic mutations at the DNA level. To avoid ambiguity, **we revised the relevant text to explicitly describe these findings as transcriptomic-level evidence.**

Page 2, Lines 63-64 (**Results**): To provide transcriptomic-level support for these findings, we performed bulk RNA sequencing ...

Page 5, Lines 167-169 (**Results**): ...gCSF stimulation of hPLMFs enriched transcriptional programmes related to NF-κB activation (Fig. 3a).

Figure 2: Human Studies
+ Pathological fibroblasts (CTHRC1$^+$and SMA$^+$) were higher in GBM patients.
+ Single-cell RNA-seq demonstrated higher expression of fibrotic markers and fibrosis-related pathways.
+ GBM-derived CSF induced a myofibroblast phenotype and pathological changes driven by reprogramming molecules (i.e. secreted and dysregulated proteins).

Figure 3: Human Studies + Mouse Experiments
+ The drug inhibition, siRNA knockdown, and conditional knockout of the NF-κB subunit P65NF-κB experiments supported the pathway induction.
+ The NF-κB subunit P65 knockout in fibroblasts did not affect CSF clearance in healthy mice, but CSF clearance improved in the conditional knockout mice.

Figures 4 + 5 + 6: Mouse Experiments
+ The fibroblast-specific P65 conditional knockout mice had reduced tumor burden and prolonged survival.
+ The above changes were associated with increased T cell infiltration and activation in the tumor microenvironment.
+ The replacement model recapitulated that gCSF caused reduced CSF clearance, exhibited faster tumor progression, and lower survival.
+ Reduced CSF clearance induces CD8$^+$ T cell exhaustion.
+ The anti-PD-1 and P65 inhibitor combination treatment dramatically reduced tumor size and prolonged survival.

Figure 6: Human Studies
+ CD8$^+$ T cell exhaustion was positively correlated with collagen-related pathways.

Response: We sincerely thank the reviewer for the thoughtful comments and constructive summary of our key findings across the human and mouse studies.

Discussion
+ The non-tumor leptomeninges come from epilepsy patients. While the authors acknowledge the inflammatory features of epilepsy, a brief discussion of how this might bias fibrosis or immune signatures should be added.

Response: We thank the reviewer for raising this important point regarding the source of our non-tumor leptomeningeal controls. We agree that leptomeninges obtained from epilepsy patients may exhibit a baseline inflammatory milieu, which could in principle bias fibrosis-related signals and/or immune transcriptional signatures relative to leptomeninges from individuals without epilepsy.

To minimize confounding, control tissues were obtained from neurosurgical resections without radiographic evidence of neoplastic disease, and GBM leptomeningeal specimens were reviewed to exclude direct tumor cell infiltration. However, we did not perform a dedicated quantitative assessment of subtle inflammatory cell infiltration in epilepsy leptomeninges (e.g., systematic immune-marker staining and/or inflammation scoring). Therefore, we cannot fully exclude that epilepsy-associated inflammation contributes to baseline ECM/immune signatures. **As suggested, we added a brief discussion explicitly acknowledging this potential bias and listing it as a study limitation.** We also note that future studies incorporating non-epilepsy control leptomeninges and/or additional histologic and molecular quantification of inflammation will further strengthen the conclusions.

Page 11, Lines 432-437 (**Discussion**): First, non-tumor leptomeningeal control tissues were obtained from epilepsy surgeries. Because epilepsy can be associated with chronic neuroinflammation, some baseline immune or extracellular matrix alterations in these leptomeningeal samples cannot be completely excluded, although neoplastic disease was excluded radiographically and tumor infiltration was excluded pathologically. Future studies incorporating additional non-epilepsy controls and quantitative assessment of inflammatory infiltrates will be valuable.

Methods
+
The fibroblast-specific P65 conditional knockout mice experiment was particularly strong.

Response: We sincerely thank the reviewer for all insightful and constructive comments, which have substantially improved the quality, clarity, and rigor of our manuscript.

Point-by-point response to the reviewers

REVIEWERS' COMMENTS

Reviewer #1 (Remarks to the Author)

I appreciate the authors'efforts to revise the manuscript. Several of my previous concerns have been addressed in a constructive manner. In particular, the authors now more clearly acknowledge that their tracer-based assays do not isolate a single CSF clearance route, and the revised interpretation of the post-resection data is more appropriate. The added clarification regarding tumor location and the distinction between local PC-MRI velocity metrics and global CSF clearance also improves the manuscript.

However, one key concern remains only partially addressed: the mechanistic interpretation of the apparent dissociation between increased ventricular CSF velocity and reduced clearance.

The authors'explanation that PC-MRI-derived velocity reflects local, plane-specific pulsatile motion rather than net CSF turnover is conceptually reasonable. However, the proposed mechanisms, including inefficient oscillatory flow, increased downstream outflow resistance, altered craniospinal compliance, and possible ICP-related changes, remain hypotheses rather than validated mechanisms. The current revision does not provide direct experimental or quantitative physiological evidence to support these interpretations.

The added ventricular size analysis is useful but insufficient to validate an ICP-related or obstructive high-pressure mechanism. A mild increase in third-ventricle width, in the absence of significant changes in the Huckman or Evans indices and without direct ICP measurements, does not establish CSF accumulation, hydrocephalus, elevated ICP, or ineffective oscillatory flow.

Therefore, I recommend that the authors either provide additional evidence supporting this interpretation or further temper the mechanistic language. Useful supporting analyses could include direct ICP measurements, quantitative PC-MRI parameters such as net flow, stroke volume, forward/reverse flow volumes, pulsatility index, or regurgitant fraction, or correlation analyses linking ventricular CSF velocity with tracer-based clearance, ventricular indices, tumor location, tumor volume, or ventricular proximity.

If such experiments or analyses are beyond the scope of the current study, the manuscript should explicitly state that the velocity-clearance dissociation is an observed association, and that ineffective oscillatory flow, altered compliance, increased outflow resistance, and ICP-related changes remain speculative explanations requiring future validation.

Overall, the revised manuscript is improved, but this mechanistic issue remains incompletely resolved. I would consider this point only partially addressed unless the authors either provide additional supporting evidence or substantially weaken the related mechanistic claims.

Response: We thank the reviewer for this important and constructive comment. We agree that our data do not directly establish the physiological mechanism underlying the apparent dissociation between increased ventricular CSF velocity and reduced tracer-based clearance. In particular, we did not measure ICP or more comprehensive PC-MRI flow parameters such as net flow, stroke volume, forward/reverse flow volumes, pulsatility index, or regurgitant fraction. **We therefore agree that the mechanistic interpretations proposed in the previous revision were stated too strongly.**

In response, we have substantially tempered the relevant language in both the Results and Discussion. In the Results section, we now emphasize that PC-MRI reports local, plane-specific CSF motion rather than whole-system CSF turnover or net clearance, and that increased local velocity should not be interpreted as increased global CSF

clearance. In the Discussion, we now explicitly state that the dissociation between ventricular CSF velocity and tracer-based clearance is an observed association in the current study, and that possible explanations such as inefficient oscillatory flow, altered craniospinal compliance, increased outflow resistance, or ICP-related changes remain speculative and require future validation. These changes are reflected in the revised text as follows:

Page 3, Lines 96-99 (**Results**): Because PC-MRI primarily reports local, plane-specific CSF motion rather than whole-system CSF turnover or net clearance, these findings indicate altered regional ventricular CSF dynamics in GBM. Notably, the increased local velocity metrics observed by PC-MRI should not be taken to imply increased global CSF clearance.

Page 9, Lines 370-381 (**Disscusion**): We also note that the apparent dissociation between increased ventricular CSF velocity and reduced tracer-based clearance should be interpreted cautiously. PC-MRI-derived velocity reflects local, plane-specific pulsatile motion and does not directly quantify net CSF turnover, effective solute transport, downstream outflow resistance, craniospinal compliance, or intracranial pressure. Therefore, although inefficient oscillatory flow, altered compliance, increased outflow resistance, or ICP-related changes could potentially contribute to this dissociation, our data do not directly test these mechanisms, and these possibilities remain speculative. Accordingly, in the current study, increased ventricular CSF velocity and reduced clearance should be regarded as an observed association rather than evidence for a defined hydrodynamic mechanism. Future studies incorporating direct ICP measurements and more comprehensive PC-MRI metrics, including net flow, stroke volume, forward and reverse flow volumes, pulsatility index, or regurgitant fraction, will be needed to resolve the underlying physiology.

Reviewer #2 (Remarks to the Author)
The authors have thoroughly addressed my concerns, now including acknowledging limitations of the study (e.g., generalizability, tumor subtypes), adding functional data for proteomics to demonstrate causality (PDGFR inhibition), including controls for fibroblast specificity, discussing potential clinical confounders, and clarifying statistical analyses.

Response: We thank the reviewer for the positive and encouraging assessment of our revised manuscript. We are pleased that these revisions have addressed the reviewer's concerns.

Reviewer #3 (Remarks to the Author)

I appreciate the effort the authors put into revising their manuscript. The mechanistic links are now much stronger, and I am happy to recommend it for publication, provided that the additional comments, limited to the scope of this revision, are addressed.

I have several additional comments:

1. All newly generated datasets must be deposited in public repositories, such as NCBI GEO, and released by the publication date at the latest, without restrictions, as is standard in the field. This includes single-cell transcriptomics in human and mouse, bulk RNA-seq, proteomics, etc.

Response: We thank the reviewer for this comment. **We have deposited all newly generated datasets in appropriate public repositories in accordance with the journal's checklist requirements**, and these data will be released without restriction by the date of publication at the latest. The relevant accession numbers have been added to the revised manuscript.

Page 22-23, Lines 917-949 (**Data Availability**): Raw mass spectrometry proteomics data of CSF samples from non-tumour controls and GBM patients have been deposited in the iProX database, which serves as a core member of the ProteomeXchange Consortium, under accession IPX0017901000 (https://www.iprox.cn/page/project.html?id=IPX0017901000). These proteomics data are publicly accessible without restriction.

The bulk RNA-seq data from non-neoplastic human leptomeninges used in this study were obtained as controlled-access third-party data from the Synapse platform (syn34512705; https://www.synapse.org) and are not publicly redistributable. The sequencing data generated in this study have been deposited in the National Genomics Data Center (NGDC) under BioProject PRJCA061682 (https://ngdc.cncb.ac.cn). The associated OMIX accession numbers are as follows:

Single-cell RNA-seq data of tumor tissues from CT2A tumour-bearing mice: OMIX016098 (https://ngdc.cncb.ac.cn/omix/release/OMIX016098) - publicly open;

Bulk RNA-seq data of human leptomeningeal tissues from GBM patients: OMIX017887 (https://ngdc.cncb.ac.cn/omix/release/OMIX017887) - controlled access;

RNA-seq data of human pia-arachnoid leptomeningeal fibroblast-like cells (hPLMFs) co-cultured with control CSF (cCSF) or GBM-derived CSF (gCSF): OMIX017890 (https://ngdc.cncb.ac.cn/omix/release/OMIX017890) - controlled access;

Single-cell RNA-seq data of leptomeningeal tissue from non-tumour controls and GBM patients: OMIX017990 (https://ngdc.cncb.ac.cn/omix/release/OMIX017990) - controlled access.

Consistent with Chinese human genetic resource regulations, the human RNA-seq datasets are deposited under controlled access. Access can be obtained by contacting the corresponding author (shizhifeng@fudan.edu.cn) with a brief description of the intended use and will be granted upon approval by the data owners under a data use agreement (see Data_Use_Agreement provided as Supplementary Files). To ensure full transparency and reproducibility, all processed summary data required to support the main conclusions, including differentially expressed genes (Supplementary Tables 1 and 3) and differential protein expression (Supplementary Table 2), are provided as Supplementary Information and constitute an independently verifiable resource. Source data underlying the figures are provided as a Source Data file. Additional data supporting the findings are available in the Article and Supplementary Information. All other data are available from the corresponding author upon reasonable request.

2. Human analyses are still quite correlative and minimalistic. These results should be interpreted with extreme caution, especially given that the main models used do not faithfully recapitulate that of human GBM, and only limited validation with less immunogenic lines was provided during the revision. These should be outlined in the study limitations section. Similarly, for the sex-specific findings, they used only male mice.

Response: We thank the reviewer for this helpful comment. We agree that the human analyses remain correlative and limited, and that they should be interpreted cautiously, especially because the mechanistic studies rely primarily on syngeneic mouse models that do not fully recapitulate human GBM heterogeneity. **We have therefore expanded the Discussion to explicitly acknowledge these limitations, including the limited validation in less immunogenic models. We also now state explicitly that all mechanistic experiments were performed in male mice and that potential sex-dependent effects remain unresolved.**

Page 11, Lines 446-459 (**Disscusion**): Second, our mechanistic evidence is derived primarily from the GL261 and CT-2A models together with correlative human datasets; therefore, the extent to which this CSF-leptomeningeal fibrosis axis generalizes across molecular GBM subtypes remains to be established in larger, subtype-stratified cohorts and additional models. Importantly, these syngeneic mouse models do not fully recapitulate the genetic, cellular and immunological heterogeneity of human GBM, and validation in less immunogenic models in the current study remains limited. Accordingly, the human analyses presented here should be interpreted cautiously, as they are associative, based on relatively limited clinical and pathological readouts, and not sufficient to establish causality. Although we controlled for age and sex, our clinical analyses remain susceptible to residual confounding by molecular subtype and treatment-related factors. Third, all mechanistic mouse experiments in the current study were performed in male mice. Therefore, potential sex-dependent differences in CSF dynamics, leptomeningeal remodeling, fibroblast activation and antitumor immunity were not addressed here. Future studies incorporating both male and female animals will be required to determine the generalizability of these findings across sex.

3. Bioluminescent measurements, quantified here as radiance, are not directly representative of tumor size per se but rather of tumor burden.

Response: We thank the reviewer for this important clarification. We agree that bioluminescent measurements quantified as radiance reflect tumor burden rather than tumor size per se. **We have revised the text accordingly throughout the manuscript.**

4. The current gating strategy seems to overestimate the CD45 infiltrate as well as the other immune fractions. Data has to be reanalyzed and replotted. Moreover, PD1 alone is not a marker of exhaustion. Intracellular TOX staining on top of PD1 would be more suitable.

Response: We thank the reviewer for this important comment and agree with the concern. In the original version of Extended Data Fig. 4e,f, the gating strategy appears to have overestimated the $CD45^+$ infiltrate. Therefore, based on the single-stained controls, **we reanalyzed the flow cytometry data and replotted Extended Data Fig. 4e,f accordingly**. After reanalysis, the proportion of $CD45^+$ infiltrating cells is now within the range reported in the literature. We thank the reviewer for pointing this out.

We also agree that PD-1 alone is not sufficient to define T-cell exhaustion, and that intracellular TOX staining in combination with PD-1 provides a more appropriate assessment of exhausted T cells. Therefore, **we repeated the**

flow cytometry analysis in tumors from the P65$^{f/f}$ and P65$^{f/f}$Col1a2^{Cre} groups using PD-1 together with intracellular TOX staining (PE anti-mouse TOX Antibody, Biolegend, 377122). **The results consistently show a reduced proportion of exhausted T cells in the P65$^{f/f}$ Col1a2^{Cre} group, which is in agreement with the PD-1^{+} results presented in the main text (gated on CD45^{+}CD8^{+} cells).**

We thank the reviewer for this valuable suggestion.

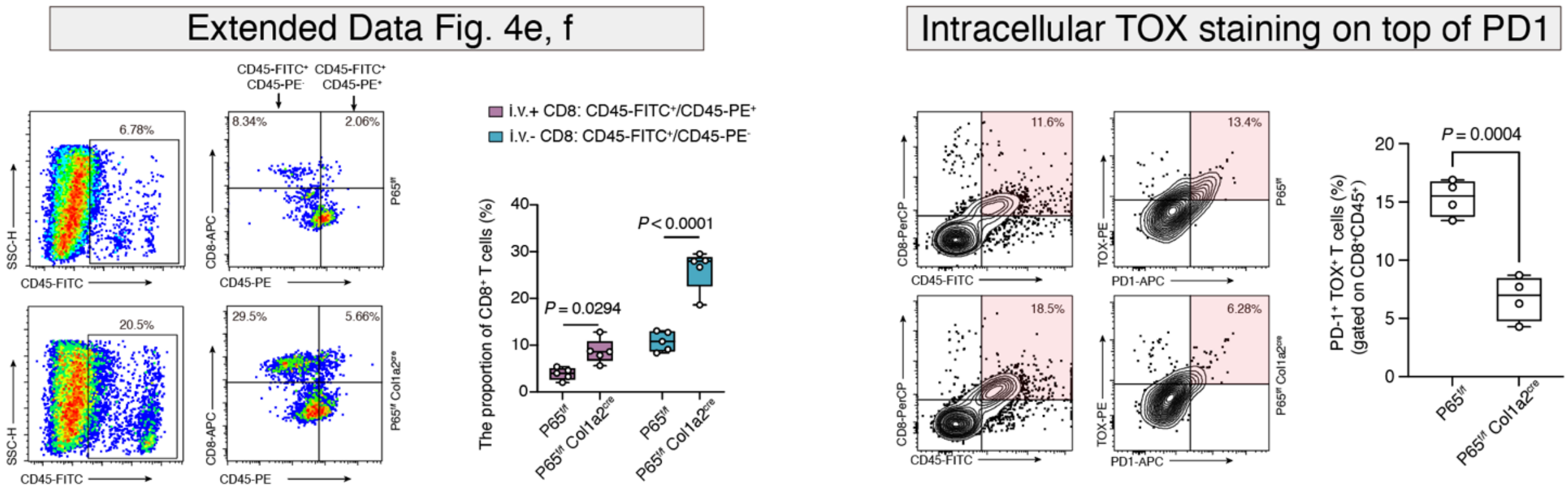

Response Figure. 1 | Left, reanalysis and replotting of Extended Data Fig. 4e,f based on the revised gating strategy; *P*-values, one-way ANOVA with Tukey's test. Right, representative flow cytometry plots and quantification of PD-1^{+} TOX^{+} cells (gated on CD45^{+} CD8^{+} cells; n = 4); *P*-values, unpaired two-tailed Student t-test.
